# Single and double activation of acetone by isolobal B≡N and B≡B triple bonds†
†Electronic supplementary information (ESI) available: General experimental details, characterization data for all reported compounds and details of the DFT calculations. CCDC 1830168–1830171 and 1830420. For ESI and crystallographic data in CIF or other electronic format see DOI: 10.1039/c8sc01249k


**DOI:** 10.1039/c8sc01249k

**Published:** 2018-04-30

**Authors:** Julian Böhnke, Tobias Brückner, Alexander Hermann, Oscar F. González-Belman, Merle Arrowsmith, J. Oscar C. Jiménez-Halla, Holger Braunschweig

**Affiliations:** a Institut für Anorganische Chemie , Julius-Maximilians-Universität Würzburg , Am Hubland , 97074 Würzburg , Germany . Email: h.braunschweig@uni-wuerzburg.de; b Institute for Sustainable Chemistry & Catalysis with Boron , Julius-Maximilians-Universität Würzburg , Am Hubland , 97074 Würzburg , Germany; c Departamento de Química , Universidad de Guanajuato , Noria Alta S/N , 36050 Guanajuato , México . Email: jjimenez@ugto.mx

## Abstract

B≡N and B≡B triple bonds induce C–H activation of acetone to yield a (2-propenyloxy)aminoborane and an unsymmetrical 1-(2-propenyloxy)-2-hydrodiborene, respectively. DFT calculations showed that, despite their stark electronic differences, both the B≡N and B≡B triple bonds activate acetone *via* a similar coordination-deprotonation mechanism. In contrast, the reaction of acetone with a cAAC-supported diboracumulene yielded a unique 1,2,3-oxadiborole, which according to DFT calculations also proceeds *via* an unsymmetrical diborene, followed by intramolecular hydride migration and a second C–H activation of the enolate ligand.

## Abstract 2

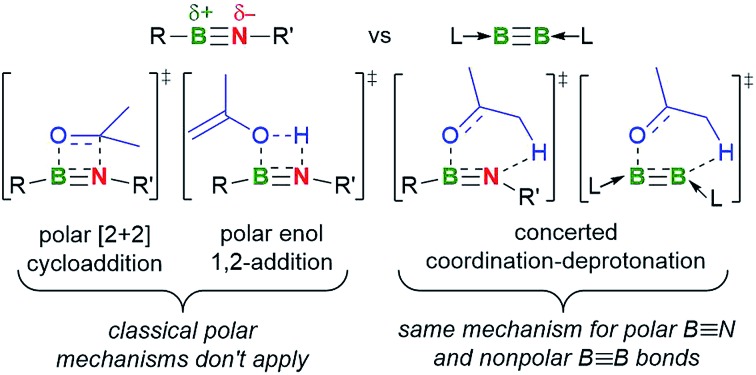
Polar and apolar boron-based triple bonds promote the single and double C–H activation of acetone following similar coordination-deprotonation mechanisms.

Due to their intrinsic electron deficiency, linear compounds containing a multiply bonded, sp-hybridised boron atom are far more reactive and difficult to isolate than isolobal carbon-based compounds. Owing to their ease of derivatisation, monomeric iminoboranes of the form RB≡NR′ (R, R′ = anionic substituents),^1^ which are formally isoelectronic to alkynes,^2^ have been the most widely studied class of two-coordinate boron compounds.^3^ The strong polarisation of the B≡N bond enables their participation in a vast array of spontaneous [2 + 2] cycloaddition^4^ and 1,2-addition reactions^5^ with polar substrates inaccessible to their alkyne counterparts. Only recently has our group shown that, with a suitable transition metal catalyst, iminoboranes can undergo [2 + 2] and [2 + 4] cycloaddition reactions with nonpolar alkynes.^6^

While linear RB≡NR′ compounds have been studied for over 30 years, isolobal LB≡BL compounds (L = neutral donor ligand) displaying two dicoordinate, zero-valent boron atoms long eluded isolation. Since our report of the first stable diboryne, (IDip)B≡B(IDip) (**I**, IDip = 1,3-bis(2,6-diisopropylphenyl)-imidazolidin-2-ylidene),^7^ we have shown that, by varying the π acceptor ability of L, the electronics and reactivity of these compounds can be fine-tuned.^8^,^9^ Thus, whereas unsaturated N-heterocyclic carbene (NHC)-supported diborynes such as **I** are inert towards H_2_,^10^ (SIDep)B≡B(SIDep) (**II**, SIDep = 1,3-bis(2,6-diethylphenyl)-4,5-(dihydro)imidazolidin-2-ylidene), which is supported by saturated NHCs of intermediate π acidity,^11^ adds H_2_ at 80 °C to yield a 1,2-dihydrodiborene.^10^ In turn, the use of even stronger π-accepting cyclic (alkyl)(amino)carbenes (cAACs)^12^ yields the cumulenic species (^Me^cAAC)···B=B···(^Me^cAAC) (**III**, ^Me^cAAC = 1-(2,6-diisopropylphenyl)-3,3,5,5-tetramethyl-pyrrolidin-2-ylidene),^13^ which, unlike **I** and **II**, activates H_2_ at room temperature^10^ and undergoes spontaneous [2 + 2] and [2 + 4] cycloadditions with acetylene.^14^

Intrigued by the seeming lack of reactivity overlap between isolobal linear RB≡NR′ and LB≡BL species (Fig. 1), we were eager to investigate whether the diboron compounds undergo spontaneous polar cycloaddition reactions similar to those of iminoboranes. Herein we compare the reactivity of **I–III** and a highly sterically hindered iminoborane, (TMP)B≡NAr* (**IV**, Ar*= (2,6-(CHPh_2_)_2_-4-*t*BuC_6_H_2_); TMP = 2,6-tetramethylpiperidyl), towards acetone and show that, despite their marked electronic differences, compounds **II** and **IV** activate acetone following a similar mechanism, whereas cumulene **II** promotes an unprecedented spontaneous double activation of acetone.

**Fig. 1:**
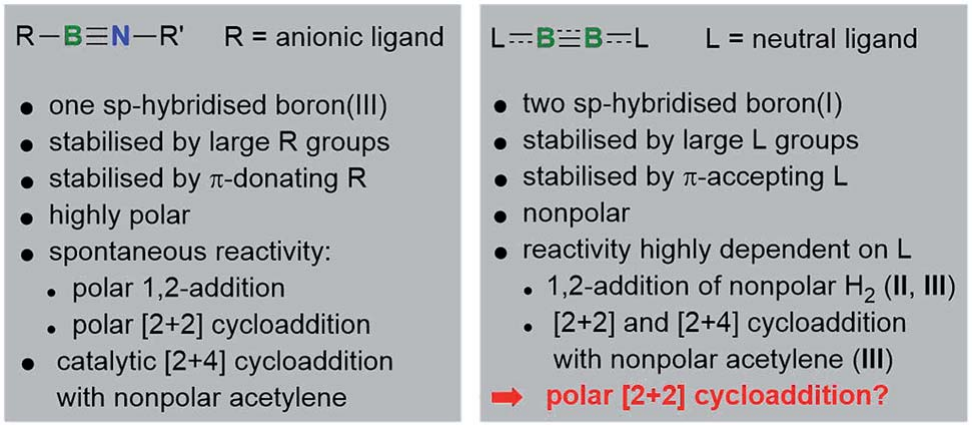
Side-by-side comparison of iminoboranes and diborynes.

Heating a suspension of **IV** in hexanes with excess acetone overnight at 70 °C resulted in clean formation of the (2-propenyloxy)aminoborane **1** (Scheme 1A). ^11^B NMR data of **1** showed a resonance at 24.8 ppm, while the ^1^H NMR spectrum displayed a NH singlet at 3.49 ppm and two characteristic 1H resonances for the terminal methylidene protons of the enolate ligand at 4.36 and 4.11 ppm.

**Scheme 1:**
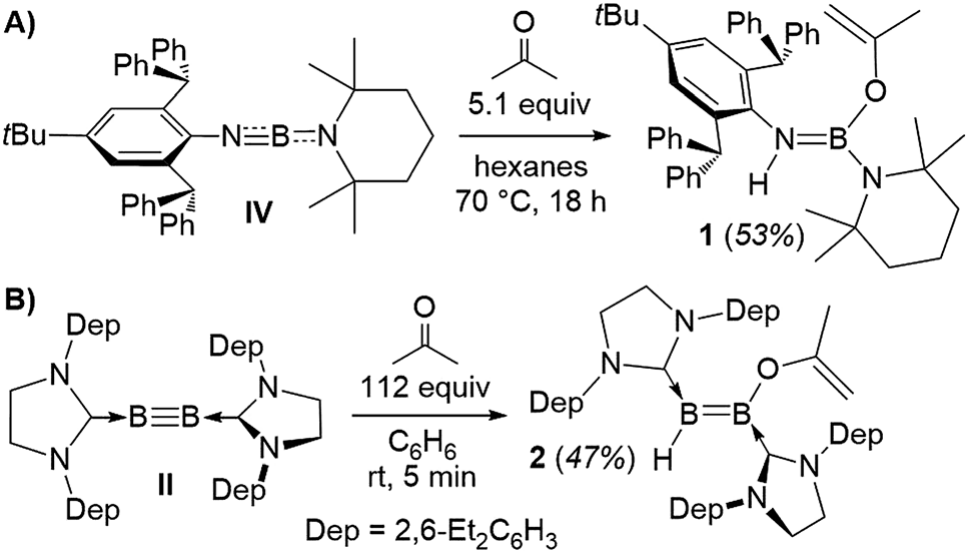
Enolic activation of acetone by (A) iminoborane **IV** and (B) diboryne **III**. Dep = 2,6-diethylphenyl.

X-Ray crystallographic analysis of **1** (Fig. 2) confirms the sp^2^-hybridisation at B1 (Σ(∠B1) = 359.9(18)°) and N1 (B1–N1–C4 126.92(16)°) as well as the elongation of B1–N1 to a single bond (1.424(3) Å). The 2-propenyloxide ligand coordinated to B1 displays a C1–O1 single bond (1.343(2) Å) and a terminal C1=C2 double bond (1.320(3) Å). Formally, compound **1** results from the addition of 2-propenol, the enol form of acetone, across the polar B≡N triple bond of iminoborane **IV**. While there is, to our knowledge, no literature precedent for the reactivity of monomeric iminoboranes with enolisable ketones, the dimeric iminoborane [BuB≡N*t*Bu]_2_ has been shown to undergo 1,4-enol addition to the ring-opened iminoborane dimer with acetone, acetophenone and 3,3-dimethylbutan-2-one.^15^ This contrasts with the reactivity of iminoboranes towards aldehydes4*d* and CO_2_,4*c* which yields the 1,3,2-oxazaboretidine [2 + 2] cycloaddition products.

**Fig. 2:**
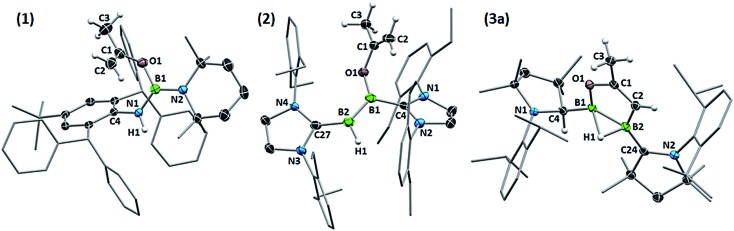
Crystallographically determined solid-state structures of **1**, **2** and **3a**. Atomic displacement ellipsoids depicted at the 50% probability level. Atomic displacement ellipsoids of peripheral substituents omitted for clarity. Hydrogen atoms omitted, except for those of the activated acetone moieties, the protonated cAAC carbon atom and those bound to boron. Selected bond lengths (Å): (**1**) B1–N1 1.424(3), B1–N2 1.429(3), B1–O1 1.418(3), O1–C1 1.343(2), C1–C2 1.320(3); (**2**) B1–C4 1.574(4), B2–C27 1.523(4), B1–B1 1.599(4), B2–H1 1.09(2), B1–O1 1.455(3), O1–C1 1.352(3), C1–C2 1.316(4); (**3a**) B1–C4 1.5295(19), B2–C24 1.5295(19), B1–B2 1.721(2), B1–H1 1.485(17), B2–H1 1.213(16), B1–O1 1.4064(17), O1–C1 1.3824(15), C1–C2 1.3301(18), C2–B2 1.5743(18).

Whereas diboryne **I** proved unreactive towards acetone even under forcing conditions, diboryne **II** reacted rapidly with excess acetone in benzene at room temperature to yield the green-coloured 1,2-enol addition product **2** (Scheme 1). Compound **2** presents two broad ^11^B NMR resonances at 38.1 and 19.3 ppm in a 1 : 1 ratio, attributable to the BH and the BOC_3_H_5_ moieties of the unsymmetrical diborene, respectively. The ^1^H NMR spectrum displayed two inequivalent SIDep ligands, as well as the inequivalent terminal methylene protons of the 2-propenyloxide ligand at 3.93 and 3.47 ppm. X-Ray crystallographic analysis of **2** showed a *trans*-1-alkoxy-2-hydrodiborene with a B–B double bond of 1.599(4) Å similar to that of its dihydrodiborene relative, (SIDep)HB=BH(SIDep) (1.589(4) Å).^10^ The SIDep ligand at the BH moiety is near coplanar with the diborene core (torsion (N4, C27, B2, B1) 12.1(5)°) and displays a short B2–C27 bond (1.523(4) Å), indicative of π backdonation. In contrast, the SIDep ligand supporting the BOC_3_H_5_ moiety is twisted *ca.* 35.5° out of the diborene plane and displays a pure σ-donor interaction (B1–C4 1.574(4) Å). The planar 2-propenyloxide ligand lies at a *ca.* 58° angle with respect to the diborene plane, and its bond lengths (O1–C1 1.352(3), C1=C2 1.316(4) Å) are similar to those of **1**. With Kinjo and co-workers recently reporting the first diborene with two different donor ligands^16^ and our group having just published the first fully unsymmetrical diborene,^17^ compound **2** is only the second unsymmetrical diborene with respect to the anionic substituents.

TDDFT calculations performed upon the optimised geometry of **2** at the (smd: *n*-pentane)lc-ωPBE/6-311+g(d) level of theory provided a maximum UV-vis absorbance at 592 nm (see Table S1 and Fig. S24 in the ESI†), which is in good agreement with the experimentally measured absorbance maximum in pentane at 605 nm (Fig. S16†). This corresponds to the HOMO–LUMO transition from the π-bonding orbital of the B=B double bond into the empty p_*z*_ orbital of the carbene carbon of the SIDep ligand supporting the BOC_3_H_5_ moiety, and is responsible for the blue-green color of the compound.

Surprisingly, the 1 : 1 reaction of diboracumulene **III** with acetone did not yield the expected cAAC analogue of **2**. Instead, ^11^B NMR data revealed a 92 : 8 mixture of two sp^2^–sp^3^ diborane products, the major one (**3a**) showing two broad singlets at 42.8 (full width at half maximum: fwhm ≈ 370 Hz) and –1.9 ppm (fwhm ≈ 130 Hz), and the minor (**3b**) presenting a very broad resonance at 63.0 ppm (fwhm ≈ 630 Hz) and a broad *B*H doublet at –15.0 ppm (^1^*J*_B–H_ = 50.8 Hz), suggesting a non-bridging hydride. The ^1^H NMR spectrum of the mixture showed very similar sets of resonances for **3a** and **3b**, which strongly suggests an isomeric relationship. Both compounds display one neutral cAAC ligand and one C1-protonated cAAC ligand (*δ* = **3a** 4.02, **3b** 4.24 ppm) as well as a single 1H alkene resonance (*δ* = **3a** 3.50, **3b** 3.78 ppm) (Scheme 2).

**Scheme 2:**
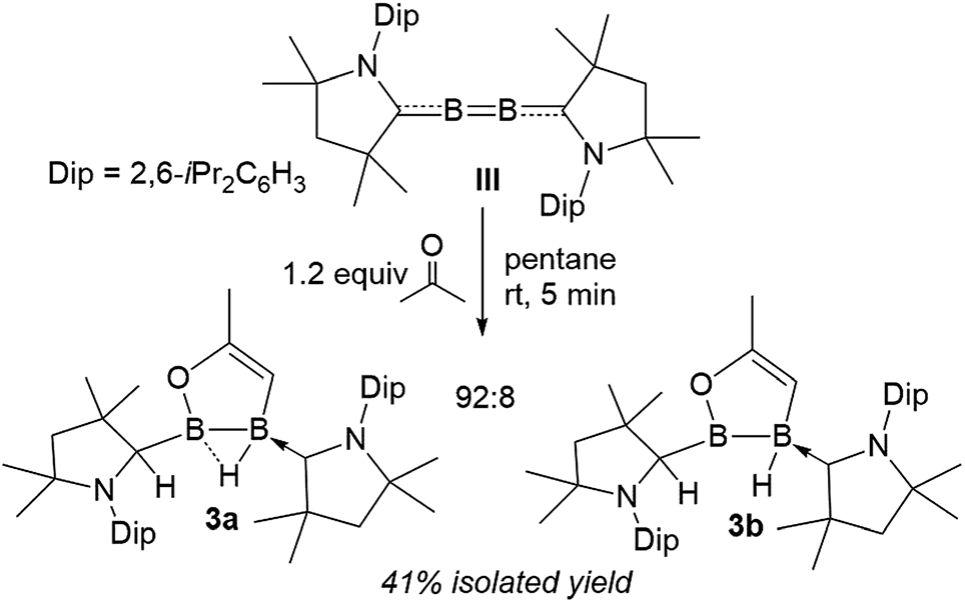
Double activation of acetone by diboracumulene **III**.

Single-crystal X-ray crystallography revealed a unique planar 2,3-dihydro-5-methyl-1,2,3-oxadiborole heterocycle displaying an endocyclic C1=C2 double bond (1.3301(18) Å, Fig. 2). The B–B bond is unsymmetrically μ^2^-bridged by a hydride (B1–B2 B1–H1 1.213(16), B2–H1 1.485(17) Å) positioned orthogonally to the B_2_C_2_O heterocycle (torsion (H1, B2, B1, C2) 105.7(9)°) and shows a bond length of 1.721(2) Å typical of a diborane (**5**). The alkenylborane moiety around B1 is supported by a neutral cAAC ligand with a relatively short B1–C4 bond (1.5295(19) Å) and forms an angle of only *ca.* 19° with the plane of the B_2_C_2_O heterocycle (torsion (N1, C4, B1, C2) 14.4(2)°), which is indicative of π conjugation. The enoxyborane moiety around B2 bears a protonated cAAC ligand displaying clear sp^3^-hybridisation at C24 (B2–C2 4 1.6045(18), C24–N2 1.4878(16) Å). The structure of **3a** is reminiscent of the products obtained from the reduction of (SIMes)BBr_2_BAr_2_ diborane (**5**) precursors (SIMes = 1,3-Mes_2_-4,5-dihydroimidazol-2-ylidene, Mes = 2,4,6-trimethylphenyl; Ar = Mes, 9-anthryl). These display a central, μ^2^-hydride-bridged, planar B_2_C_5_ heterocycle, resulting from the C–H activation of one aryl substituent by an intermediate boraborylene, and coordinated on one side by a neutral SIMes ligand and on the other by the second aryl substituent.^18^

Although single crystals of the minor species in solution were never obtained, the propensity for cAAC-supported hydroboranes to undergo 1,2-hydrogen shifts from boron to an adjacent cAAC carbene centre, which has been demonstrated both experimentally and computationally,^19^ first prompted us to identify the second isomer as compound **3_taut_**, a tautomeric form of **3a**, in which the neutral cAAC ligand coordinates to the enoxyborane moiety, and the protonated cAAC ligand coordinates to the alkenylborane moiety (Fig. 3). DFT optimisations at the ONIOM(M06-2X/6-311+G(d):PM6) level (see ESI† for details) showed, however, that **3_taut_** is 8.4 kcal mol^–1^ higher in energy than **3a** and that its calculated ^11^B NMR shifts (*δ* = 45.1, 6.1 ppm) do not fit the experimental data (*δ* = 63.0, –15.0 ppm). Since **3a** presents two stereocentres, one at B2, which is locked by the B_2_C_2_O ring and the asymmetrically bridging hydride, and one at the protonated cAAC carbon atom, the other possibility is that **3a** and **3b** could be diastereomers. This would also fit the observation that they do not exchange in solution even at high temperatures. To test this, the geometries and ^11^B NMR chemical shifts of the possible diastereomeric pairs derived from **3a** were computed (Fig. 3).

**Fig. 3:**
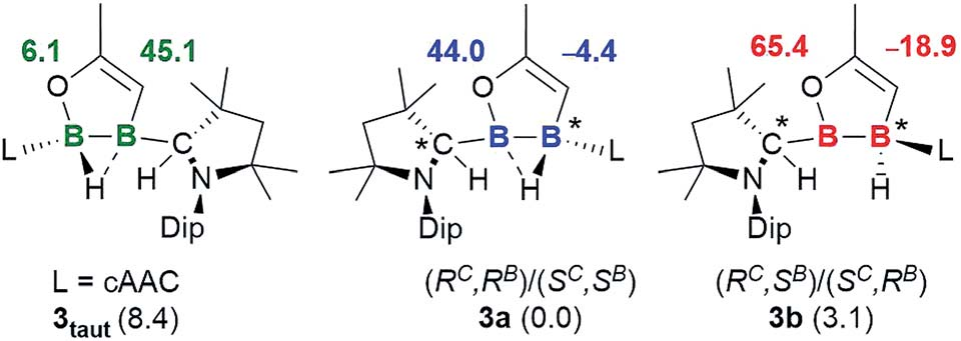
Calculated ^11^B NMR shifts (ppm, in bold) for the optimised structures of tautomer **3_taut_**, and the diastereomeric pair **3a** and **3b** at the ONIOM(M06-2X/6-311+G(d):PM6) level. Experimental shifts for comparison: **3a**: *δ* = 42.8 and –1.9 ppm; **3b**: *δ* = 63.0 and –15.0 ppm. Relative energies (kcal mol^–1^) in brackets.

The predicted ^11^B NMR chemical shifts for the (*R*^C^,*R*^B^)/(*S*^C^,*S*^B^)-**3a** pair (*δ*_calc_ = 44.0, –4.4 ppm) adequately match the experimentally-observed shifts (*δ*_exp_ = 42.8, –1.9 ppm; Δ(*δ*) ≈ ±2 ppm). Calculations on the diastereomeric pair showed that a form with a non-bridging hydride is the most likely. This also correlates well with the observation that, unlike **3a**, which shows two very broad ^11^B NMR resonances typical for a μ^2^-hydride-bridged diborane, **3b** shows a doublet at –15.0 ppm (^1^*J*_11B–1H_ = 50.8 Hz), indicating a terminal hydride rather than a bridging one. The predicted ^11^B NMR chemical shifts for the (*R*^C^,*S*^B^)/(*S*^C^,*R*^B^)-**3b** pair (*δ*_calc_ = 65.4, –18.9 ppm) are comparable to the experimental ones (*δ*_exp_ = 63.0, –15.0 ppm; Δ(*δ*) ≈ ±3 ppm). The relative energy of (*R*^C^,*S*^B^)/(*S*^C^,*R*^B^)-**3b**, at 3.1 kcal mol^–1^ above (*R*^C^,*R*^B^)/(*S*^C^,*S*^B^)-**3a**, is consistent with the experimentally observed ratio of 92 : 8.

The spontaneous formation of **3a**/**b** is particularly remarkable in view of the fact that there is seemingly no literature precedent for a one-step, uncatalysed, 100% atom-efficient double C–H activation of acetone or other enolisable ketones. We were therefore keen to investigate the mechanism of the formation of **3a**/**b** and compare it to that of the boron enolates **1** and **2**. While the reaction of dimeric iminoboranes with enolisable ketones always yielded the 1,4-enol addition products, Paetzold and co-workers showed that with acetophenone, which is less prone to enolisation, a [2 + 4] cycloaddition product can also be isolated.^15^ However, it remained unclear whether or not the latter is an intermediate to the former. For comparison, nonpolar disilenes are known to first undergo [2 + 2] cycloaddition with acetone and acetophenone to form the corresponding 1,2,3-oxadisiletane heterocycles, which then rearrange to the 1,2-enol addition products.^20^ In our case, however, careful monitoring of the reaction of iminoborane **IV** and diboryne **II** with acetone showed no evidence of [2 + 2] cycloaddition products or intermediates.

DFT calculations carried out at the D3-PBE0/6-31G(d) level for **IV** and at the ONIOM(M06-2X/6-311+G(d):PM6) level for **II** and **III** showed that acetone activation does not proceed *via* 1,2-enol addition, as the enol form of acetone lies 15.3 kcal mol^–1^ higher than the reactants, well above the activation energy for direct acetone addition (Fig. 4, see ESI† for details on the methodology and the optimised structures of all reactants, products, intermediates and transition states).

**Fig. 4:**
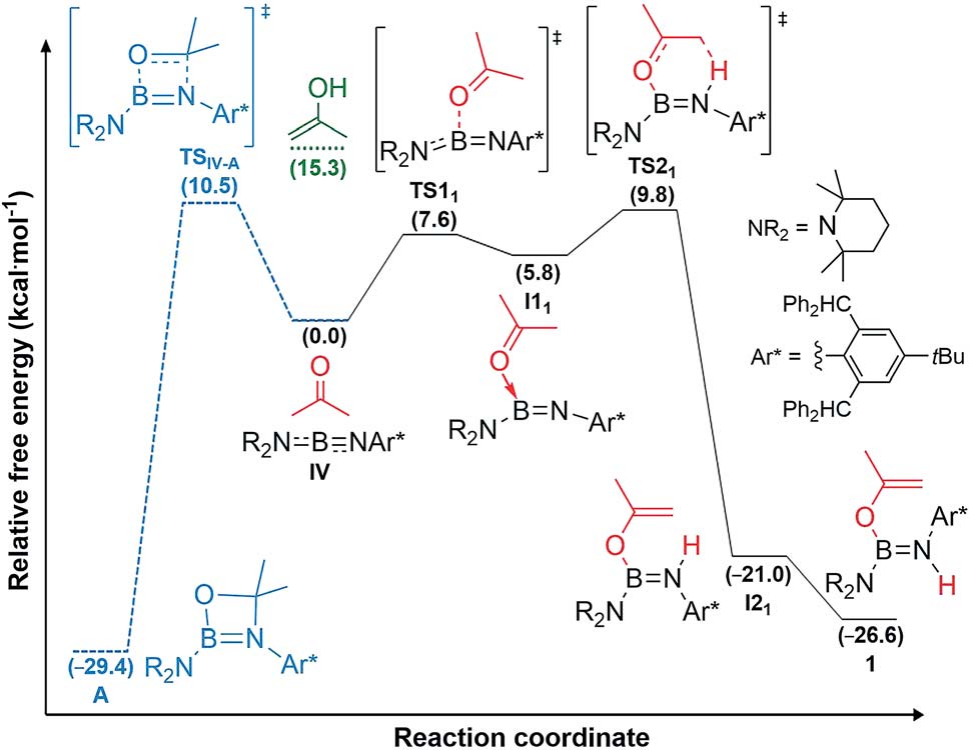
Mechanisms of acetone addition to iminoborane **IV** to yield aminoborane **1** (straight lines in black) and alternative [2 + 2] cycloaddition to yield **A** (dashed lines in blue), as well as energy level of the enol form of acetone (green) calculated at the D3-PBE0/6-31G(d) level of theory. Gibbs free energies (kcal mol^–1^) in brackets.

For iminoborane **IV** two plausible mechanisms were investigated, the first *via* a 4,4-dimethyl-1,3,2-oxaboretidine [2 + 2] cycloaddition product (**A**), the second *via* concerted acetone coordination-deprotonation (Fig. 4). Although the cycloaddition product **A** is calculated to be more stable than **1** by 2.8 kcal mol^–1^, the energy barrier for the formation of **A** is slightly higher than for **1**.§
§Since the calculated difference in energy barrier for products **1** and **A** is so low, the reaction of iminoborane **IV** with acetone was also carried out at higher temperatures to see if **A** could be obtained instead. However, this only led, beside the formation of **1**, to an accumulation of intractable decomposition products. Furthermore, as there is no thermodynamically viable reaction path from **A** to **1**, a [2 + 2] cycloaddition mechanism followed by rearrangement to **1** can be ruled out.

Instead, for compounds **II–IV** the first reaction step involves coordination of the carbonyl oxygen atom to one boron centre to form the acetone adducts **I1_1_**, **I1_2_** and **I1_3_** (Δ*G*‡1 = 7.6 (**IV**), 20.6 (**II**), 10.1 (**III**) kcal mol^–1^), respectively (Fig. 4 and 5). This step is followed in all three cases by C–H activation of one of the pendant methyl groups of the coordinated acetone by either the nitrogen atom (for **IV**) or the electron-rich, second boron centre (for **II** and **III**), to yield the *cis*-aminoborane **I2_1_**, and the SIDep- and cAAC-supported *cis*-diborenes **I2_2_** and **I2_3_**, respectively (Δ*G*‡2 = 4.0 (**IV**), 14.1 (**II**), 14.9 (**III**) kcal mol^–1^). Finally, the *trans*-aminoborane **1** and the *trans*-diborenes **2** and **I4_3_** are obtained by rotation around the B–N and B–B bond, respectively. Overall, the formation of **2** presents the highest energy barrier and is also the most exergonic (Δ*G* = –43.2 kcal mol^–1^), followed by that of **1** (Δ*G* = –26.6 kcal mol^–1^) and **I4_3_** (Δ*G* = –27.4 kcal mol^–1^).

**Fig. 5:**
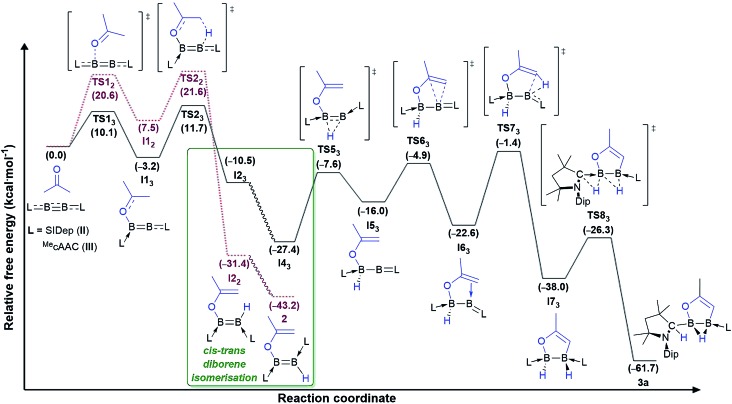
Mechanisms of double acetone activation by diboryne **II** (dotted lines in purple) and cumulene **III** (straight lines in black) calculated at the ONIOM(M06-2X/6-311+G(d):PM6) level of theory. Gibbs free energies (kcal mol^–1^) in brackets. Mechanistic detail of the *cis–trans* diborene isomerisation step provided in Fig. 6.

The exergonic isomerisation step leading from the *cis*-diborenes **I2_2_** and **I2_3_** to the *trans*-diborenes **2** and **I4_3_**, respectively, was further investigated to determine the rotation barrier in each case. Interestingly, DFT calculations showed two distinct mechanisms at work for the SIDep-stabilised and the cAAC-stabilised diborene, respectively (Fig. 6). For the SIDep analogue **I2_2_**, rotation about the B–B bond is facilitated by shifting the π-electron density of the B=B double bond into the π backbonding to the unsaturated carbene ligands. The resulting transition state **TS3_2_** now displays a B–B single bond, which allows facile rotation. The isomerisation process from **I2_2_** to **2** occurs with a low barrier of 9.7 kcal mol^–1^. In contrast, the lowest energy pathway for the cAAC analogue **I2_3_** proceeds *via* a 1,2-hydride shift from boron to the adjacent cAAC carbene carbon to yield the intermediate diborene **I3_3_** (Δ*G*‡3 = 8.9 kcal mol^–1^), in which the boron bearing the now protonated cAAC ligand is sp-hybridised. Rotation about this B–C_cAACH_ single bond and a second 1,2-hydride shift back to the boron centre then yield the *trans*-diborene **I4_3_** with a low barrier of 9.3 kcal mol^–1^. This pathway is assisted on the one hand by the facile 1,2-hydride shuttling chemistry displayed by cAAC hydroboron compounds^19^ and on the other hand by the very strong π acceptor properties of cAAC,^12^ which enable the stabilisation of the coordinatively saturated intermediate **I3_3_**.

**Fig. 6:**
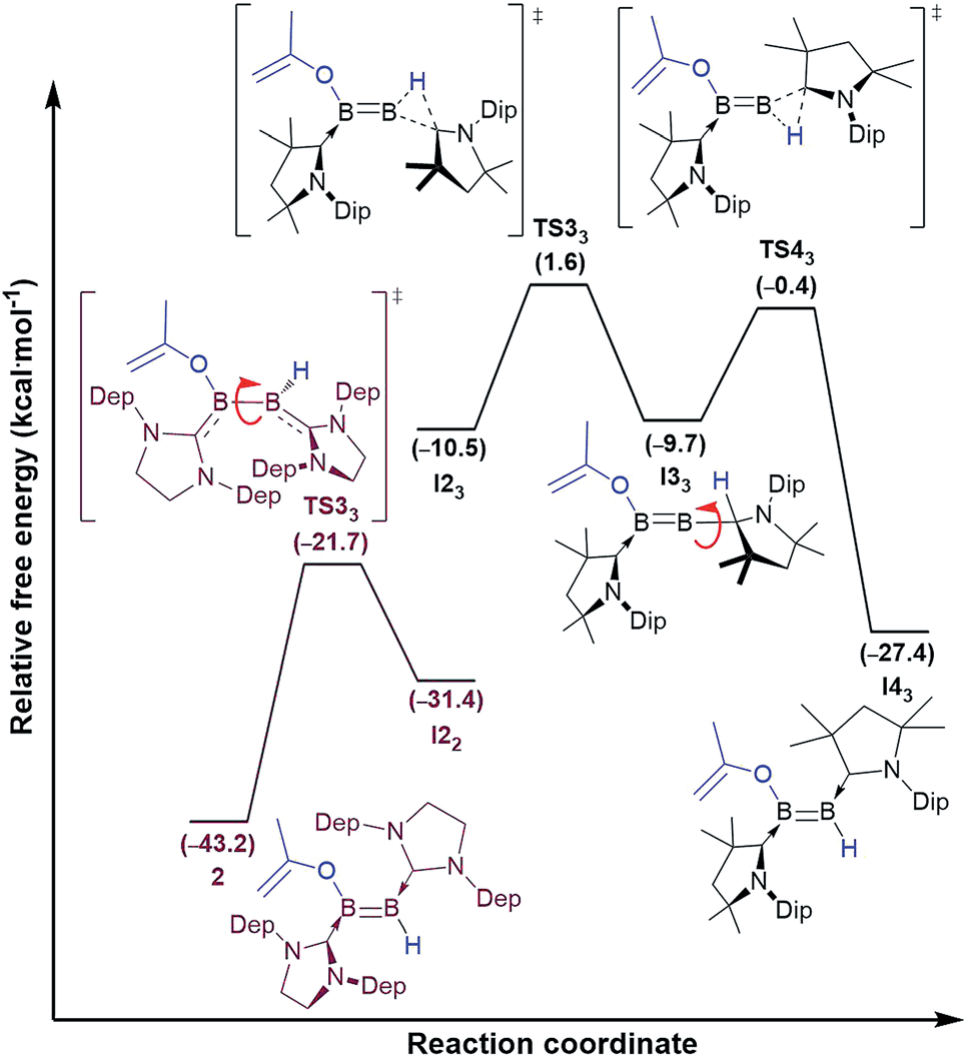
Divergent mechanisms of *cis*-to-*trans* isomerisation for diborene **I2_2_** (purple lines) and **I3_2_** (black lines) calculated at the ONIOM(M06-2X/6-311+G(d):PM6) level of theory. Gibbs free energies (kcal mol^–1^) in brackets.

For cumulene **III**, however, the reaction does not stop at *trans*-diborene **I4_3_** (Fig. 3). The latter undergoes hydride migration from B1 to B2 to form the (alkoxy)hydroboryl-(alkylidene)borane **I4_3_** (Δ*G*‡3 = 19.8 kcal mol^–1^). Coordination of the pendant terminal alkene to the two-coordinate boron yields adduct **I5_3_**, which is 6.6 kcal mol^–1^ more stable. Subsequent C–H activation of the methylidene moiety yields the bis(cAAC)-stabilised 1,2,3-oxadiborole **I6_3_** (Δ*G*‡5 = 21.2 kcal mol^–1^). This is the highest energy barrier in the entire reaction mechanism. **I6_3_** then tautomerises to compound **3a** by concomitant migration of the hydride on B1 to the adjacent cAAC carbene centre and bridging of the hydride on B2 (Δ*G*‡6 = 11.7 kcal mol^–1^). Overall the formation of **3a** from **III** and acetone is exergonic by 61.7 kcal mol^–1^, which explains why the intermediate diborene cannot be isolated.

To conclude, we have shown that three linear, isolobal, multiply bonded boron compounds, iminoborane **IV**, diboryne **II** and cumulene **III**, all activate acetone *via* a similar acetone coordination-deprotonation mechanism, regardless of their polar or nonpolar nature. For the iminoborane-based reaction, an enol addition mechanism and a mechanism proceeding *via* a [2 + 2] cycloaddition intermediate, as would normally be expected for such a polar compound, were both ruled out. For diboron compounds **II** and **III** the addition of acetone first yields a *cis*-diborene intermediate which isomerises to the thermodynamic *trans*-diborene product through a low energy barrier. Calculations showed that this isomerisation process heavily relies on the π-accepting nature of the carbene ligands, coupled, in the case of the cAAC-supported diborene, with a hydride shuttling mechanism from boron to the carbene carbon and back. These cAAC-specific properties also enable an unprecedented second C–H activation of the enolate ligand to yield a novel 1,2,3-oxadiborole heterocycle, demonstrating once again the unique reactivity of cAAC-supported low-valent boron compounds.

Overall this study should act as a reminder that the parallels all too eagerly drawn between organic compounds and their isoelectronic/isolobal inorganic p-block counterparts only rarely translate into actual organomimetic behaviour when it comes to reactivity or reaction mechanisms. Furthermore, this first example of reactivity overlap between polar and nonpolar boron-based triple bonds opens up new avenues for attempting reactions that may have been previously disregarded, such as the addition of nonpolar small molecules to iminoboranes or, alternatively, of polar molecules to diborynes.

## Conflicts of interest

There are no conflicts to declare.

## Supplementary Material

Supplementary information

Crystal structure data
