# Supplementary material for: Single and double activation of acetone by isolobal B[triple bond, length as m-dash]N and B[triple bond, length as m-dash]B triple bonds
Source: Chem Sci. 2018 Apr 30;9(24):5354–9. doi: 10.1039/c8sc01249k (PMC6009441; doi:10.1039/c8sc01249k)
Supplement: Supplementary file 1 [file SC-009-C8SC01249K-s001.pdf]

Electronic Supplementary Material (ESI) for Chemical Science.
This journal is © The Royal Society of Chemistry 2018

Supporting Information for:

Single and Double Activation of Acetone by Isolobal B≡N and B≡B Triple Bonds

Julian Böhnke,[a,b] Tobias Brückner,[a,b] Alexander Hermann,[a,b] Oscar F. González-Belman,[c] Merle Arrowsmith,[a,b] J. Oscar C. Jiménez-Halla,*[,c] Holger Braunschweig*[,a,b]

[a] *Institut für Anorganische Chemie, Julius-Maximilians-Universität Würzburg, Am Hubland, 97074 Würzburg, Germany*

[b] *Institute for Sustainable Chemistry & Catalysis with Boron, Julius-Maximilians-Universität Würzburg, Am Hubland, 97074 Würzburg, Germany*

[c] *Departamento de Química, Universidad de Guanajuato, Noria Alta S/N, 36050 Guanajuato, México*

Table of contents

Methods and materials 2

Synthetic procedures 3

NMR spectra of isolated new compounds 7

UV/Vis Spectra 22

IR spectra 23

X-ray crystallographic data 25

DFT calculations 28

Methodology 28

TDDFT results for compound **2** 31

Geometry optimisation and ^{11}B NMR shift calculations of **3a** and **3b** 32

Reaction mechanisms 34

Coordinates of reactants, transition states, intermediates and products 62

References 109

Methods and materials

All manipulations were performed either under an atmosphere of dry argon or in vacuo using standard Schlenk line or glovebox techniques. Deuterated solvents were dried over activated 4 Å molecular sieves and degassed by three freeze-pump-thaw cycles prior to use. All other solvents were distilled and degassed from appropriate drying agents. NMR spectra were acquired on a Bruker Avance 400 NMR spectrometer (^{1}H and ^{1}H{^{11}B}: 400.1 MHz, ^{11}B{^{1}H}: 128.4 MHz, ^{13}C{^{1}H} and ^{13}C{^{1}H,^{11}B}: 100.6 MHz) or a Bruker Avance 500 NMR spectrometer (^{1}H and ^{1}H{^{11}B}: 500.1 MHz, ^{11}B{^{1}H}: 160.5 MHz, ^{13}C{^{1}H} and ^{13}C{^{1}H,^{11}B}: 125.8 MHz). Chemical shifts (δ) are given in ppm and internally referenced to the carbon nuclei (^{13}C{^{1}H}) or residual protons (^{1}H) of the solvent. ^{11}B{^{1}H} NMR spectra were referenced to [$BF_3 \cdot OEt_2$] as an external standard. UV/Vis spectra were acquired on a JASCO-V660 UV/Vis spectrometer under inert conditions inside a glovebox. Microanalyses (C, H, N) were performed on an Elementar vario MICRO cube elemental analyzer. Solid-state IR spectra were acquired on a Bruker Alpha using a setup with a Bruker diamond crystal single reflection ATR system. High-resolution mass spectrometry data was obtained from a Thermo Scientific Exactive Plus spectrometer in ASAP or LIFDI mode. Diborenes **2** and **3** were too sensitive to obtain meaningful elemental analysis data, which is why they were only characetrised by HRMS.

Solvents and reagents were were purchased from Sigma Aldrich. Deuterated solvents were degassed with three freeze-pump-thaw cycles and stored over molecular sieves in Young ampoules or in a glovebox. Acetone was dried by distillation over anhydrous magnesium sulfate and stored over molecular sieves. Ar*NH_2 (Ar* = C_6H_2-2,6-($CHPh_2$)$_2$-4-^{t}Bu),[1] LiTMP (TMP = 2,2,6,6-tetramethylpiperidine),[2] diboryne **II**[3] and cumulene **III**[4] were synthesised using literature procedures.

Synthetic procedures

Ar*N(H)BCl$_2$

A stirred solution of 3.15 g (6.5 mmol) Ar*NH$_2$ in 100 mL toluene was cooled to –78 °C and *n*BuLi (4.08 mL of a 1.6 M solution in hexane, 6.5 mmol) was added dropwise. The solution was warmed to rt and stirred for 4 h. The resulting orange suspension was again cooled to –78 °C and BCl$_3$ in hexanes was added dropwise (7.18 mL of a 1.0 M solution in hexane, 7.2 mmol). After stirring overnight at rt, the reaction mixture was filtered and all volatiles were removed *in vacuo*. The residue was washed with hexanes to yield crude Ar*NHBCl$_2$ as a pale yellow solid (3.17 g). Despite multiple recrystallizations samples of Ar*NHBCl$_2$ always remained contaminated with small amounts of boron-containing impurities. Furthermore the compound decomposed upon attempted inert atmosphere column chromatography. As a result crude Ar*NHBCl$_2$ was employed in the next reaction step (*vide infra*). ^{1}H NMR (500 MHz, C$_6$D$_6$): δ = 7.21 (br, 4H, Ar-*H*), 7.14-7.05 (m, 14H, Ar-*H*), 7.11 (s, 4H, Ar-*H*), 5.72 (s, 2H, C*H*Ph$_2$), 5.01 (s, 1H, N*H*), 1.00 (s, 9H, C(C*H$_3$*)$_3$) ppm. ^{13}C{^{1}H} NMR (126 MHz, C$_6$D$_6$): δ = 149.8, 143.2, 141.9, 134.7, 130.2, 129.7, 128.8, 126.9, 126.2 (Ar-*C*), 53.4 (*C*HPh$_2$), 34.7 (*C*(CH$_3$)$_3$), 31.1 (*C*H$_3$) ppm. ^{11}B NMR (161 MHz, C$_6$D$_6$): δ = 33 ppm. Elemental analysis: calcd for C$_{36}$H$_{34}$BCl$_2$N: C 76.89, H 6.09, N 2.49; found: C 77.22, H 6.23, N 2.46%. *Note: this analysis was performed on handpicked single crystals to ensure compound purity.*

Ar*N(H)BCl(TMP)

Ar*N(H)BCl$_2$ (1.33 g, 2.37 mmol) and LiTMP (350 mg, 2.37 mmol) were combined in a flask and hexane (40 mL) and toluene (20 mL) were added. After stirring the mixture for 2 h, the suspension was filtered and all volatiles were removed *in vacuo*. Hexane (10 mL) was added to the residue and the mixture was cooled to –78 °C. After centrifugation, Ar*N(H)BCl(TMP) was isolated as a colourless solid (1.02 g, 1.53 mmol, 65%). ^{1}H NMR (500 MHz, 296 K, C$_6$D$_6$): δ = 7.44 (m, 4H, Ar-*H*), 7.22-7.18 (m, 4H, Ar-*H*), 7.15-7.14 (m, 5H, Ar-*H*), 7.12-7.05 (m, 7H, Ar-*H*), 7.03-6.98 (m, 2H, Ar-*H*), 6.24 (s, 2H, C*H*Ph$_2$), 4.14 (s, 1H, N*H*), 1.48-1.45 (m, 2H, C*H*$_2$), 1.39-1.36 (m, 4H, C*H*$_2$), 1.21 (s, 12H, C*H*$_3$), 1.02 (s, 9H, C(C*H$_3$*)$_3$) ppm. ^{13}C{^{1}H} NMR (126 MHz, 296 K, C$_6$D$_6$): δ = 148.5, 145.4, 144.0, 141.4, 137.9 (Ar-*C$_q$*), 130.6, 129.7, 128.6, 126.5 (Ar-*C*), 53.2 (*C*HPh$_2$), 53.1 (*C*(CH$_3$)$_2$), 36.8 (*C*H$_2$), 34.6 (*C*(CH$_3$)$_3$), 32.1 (C(*C*H$_3$)$_2$), 31.2 (*C*H$_3$), 15.6 (CH$_2$-*C*H$_2$-CH$_2$) ppm. ^{11}B{^{1}H} NMR (128 MHz, 296 K, C$_6$D$_6$): δ = 30.4 ppm (br s, FWMH = 583.8 Hz). Elemental analysis: calcd for C$_{45}$H$_{52}$BClN$_2$: C 80.01, H 7.86, N 4.20; found: C 79.86, H 7.84, N 3.92%.

Ar*N≡B(TMP), IV

Ar*N(H)BCl(TMP) (0.82 g, 1.23 mmol) and LiTMP (0.24 g, 1.60 mmol) were combined in a flask and hexane (25 mL) was added. The suspension was heated to 80 °C in a closed flask for 2 h, in which time the suspension turned orange. The mixture was filtered, the solution was reduced in vacuum and cooled to –30 °C to give colourless crystals of **IV** (0.44 g, 699 μmol, 57%). ^{1}H NMR (500 MHz, 296 K, C_6D_6): δ = 7.26-7.25 (m, 8 H, Ar-*H*), 7.14 (m, 2 H, Ar-*H*), 7.13 (m, 4 H, Ar-*H*), 7.12-7.11 (m, 4 H, Ar-*H*), 7.05-7.02 (m, 4 H, Ar-*H*), 6.33 (s, 2 H, C*H*Ph_2), 1.33-1.28 (m, 2 H, CH_2), 1.13 (s, 9 H, C(CH_3)$_3$), 1.07-1.05 (m, 4 H, CH_2), 0.93 (s, 12 H, CH_3) ppm. ^{13}C{^{1}H} NMR (126 MHz, 296 K, C_6D_6): δ = 145.5, 143.2, 138.7, 137.3 (Ar-C_q), 130.3, 128.4, 126.2, 125.7 (Ar-*C*), 53.8 (*C*HPh_2), 51.7 (*C*(CH_3)$_2$), 38.2 (*C*H_2), 34.6 (C_q, *C*(CH_3)$_3$), 31.7 (C(*C*H_3)$_2$), 31.6 (*C*H_3), 17.7 (CH_2-*C*H_2-CH_2) ppm. ^{11}B{^{1}H} NMR (161 MHz, 296 K, C_6D_6): δ = 12 ppm (br s, FWMH = 393.0 Hz). IR (toluene): ν = 2036 (B≡N), 1998 (B≡N) cm^{-1}. Elemental analysis: calcd for $C_{45}H_{51}BN_2$: C 85.69, H 8.15, N 4.44; found: C 85.25, H 8.16, N 4.37%.

(Ar*NH)B(TMP)(OC=CH$_2$Me), 1

A suspension of 67.0 mg (106 μmol) **I** in 1 mL hexane was treated with 0.04 mL (544 μmol, 5.1 equiv.) of acetone. The mixture was heated overnight at 70 °C and filtered. Colourless crystals were obtained by slow evaporation of a hexane solution at –30 °C. Yield: 38.7 mg (56.2 μmol, 53%). *Note: the reaction also proceeded at room temperature, but never to completion.* ^{1}H NMR (400 MHz, C_6D_6): δ = 7.26 (br, 7H, Ar-*H*), 7.18 (s, 3H, Ar-*H*), 7.15 (s, 3H, Ar-*H*), 7.11–7.08 (m, 2H, Ar-*H*), 7.07 (s, 2H, Ar-*H*), 7.06–7.00 (m, 5H, Ar-*H*), 6.22 (s, 2H, C*H*(Ph)$_2$), 4.36 (s, 1H, (CH_2)CO), 4.11 (s, 1H, (CH_2)CO), 3.49 (s, 1H, N-*H*), 1.52 (s, 3H, (CH_3)CO), 1.49–1.46 (m, 2H, CH_2C$H_2$$CH_2$), 1.47–1.43 (m, 4H, C$H_2$$CH_2CH_2$), 1.19 (s, 12H, C(C$H_3$)$_2$), 1.05 (s, 9H, C(C$H_3$)$_3$). ^{13}C{^{1}H} NMR (126 MHz, C_6D_6): δ = 157.3 (*C*O), 147.1 (C_{ar}), 145.1 (C_{ar}), 143.6 (C_{ar}), 140.7 (C_{ar}), 138.7 (C_{ar}), 130.2 (CH_{ar}), 130.0 (CH_{ar}), 128.7 (CH_{ar}), 126.8 (CH_{ar}), 126.5 (CH_{ar}), 126.0 (CH_{ar}), 90.3 (*C*H_2)CO), 53.2 (*C*H(Ph)$_2$), 52.7 (*C*(CH_3)$_2$), 38.8 (*C*$H_2$$CH_2$*C*$H_2$), 34.6 (*C*($CH_3$)$_3$), 32.5 (C(*C*$H_3$)$_2$), 31.3 (C(*C*$H_3$)$_3$) 22.1 ((*C*$H_3$)CO), 16.3 ($CH_2$*C*$H_2$$CH_2$). ^{11}B NMR (128 MHz, C_6D_6): δ = 24.8 (br s, FWMH = 567.9 Hz). IR (solid state): ν = 3414 (N-H), 1648 (C=C) cm^{-1}. Elemental analysis: calcd for $C_{48}H_{57}BN_2O$: C 83.70, H 8.34, N 4.07; found: C 82.93, H 8.35, N 4.17%. LIFDI-MS [$C_{48}H_{57}BN_2O$]: m/z (calculated) = 688.4558; m/z (found): 688.4553.

(SIDep)B(OC=CH_2Me)=BH(SIDep), 2

A solution of 40 mg (58.0 µmol) of **II** in 3 mL benzene was treated with 300 µL acetone (6.54 mmol, 112 equiv.). The reaction mixture instantly turned from red to green. After removal of the solvent under reduced pressure, the residue was extracted with pentane. Recrystallisation at –70 °C in pentane gave **2** as a green solid (20.4 mg, 27.2 µmol, 47% yield). *Note: the addition of 1 – 10 equiv. acetone resulted in extremely slow reaction times, which is why such a large excess had to be used, as heating led to decomposition. In pure acetone, however, the reaction did not proceed as cleanly as in the 10:1 benzene:acetone mixture.* ^{1}H NMR (400 MHz, C_6D_6): δ = 7.09–7.08 (m, 2H, Ar-*H*), 7.07–7.06 (m, 6H, Ar-*H*), 6.98–6.96 (m, 4H, Ar-*H*), 3.93 (s, 1H, C=C*H*), 3.47 (s, 1H, C=C*H*), 3.20 (d, ^{2}J = 13.3 Hz, 8H, NCH_2) 2.82–2.53 (m, 17H, Et-CH_2, B*H*), 1.26 (q, ^{2}J = 8.0 Hz, 24H, CH_3), 0.82 (s, 3H, CH_3) ppm. ^{13}C{^{1}H} (126 MHz, C_6D_6): δ = 161.2 ($C_{qO–C}$), 142.0 (C_q), 141.4 (C_q), 141.1 (C_q), 140.2 (C_q), 127.0 (C_{ar}), 126.9 (C_{ar}), 126.1 (C_{ar}), 125.9 (C_{ar}), 81.8 (C=CH_2), 51.8 (NCH_2), 51.0 (NCH_2), 24.5 (CH_2), 24.3 (CH_3), 14.3 (CH_3). ^{11}B NMR (128 MHz, C_6D_6): δ = 38.1 (*B*H), 19.3 (*B*O) ppm. IR (solid state): ν = 1627 (C=C), 1221 (C=C-O-C), 1187 (C-O) cm^{-1}. UV-vis (pentane): λ_{max} = 605 nm. LIFDI-MS [$C_{49}H_{66}B_2N_4O$]: m/z (calculated) = 748.5405; m/z (found) = 748.5417.

(cAACH)B-*µ*-H-*µ*-(OC=CH_2CH_2)-B(cAAC), 3

29.4 mg (506 *µ*mol) of purified acetone were added to a stirring solution of 250 mg **III** (422 *µ*mol) in 10 mL pentane. The reaction mixture instantly turned from purple to yellow. After removal of all volatiles *in vacuo* the dark yellow precipitate was washed with 3 x 1 mL cold pentane. Yellow crystals were obtained by slow evaporation of a hexane solution at –30 °C. Yield: 112 mg (172 *µ*mol, 41%). The NMR spectra showed the presence of two distinct tautomers **3a** and **3b** in a 92:8 ratio. ^{1}H NMR (500 MHz, C_6D_6), major tautomer **3a**: δ = 7.23–7.17 (m, 4H, Ar-*H*), 7.05–7.03 (m, 2H, Ar-*H*), 4.34 (sept, 1H, ^{3}J = 6.7 Hz, ^{i}Pr-C*H*), 4.02 (s, 1H, BC_{cAAC}-*H*), 3.78 (sept, 1H, ^{3}J = 6.7 Hz, CH_{iPr}), 3.50 (s, 1H, C=C*H*), 2.70 (sept, 1H, ^{3}J = 6.6 Hz, ^{i}Pr-C*H*), 2.62 (sept, 1H, ^{3}J = 6.5 Hz, ^{i}Pr-C*H*), 2.31, 2.19 (two d, 1H each, ^{2}J = 12.2 Hz, CH_2), 2.04 (s, 3H, (CH_3)CO), 1.90, 1.71, 1.66 (three s, 3H each, C(CH_3)$_2$), 1.63 (d, 3H, ^{3}J = 6.6 Hz, ^{i}Pr-CH_3), 1.56 (s, 3H, C(CH_3)$_2$), 1.50, 1.45 (two d, 1H each, ^{2}J = 12.9 Hz, CH_2), 1.41 (d, 3H, ^{3}J = 6.7 Hz, ^{i}Pr-CH_3), 1.31 (s, 1H, B*H*), 1.28, 1.25 (two d, 3H each, ^{3}J = 6.7 Hz, ^{i}Pr-CH_3), 1.23 (s, 3H, C(CH_3)$_2$), 1.12, 1.10, 1.09 (three d, 3H each, ^{3}J = 6.6 Hz, ^{i}Pr-CH_3), 1.06 (s, 3H, C(CH_3)$_2$), 1.02 (d, 3H, ^{3}J = 6.6 Hz, ^{i}Pr-CH_3), 0.95 (s, 3H, C(CH_3)$_2$), 0.88 (d, 3H, ^{3}J = 6.6 Hz, ^{i}Pr-CH_3), 0.77 (s, 3H, C(CH_3)$_2$); minor tautomer **3b**: δ = 7.03-7.23 (m, 6H, Ar-*H*), 4.70 (sept, 1H, ^{3}J = 6.7 Hz, ^{i}Pr-C*H*), 4.24 (s, 1H, BC_{cAAC}-*H*), 3.78 (s, 1H, C=C*H*), 3.69 (sept,

1H, ^3J = 6.7 Hz, ^{i}Pr-C*H*), 2.70 (^{i}Pr-C*H* detected by COSY), 2.53 (sept, 1H, ^3J = 6.6 Hz, ^{i}Pr-C*H*), 2.18 (CH_2 detected by COSY), 2.04 (s, 3H, OC(CH_3)), 1.96 (d, 1H, ^2J = 12.2 Hz, CH_2), 1.76 (s, 3H, C(CH_3)$_2$), 1.57 (s, 3H, C(CH_3)$_2$), 1.54 (d, 3H, ^3J = 7.0 Hz, ^{i}Pr-CH_3), 1.51 (s, 3H, C(CH_3)$_2$), 1.39, 1.38, 1.37 (three ^{i}Pr-CH_3, detected by COSY), 1.35 (s, 3H, C(CH_3)$_2$), 1.32 (d, 3H, ^3J = 7.0 Hz, ^{i}Pr-CH_3), 1.21, 1.18 (two s, 3H each, C(CH_3)$_2$), 1.08 (^{i}Pr-CH_3, detected by COSY), 0.98 (d, 3H, ^3J = 7.0 Hz, ^{i}Pr-CH_3), 0.90, 0.86 (two s, 3H each, C(CH_3)$_2$). *Note: the ^{1}H NMR resonances of **3b** detected by COSY overlapped with those of **3a**.* ^{11}B NMR (160.5 MHz, C_6D_6), major tautomer **3a**: δ = 42.8 (broad, FWMH ≈ 660 Hz), –1.9 (broad s, FWMH ≈ 190 Hz); minor tautomer **3b**: δ = 63.0 (broad, FWMH ≈ 730 Hz), –15.0 (broad d, ^1J = 50.8 Hz, FWMH ≈ 120 Hz). ^{13}C{^{1}H} NMR (C_6D_6, 125.8 MHz), major tautomer: 215.1 ($C_{carbene}$), 157.0 (*C*O), 154.4 (C_{ar}), 151.0 (C_{ar}), 147.2 (C_{ar}), 147.0 (C_{ar}), 144.0 (C_{ar}), 135.3 (C_{ar}), 129.4 (C_{ar}), 126.3 (C_{ar}), 125.5 (C_{ar}), 124.6 (C_{ar}), 124.44 (C_{ar}), 124.43 (C_{ar}), 108.9 (CH_{vinyl}), 72.5 (N*C*(CH$_3$)$_2$), 70.4 ($C_{CAAC\text{-}H}$), 63.3 (N*C*(CH$_3$)$_2$), 60.2 (*C*H$_2$), 53.6 (*C*H$_2$), 49.5 (*C*(CH$_3$)$_2$), 42.1 (*C*(CH$_3$)$_2$), 34.6 (C(*C*H$_3$)$_2$), 33.2 (C(*C*H$_3$)$_2$), 32.6 (C(*C*H$_3$)$_2$), 32.1 (C(*C*H$_3$)$_2$), 32.01 (C(*C*H$_3$)$_2$), 29.7 (*C*H(CH$_3$)$_2$), 28.9 (*C*H(CH$_3$)$_2$), 28.84 (C(*C*H$_3$)$_2$), 28.78 (C(*C*H$_3$)$_2$), 28.2 (C(*C*H$_3$)$_2$), 28.0 (*C*H(CH$_3$)$_2$), 27.5 (*C*H(CH$_3$)$_2$), 26.6 (CH(*C*H$_3$)$_2$), 25.89 (CH(*C*H$_3$)$_2$), 25.87 (CH(*C*H$_3$)$_2$), 25.6 (CH(*C*H$_3$)$_2$), 25.2 (CH(*C*H$_3$)$_2$), 24.8 (CH(*C*H$_3$)$_2$), 24.7 (CH(*C*H$_3$)$_2$), 24.0 (CH(*C*H$_3$)$_2$), 19.0 (*C*H$_3$)CO). IR (solid state): ν = 1913 (B-H), 1609 (C=C) cm^{-1}. UV-vis (pentane): λ_{max} = 397 nm. ASAP-MS [$C_{43}H_{68}B_2N_2O$ + H]$^+$: m/z (calculated) = 651.5591; m/z (found) = 651.5591.

NMR spectra of isolated new compounds

Figure S1. ^{1}H NMR spectrum of **Ar*N(H)BCl(TMP)** in C_6D_6. The triplet at 0.89 ppm and the corresponding multiplet at 1.24 ppm are from residual hexane (Hex), the singlet at 0.31 ppm to grease.

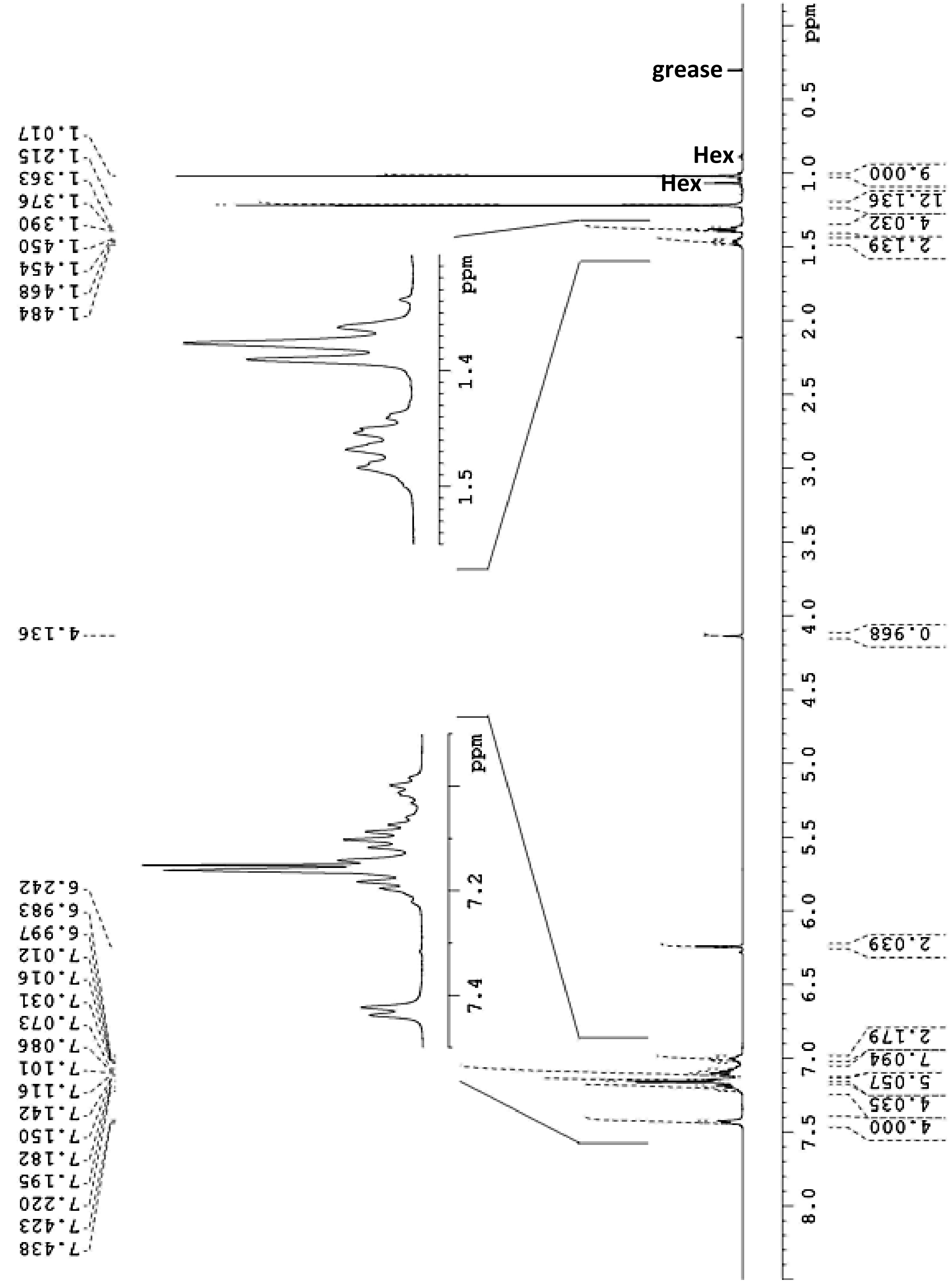

Figure S2. ^{11}B NMR spectrum of **Ar*N(H)BCl(TMP)** in C_6D_6.

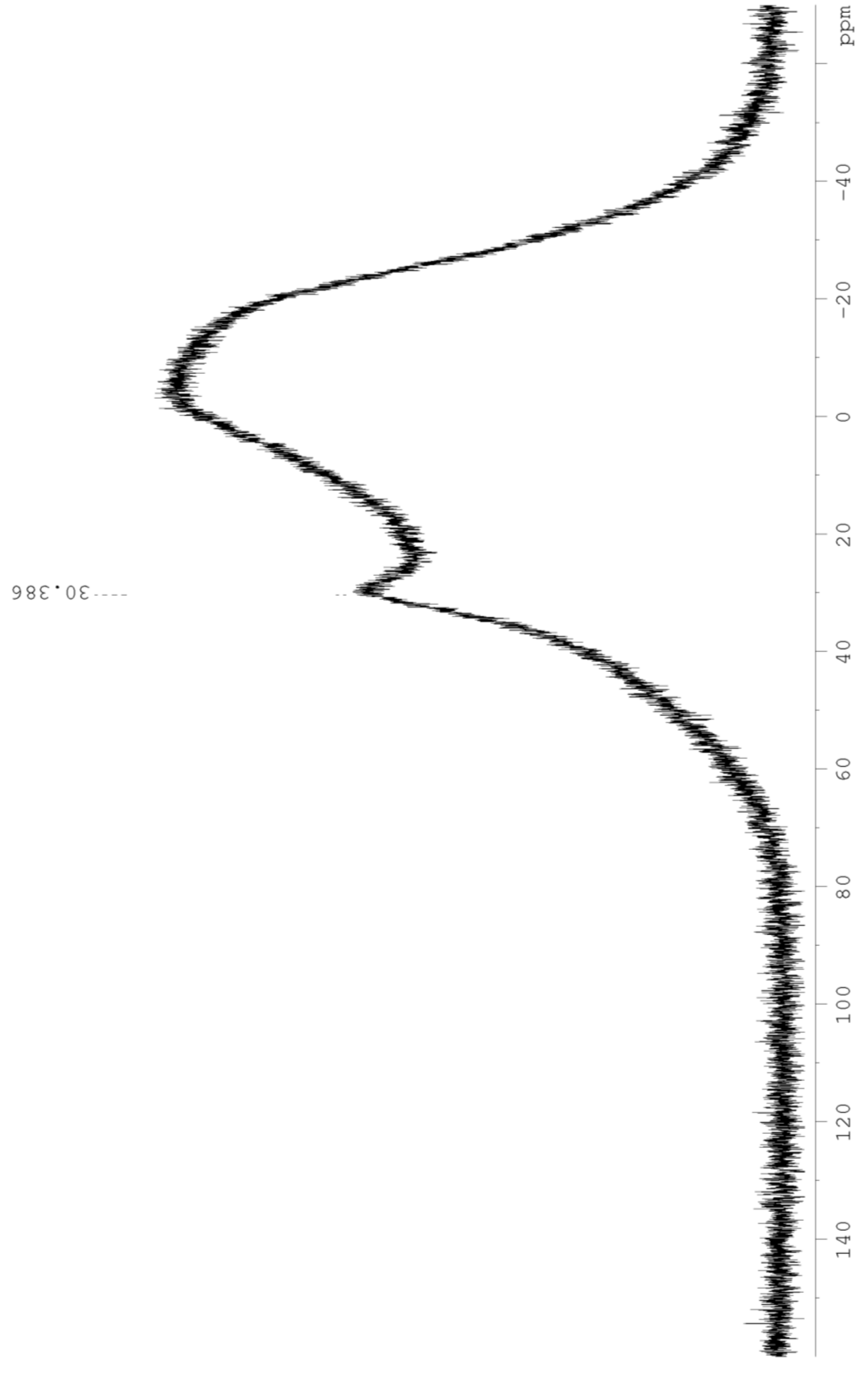

Figure S3. $^{13}C\{^{1}H\}$ NMR spectrum of of **Ar*N(H)BCl(TMP)** in C_6D_6. The resonances at 14.3, 23.0 and 32.0 ppm correspond to residual hexane.

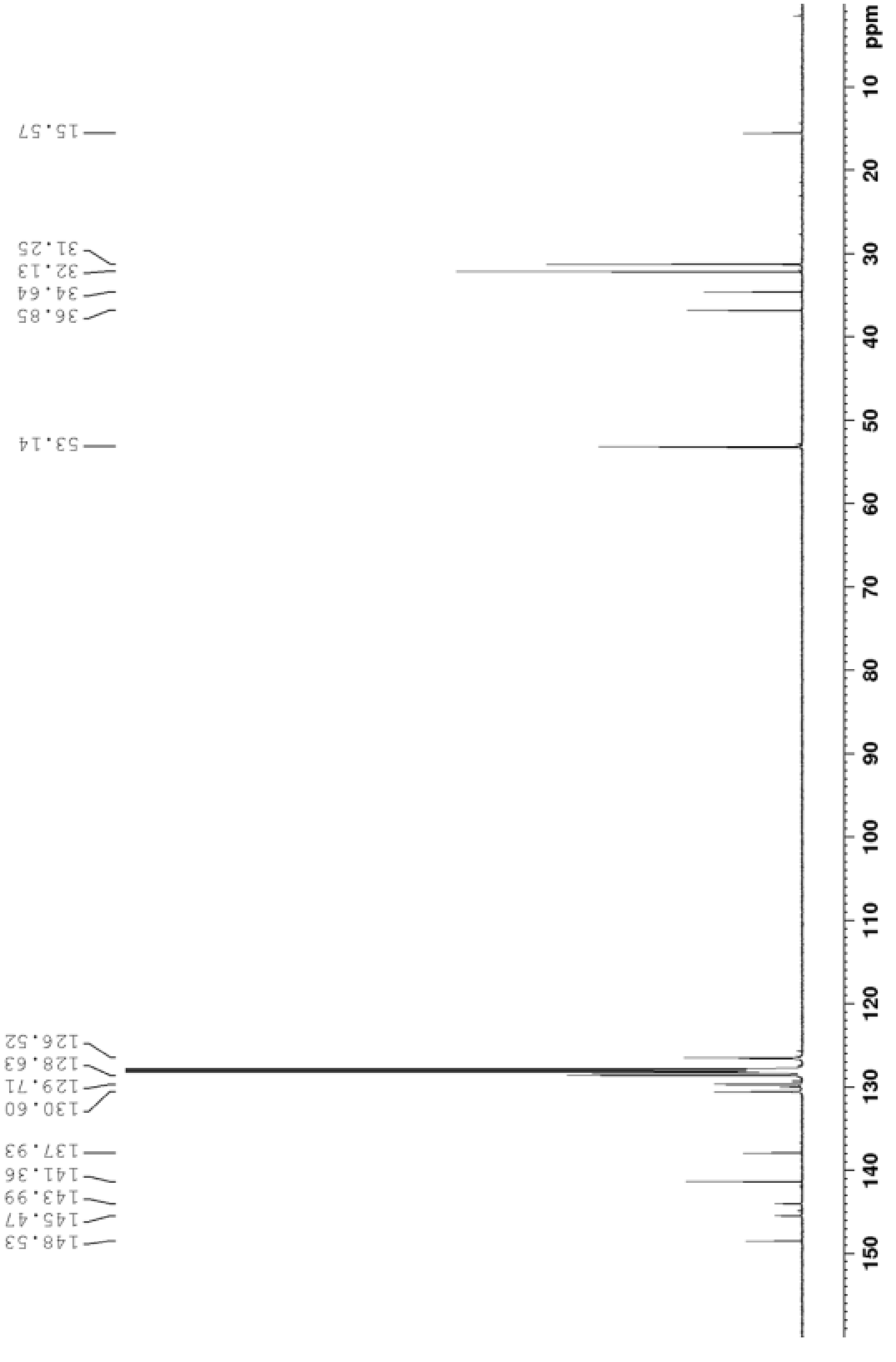

Figure S4. ^{1}H NMR spectrum of **IV** in C_6D_6. The triplet at 0.89 ppm and the corresponding multiplet at 1.24 ppm are from residual hexane.

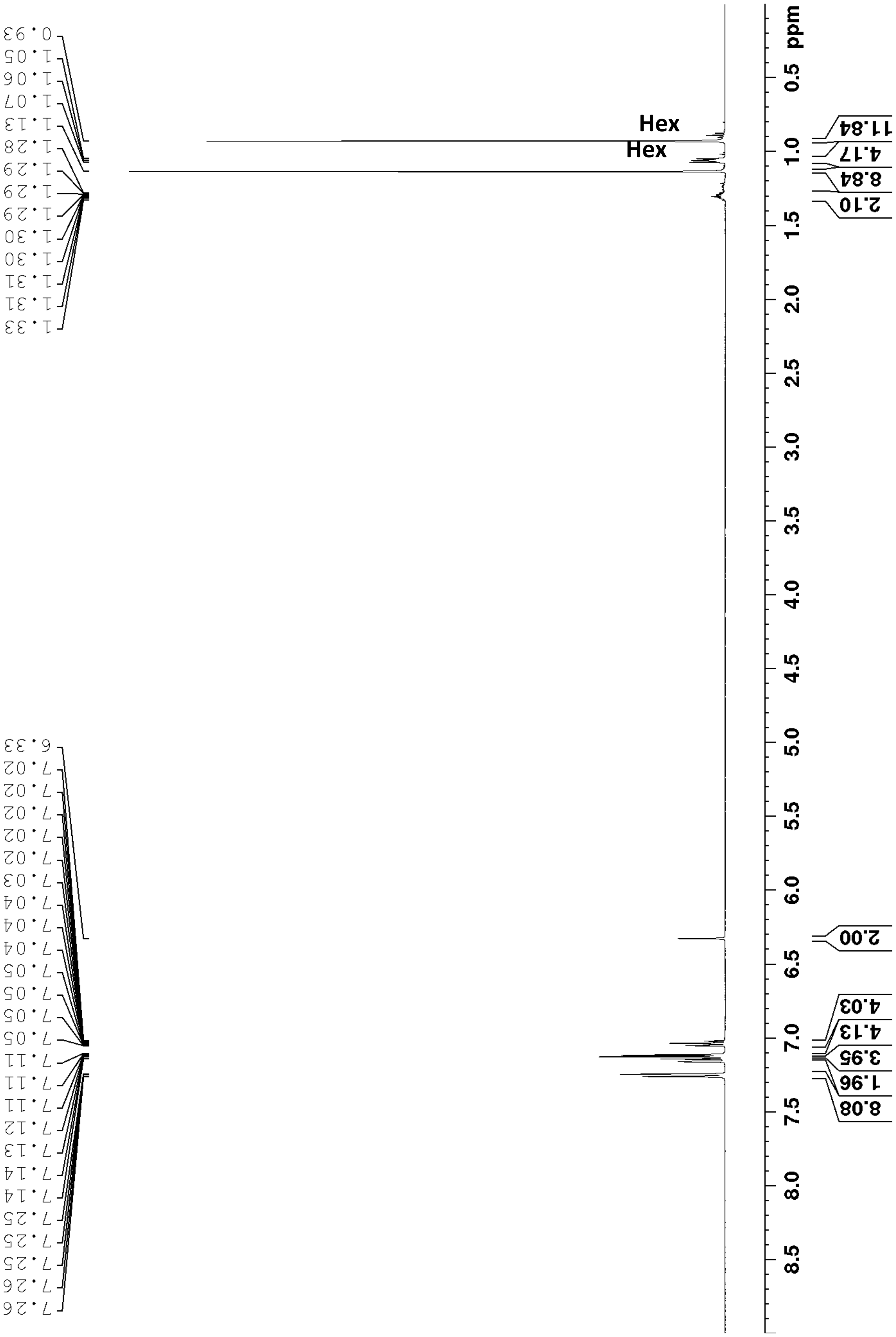

Figure S5. ^{11}B NMR spectrum of **IV** in C_6D_6.

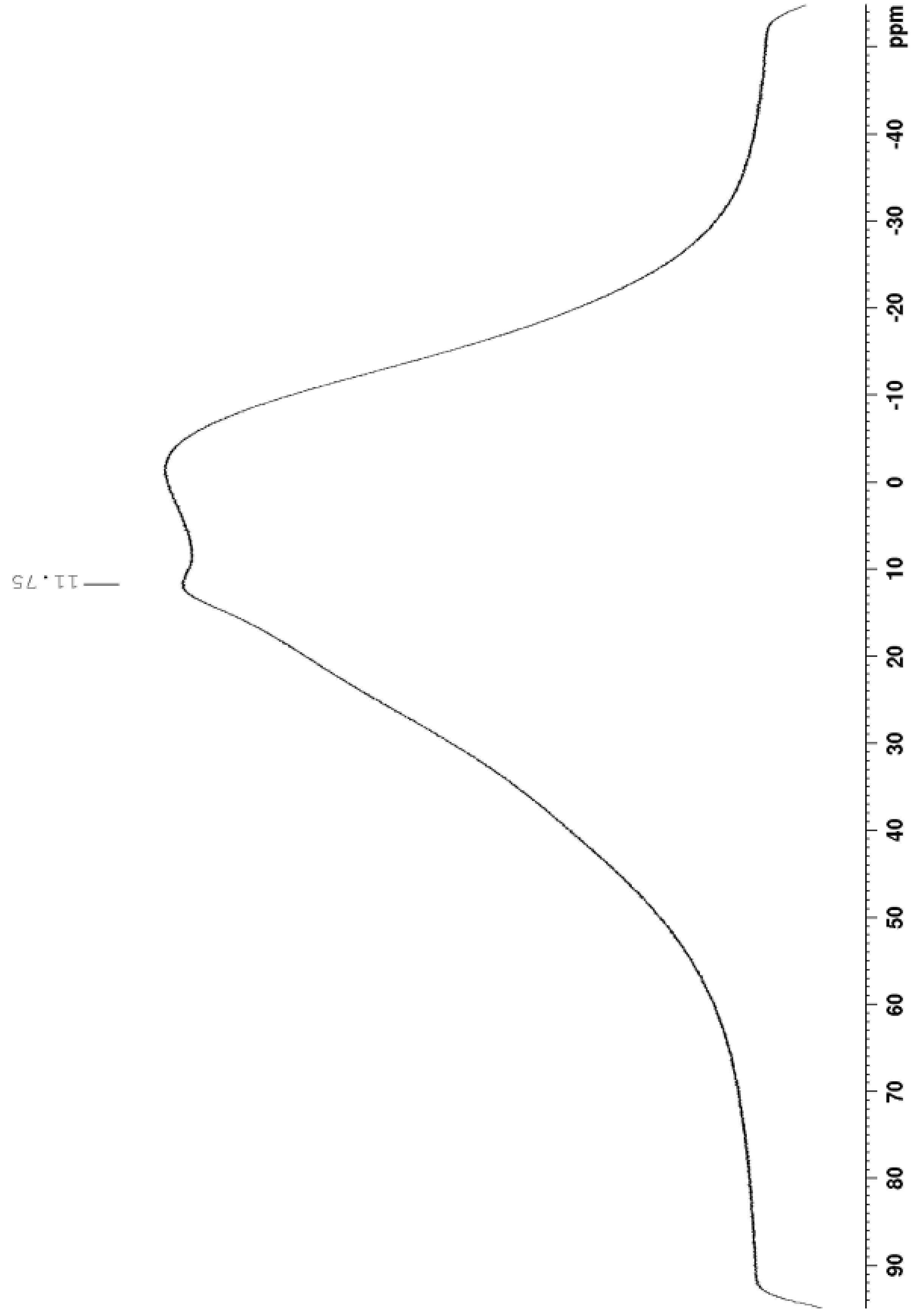

Figure S6. $^{13}C\{^1H\}$ NMR spectrum of **IV** in C_6D_6. The resonances at 14.2, 23.3 and 32.3 ppm correspond to residual hexane.

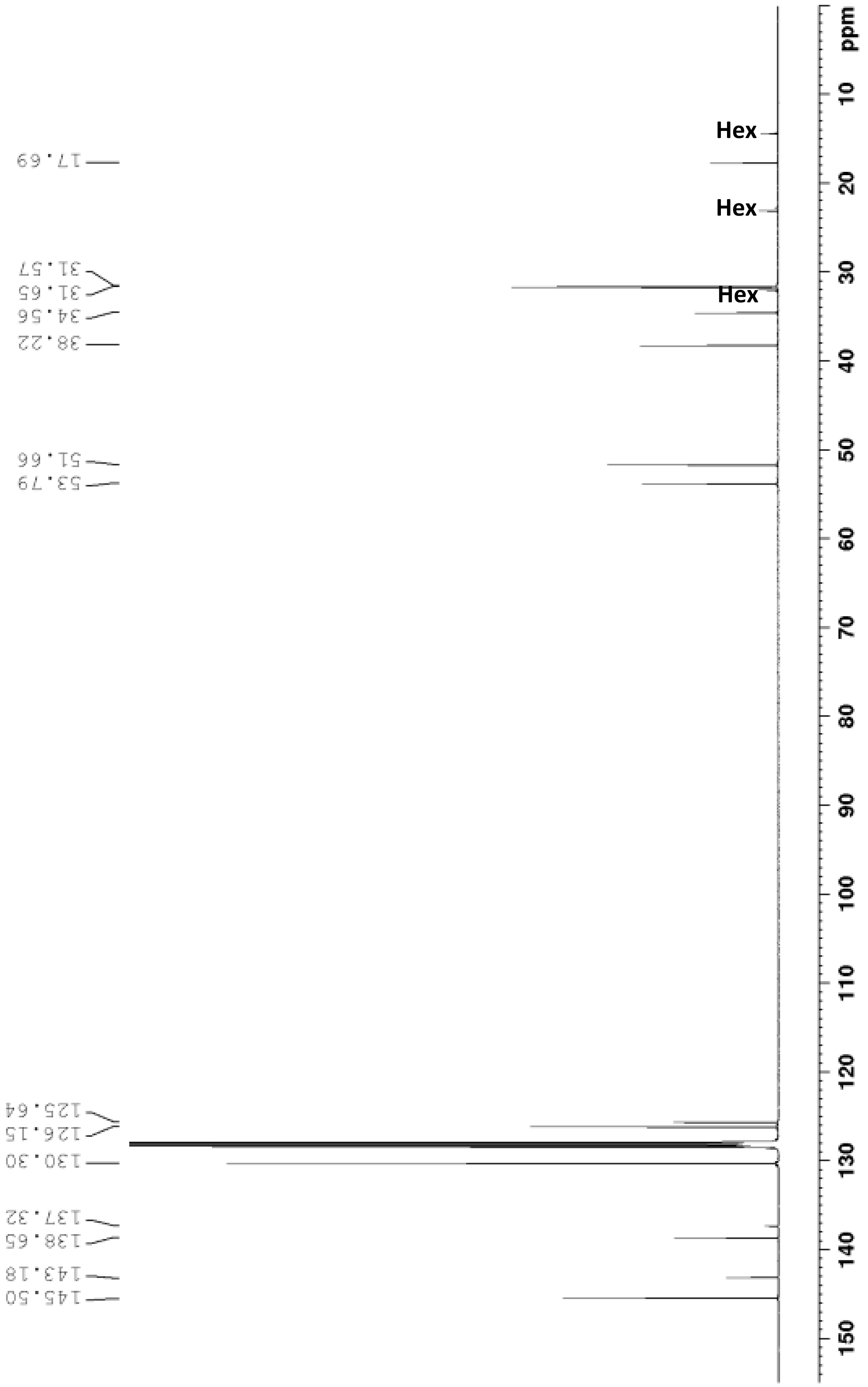

Figure S7. ^{1}H NMR spectrum of **1** in C_6D_6. The triplet at 0.89 ppm and the corresponding multiplet at 1.24 ppm are from residual hexane.

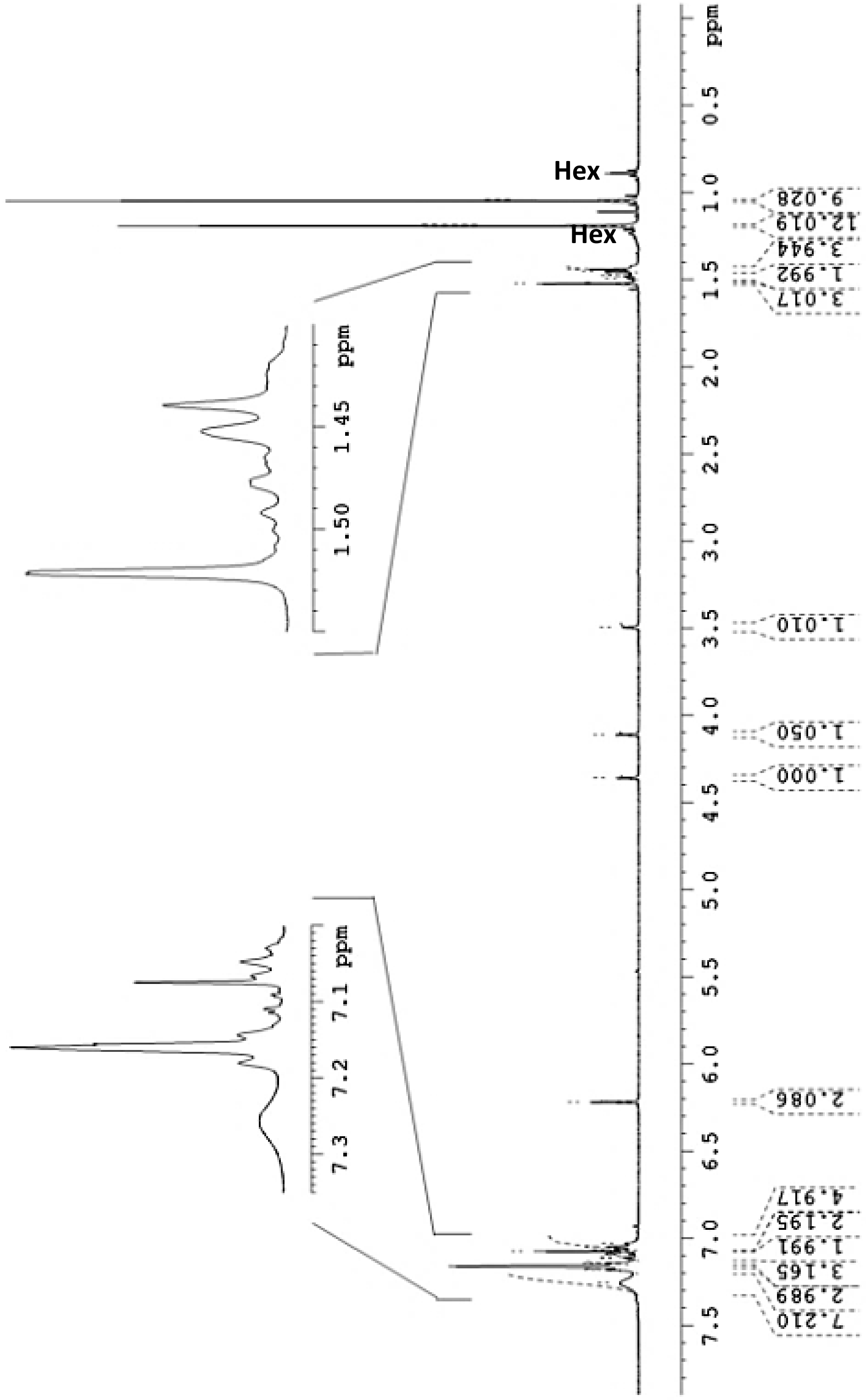

Figure S8. ^{11}B NMR spectrum of **1** in C_6D_6.

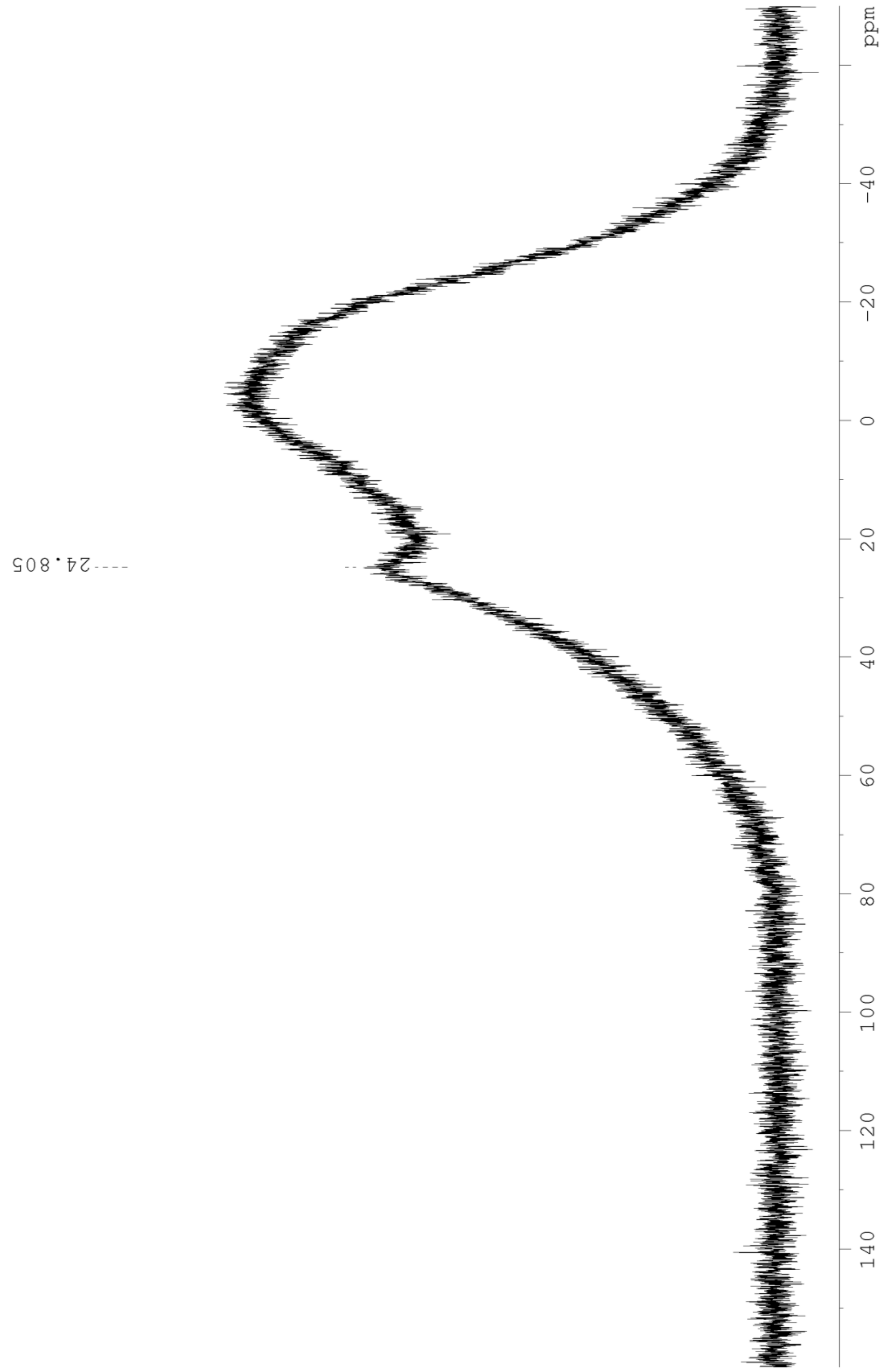

Figure S9. $^{13}C\{^{1}H\}$ NMR spectrum of **1** in C_6D_6. The resonances at 14.2, 23.3 and 32.3 ppm correspond to residual hexane.

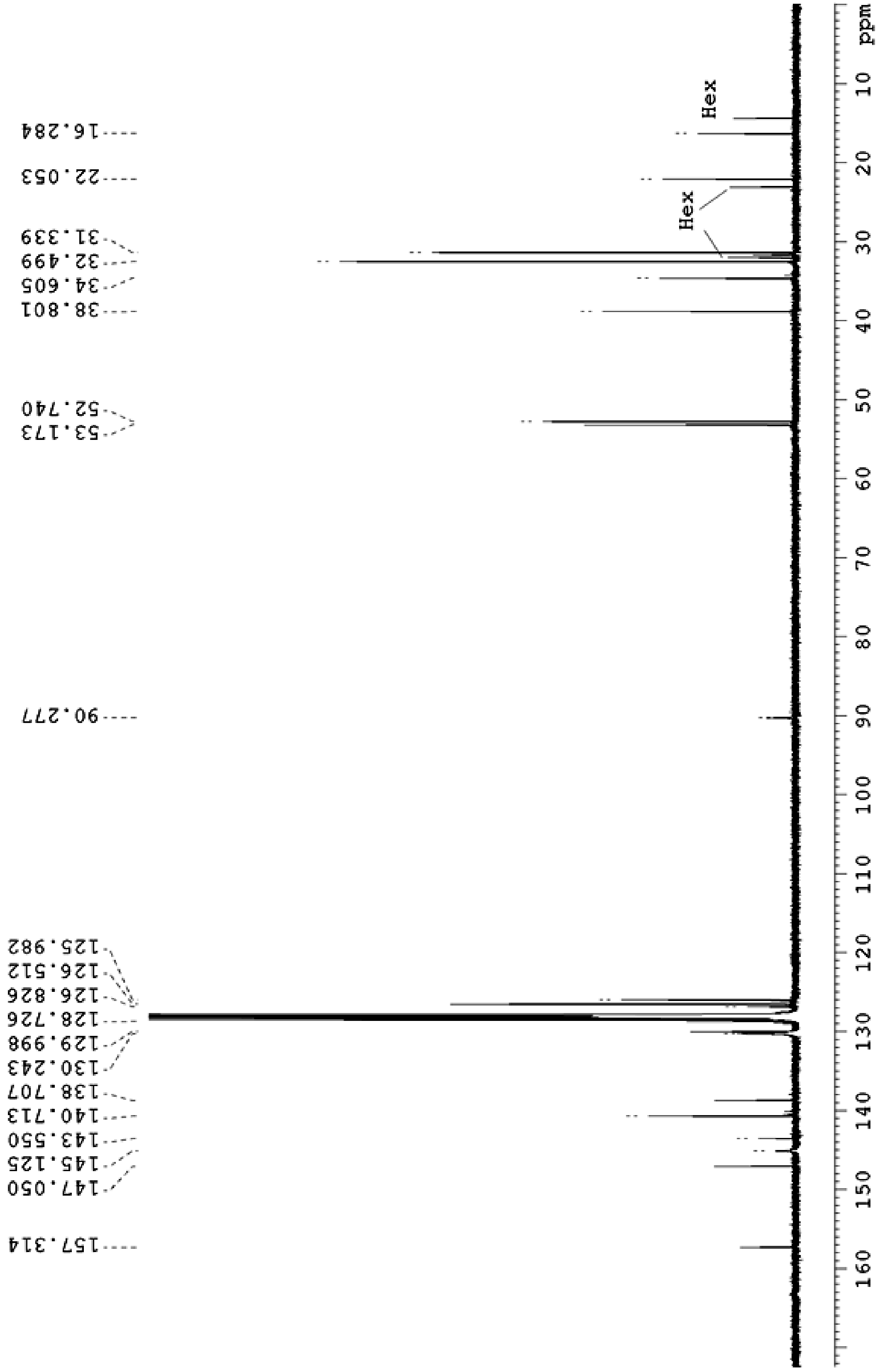

Figure S10. ^{1}H NMR spectrum of **2** in C_6D_6.

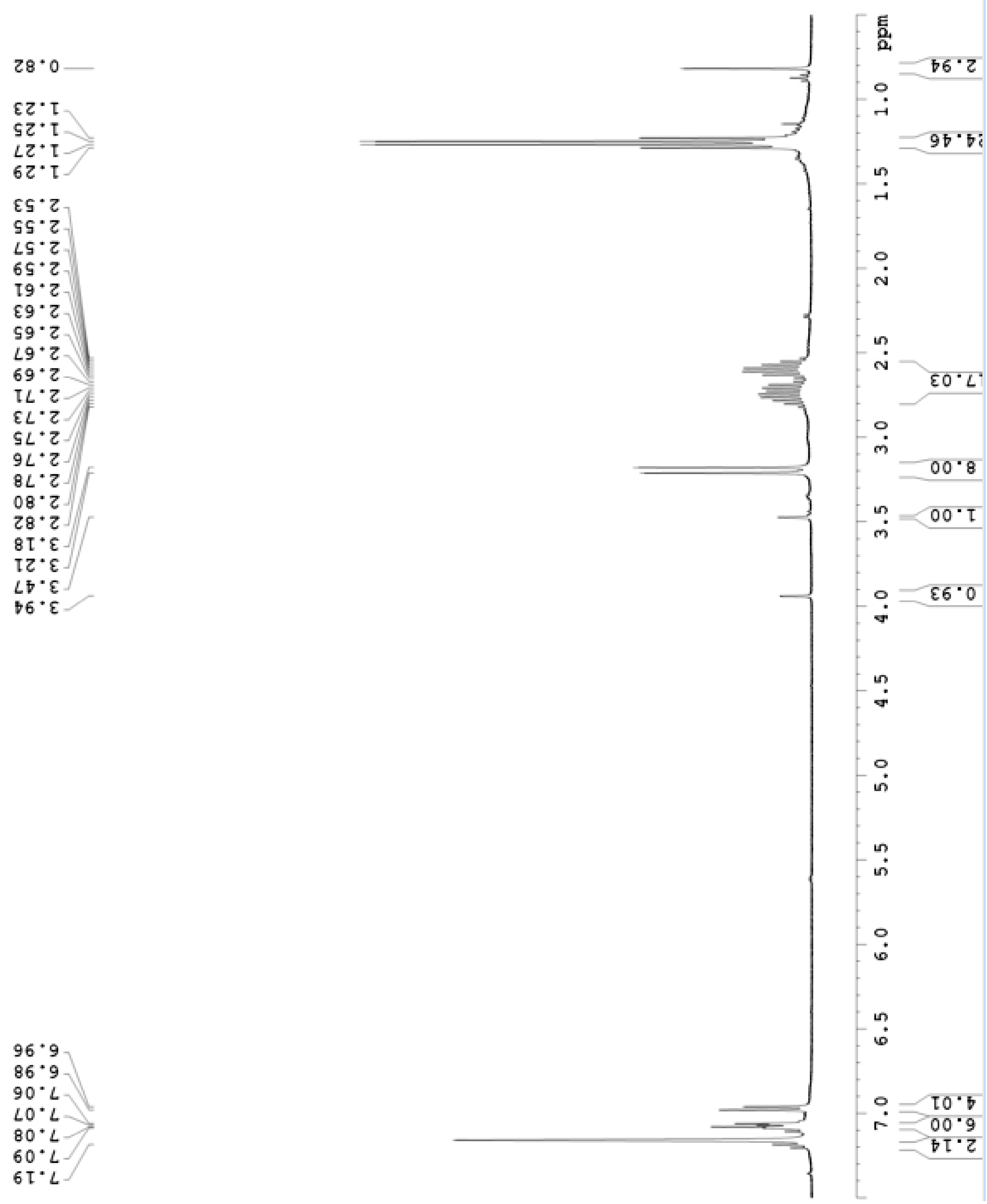

Figure S11. ^{11}B NMR spectrum of **2** in C_6D_6.

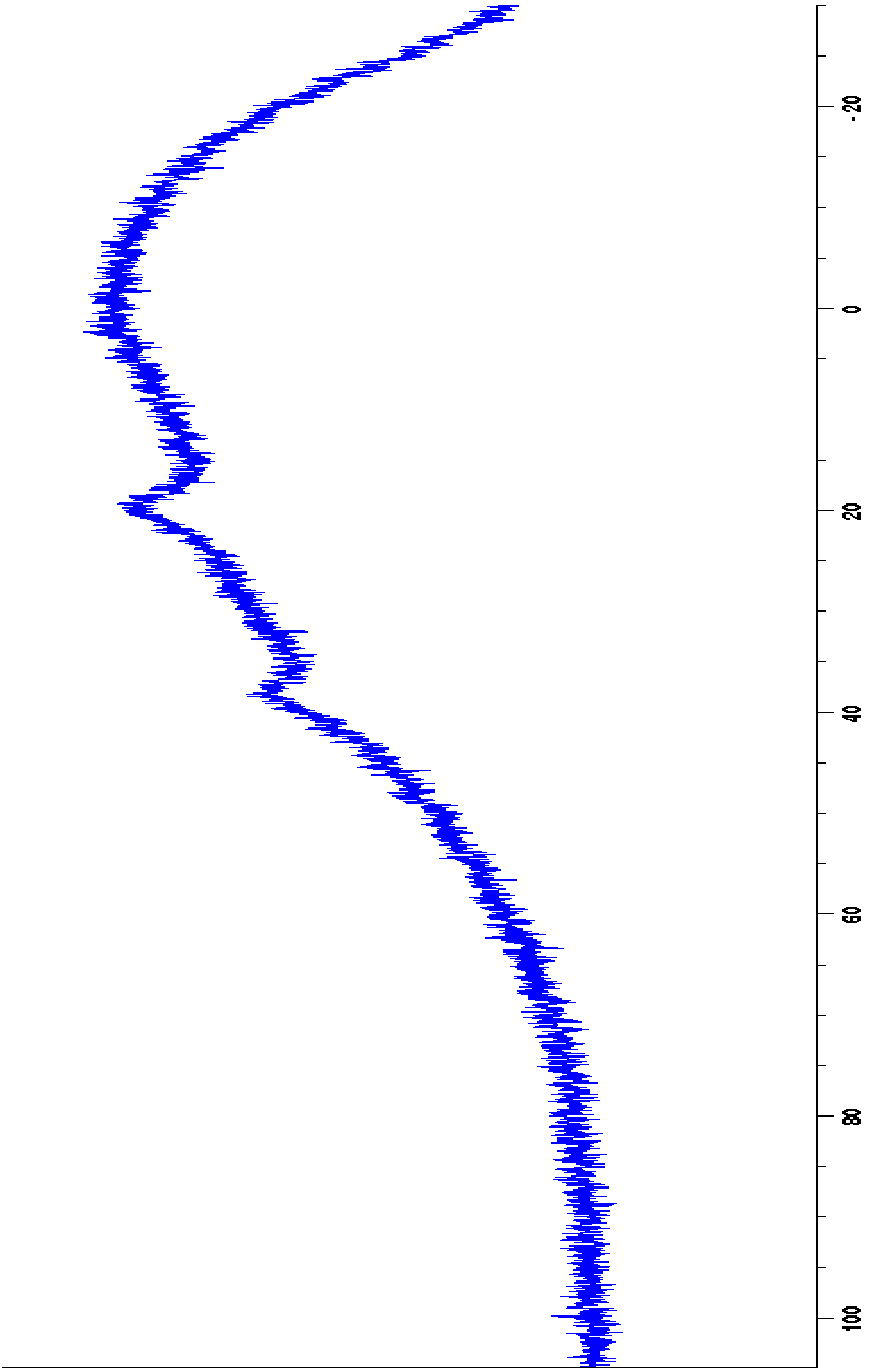

Figure S12. $^{13}C\{^{1}H\}$ NMR spectrum of **2** in C_6D_6.

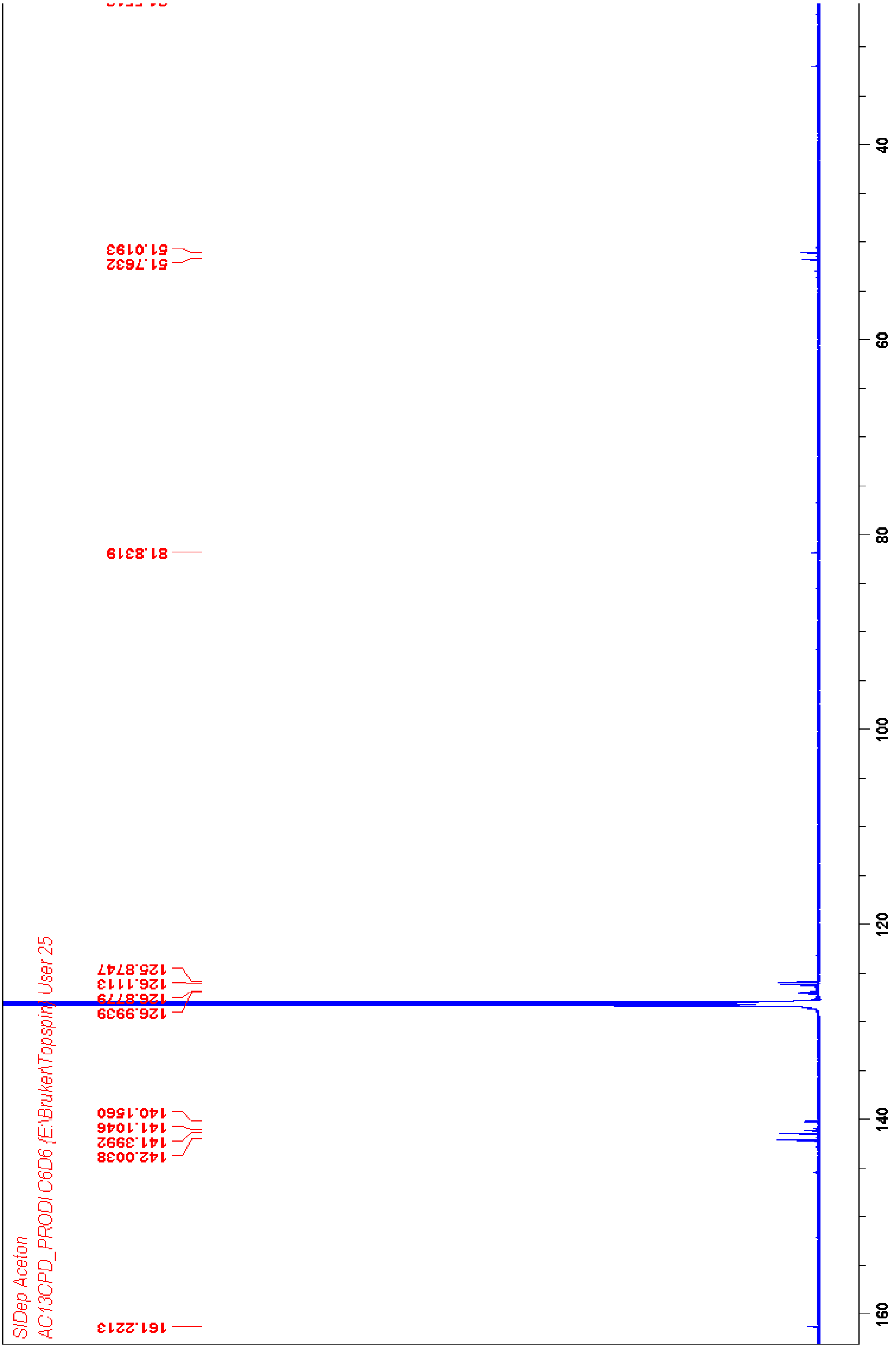

Figure S13. ^{1}H NMR spectrum of **3** in C_6D_6.

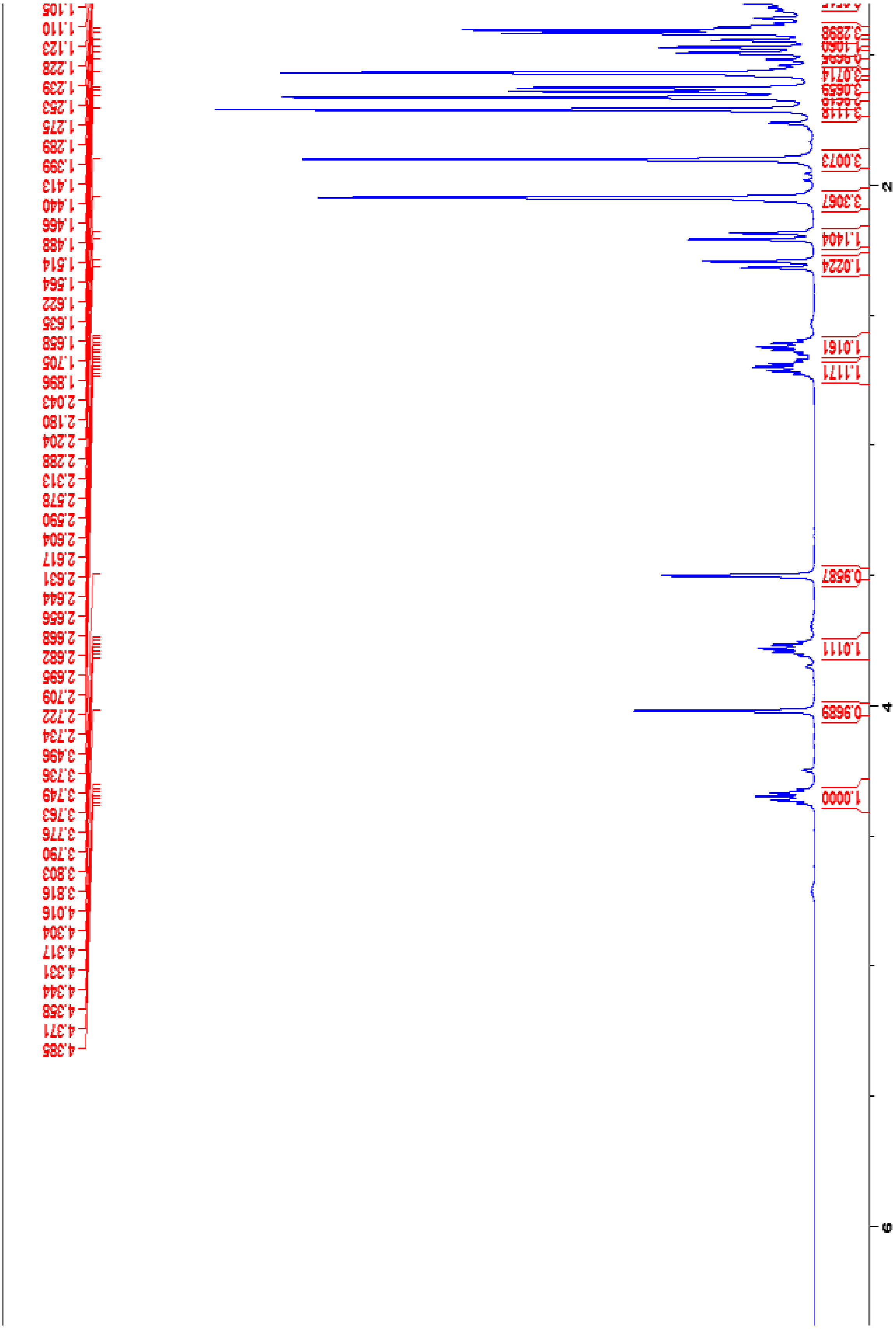

Figure S14. ^{11}B NMR spectrum of **3** in C_6D_6 recorded at 70 °C. The major set of resonances (picked in red) corresponds to tautomer **3a**, the minor set (in green) to tautomer **3b**.

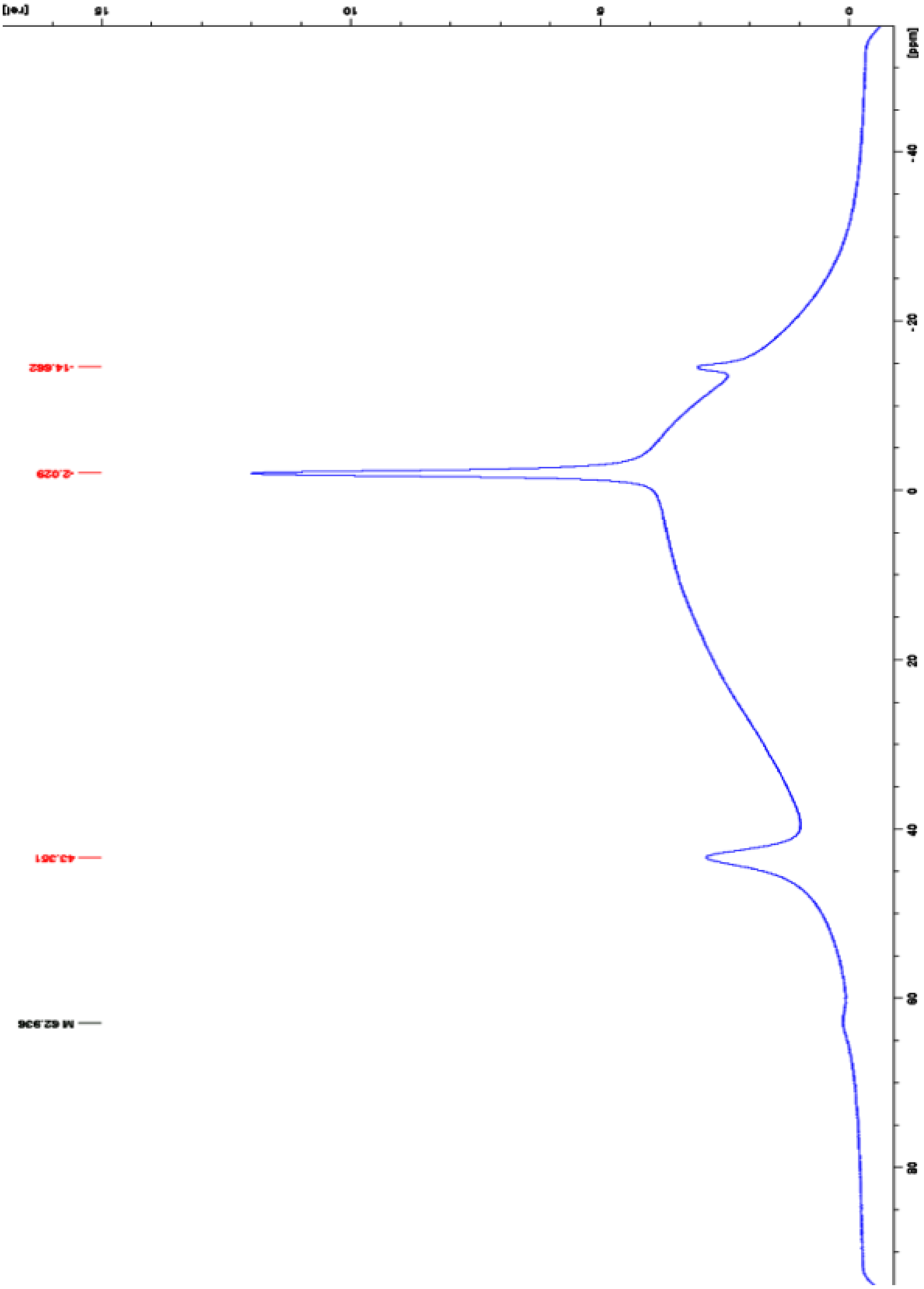

Figure S15. $^{13}C\{^{1}H\}$ NMR spectrum of **3** in C_6D_6.

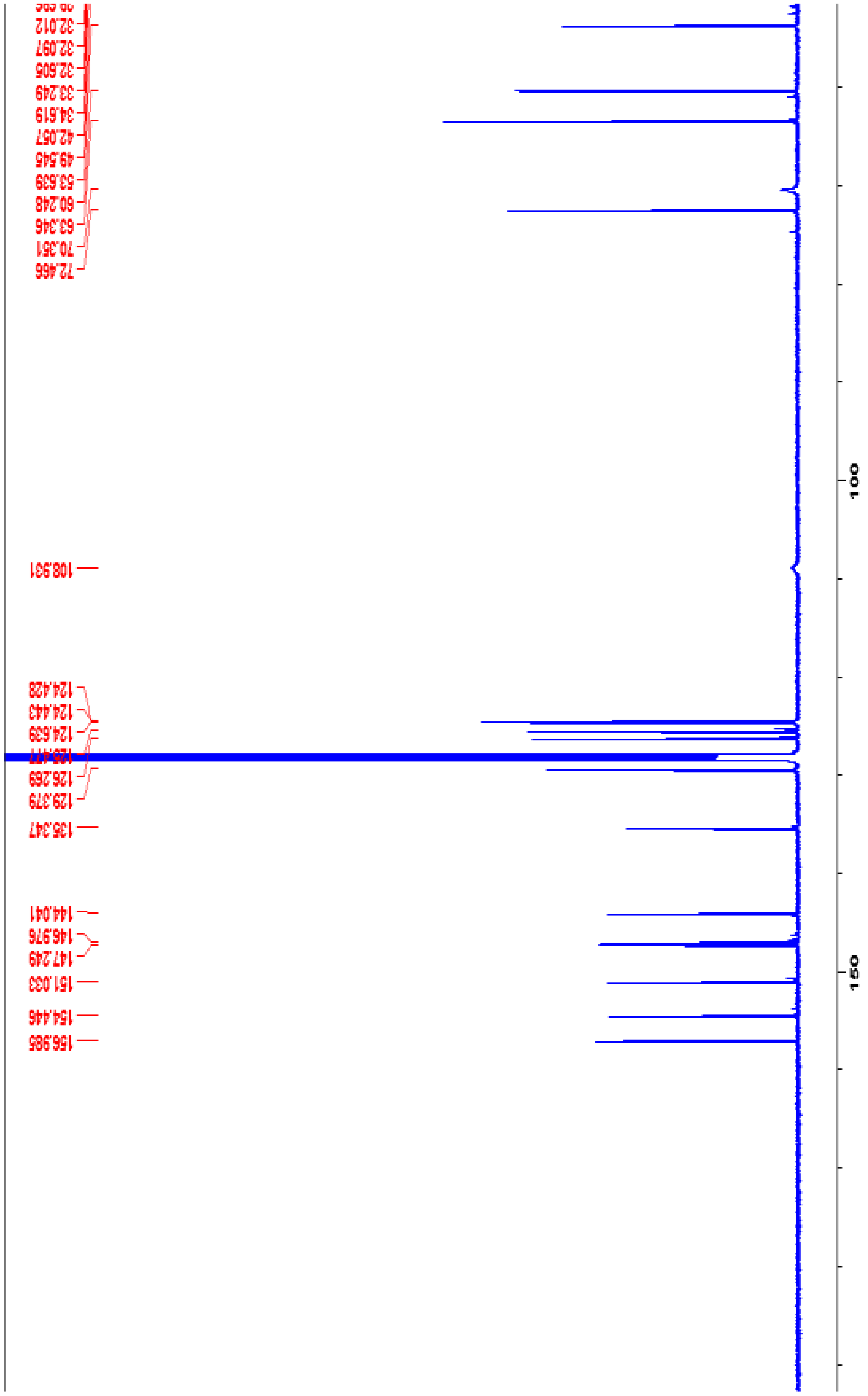

UV/Vis Spectra

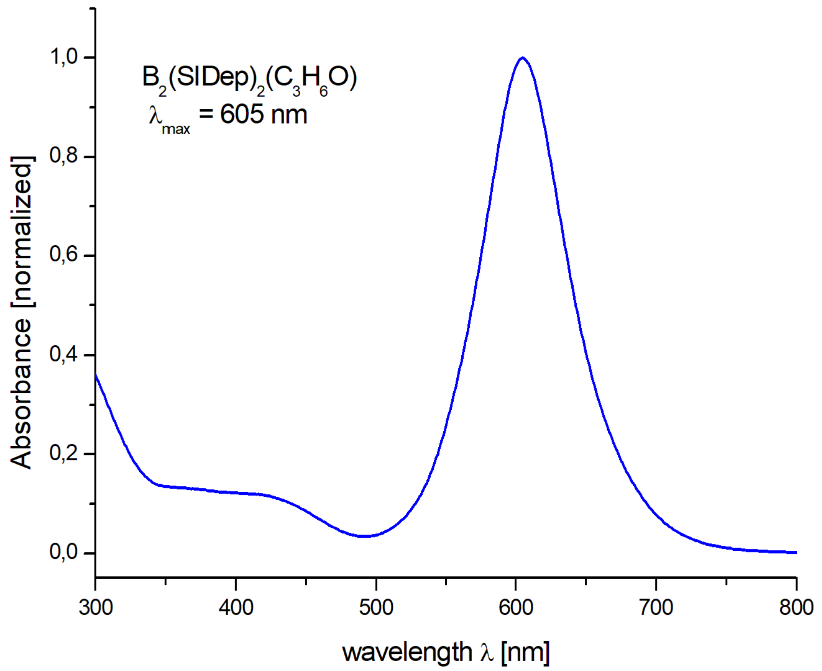

Figure S16. UV-vis absorption spectrum of **2** in pentane (c ≈ 2.0 x 10^{-5} M). λ_{max} = 605 nm.

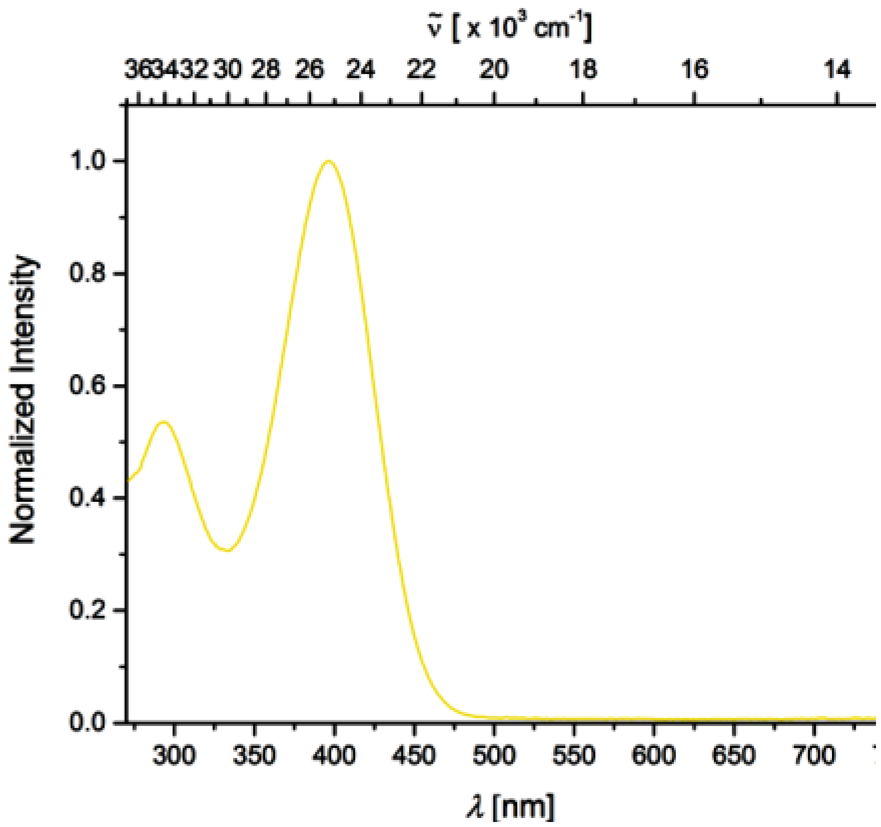

Figure S17. UV-vis spectrum of **3** in THF (c ≈ 6.2 x 10^{-5} M) . λ_{max}= 397 nm.

IR spectra

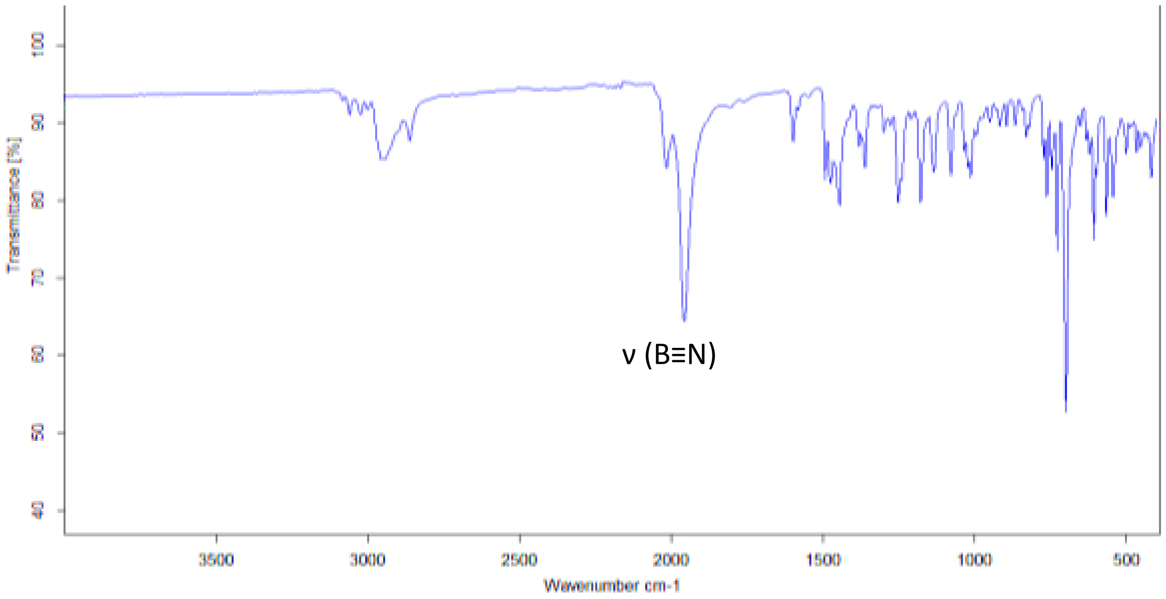

Figure S18. IR spectrum of **IV** in toluene.

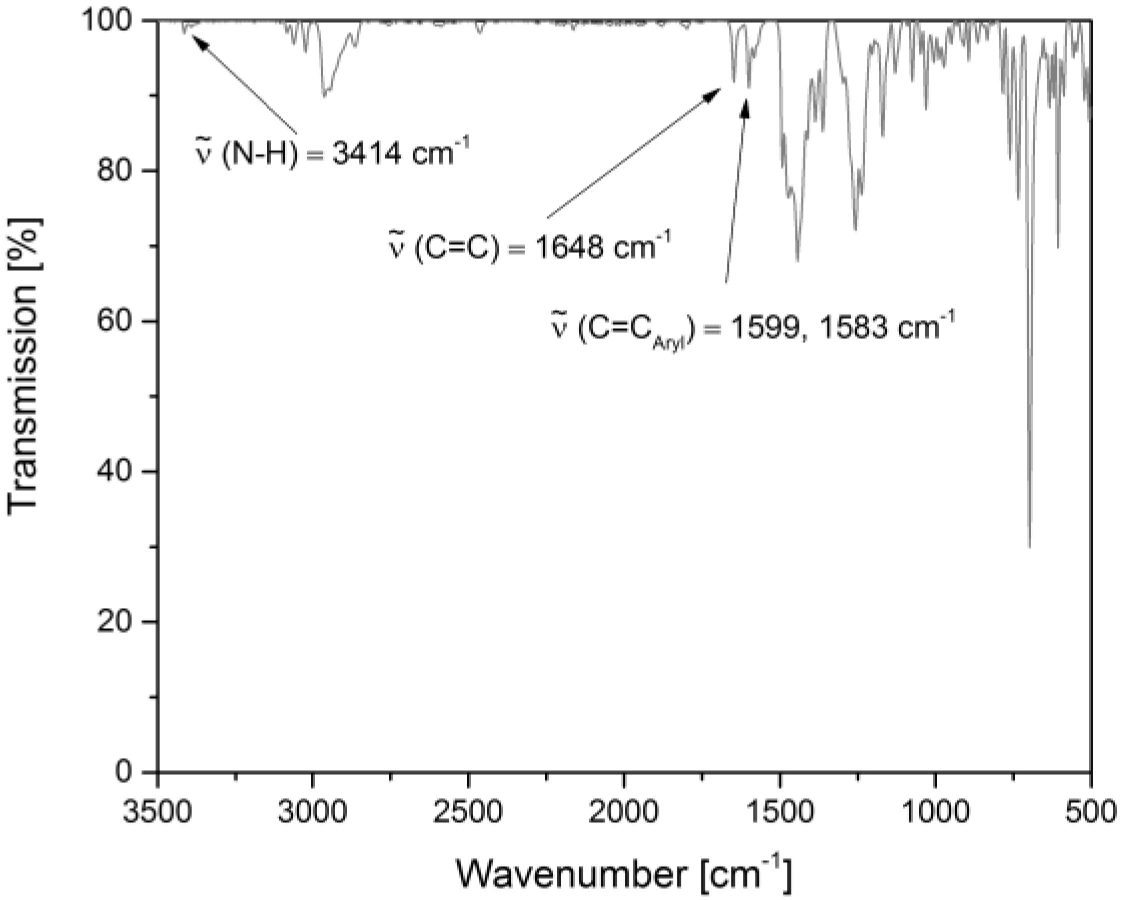

Figure S19. Solid-state IR spectrum of **1**.

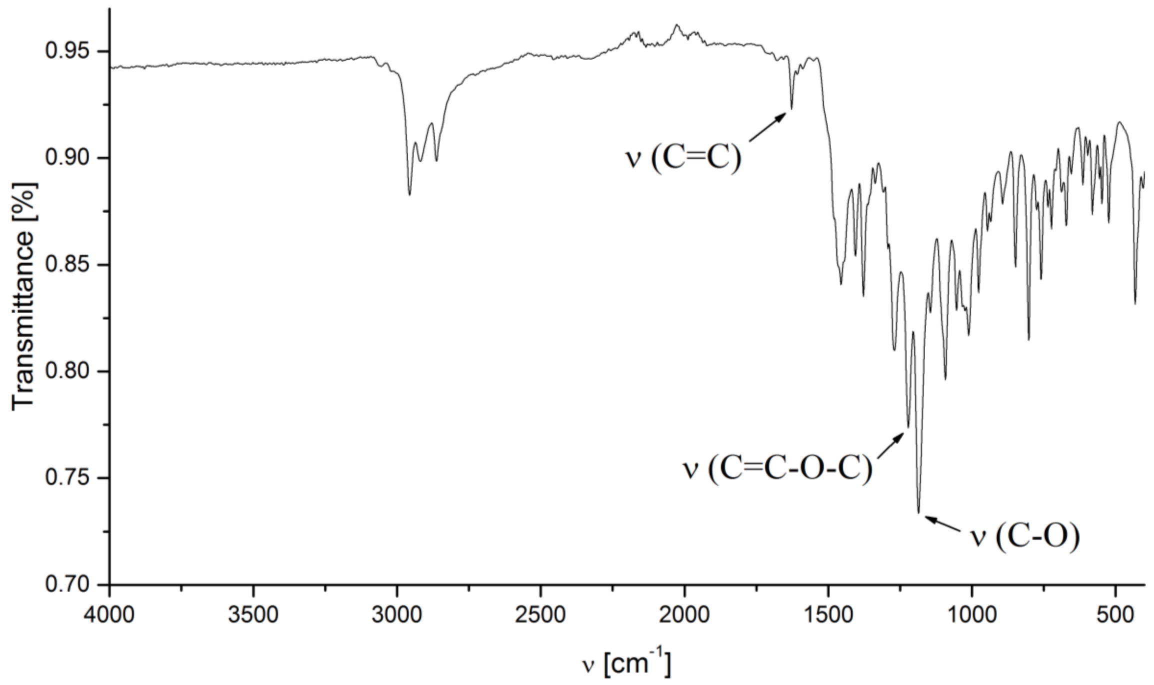

Figure S20. Solid-state IR spectrum of **2**.

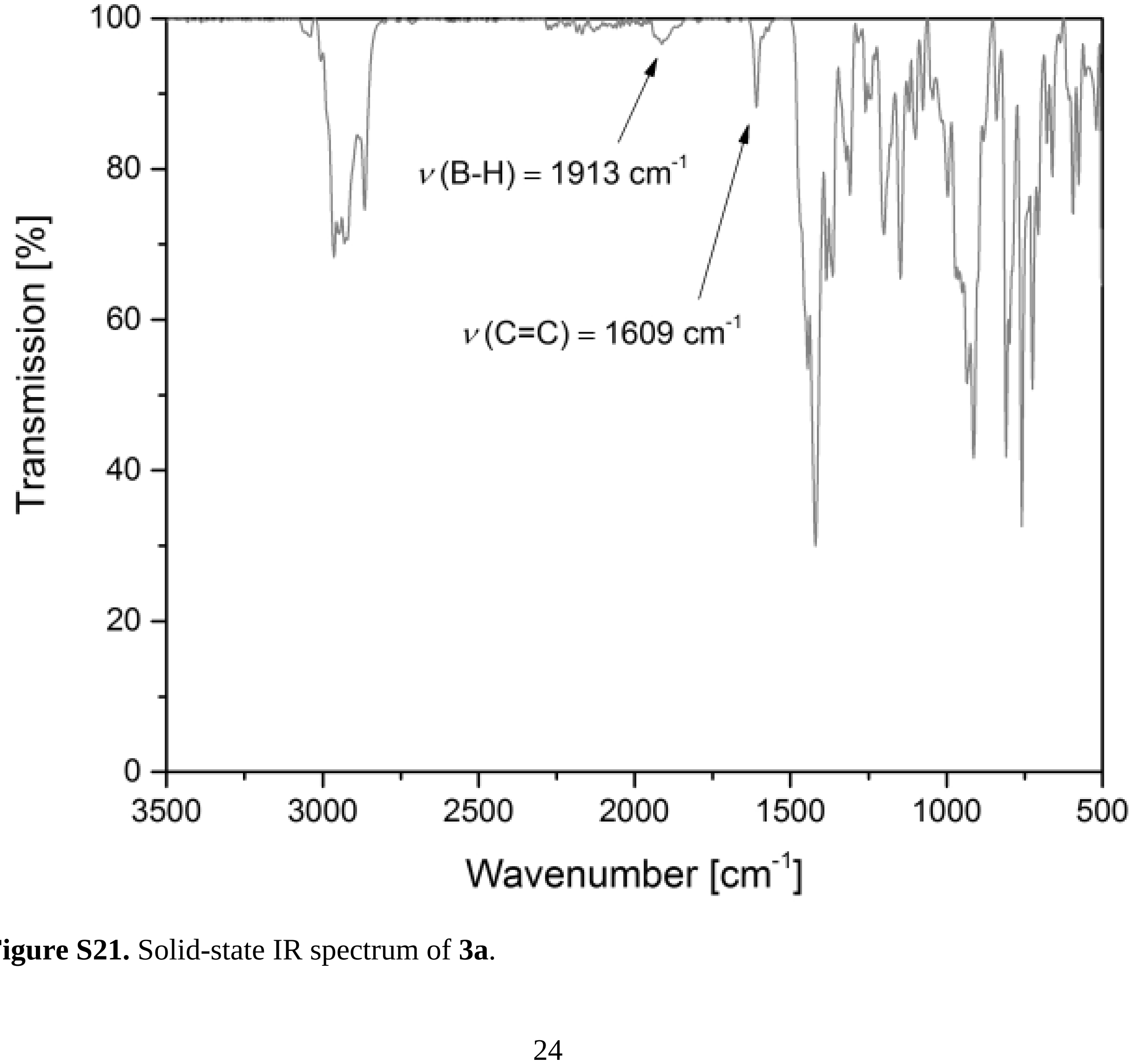

Figure S21. Solid-state IR spectrum of **3a**.

X-ray crystallographic data

Crystal data of all compounds were collected on a Bruker X8-APEX II diffractometer with a CCD area detector (compounds **1** and **2**) or a Bruker D8 Quest diffractometer with a CMOS area detector (**Ar*N(H)BCl(TMP)**, compounds **IV** and **3a**), both with *m*-layer mirror monochromated $Mo_{K\alpha}$ radiation. The structures were solved using intrinsic phasing methods,[5] refined with the ShelXL software package[6] and expanded using Fourier techniques. All non-hydrogen atoms were refined anisotropically. Hydrogen aroms were refined isotropically and assigned to idealised positions. CIF files of crystallographic structures have been deposited with the Cambridge Crystallographic Data Centre, CCDC numbers 1830169 (**Ar*N(H)BCl(TMP)**), 1830171 (**IV**), 1830168 (**1**), 1830420 (**2**) and 1830170 (**3a**).

Refinement details for **1**: A BUMP 0.01 restraint was applied to avoid intramolecular H-H contacts.

Refinement details for **2**: The asymmetric unit contains one molecule of the alkoxy(hydro)diborene and half a molecule of pentane positioned on an inversion center. The latter was modelled with C-C bond restraints of 1.53 Å and SIMU 0.005. Three of the ethyl substituents of the NHC ligand were two-fold disordered in the terminal methyl group: (C25 > C26) in a 77:23 ratio, (C38 > C39) in a 78:22 ratio and (C48 > C49) in a 56:44 ratio. The methylene carbon atom was kept shared between the two parts and its hydrogen atom modelled only for the major part. The C-C bond lengths of each part were restrained with SADI, and the ADPs with SIMU 0.01. The H-H distances between the methylene H atoms and the methyl H atoms of the minor part were restrained with DFIX –1.9 0.002 to avoid bumping.

Crystal data for **Ar*N(H)BCl(TMP)**: $C_{45}H_{52}BClN_2$, M_r = 667.14, colourless block, 0.302×0.26×0.17 mm^3, monoclinic space group $C2/c$, a = 26.740(10) Å, b = 9.4128(11) Å, c = 30.763(12) Å, β = 100.73(3)°, V = 7608(4) $Å^3$, Z = 8, ρ_{calcd} = 1.165 g·cm^{-3}, μ = 0.134 mm^{-1}, F(000) = 2864, T = 100(2) K, R_1 = 0.0666, wR^2 = 0.1474, 7492 independent reflections [$2\theta \leq 52.044°$] and 453 parameters.

Crystal data for **IV**: : $C_{45}H_{51}BN_2$, M_r = 630.68, colourless block, 0.29×0.24×0.168 mm^3, monoclinic space group $P2_1/c$, a = 12.303(5) Å, b = 12.532(3) Å, c = 24.192(17) Å,

β = 100.95(4)°, V = 3662(3) Å^3, Z = 4, ρ_{calcd} = 1.144 g·cm^{-3}, μ = 0.065 mm^{-1}, F(000) = 1360, T = 100(2) K, R_1 = 0.1478, wR^2 = 0.1431, 7476 independent reflections [2θ≤52.742°] and 440 parameters.

Crystal data for **1**: $C_{48}H_{57}BN_2O$, M_r = 688.76, colorless block, 0.108×0.101×0.067 mm^3, triclinic space group $P\bar{1}$, a = 12.799(2) Å, b = 13.808(2) Å, c = 13.844(2) Å, α = 64.938(4)°, β = 70.414(4)°, γ = 66.334(4)°, V = 1989.2(6) Å^3, Z = 2, ρ_{calcd} = 1.150 g·cm^{-3}, μ = 0.067 mm^{-1}, F(000) = 744, T = 100(2) K, R_1 = 0.0538, wR^2 = 0.1391, 8139 independent reflections [2θ≤52.744°] and 477 parameters.

Crystal data for **2**: $C_{49}H_{66}B_2N_4O$•$(C_5H_{12})_{0.5}$, M_r = 784.79, green block, 0.265×0.191×0.093 mm^3, triclinic space group $P\bar{1}$, a = 10.756(11) Å, b = 12.715(12) Å, c = 18.183(13) Å, α = 81.15(5)°, β = 87.75(3)°, γ = 68.37(4)°, V = 2284(4) Å^3, Z = 2, ρ_{calcd} = 1.141 g·cm^{-3}, μ = 0.067 mm^{-1}, F(000) = 854, T = 100(2) K, R_1 = 0.0616, wR^2 = 0.1617, 9261 independent reflections [2θ≤52.744°] and 598 parameters.

Crystal data for **3a**: $C_{43}H_{68}B_2N_2O$, M_r = 650.61, yellow block, 0.202×0.169×0.147 mm^3, monoclinic space group $P2_1/n$, a = 9.499(4) Å, b = 22.755(7) Å, c = 19.041(6) Å, β = 91.58(3)°, V = 4114(2) Å^3, Z = 4, ρ_{calcd} = 1.050 g·cm^{-3}, μ = 0.060 mm^{-1}, F(000) = 1432, T = 100(2) K, R_1 = 0.0551, wR^2 = 0.1128, 8385 independent reflections [2θ≤52.744°] and 454 parameters.

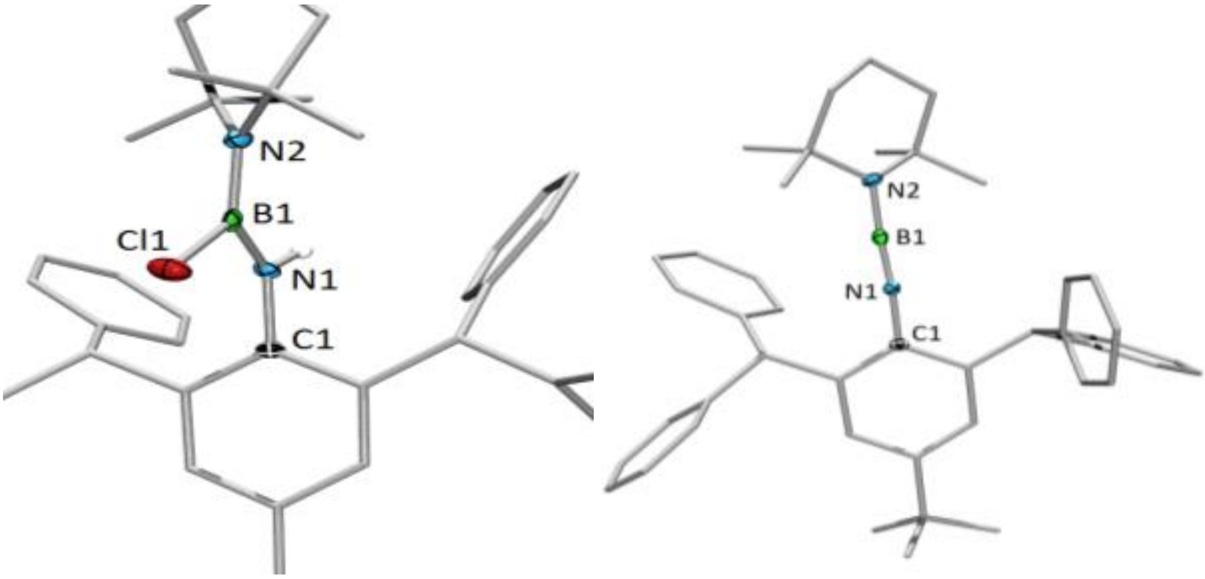

Figure S22. Crystallographically determined solid-state structures of **Ar*N(H)BCl(TMP)** (left) and **IV** (right). Atomic displacement ellipsoids depicted at 50% probability level. Atomic displacement ellipsoids of peripheral substituents omitted for clarity. Hydrogen atoms omitted, except for that bound to nitrogen. Selected bond lengths (Å) and angles (°): **Ar*N(H)BCl(TMP)** N2-B1 1.421(4), B1-N1 1.394(4), B1-Cl1 1.828(3), N2-B1-N1 126.2(3), B1-N1-C1 128.4(3); **IV** N2-B1 1.385(3), B1-N1 1.252(3), N2-B1-N1 175.5(3), B1-N1-C1 163.3(2).

DFT calculations

Methodology

For the iminoborane IV + acetone reaction

Geometry optimisations were performed in the gas phase using the Gaussian09 computational package[7] without any symmetry restriction. We chose the DFT formalism for our calculations carried out with the PBE0 hybrid density-functional that includes 25% of HF exchange in its formulation.[8] Grimme's D3 method[9] for incorporating dispersion effects was explicitly included in the geometry optimisations. The electronic configurations of hydrogen, carbon, nitrogen, boron and oxygen atoms were described with Pople's double-ζ 6-31G* basis set containing one polarisation function.[10] Later, harmonic frequency calculations were done for two reasons: 1) to verify the nature of the stationary points on the potential energy surface (reaction intermediates and products must have zero negative eigenvalues in the Hessian and transition states one and only one negative eigenvalue which corresponds to the reaction coordinate); and 2) for incorporating the zero-point energy as well as thermal corrections to our reported energy values at 353.15 K (80 °C) and 1 atm. Single-point calculations on the PBE0-optimised geometries were done by using the M06 functional[11] with the same basis set for all the atoms. Moreover, in these single-point calculations, the solvent effect was also added by utilising Truhlar and coworkers' SMD solvation model[12] using *n*-hexane as the solvent of reaction.

For the diboryne II and cumulene III + acetone reactions

For the exploration of the potential energy surface of the reaction between acetone and diboryne **II** (Schemes 3 and 4) and cumulene **III** (Schemes 5 and 6) a combined semi-empirical-DFT approach (two-layered ONIOM scheme)[13] was used to explore the potential energy surface (PES) of our studied system. The inner part of the molecules (NHC and cAAC rings, B-B bond and acetone) was treated with the highly parametrised, empirical hybrid density-functional M06-2X developed by Zhang and Truhlar,[11] in combination with the split-valence triple-ζ quality basis set with polarisation and diffuse functions 6-311+G(d). For the outer parts, the 2,6-diethylphenyl (Dep) and 2,6-diisopropylphenyl (Dip) substituents and methyl groups on cAAC were defined as the steric hindrance contributors, described at the semi-empirical PM6 level[14] which provides a good model for weak interactions such as

hydrogen bonding, and has been shown to be significantly better for reproducing *ab initio* TS structures and barrier heights.[15] Electronic embedding was also utilised to incorporate the partial charges of the PM6 region into the DFT Hamiltonian.[16] This computational approach is called ONIOM(M06-2X/6-311+G(d):PM6). The final computed S-value was low (-0.073293) which shows that the ONIOM2 partition Scheme is very suitable for this study.

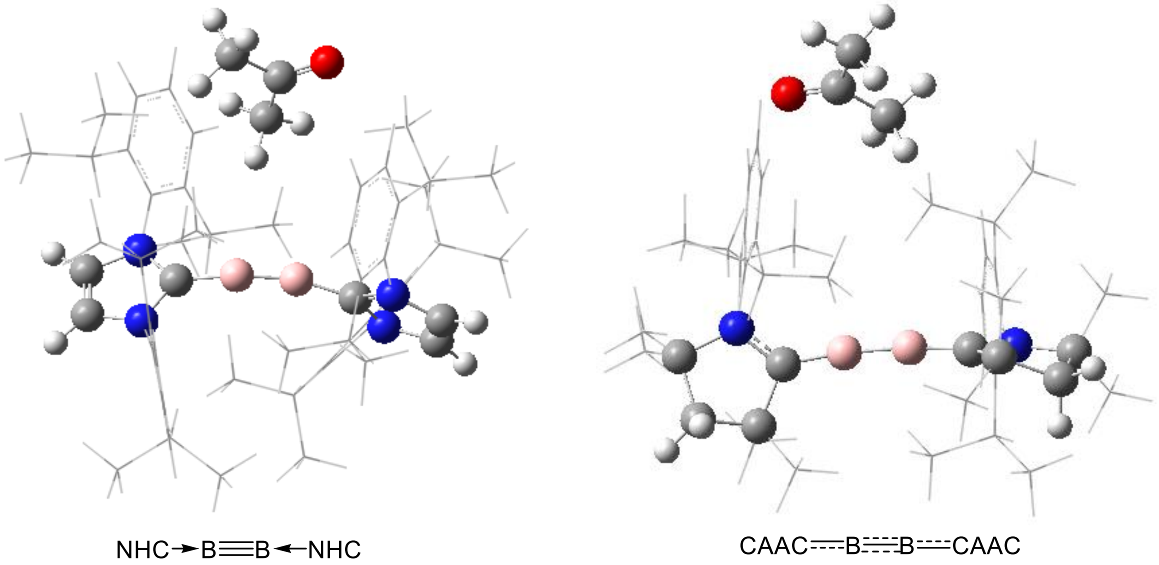

Figure S23. Partition scheme used for ONIOM calculations. M06-2X/6-311+G(d) layer is represented in ball and stick mode whereas the PM6 layer is shown in wireframe mode.

For the optimisation of 3b and calculated ^{11}B NMR shifts

NMR–GIAO calculations for the optimized geometries of the two isomeric products **3a** and **3b** were carried out with the same density functional as for the ONIOM calculations but with a bigger basis set from Weigend and Ahlrichs[17] for the full system (M06-2X/TZVPD). The calculated ^{11}B shielding values σ were referenced to B_2H_6 as the primary reference point. The resulting chemical shifts δ were converted to the standard $F_3B{\cdot}OEt_2$ scale using the experimental value of +16.6 ppm for $\delta(B_2H_6)$:[18]

$$\delta(^{11}B_{comp}) = \sigma(^{11}B_{B2H6}) - \sigma(^{11}B_{comp}) + 16.6$$

For the investigation of the absorption spectrum of 2

TD-DFT calculations were set up over the optimized geometry of compound **2** with different functionals but keeping constant the basis set [6–311+G(d)] with and without the ONIOM

formalism as well as Truhlar and coworkers' SMD solvation model[12] using *n*-pentane as the solvent of reaction (see Table S1). From our benchmark, we conclude that the level of theory with the closest result to the experimental value was lc-ωPBE/6–311+G(d).

Table S1. Comparison of the performance among different levels of theory using TD-DFT.

**Level of Theory**	**Maximum absorption peak (nm)**	**Error** (nm)
oniom(cam-b3lyp/6-311+g(d):pm6)	543.4	61.6
(smd:n-pentane)cam-b3lyp/6-311+g(d)	635.5	–30.5
(smd:n-pentane)τ-hcth$_{hyb}$/6-311+g(d)	700.8	–95.8
cam-b3lyp/6-311+g(d)	633.7	–28.7
(smd:n-pentane)lc-ωpbe/6-311+g(d)	595.2	9.8
(smd:n-pentane)m062x/6-311+g(d)	641.5	–36.5
lc-ωpbe/6-311+g(d)	593.5	11.5
Experimental value	605	

__**TDDFT results for compound 2**__

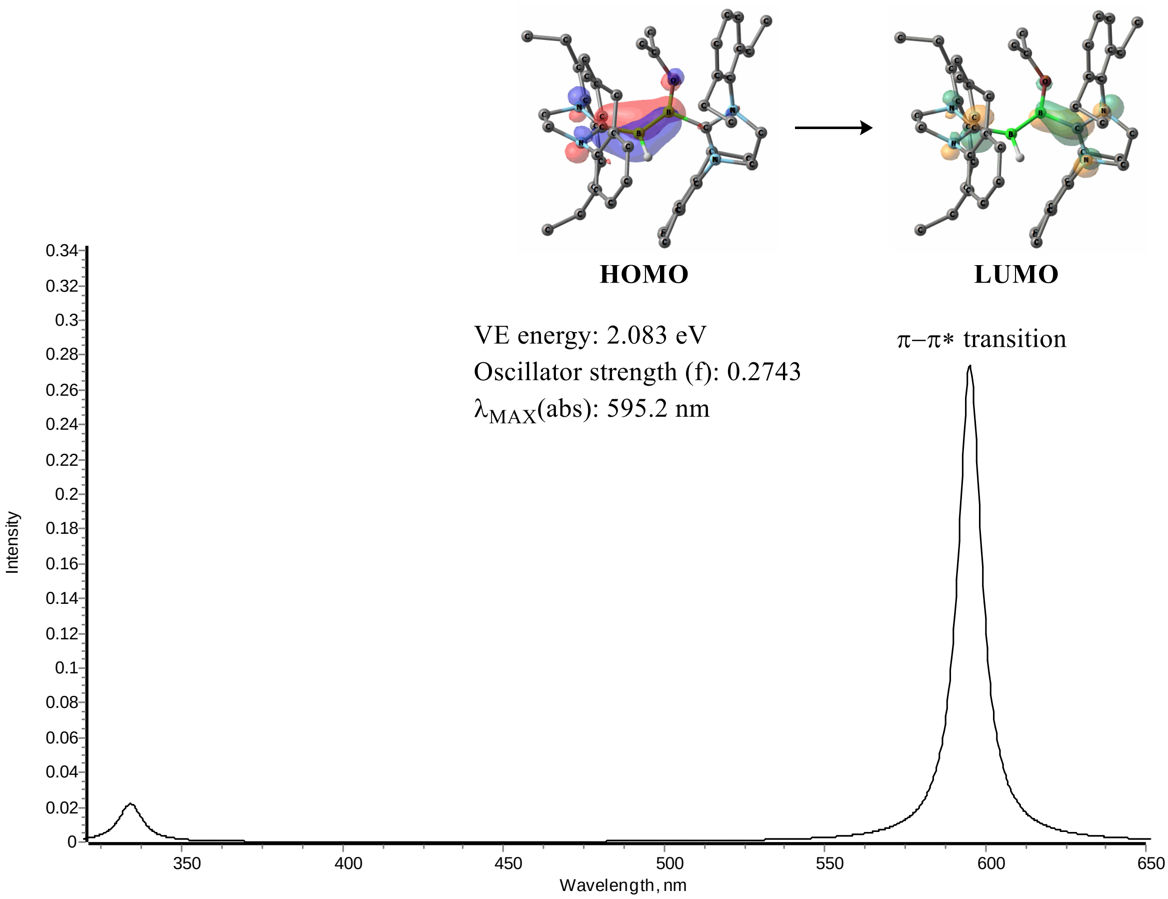

Figure S24. UV-Vis spectrum of compound **2** calculated at the (smd:n-pentane)lc-ωPBE/6-311+g(d) level.

Geometry optimisation and ^{11}B NMR shift calculations of 3a and 3b

Based on the known propensity for cAAC-supported hydroboranes to undergo 1,2-hydrogen shifts from boron to an adjacent cAAC carbene centre[19] we first considered that isomer **3b** might be tautomer $\mathbf{3_{taut}}$, with the protonated cAAC ligand bound to B2 instead of B1. However, the calculated ^{11}B NMR chemical shifts for $\mathbf{3_{taut}}$ were far from matching the experimental ones, whereas those calculated for **3a** were in relatively good agreement (Fig. S55). Besides, according to the ONIOM(M06-2X/6-311+G(d):PM6) energy values, $\mathbf{3_{taut}}$ lies 8.4 kcal·mol^{-1} higher in energy than **3a**, which contradicts the experimental observation of both isomers.

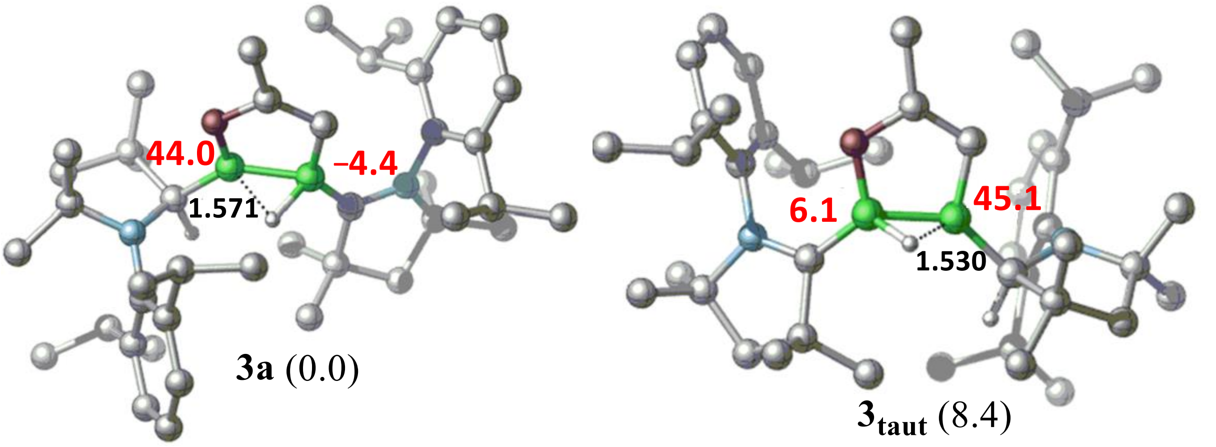

Figure S25. Optimized structures of **3a** and $\mathbf{3_{taut}}$ at the ONIOM(M06-2X/6-311+G(d):PM6) level. Calculated ^{11}B NMR chemical shift in red (ppm). Experimental shifts for comparison: **3a**: δ = 42.8 and −1.9 ppm; **3b**: δ = 63.0 and –15.0 ppm.

Since **3a** presents two stereocenters, one at the four-coordinate boron centre, which is locked by the B_2C_2O ring, and one at the protonated cAAC carbon bound to the second boron centre, the other possibility was that **3a** and **3b** could be diastereomers. This would also fit the observation that they do not exchange in solution even at high temperatures. To test this, the geometries and ^{11}B NMR signals of the two possible diastereomeric pairs of product **3a/b** were computed. The predicted NMR signals adequately match the experimental ones (δ_{exp} = 42.8 and −1.9 ppm) for (R^C,R^B)/ (S^C,S^B)-**3a** pair with a bridging hydride: δ_{calc} = 44.0 and −4.4 ppm (Δ(δ) ≈ +/– 2 ppm, Fig. S56). The calculations for the diastereomeric (R^C,S^B)/ (S^C,R^B) pair showed that the form with a non-bridging hydrogen is the most likely. The predicted NMR signals adequately match the experimental ones (δ_{exp} = 63.0 and –15.0 ppm) for the (R^C,S^B)/ (S^C,R^B)-**3b** pair with a non-bridging hydride: δ_{calc} = 65.4 and −18.9 ppm (Δ(δ) ≈ +/– 3

ppm, Fig. S56). The relative energy of (R^C,S^B)/ (S^C,R^B)-**3b**, at 3.1 kcal·mol^{-1} above (R^C,R^B)/ (S^C,S^B)-**3a**, is consistent with the experimentally observed ratio of 92:8.

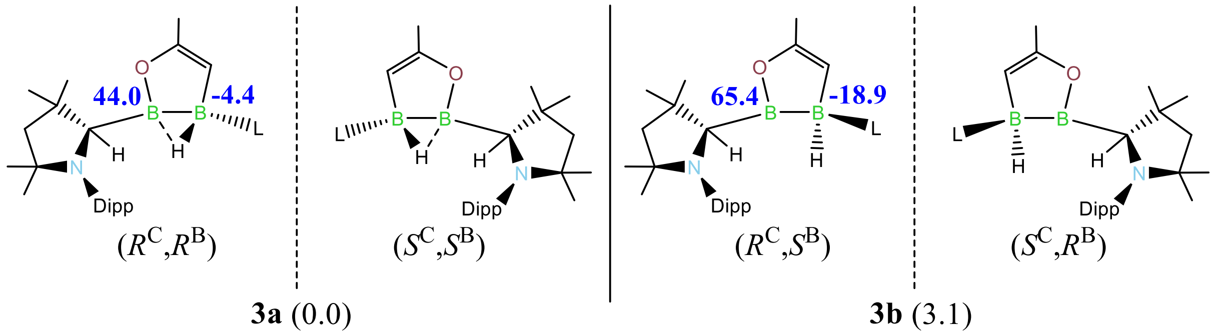

Figure S26. Calculated ^{11}B NMR shifts in blue (ppm) for the optimized geometries of the diastereomeric pairs **3a** and **3b** calculated at the ONIOM(M06-2X/6-311+G(d):PM6) level. Relative energy values in brackets are in kcal·mol^{-1}.

Reaction mechanisms

For the iminoborane IV + acetone reaction

The reaction of iminoborane **IV** with acetone was investigated computationally. The reaction mechanism is depicted in Scheme S2. First, acetone coordinates to boron through transition state **TS1$_1$** ($\Delta G^{\ddagger}$ = 7.6 kcal·mol^{-1}) producing intermediate **I1$_1$** which is 5.8 kcal·mol^{-1} higher in energy than the initial reactants. The proton transfer (**TS2$_1$**, $\Delta G^{\ddagger}$ = 4.0 kcal·mol^{-1}) then occurs readily to yield the *cis* form of the addition product, intermediate **I2$_1$**, the formation of which is exergonic by −21.0 kcal·mol^{-1}. Finally, the *trans* isomer **1** is obtained by rotation around the B–N bond and is 5.6 kcal·mol^{-1} more stable than the *cis* form. The total reaction energy was therefore highly exergonic at −26.6 kcal·mol^{-1}.

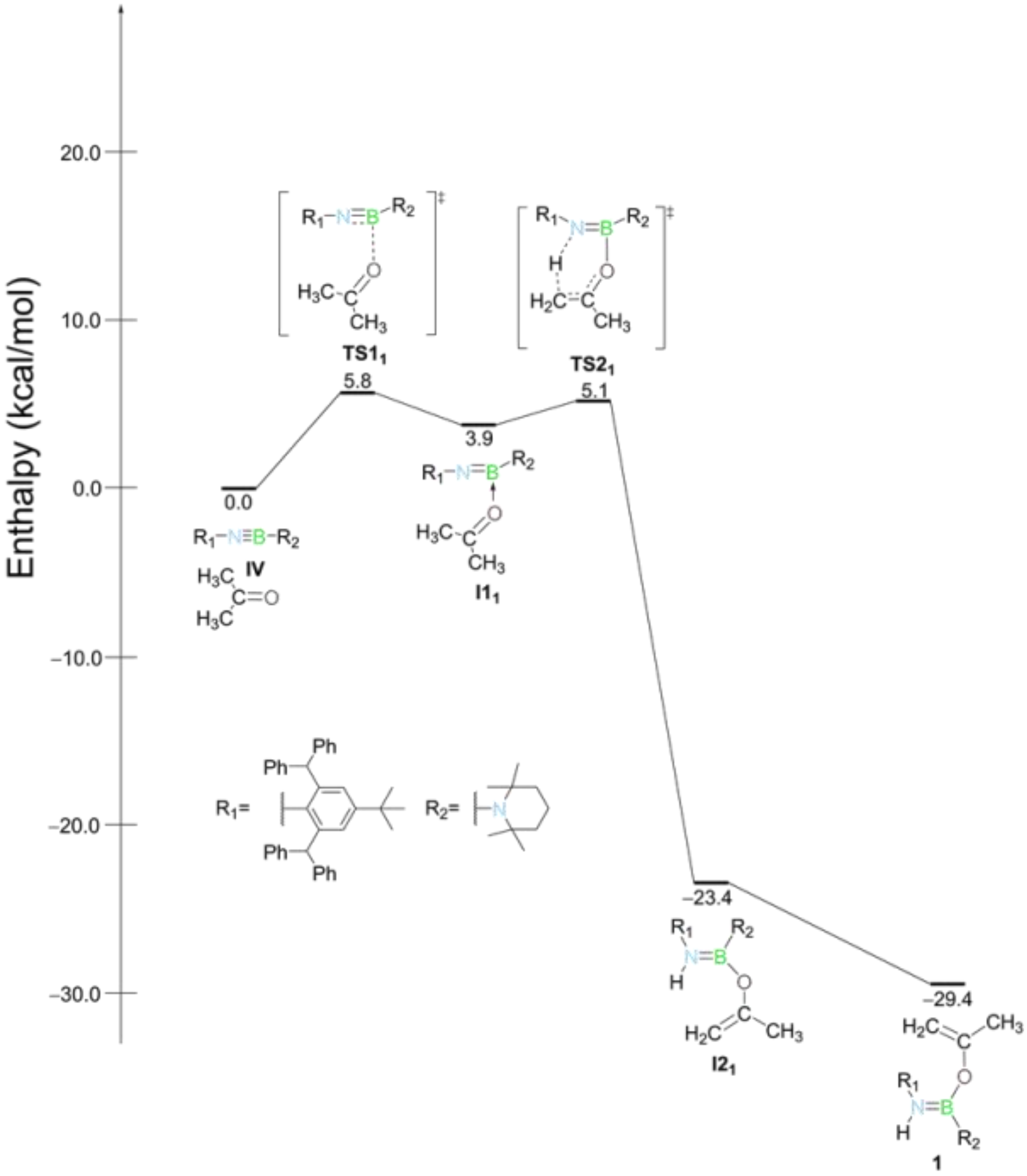

Scheme S1. Enthalpy profile of the proposed reaction mechanism between acetone and iminoborane **IV**.

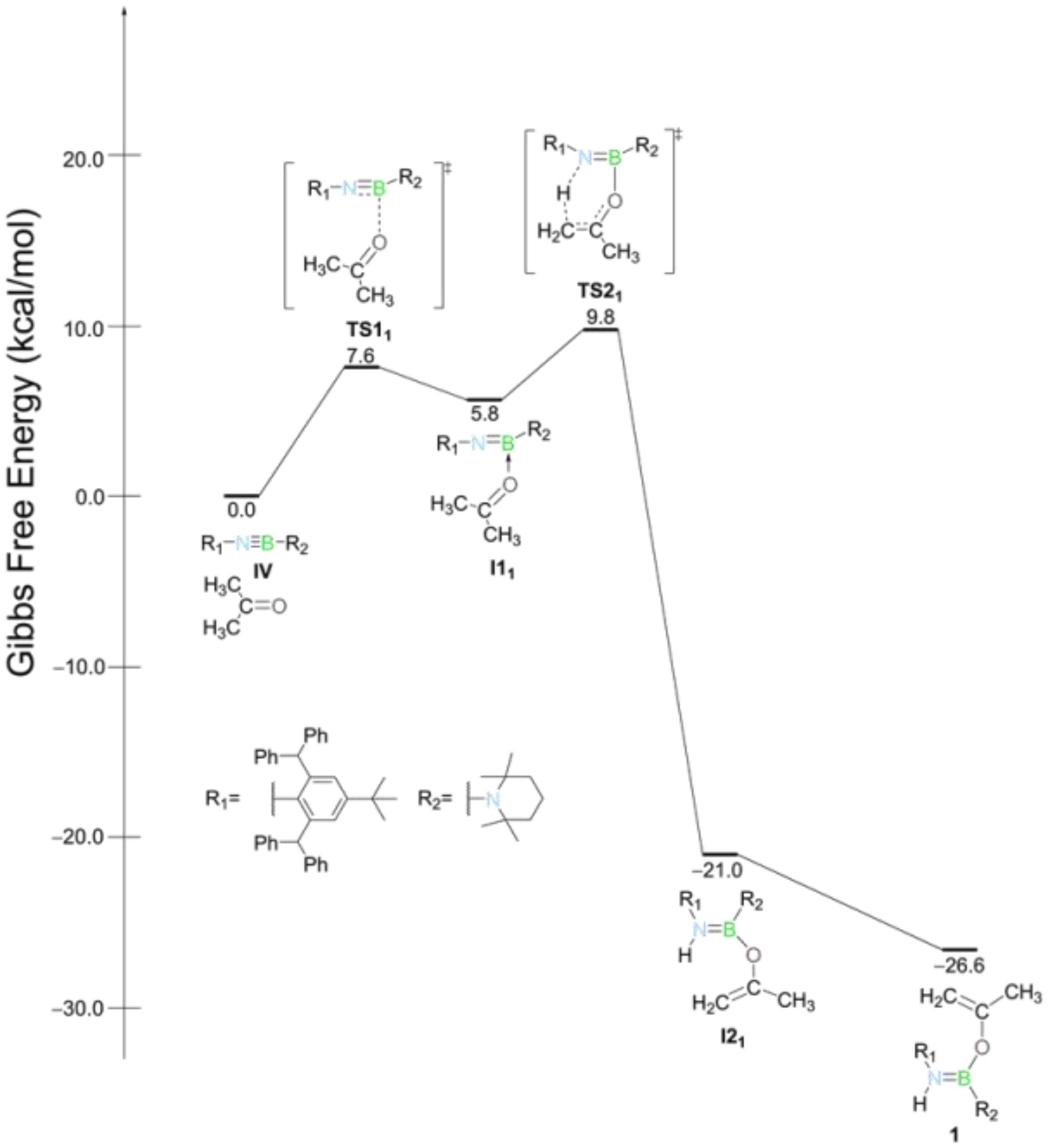

Scheme S2. Free energy profile of the proposed reaction mechanism between acetone and iminoborane **IV**.

For comparison with the hitherto proposed mechanism of acetone addition we also calculated the mechanism of the [2+2] cycloaddition between acetone and iminoborane **IV** to yield the 1,3,2-oxaboretidine **A** (Schemes 3 and 4). The energy barrier, calculated from transition state $TS_{\mathbf{IV-A}}$, is 10.5 kcal·mol^{-1}. Even though the reaction is more exergonic (–29.4 kcal·mol^{-1}) than for the formation of **IV** (–26.6 kcal·mol^{-1}) our results showed that the rate-limiting energy barrier for the formation of **IV** (7.8 kcal·mol^{-1}) at 70 °C is ca. 3 kcal·mol^{-1} more favourable than for the formation of **A** (10.5 kcal·mol^{-1}).

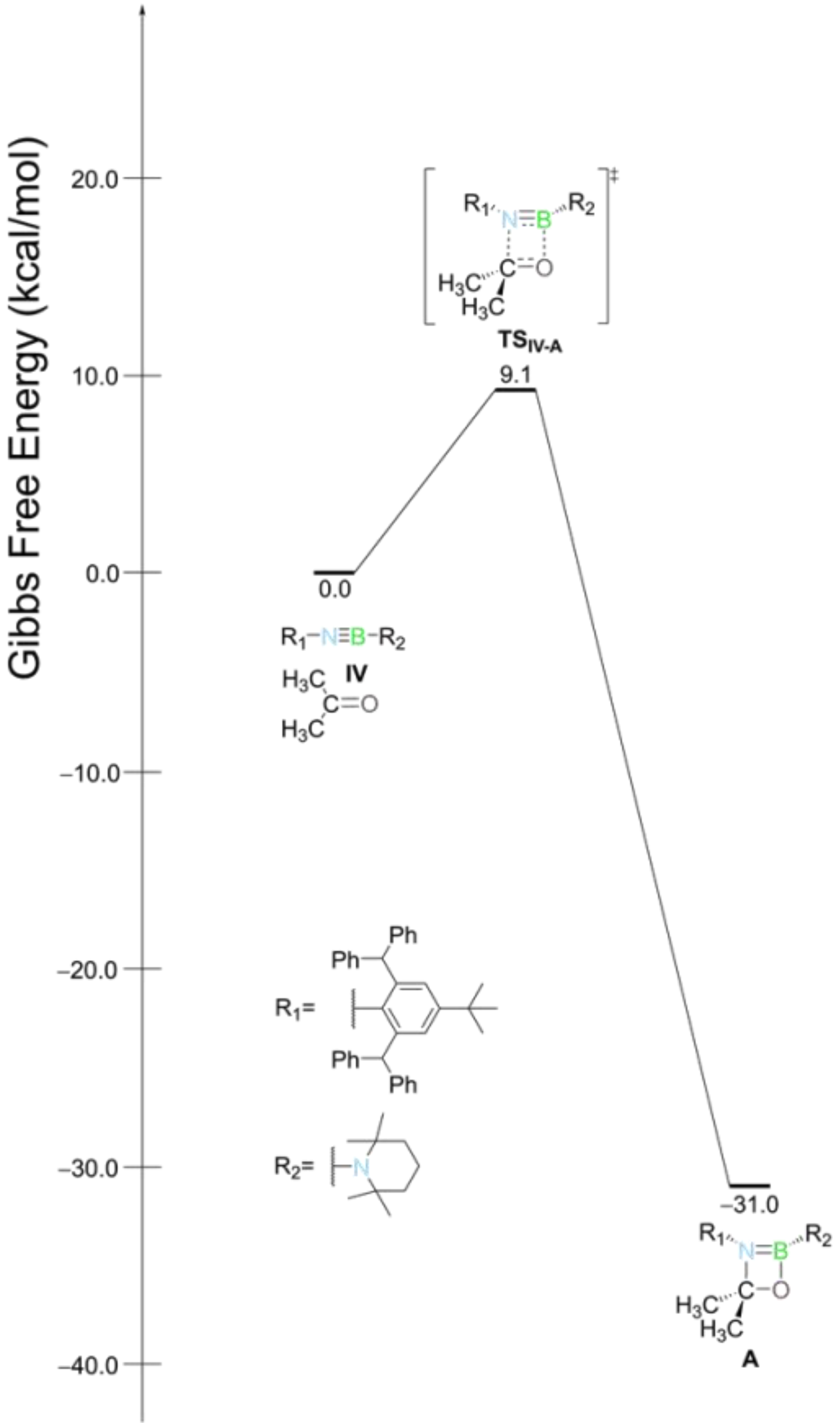

Scheme S3. Enthalpy profile of the proposed [2+2] cycloaddition mechanism between acetone and iminoborane **IV**.

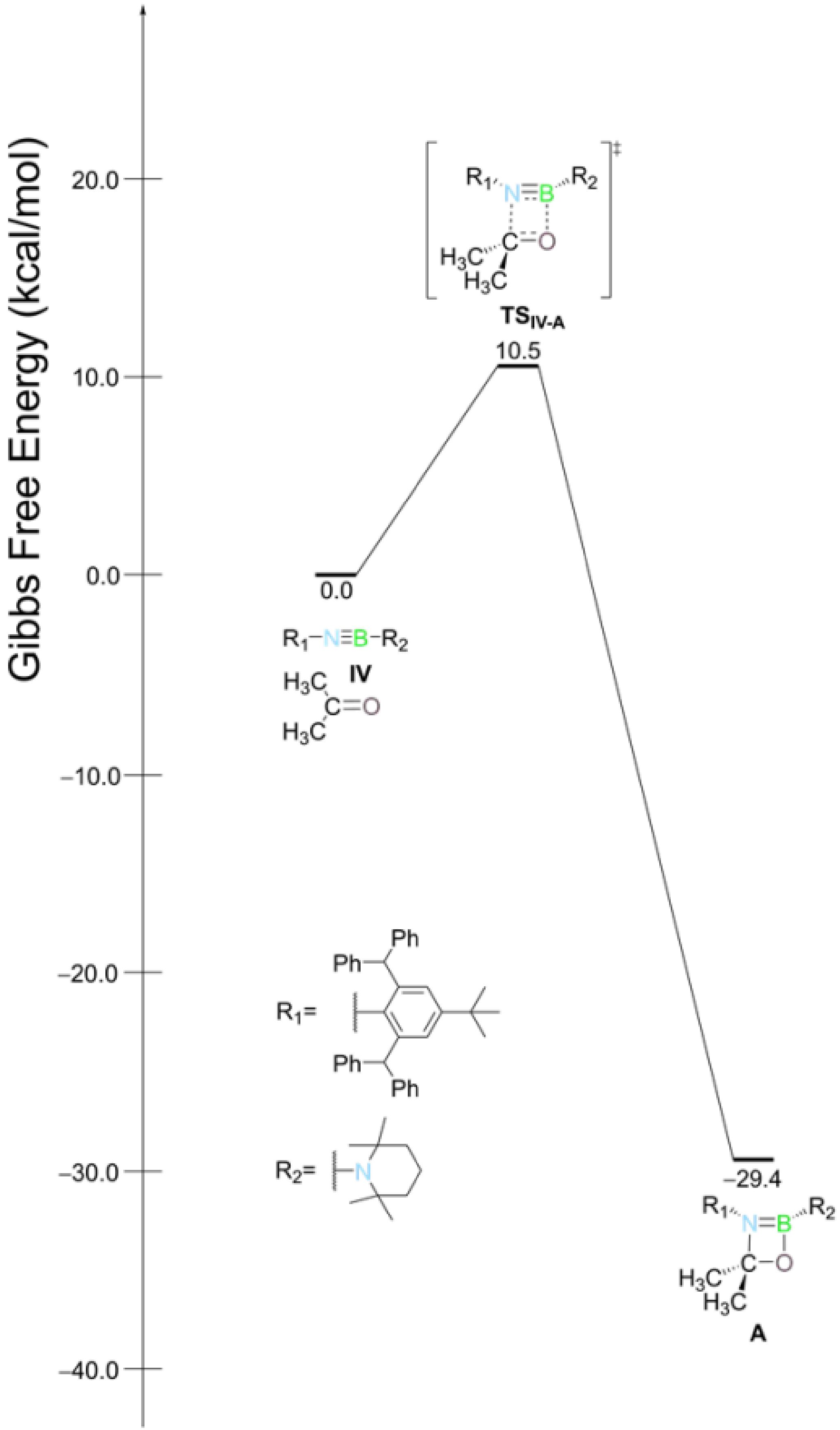

Scheme S4. Free energy profile of the proposed [2+2] cycloaddition mechanism between acetone and iminoborane **IV**.

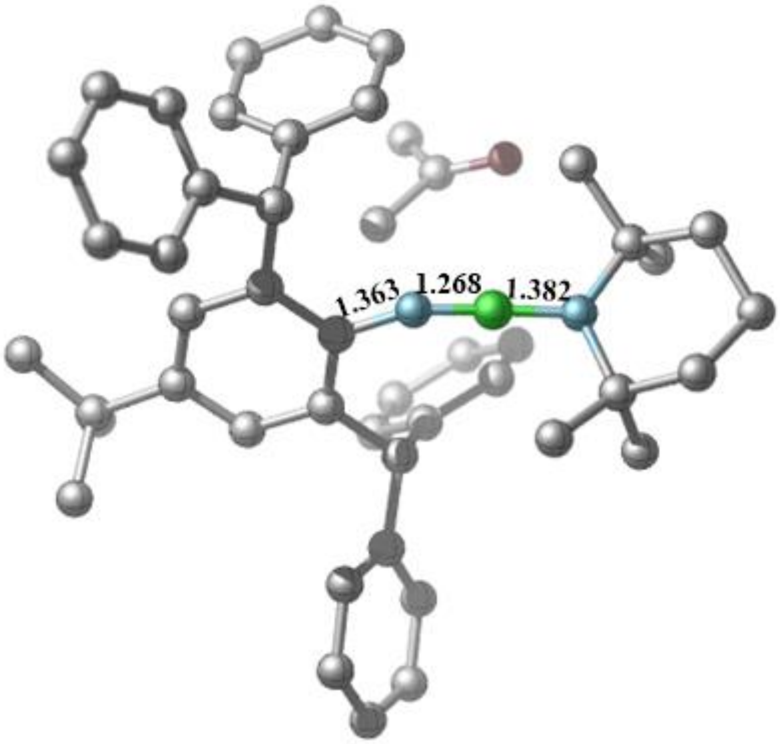

Figure S27. Optimised structure of **IV** + acetone. Distances in Å and hydrogens omitted for clarity.

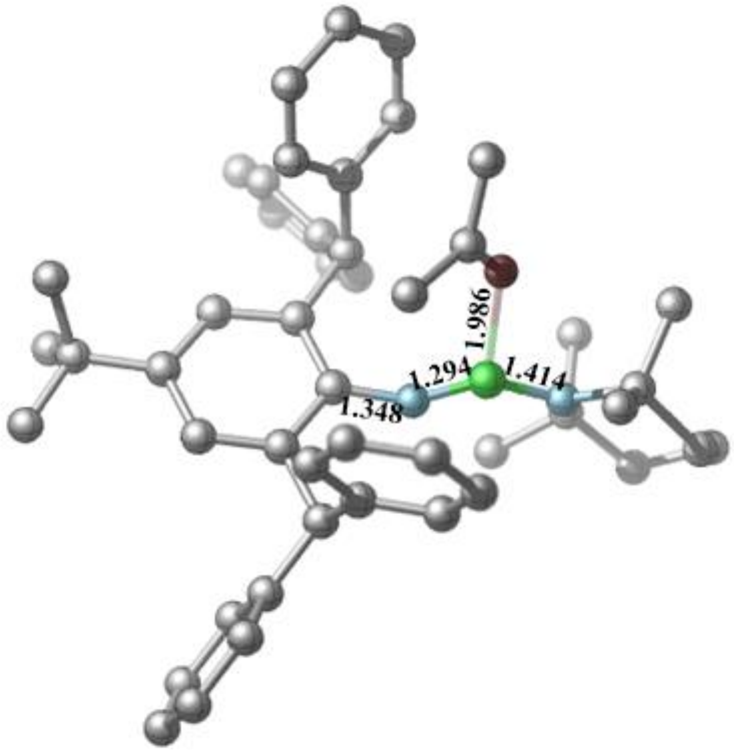

Figure S28. Optimised structure of **TS1$_1$**. Distances in Å and hydrogens omitted for clarity.

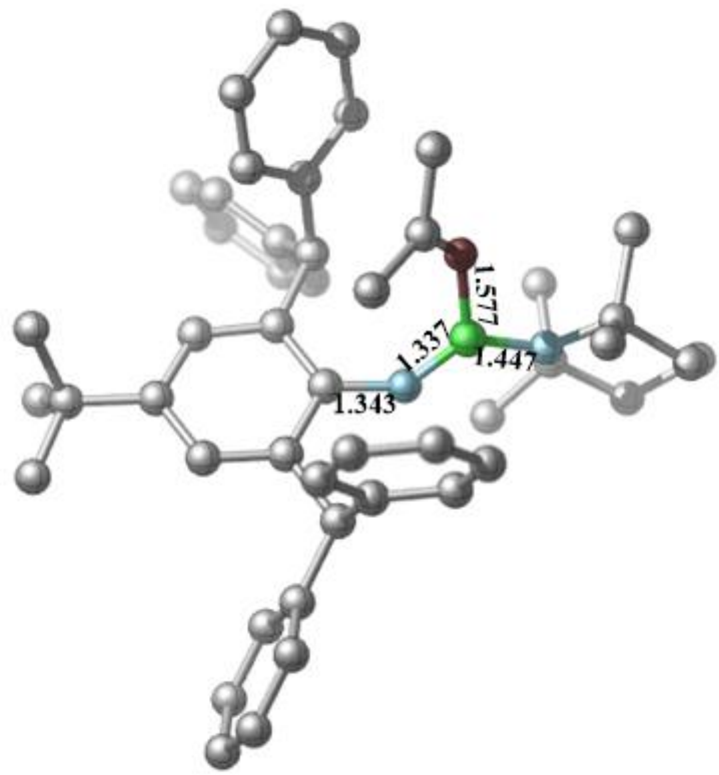

Figure S29. Optimised structure of **I1$_1$**. Distances in Å and hydrogens omitted for clarity.

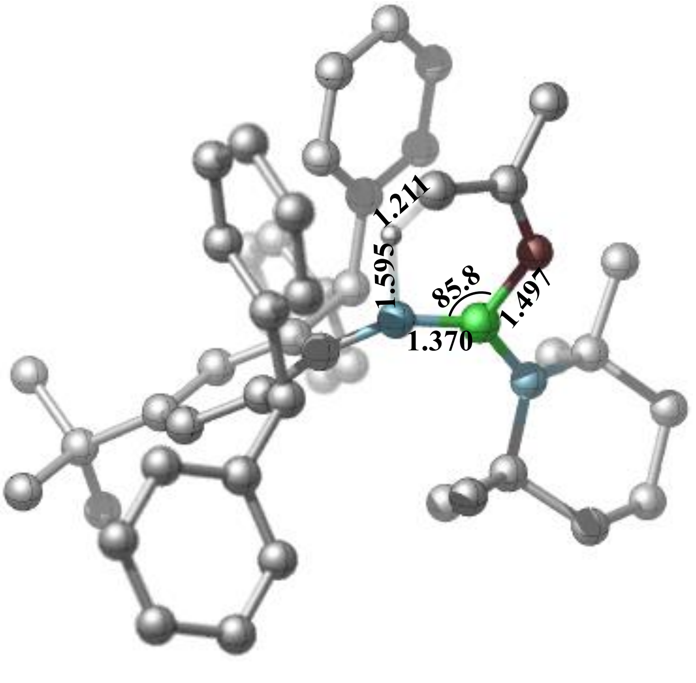

Figure S30. Optimised structure of **TS2$_1$**. Distances in Å and hydrogens omitted for clarity.

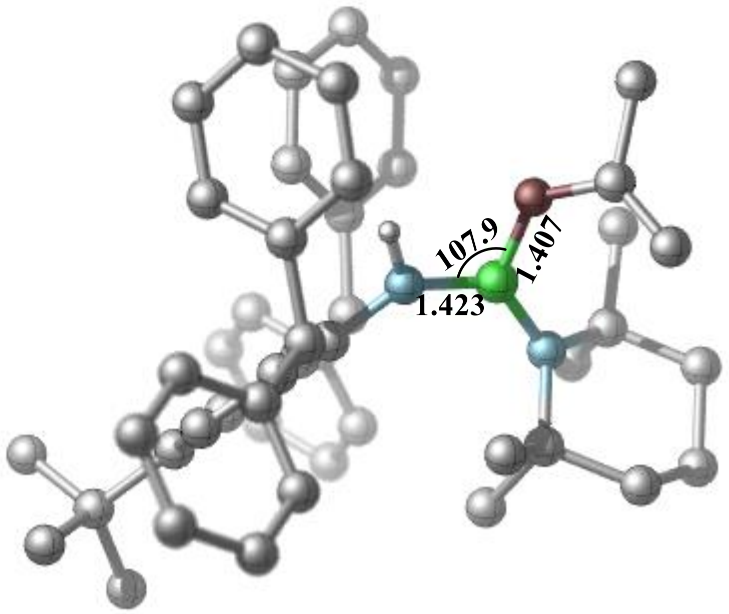

Figure S31. Optimised structure of **I2$_1$**. Distances in Å, angles in ° and hydrogens omitted for clarity.

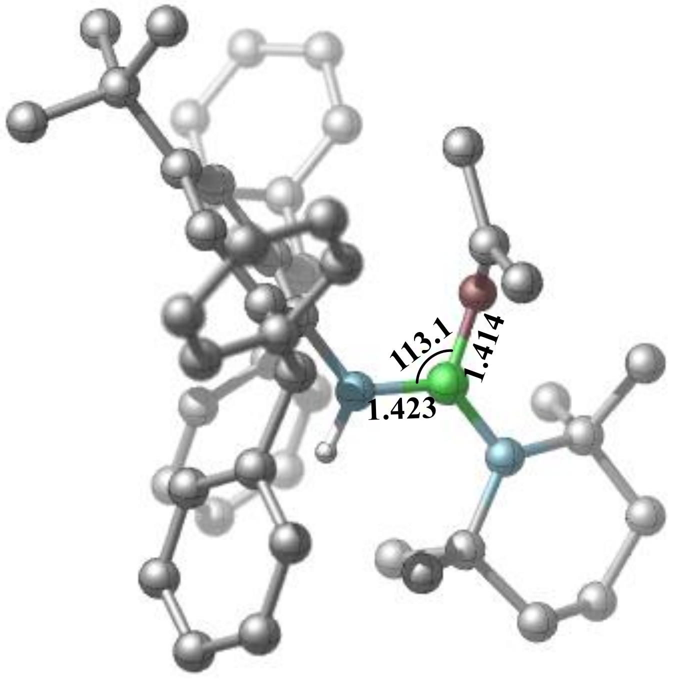

Figure S32. Optimised structure of product **1**. Distances in Å, angles in ° and hydrogens omitted for clarity.

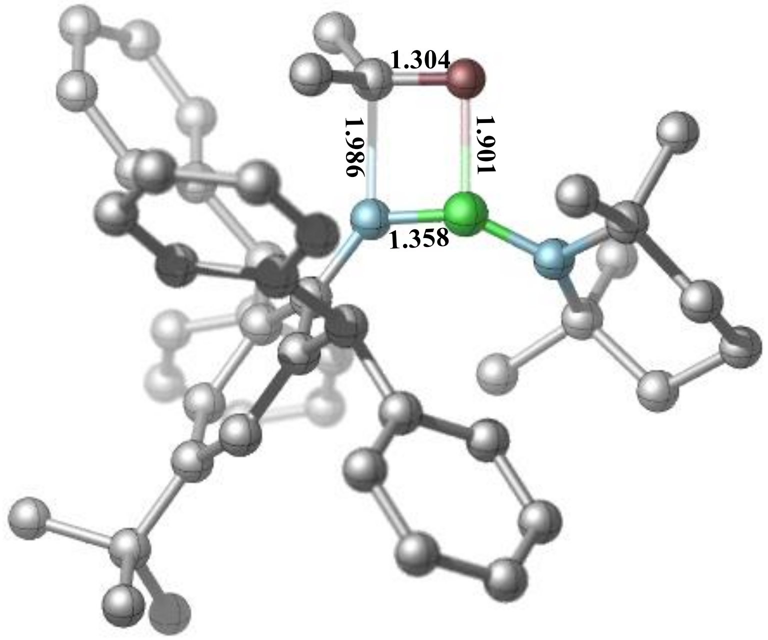

Figure S33. Optimised structure of transition state **TS_{IV-A}**. Distances in Å and hydrogens omitted for clarity.

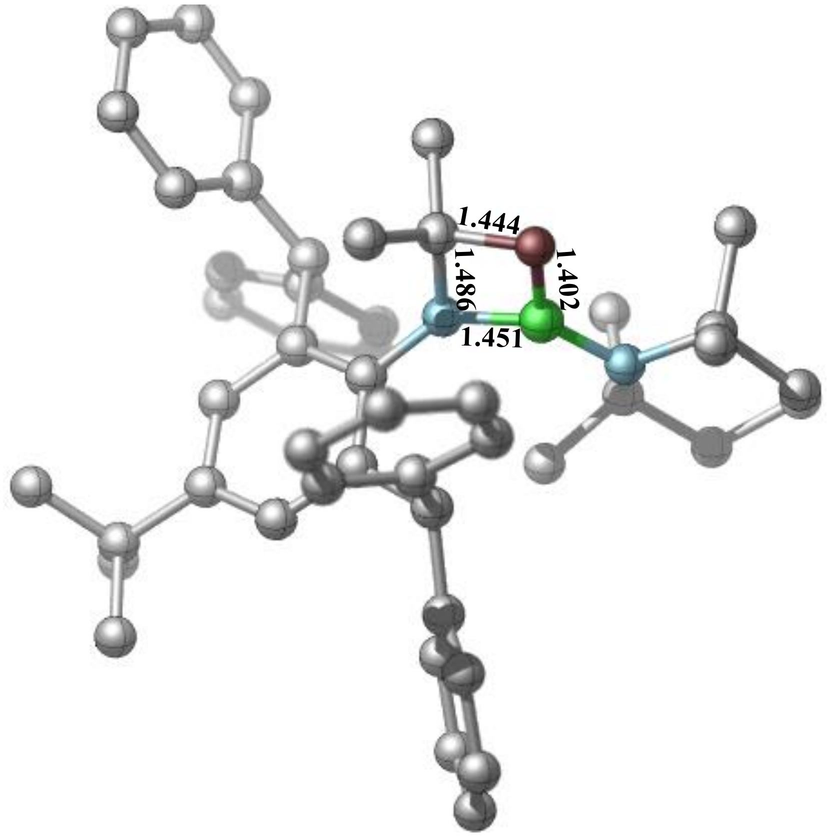

Figure S34. Optimised structure of the putative [2+2] cycloaddition product **A**. Distances in Å and hydrogens omitted for clarity.

For the diboryne II + acetone reaction

The reactivity of diboryne **II** towards acetone has also been investigated computationally (see above for methodology). Scheme S6 shows the calculated energy profile. The results suggest that this reaction follows a very similar mechanistic pattern. That is, from the acetone adduct of **II**, the ketone oxygen attacks one of the boron atoms through transition state **TS1**$_{\mathbf{2}}$ ($\Delta G^{\ddagger}$ = 20.6 kcal·mol^{-1}). The intermediate adduct **I1**$_{\mathbf{2}}$ is highly reactive at 7.5 kcal·mol^{-1} above the starting materials. This then facilitates the proton migration to the second boron through **TS2**$_{\mathbf{2}}$ ($\Delta G^{\ddagger}$ = 14.1 kcal·mol^{-1}) which yields the *cis* isomer of the product, **I2**$_{\mathbf{2}}$. This step is highly exergonic, at –31.4 kcal·mol^{-1} from the starting materials. Finally, the rotation around the B–B bond (through a partial delocalization over the carbene ligands, with a mild energy barrier of 9.7 kcal·mol^{-1}, **TS3**$_{\mathbf{2}}$) leads to the *trans* isomer, **2**, which is 11.8 kcal·mol^{-1} more stable than the *cis* form.

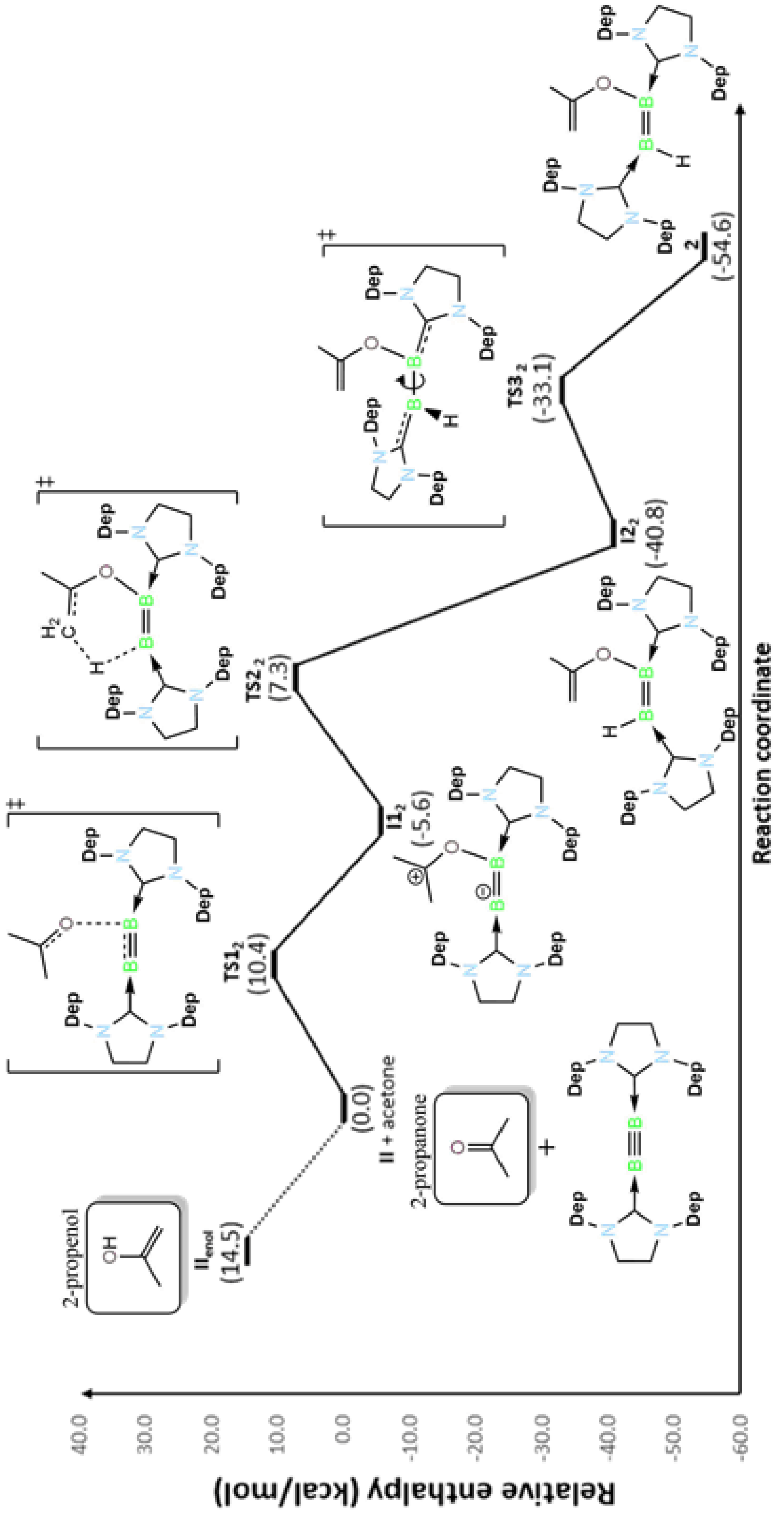

Scheme S5. Enthalpy profile of the proposed reaction mechanism between acetone and diboryne **II**.

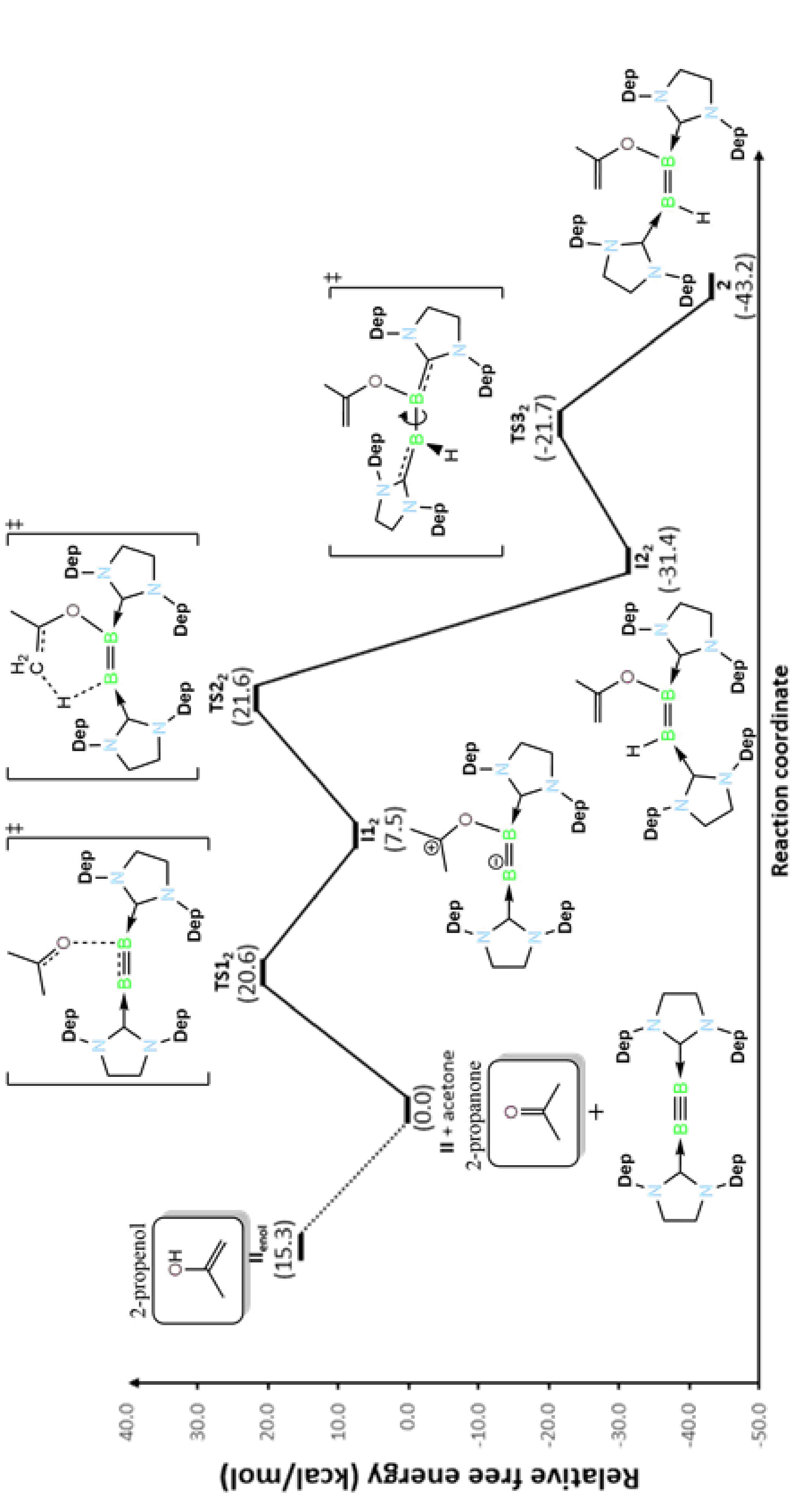

Scheme S6. Free energy profile of the proposed reaction mechanism between acetone and diboryne **II**.

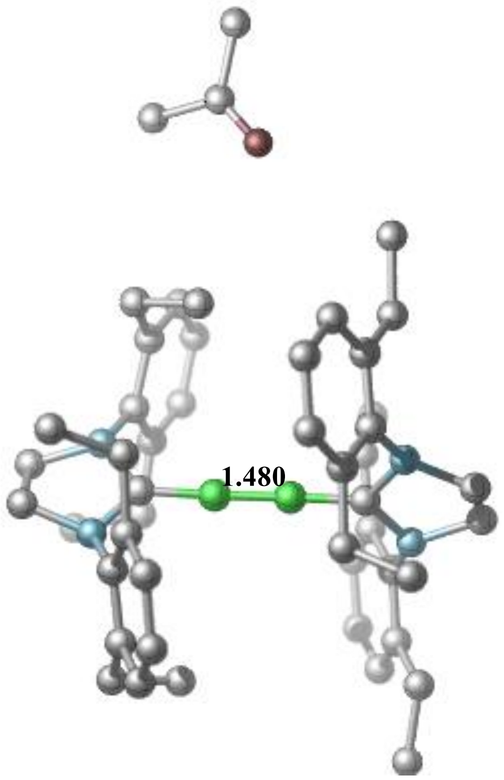

Figure S35. Optimised structure of **II** and acetone. Distances in Å and hydrogens omitted for clarity.

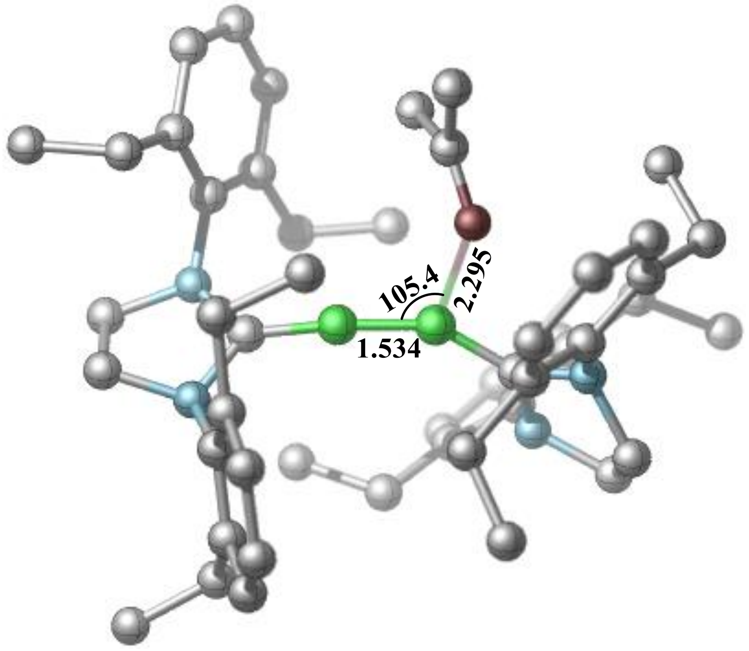

Figure S36. Optimised structure of **TS1$_2$**. Distances in Å, angles in ° and hydrogens omitted for clarity.

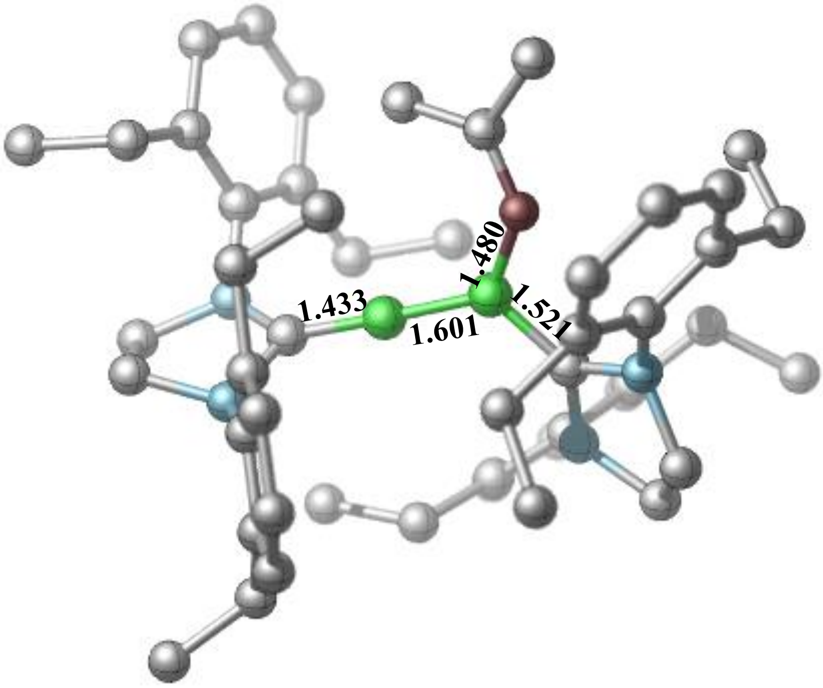

Figure S37. Optimised structure of **I1$_2$**. Distances in Å and hydrogens omitted for clarity.

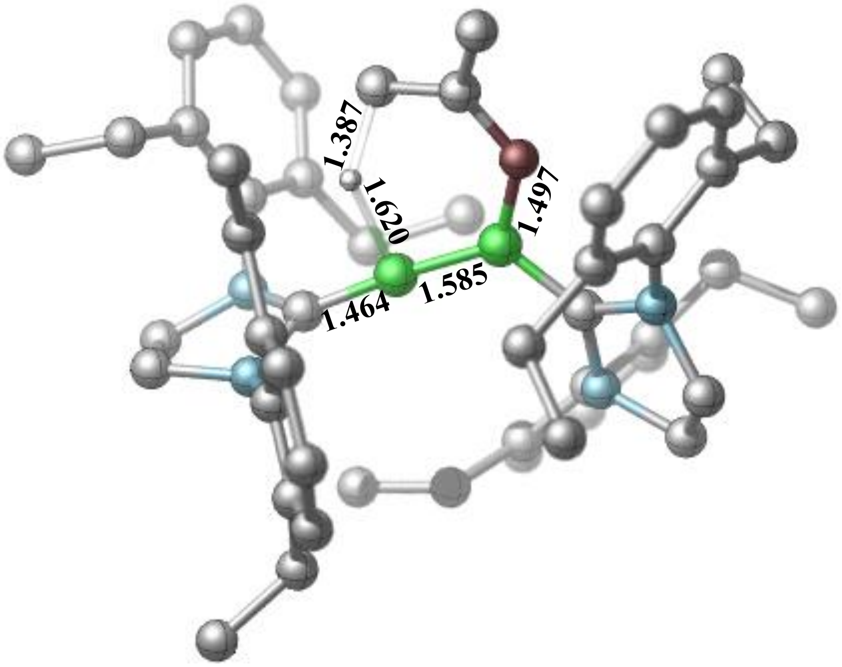

Figure S38. Optimised structure of **TS2$_2$**. Distances in Å and hydrogens omitted for clarity.

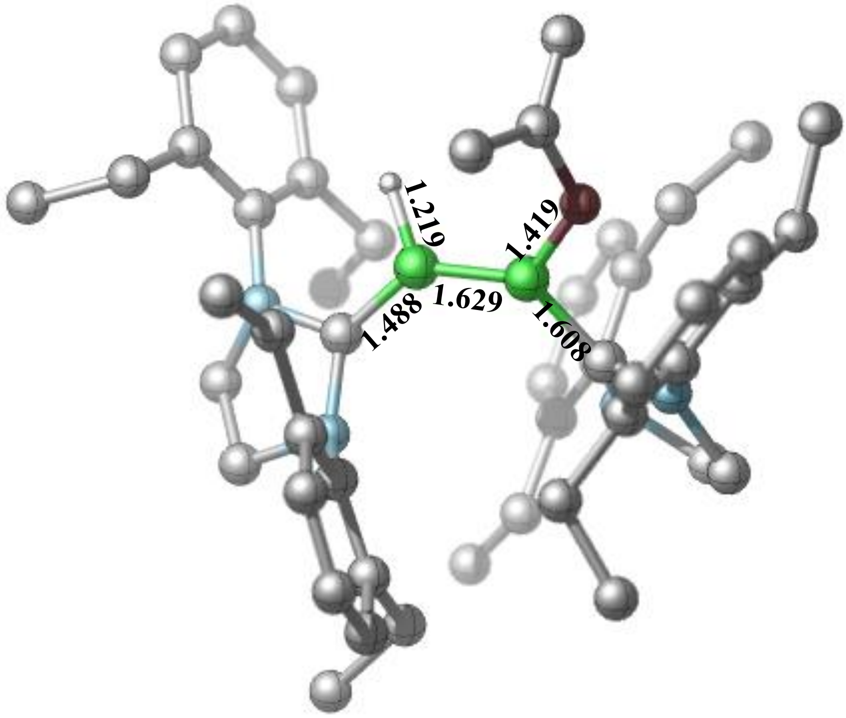

Figure S39. Optimised structure of **I2$_2$**. Distances in Å and hydrogens omitted for clarity.

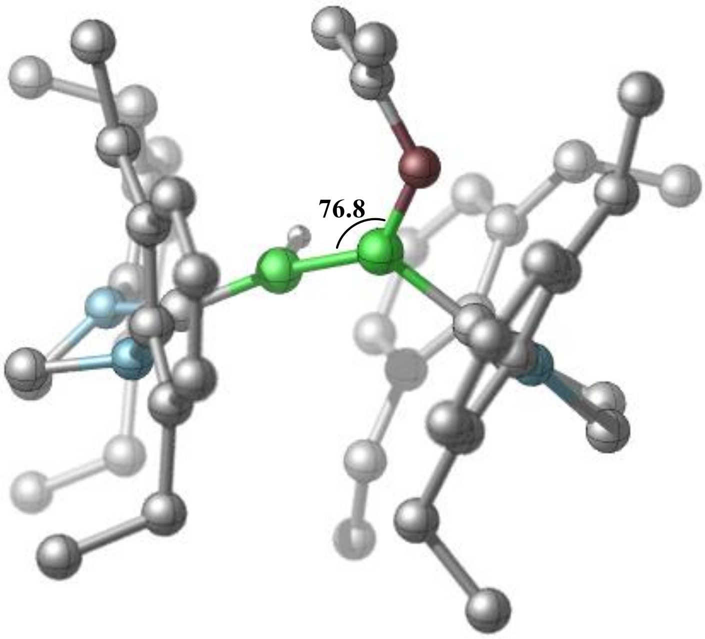

Figure S40. Optimised structure of **TS3$_2$**. Dihedral angle in ° and hydrogens omitted for clarity.

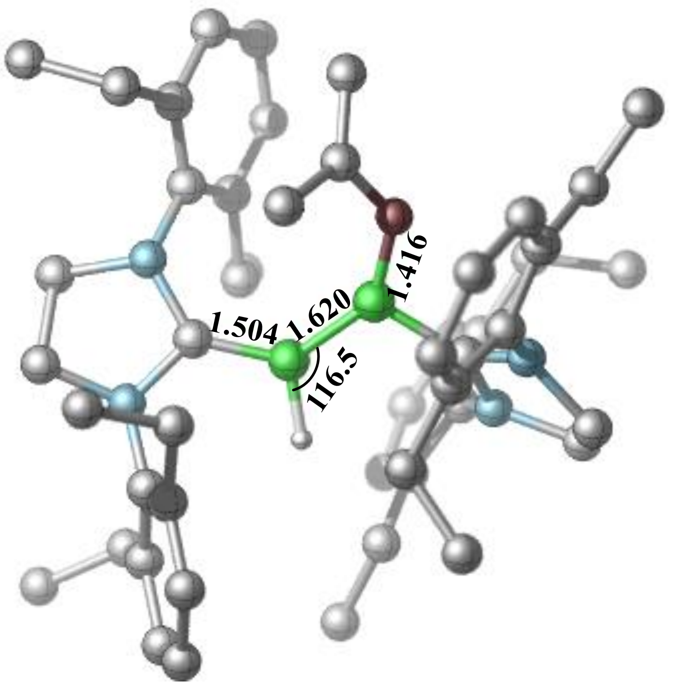

Figure S41. Optimised structure of **2**. Distances in Å, angles in ° and hydrogens omitted for clarity.

For the cumulene III + acetone reaction

The mechanism of the formation of **3** was also investigated by theoretical calculations. Our proposed reaction mechanism is depicted in Scheme S8. The first two steps are identical to those for the diboryne and iminoborane species. Initially, the acetone oxygen attacks one of the boron atoms to produce intermediate **I1$_3$** which is −3.2 kcal·mol^{-1} downhill. This step is achieved through transition state **TS1$_3$** (ΔG‡ = 10.1 kcal·mol^{-1}). Then, the protonation of the other boron atom occurs (**TS2$_3$**; ΔG‡ = 14.9 kcal·mol^{-1}) producing **I2$_3$**, which is exergonic by −7.3 kcal·mol^{-1}. This *cis* form transforms by rotation around the B−B bond into the *trans* isomer, which is 16.9 kcal·mol^{-1} more stable (**I4$_3$**). Very interestingly, this rotation occurs by 1,2-migration of the boron-bound hydrogen to the carbene carbon atom through **TS3$_3$** (ΔG‡ = 12.1 kcal·mol^{-1}) to yield **I3$_3$**, which is only 0.8 kcal·mol^{-1} above the *cis* isomer, **I2$_3$**. This means that **I2$_3$** and **I3$_3$** are in equilibrium each other. Then, the B−C single bond can rotate uphill to **TS4$_3$** to effect the 1,2-hydrogen migration in the opposite direction, from carbon to boron (ΔG‡ = 9.3 kcal·mol^{-1}) that leads to diborene **I4$_3$**, the *trans* isomer. The fact that this rotation step is so different to that of the SIDep diborene **I2$_2$** is due, on the one hand, to the stronger π backdonation afforded by cAAC ligands which allows the stabilisation of coordinatively unsaturated **I3$_3$**, and on the other hand, to the enhanced 1,2-hydrogen migration enabled by cAAC-hydroboron compounds.[19] This is followed by hydrogen migration to the oxygen-bound boron (**TS5$_3$**; ΔG‡ = 19.8 kcal·mol^{-1}) to obtain the borylene-borane **I5$_3$**. The vacancy thus formed on the second boron atom enables the double bond of the enolate to coordinate to it (**TS6$_3$**; ΔG‡ = 11.1 kcal·mol^{-1}), leading to **I6$_3$** which is an exergonic step by −6.6 kcal·mol^{-1}. At this point, the rearrangement of highest energy (**TS7$_3$**; ΔG‡ = 21.2 kcal·mol^{-1}) consists of the alkene-coordinated boron activating the second C−H bond, generating a 4-methyl-1,2-oxadiborole (**I7$_3$**). Finally, although the two cAAC stabilising ligands are in *trans* position, the high steric hindrance forces an intramolecular migration of one boron-bound hydrogen to the adjacent carbene carbon with an energy barrier of only 11.7 kcal·mol^{-1} (**TS8$_3$**), which also allows the remaining boron-bound hydrogen to bridge between the two boron atoms, yielding the product of reaction **3**.

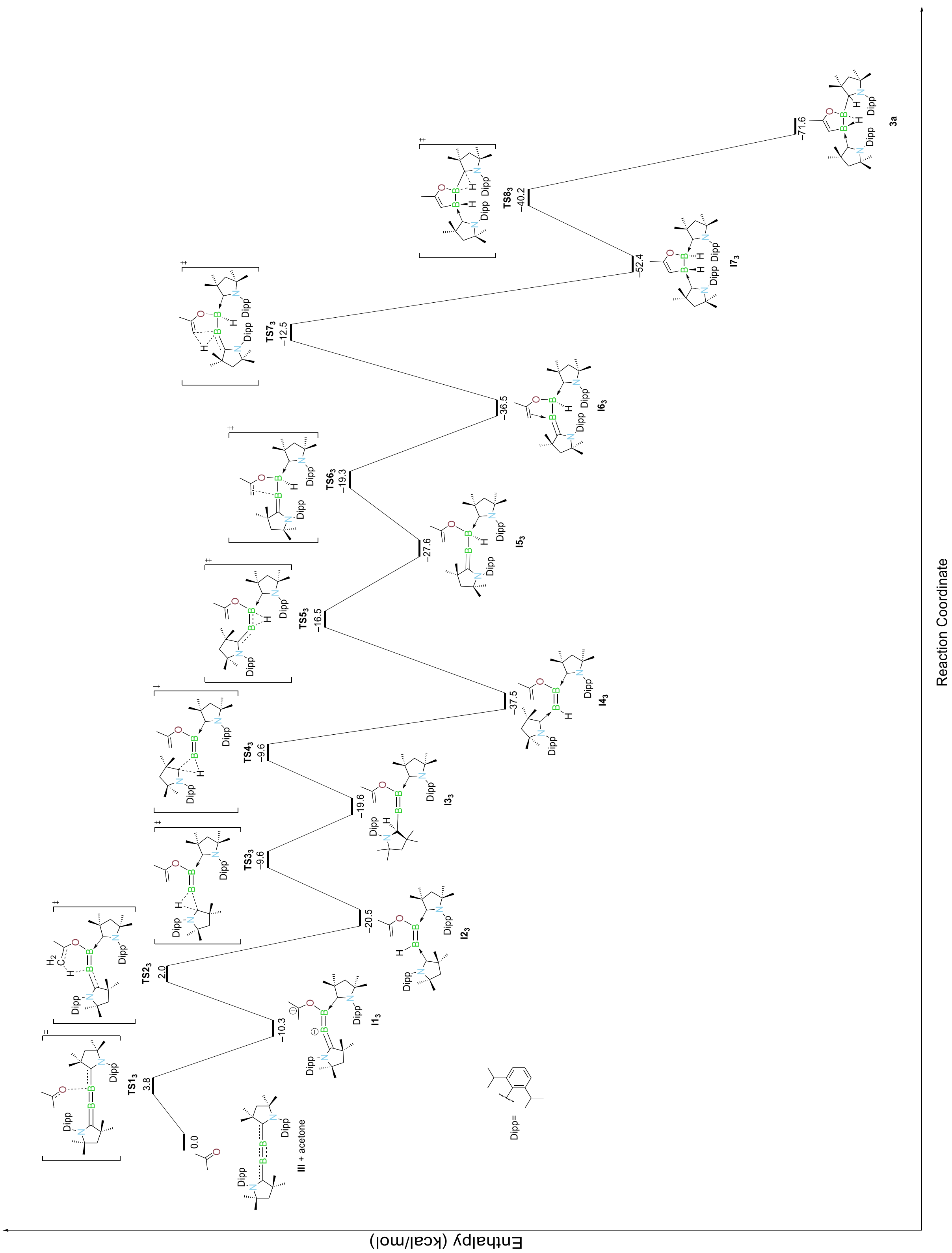

Scheme S7. Enthalpy profile of the proposed reaction mechanism between acetone and **III**.

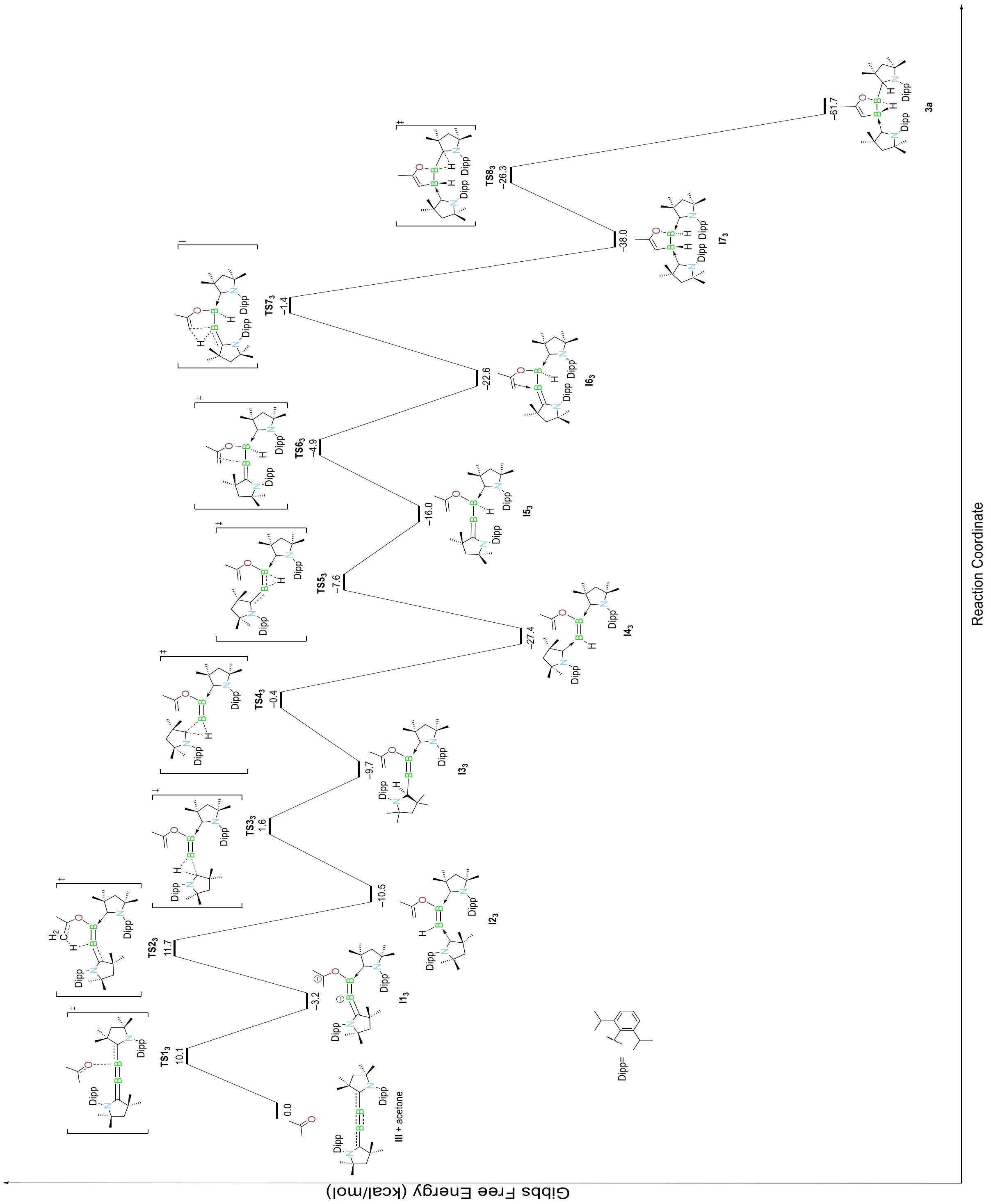

Scheme S8. Free energy profile of the proposed reaction mechanism between acetone and **III**.

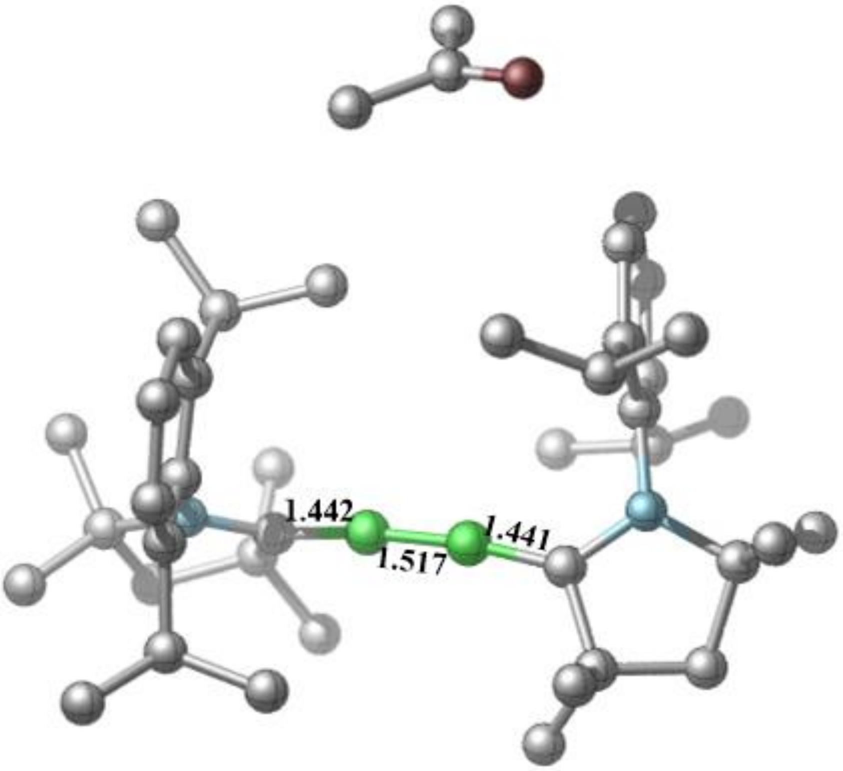

Figure S42. Optimised structure of {**III** + acetone}. Distances in Å and hydrogens omitted for clarity.

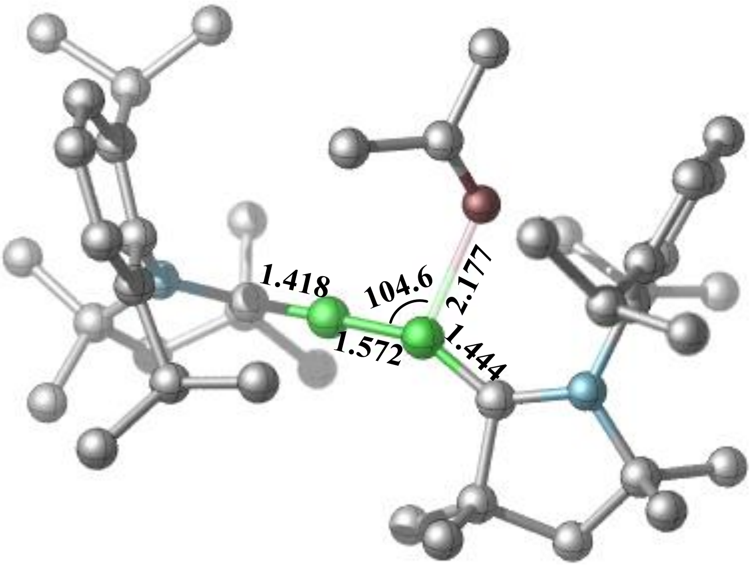

Figure S43. Optimised structure of **TS1$_3$**. Distances in Å, angles in ° and hydrogens omitted for clarity.

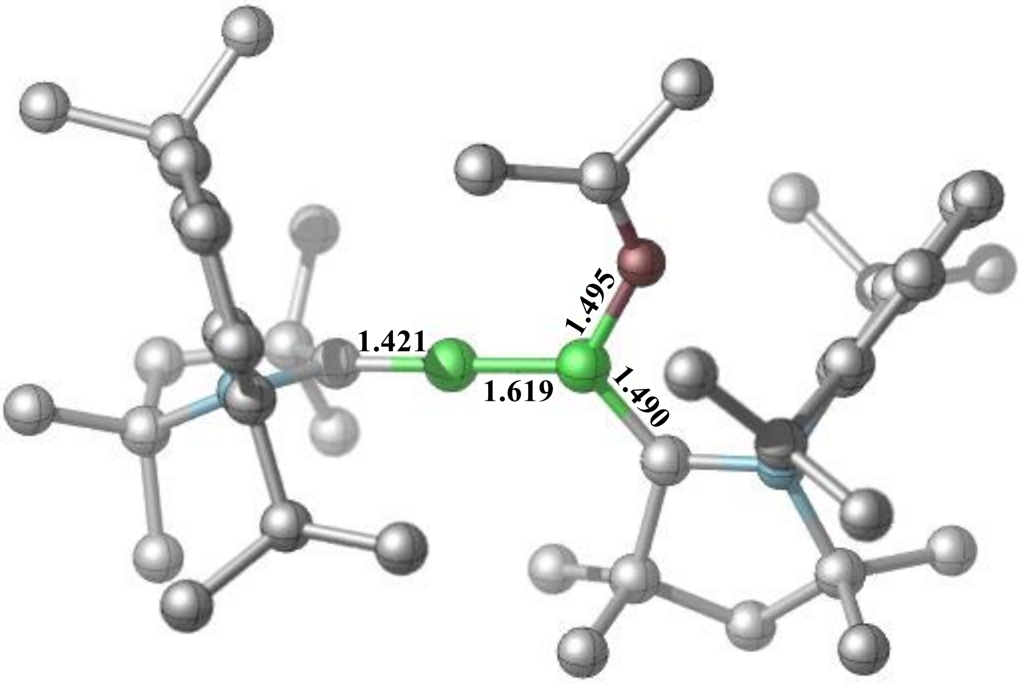

Figure S44. Optimised structure of **11_3**. Distances in Å, hydrogens omitted for clarity.

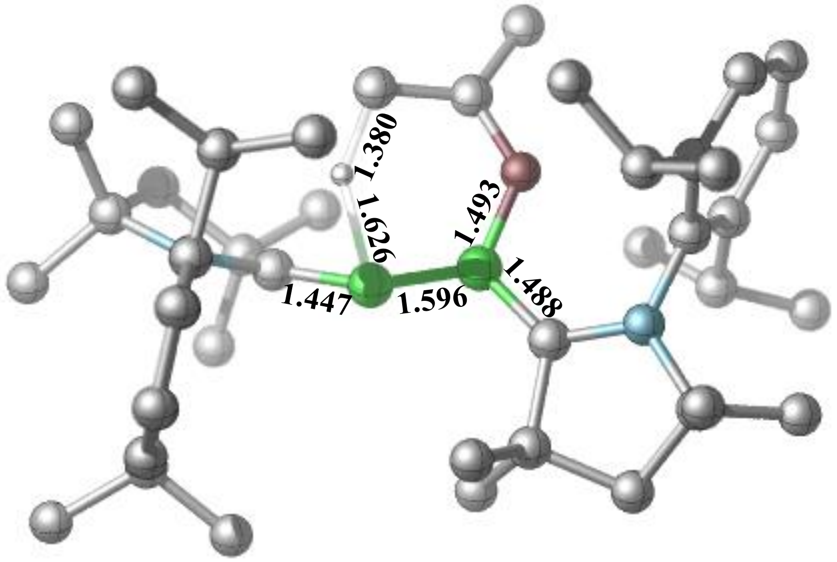

Figure S45. Optimised structure of **$TS2_3$**. Distances in Å, hydrogens omitted for clarity.

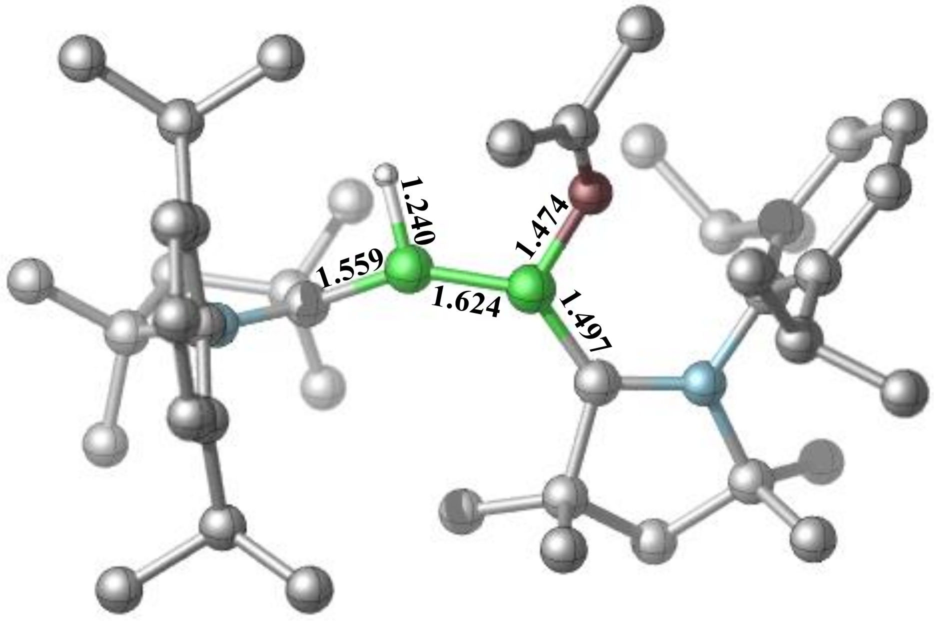

Figure S46. Optimised structure of **I2$_3$**. Distances in Å, hydrogens omitted for clarity.

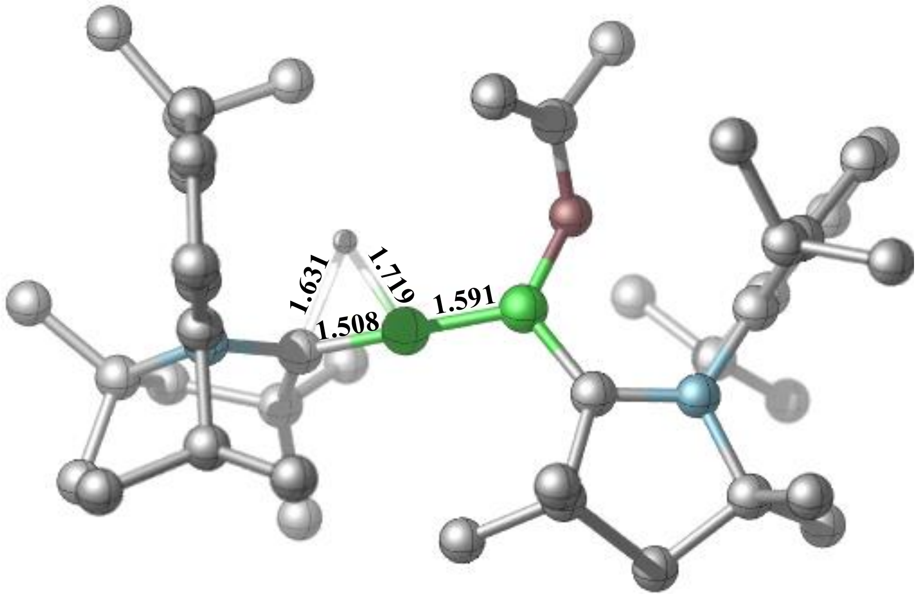

Figure S47. Optimised structure of **TS3$_3$**. Distances in Å, hydrogens omitted for clarity.

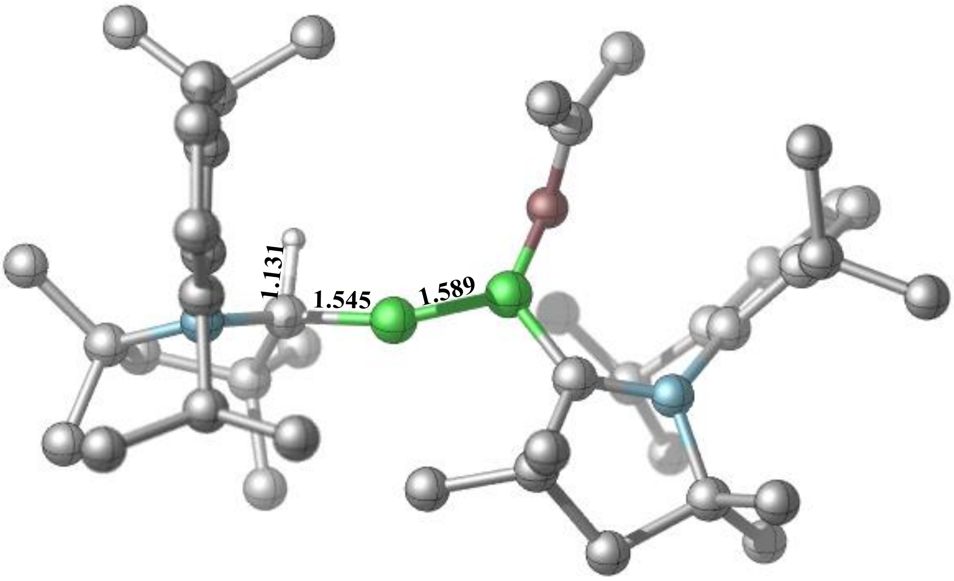

Figure S48. Optimised structure of **I3$_3$**. Distances in Å, hydrogens omitted for clarity.

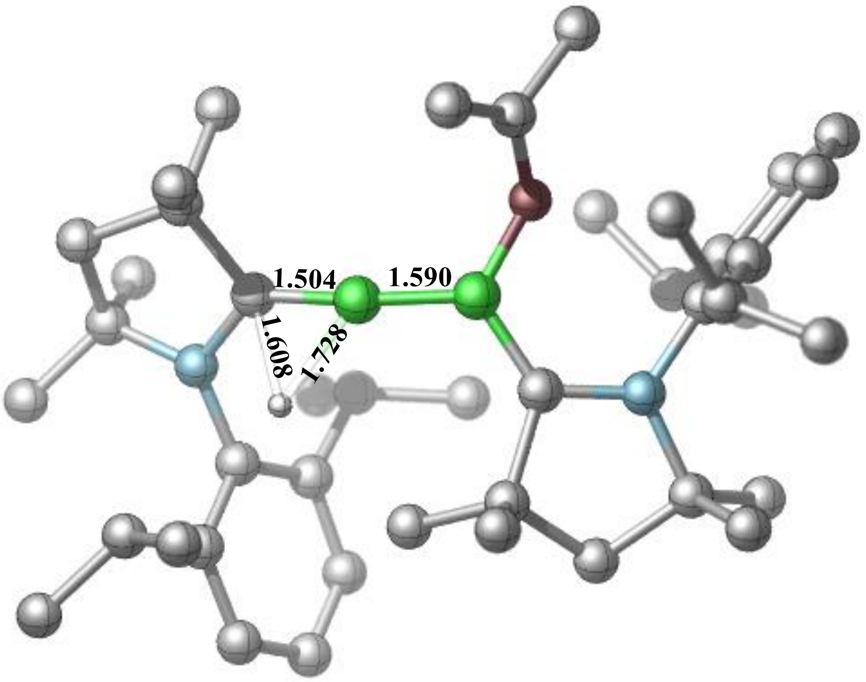

Figure S49. Optimised structure of **TS4$_3$**. Distances in Å, hydrogens omitted for clarity.

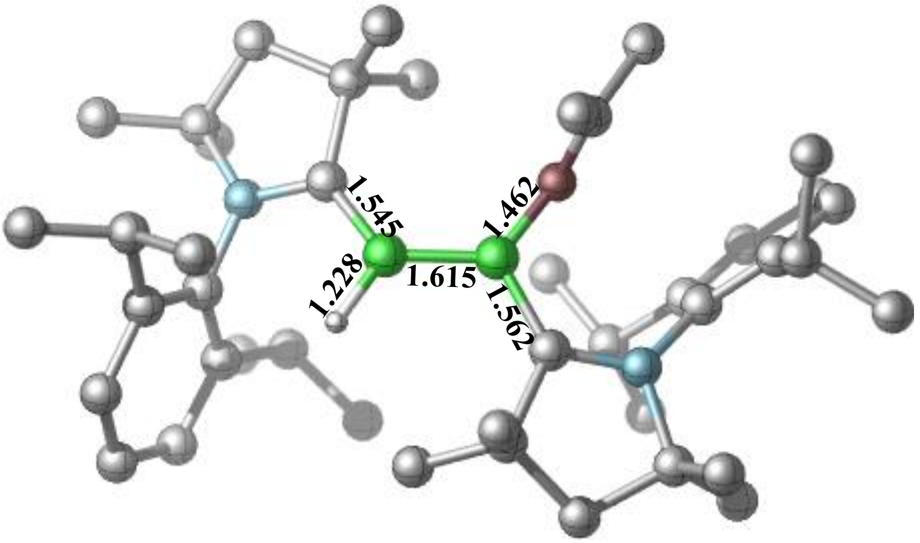

Figure S50. Optimised structure of **I3$_3$**. Distances in Å, hydrogens omitted for clarity.

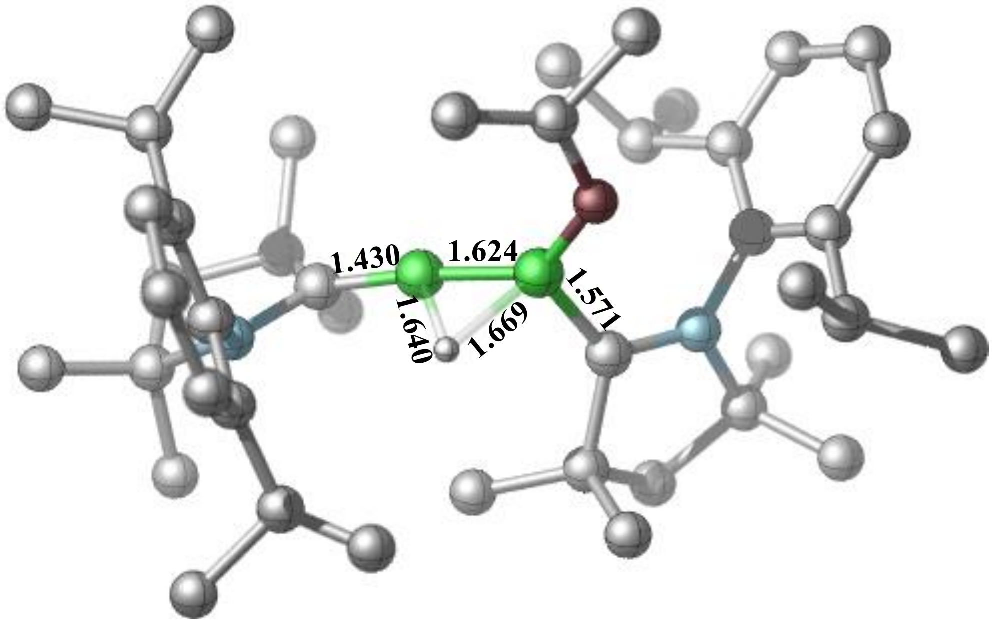

Figure S51. Optimised structure of **TS3$_3$**. Distances in Å, hydrogens omitted for clarity.

.

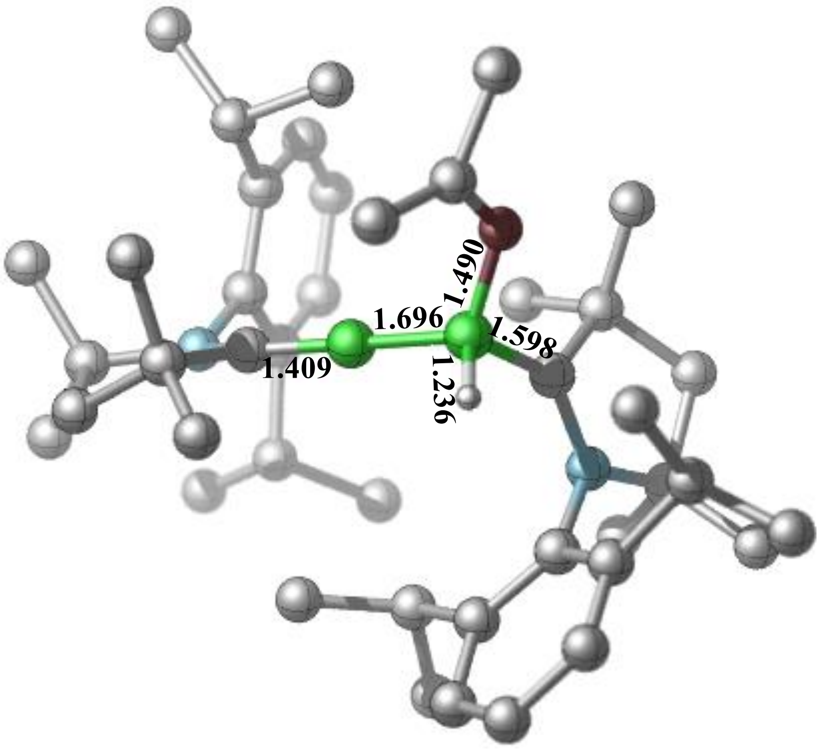

Figure S52. Optimised structure of **I4$_3$**. Distances in Å, hydrogens omitted for clarity.

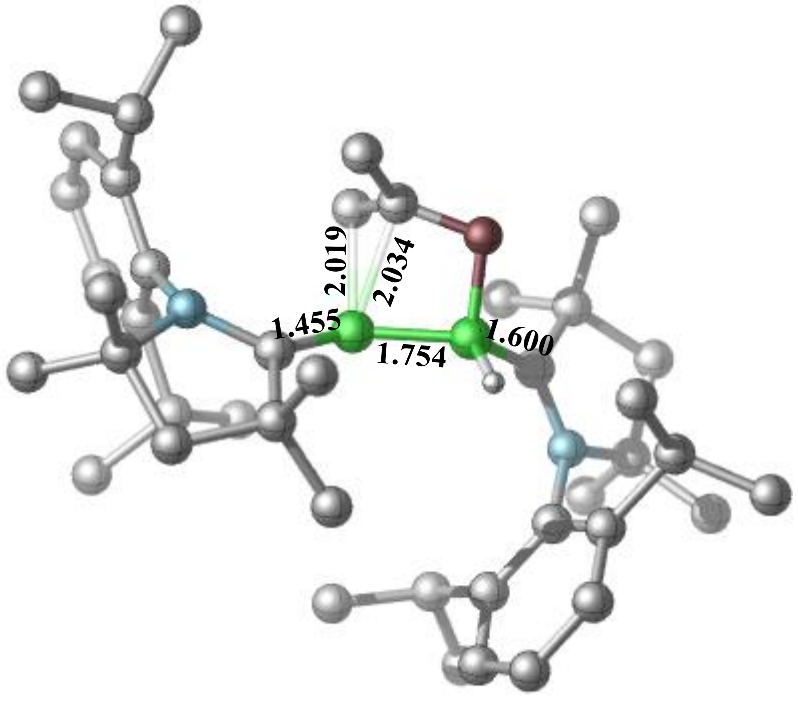

Figure S53. Optimised structure of **TS4$_3$**. Distances in Å, angles in ° and hydrogens omitted for clarity.

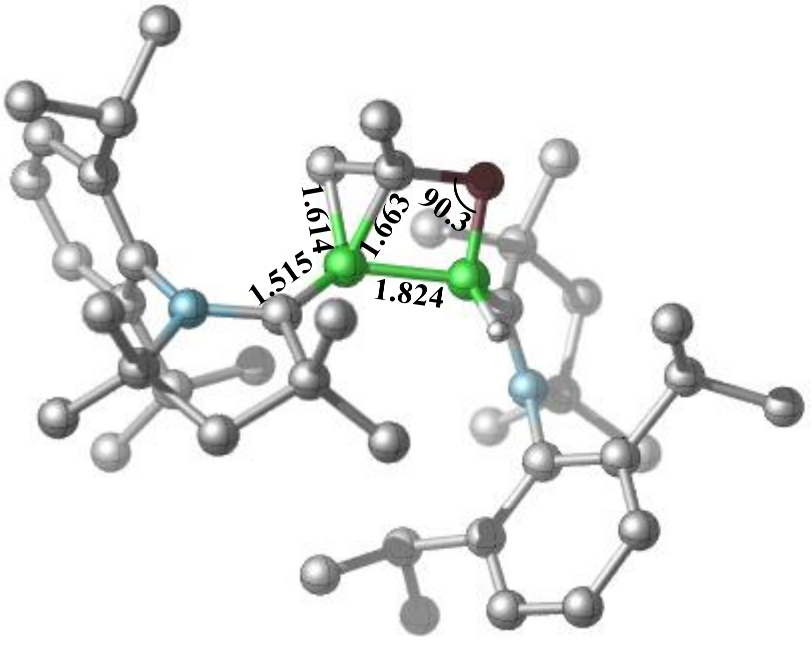

Figure S54. Optimised structure of **I5$_3$**. Distances in Å, angles in ° and hydrogens omitted for clarity.

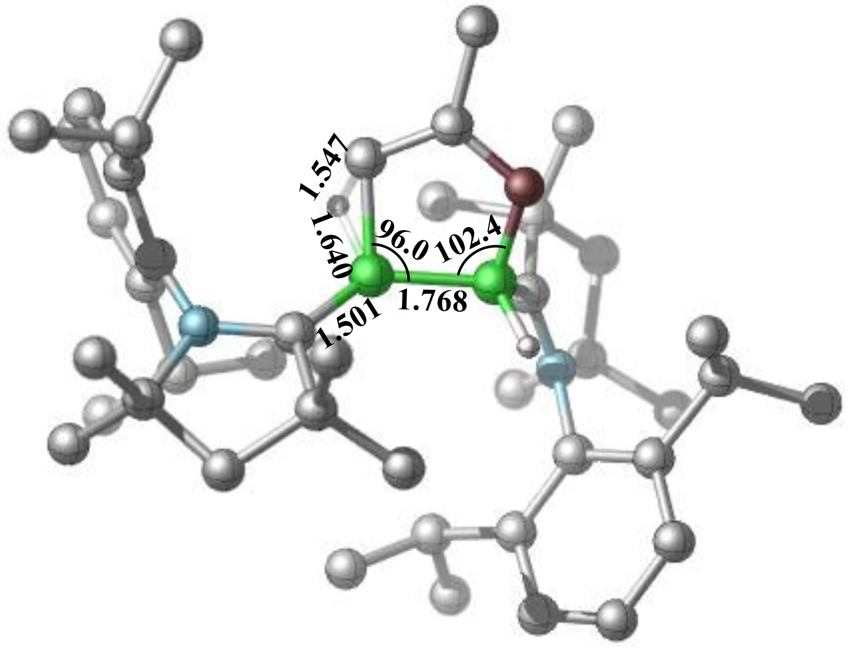

Figure S55. Optimised structure of **TS5$_3$**. Distances in Å, angles in ° and hydrogens omitted for clarity.

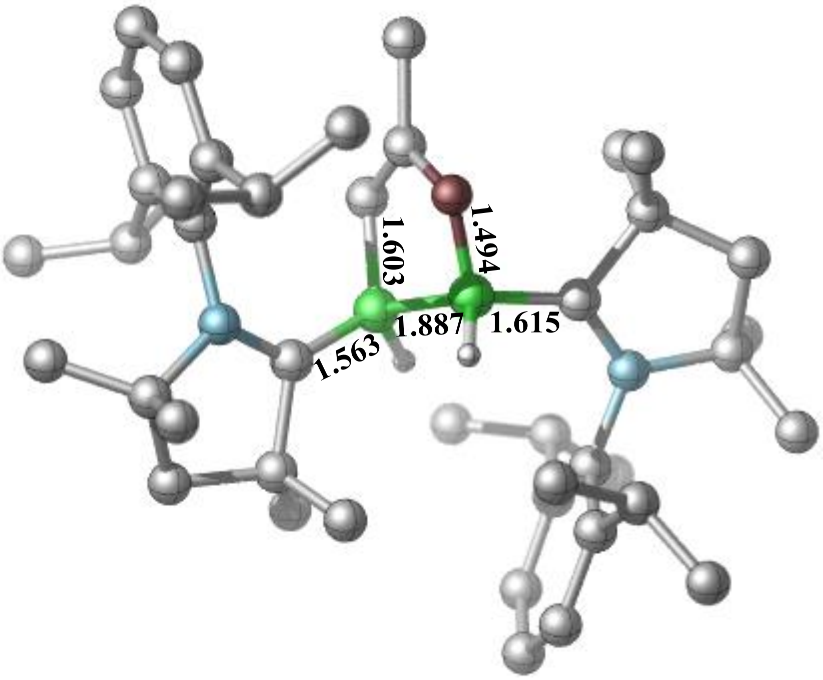

Figure S56. Optimised structure of **I6$_3$**. Distances in Å, hydrogens omitted for clarity.

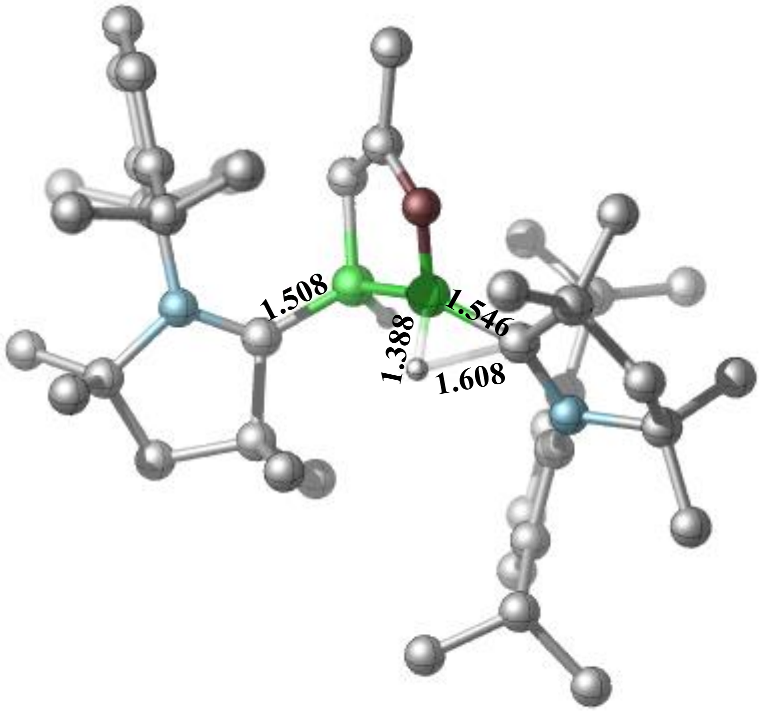

Figure S57. Optimised structure of **TS6$_3$**. Distances in Å, hydrogens omitted for clarity.

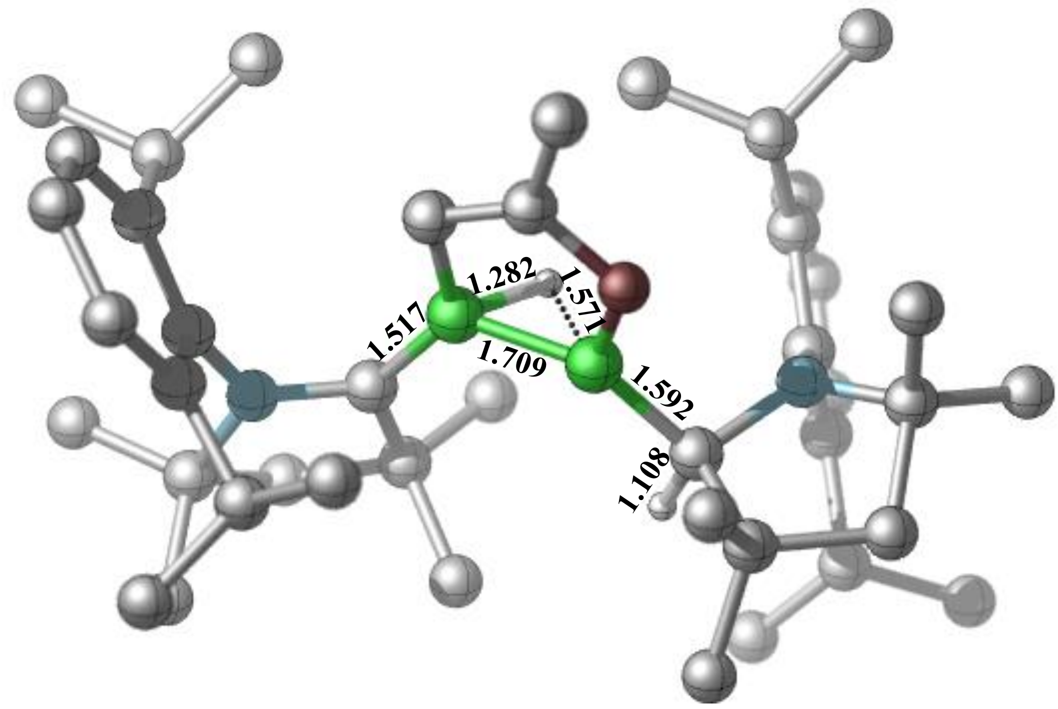

Figure S58. Optimised structure of **3a**. Distances in Å, hydrogens omitted for clarity.

Coordinates of reactants, transition states, intermediates and products

Table S2. Cartesian coordinates (xyz) of the optimised geometries for all the species involved in the {**IV** + acetone} reaction mechanism to yield **1** calculated at the D3-PBE0/6-31G(d) level. Coordinates are given in Å.

IV + acetone

E(scf)= –2070.82175497 a.u.

8	1.776137000	-2.693111000	2.722819000
7	0.367833000	-0.585838000	-1.039266000
7	2.826471000	-1.430991000	-1.539770000
6	0.858188000	-2.038520000	3.185566000
6	0.463195000	-2.158339000	4.637948000
1	0.551041000	-1.175305000	5.119278000
1	1.097488000	-2.886573000	5.150586000
1	-0.590165000	-2.460978000	4.701756000
6	-0.595862000	0.264094000	-0.583585000
6	-1.948135000	-0.130773000	-0.577843000
6	-2.927344000	0.739535000	-0.102999000
1	-3.957812000	0.399789000	-0.123935000
6	-2.621546000	2.020747000	0.347335000
6	-1.279772000	2.404517000	0.319814000
1	-1.006241000	3.408424000	0.634919000
6	-0.268340000	1.560438000	-0.119256000
6	-3.671219000	3.006474000	0.837395000
6	-3.336555000	3.436258000	2.271047000
1	-4.082279000	4.156791000	2.631737000
1	-2.349762000	3.911413000	2.321982000
1	-3.332793000	2.568912000	2.942198000
6	-5.075411000	2.406595000	0.827902000
1	-5.793947000	3.150169000	1.193064000
1	-5.133288000	1.524445000	1.477766000
1	-5.373775000	2.108290000	-0.184667000
6	-3.662835000	4.241611000	-0.072204000
1	-4.397523000	4.976691000	0.282274000
1	-3.918519000	3.960444000	-1.101032000
1	-2.675335000	4.717853000	-0.084078000
6	-2.317449000	-1.537289000	-1.009663000
1	-1.538121000	-1.862891000	-1.712017000
6	-2.211983000	-2.431169000	0.209082000
6	-1.149136000	-3.322810000	0.328813000
1	-0.434301000	-3.392276000	-0.483893000
6	-0.974591000	-4.078601000	1.486606000
1	-0.114964000	-4.736594000	1.576469000
6	-1.886209000	-3.965654000	2.533236000
1	-1.755821000	-4.554631000	3.437522000
6	-2.967316000	-3.088845000	2.415679000
1	-3.684124000	-2.996302000	3.227939000
6	-3.122416000	-2.320668000	1.265594000

$TS1_1$

E(scf)= –2070.81042569 a.u.

ν_{min}= –165.0859 cm^{-1}

8	2.477163000	-0.359288000	1.261591000
7	0.397032000	-0.837753000	-0.413617000
7	2.660115000	-1.997610000	-0.886828000
6	1.949385000	-0.154434000	2.356885000
6	2.738495000	0.492676000	3.445737000
1	2.197201000	1.374621000	3.808985000
1	3.729495000	0.783296000	3.093359000
1	2.817907000	-0.211685000	4.285564000
6	-0.610507000	0.048422000	-0.282161000
6	-1.944425000	-0.436778000	-0.265820000
6	-3.021479000	0.434517000	-0.150748000
1	-4.021656000	0.013101000	-0.142316000
6	-2.843436000	1.817260000	-0.090758000
6	-1.533032000	2.290084000	-0.133112000
1	-1.359350000	3.363073000	-0.107234000
6	-0.422744000	1.453329000	-0.196256000
6	-3.993994000	2.807515000	0.008593000
6	-3.843634000	3.630027000	1.294698000
1	-4.654972000	4.366072000	1.371888000
1	-2.888551000	4.168952000	1.309007000
1	-3.881420000	2.976006000	2.174975000
6	-5.353253000	2.112210000	0.034556000
1	-6.148878000	2.864323000	0.100537000
1	-5.440709000	1.442572000	0.899217000
1	-5.512839000	1.519910000	-0.874772000
6	-3.956592000	3.754276000	-1.197570000
1	-4.767249000	4.491554000	-1.123600000
1	-4.080042000	3.190508000	-2.129945000
1	-3.003223000	4.292846000	-1.250623000
6	-2.134817000	-1.939721000	-0.331873000
1	-1.386739000	-2.305228000	-1.050216000
6	-1.772690000	-2.546451000	1.005536000
6	-0.800428000	-3.541516000	1.093238000
1	-0.306009000	-3.868591000	0.182119000
6	-0.437944000	-4.078592000	2.327748000
1	0.337762000	-4.838559000	2.378421000
6	-1.056188000	-3.628277000	3.492448000
1	-0.774901000	-4.043810000	4.456668000
6	-2.032919000	-2.634689000	3.414081000
1	-2.515940000	-2.273673000	4.318810000
6	-2.381069000	-2.094027000	2.179731000

1	-3.948547000	-1.619400000	1.180598000	1	-3.118368000	-1.297495000	2.111948000
6	-3.646134000	-1.614224000	-1.743285000	6	-3.501772000	-2.346959000	-0.849824000
6	-4.549025000	-2.660917000	-1.548290000	6	-4.388094000	-3.152377000	-0.135570000
1	-4.324476000	-3.429747000	-0.814817000	1	-4.106746000	-3.520162000	0.846586000
6	-5.733971000	-2.727129000	-2.281401000	6	-5.630582000	-3.493790000	-0.672862000
1	-6.424364000	-3.549383000	-2.110513000	1	-6.307155000	-4.123268000	-0.100054000
6	-6.033599000	-1.748064000	-3.223875000	6	-6.003688000	-3.033709000	-1.931173000
1	-6.959096000	-1.797456000	-3.791685000	1	-6.972184000	-3.298341000	-2.347628000
6	-5.134465000	-0.702184000	-3.432742000	6	-5.122352000	-2.228863000	-2.655605000
1	-5.354403000	0.068049000	-4.167882000	1	-5.400872000	-1.863759000	-3.641077000
6	-3.954514000	-0.639032000	-2.700080000	6	-3.886140000	-1.892185000	-2.118180000
1	-3.260669000	0.183431000	-2.854244000	1	-3.203140000	-1.251894000	-2.671989000
6	1.176263000	2.010260000	-0.025603000	6	0.960397000	2.067569000	-0.163921000
1	1.756258000	1.397551000	-0.725820000	1	1.680601000	1.269501000	-0.377894000
6	1.687850000	1.680546000	1.362464000	6	1.349495000	2.661337000	1.182710000
6	1.187984000	2.345562000	2.487245000	6	0.449133000	2.889427000	2.224348000
1	0.457672000	3.138799000	2.353422000	1	-0.584448000	2.574117000	2.116286000
6	1.603842000	1.986214000	3.764843000	6	0.859386000	3.534039000	3.394110000
1	1.203834000	2.509963000	4.629438000	1	0.138264000	3.711893000	4.188085000
6	2.524379000	0.949481000	3.938523000	6	2.178706000	3.952573000	3.541835000
1	2.841456000	0.661199000	4.937425000	1	2.496123000	4.459264000	4.449396000
6	3.031773000	0.288574000	2.824115000	6	3.092728000	3.709118000	2.514271000
1	3.730015000	-0.534811000	2.943700000	1	4.128354000	4.022098000	2.620207000
6	2.617377000	0.659943000	1.546148000	6	2.678948000	3.070151000	1.352403000
1	2.999149000	0.128332000	0.680888000	1	3.384334000	2.894737000	0.543186000
6	1.392305000	3.453156000	-0.449796000	6	1.117994000	3.112600000	-1.257272000
6	0.701597000	3.953726000	-1.559948000	6	1.561282000	2.726404000	-2.523381000
1	-0.032178000	3.320425000	-2.051705000	1	1.831419000	1.688533000	-2.696480000
6	0.929418000	5.246885000	-2.018758000	6	1.663196000	3.653596000	-3.557119000
1	0.379489000	5.617612000	-2.880141000	1	2.013250000	3.333934000	-4.535470000
6	1.855758000	6.066127000	-1.373525000	6	1.322044000	4.986734000	-3.336986000
1	2.031206000	7.078911000	-1.726781000	1	1.400602000	5.712384000	-4.142270000
6	2.551304000	5.575963000	-0.272001000	6	0.889380000	5.384605000	-2.073317000
1	3.276288000	6.204526000	0.239025000	1	0.631797000	6.424356000	-1.887611000
6	2.322350000	4.278400000	0.185039000	6	0.795357000	4.455228000	-1.040596000
1	2.868020000	3.903542000	1.046087000	1	0.483221000	4.772287000	-0.048347000
6	3.638735000	-0.564917000	-2.437399000	6	3.010105000	-1.617414000	-2.279829000
6	4.511067000	0.419176000	-1.653288000	6	3.982121000	-0.432121000	-2.286215000
1	5.035728000	1.075895000	-2.357984000	1	4.129778000	-0.065996000	-3.310523000
1	5.261503000	-0.086550000	-1.041387000	1	4.959465000	-0.699083000	-1.874818000
1	3.897065000	1.048188000	-1.000101000	1	3.568882000	0.384335000	-1.680833000
6	2.702485000	0.248336000	-3.332274000	6	1.756152000	-1.210721000	-3.059764000
1	3.304936000	0.790911000	-4.069950000	1	2.034897000	-1.065479000	-4.110947000
1	2.123489000	0.987485000	-2.765911000	1	1.307488000	-0.288570000	-2.684350000
1	2.005569000	-0.412041000	-3.860505000	1	0.997806000	-1.997468000	-2.993081000
6	4.513035000	-1.437399000	-3.344742000	6	3.606005000	-2.810920000	-3.038577000
1	3.863233000	-1.934179000	-4.079280000	1	2.790756000	-3.515011000	-3.258393000
1	5.190487000	-0.774529000	-3.897105000	1	3.986703000	-2.440217000	-3.998748000
6	5.277833000	-2.502559000	-2.575126000	6	4.679890000	-3.552547000	-2.264379000
1	5.944875000	-2.039051000	-1.836366000	1	5.544331000	-2.900989000	-2.081666000
1	5.907681000	-3.085008000	-3.257814000	1	5.043698000	-4.408264000	-2.846148000
6	4.266329000	-3.403544000	-1.885258000	6	4.068850000	-4.022082000	-0.955233000
1	4.757186000	-4.222046000	-1.344995000	1	4.787818000	-4.597955000	-0.358337000

1	3.617481000	-3.852794000	-2.650976000
6	3.388103000	-2.644071000	-0.888450000
6	2.227230000	-3.546183000	-0.480175000
1	2.627089000	-4.483816000	-0.077204000
1	1.599672000	-3.775909000	-1.350366000
1	1.626028000	-3.084533000	0.311346000
6	4.174112000	-2.308118000	0.381883000
1	4.586284000	-3.234290000	0.800379000
1	3.500455000	-1.886604000	1.132433000
1	5.003660000	-1.620603000	0.198168000
5	1.551883000	-1.000877000	-1.221630000
6	0.053174000	-1.067938000	2.379226000
1	0.164028000	-0.061144000	2.800144000
1	0.387052000	-1.068189000	1.343808000
1	-1.008696000	-1.335363000	2.424037000

I1$_1$

E(scf)= −2070.81402143 a.u.

8	2.304229000	-0.554779000	1.101867000
7	0.352247000	-0.874133000	-0.458193000
7	2.589736000	-1.977233000	-0.898198000
6	1.818972000	-0.349400000	2.238950000
6	2.741277000	0.132738000	3.292693000
1	2.372489000	1.094401000	3.672228000
1	3.757183000	0.249558000	2.912788000
1	2.716428000	-0.577689000	4.130339000
6	-0.604224000	0.046674000	-0.252630000
6	-1.960913000	-0.388323000	-0.205480000
6	-2.999450000	0.508829000	0.007827000
1	-4.011485000	0.117326000	0.042737000
6	-2.777420000	1.882699000	0.120976000
6	-1.457426000	2.317316000	0.016093000
1	-1.248845000	3.384505000	0.054163000
6	-0.378616000	1.450362000	-0.135630000
6	-3.890862000	2.899135000	0.321700000
6	-3.637132000	3.681330000	1.616630000
1	-4.422362000	4.434257000	1.767569000
1	-2.669943000	4.197524000	1.581360000
1	-3.633526000	3.003267000	2.479397000
6	-5.264196000	2.238383000	0.417751000
1	-6.032522000	3.007199000	0.565016000
1	-5.309803000	1.539894000	1.262711000
1	-5.502044000	1.683519000	-0.498070000
6	-3.905645000	3.879172000	-0.858215000
1	-4.688931000	4.635541000	-0.713203000
1	-4.103427000	3.345099000	-1.795261000
1	-2.942671000	4.393560000	-0.958135000
6	-2.210141000	-1.875450000	-0.369649000
1	-1.476320000	-2.211881000	-1.117404000
6	-1.855749000	-2.582576000	0.918869000
6	-0.839110000	-3.537553000	0.950115000

1	3.220200000	-4.682994000	-1.183943000
6	3.562665000	-2.857623000	-0.093502000
6	2.751584000	-3.476156000	1.046049000
1	3.382665000	-4.177197000	1.605704000
1	1.894712000	-4.020012000	0.636111000
1	2.374664000	-2.725579000	1.746873000
6	4.776392000	-2.121631000	0.496141000
1	5.292067000	-2.787370000	1.200477000
1	4.463393000	-1.218016000	1.020606000
1	5.498326000	-1.841482000	-0.275605000
5	1.619121000	-1.246301000	-0.294172000
6	0.544907000	-0.554997000	2.653136000
1	-0.126337000	0.158499000	2.157386000
1	0.323890000	-1.532738000	2.217049000
1	0.340015000	-0.549231000	3.726808000

TS2$_1$

E(scf)= −2070.80727892 a.u.

ν_{min}= −669.6289 cm^{-1}

8	-1.506163000	-3.040797000	0.233442000
7	-0.036087000	-1.146179000	0.111086000
7	-1.848836000	-1.358598000	1.965599000
6	-1.132418000	-3.546096000	-0.890922000
6	-2.217886000	-4.150980000	-1.701605000
1	-2.791041000	-3.331497000	-2.157646000
1	-2.902702000	-4.712407000	-1.052234000
1	-1.824322000	-4.790730000	-2.493875000
6	0.465168000	0.121282000	-0.015747000
6	1.853359000	0.339615000	0.126268000
6	2.393280000	1.612795000	-0.040200000
1	3.462016000	1.737908000	0.100757000
6	1.601069000	2.703201000	-0.383279000
6	0.237267000	2.469967000	-0.584667000
1	-0.407542000	3.301205000	-0.871719000
6	-0.336682000	1.218435000	-0.416550000
6	2.138987000	4.116901000	-0.521298000
6	1.798740000	4.675123000	-1.911054000
1	2.170683000	5.703719000	-2.008532000
1	0.713833000	4.690860000	-2.086047000
1	2.264311000	4.062383000	-2.696578000
6	3.659244000	4.176341000	-0.339337000
1	3.998048000	5.214533000	-0.457717000
1	4.172288000	3.557084000	-1.083395000
1	3.954640000	3.827152000	0.660155000
6	1.484613000	5.008447000	0.549435000
1	1.842734000	6.040646000	0.454213000
1	1.727541000	4.638685000	1.557181000
1	0.394387000	5.008214000	0.439332000
6	2.739745000	-0.874589000	0.336669000
1	2.194272000	-1.553795000	0.999987000
6	2.938306000	-1.642990000	-0.959714000
6	3.492323000	-2.926162000	-0.902168000

1	-0.312824000	-3.767792000	0.027431000	1	3.802267000	-3.314974000	0.063798000
6	-0.464330000	-4.139108000	2.151462000	6	3.637060000	-3.693313000	-2.052571000
1	0.342751000	-4.867257000	2.161275000	1	4.062845000	-4.691533000	-1.986512000
6	-1.108663000	-3.792417000	3.337405000	6	3.238724000	-3.182893000	-3.289493000
1	-0.815919000	-4.258093000	4.275047000	1	3.345988000	-3.786308000	-4.189873000
6	-2.128968000	-2.840126000	3.314976000	6	2.709150000	-1.897468000	-3.360309000
1	-2.633933000	-2.561082000	4.236634000	1	2.402520000	-1.488382000	-4.319602000
6	-2.493767000	-2.237724000	2.114355000	6	2.564481000	-1.130806000	-2.203415000
1	-3.264850000	-1.471148000	2.091305000	1	2.153893000	-0.126415000	-2.260710000
6	-3.593851000	-2.197323000	-0.903044000	6	4.043634000	-0.524172000	1.019079000
6	-4.472541000	-3.085236000	-0.282863000	6	5.218769000	-0.289081000	0.301859000
1	-4.176395000	-3.577673000	0.638594000	1	5.215366000	-0.418937000	-0.781784000
6	-5.726997000	-3.349761000	-0.835897000	6	6.383962000	0.104172000	0.953678000
1	-6.396858000	-4.045014000	-0.335800000	1	7.291569000	0.281138000	0.381946000
6	-6.120437000	-2.729597000	-2.016856000	6	6.386995000	0.264384000	2.336770000
1	-7.098474000	-2.933753000	-2.444970000	1	7.300827000	0.568723000	2.849324000
6	-5.246657000	-1.842302000	-2.648154000	6	5.221775000	0.029403000	3.065540000
1	-5.540384000	-1.352275000	-3.573201000	1	5.215843000	0.148559000	4.145717000
6	-3.998622000	-1.582798000	-2.095669000	6	4.060212000	-0.360143000	2.406402000
1	-3.321461000	-0.879596000	-2.574688000	1	3.140953000	-0.528907000	2.963519000
6	1.019454000	2.025066000	-0.198637000	6	-1.811272000	0.967618000	-0.651375000
1	1.684716000	1.228882000	-0.554412000	1	-2.151628000	0.439167000	0.248068000
6	1.568701000	2.500720000	1.136536000	6	-2.100940000	0.040953000	-1.824534000
6	0.761795000	2.819913000	2.230150000	6	-1.112667000	-0.438992000	-2.679424000
1	-0.308475000	2.644399000	2.169485000	1	-0.075179000	-0.180435000	-2.493302000
6	1.314545000	3.376430000	3.386278000	6	-1.439654000	-1.259683000	-3.756089000
1	0.666772000	3.630203000	4.221729000	1	-0.647638000	-1.630858000	-4.404995000
6	2.684851000	3.612661000	3.467566000	6	-2.767433000	-1.605188000	-4.005149000
1	3.114292000	4.051219000	4.364410000	1	-3.024775000	-2.238091000	-4.854249000
6	3.503663000	3.274341000	2.387306000	6	-3.764679000	-1.135721000	-3.150507000
1	4.575807000	3.445292000	2.441836000	1	-4.805019000	-1.401281000	-3.328607000
6	2.947905000	2.724336000	1.238870000	6	-3.429387000	-0.324655000	-2.070412000
1	3.580249000	2.476700000	0.388851000	1	-4.209039000	0.055850000	-1.414250000
6	1.105192000	3.143828000	-1.225394000	6	-2.603173000	2.253974000	-0.730883000
6	1.329736000	2.821297000	-2.565681000	6	-3.000775000	2.882197000	0.448211000
1	1.479986000	1.780748000	-2.841580000	1	-2.786239000	2.406522000	1.396341000
6	1.358614000	3.813396000	-3.541162000	6	-3.655113000	4.110400000	0.408985000
1	1.537641000	3.544253000	-4.579094000	1	-3.966725000	4.589704000	1.334387000
6	1.160388000	5.147593000	-3.188160000	6	-3.916708000	4.730244000	-0.816045000
1	1.181553000	5.923913000	-3.948493000	1	-4.427550000	5.689190000	-0.847118000
6	0.942180000	5.479274000	-1.852863000	6	-3.514487000	4.107727000	-1.997152000
1	0.794343000	6.517493000	-1.566208000	1	-3.710740000	4.580714000	-2.957494000
6	0.921501000	4.484226000	-0.877717000	6	-2.861562000	2.877471000	-1.953570000
1	0.772445000	4.748133000	0.166600000	1	-2.549914000	2.388448000	-2.872087000
6	2.880389000	-1.576318000	-2.296214000	6	-1.117759000	-0.698094000	3.081229000
6	3.826821000	-0.367182000	-2.324940000	6	-1.177598000	0.836966000	3.054010000
1	3.958775000	-0.006186000	-3.353383000	1	-0.418742000	1.242282000	3.733427000
1	4.813328000	-0.606039000	-1.916969000	1	-2.142584000	1.234590000	3.381186000
1	3.398225000	0.449045000	-1.728813000	1	-0.953035000	1.209499000	2.044614000
6	1.602976000	-1.182658000	-3.044759000	6	0.372579000	-1.061866000	3.055301000
1	1.855750000	-1.040013000	-4.103655000	1	0.800548000	-0.810181000	4.035470000
1	1.160640000	-0.262294000	-2.658993000	1	0.919464000	-0.500142000	2.302132000
1	0.850091000	-1.970900000	-2.952226000	1	0.514377000	-2.139953000	2.883068000

6	3.485054000	-2.735551000	-3.100938000	6	-1.621977000	-1.221900000	4.439221000
1	2.686202000	-3.464231000	-3.300528000	1	-1.271563000	-2.253685000	4.553651000
1	3.813326000	-2.336310000	-4.068963000	1	-1.142046000	-0.623658000	5.223313000
6	4.613188000	-3.449437000	-2.382845000	6	-3.130669000	-1.215589000	4.585383000
1	5.456629000	-2.767469000	-2.212098000	1	-3.520406000	-0.189880000	4.540457000
1	4.991718000	-4.279056000	-2.992681000	1	-3.420211000	-1.625823000	5.563567000
6	4.058946000	-3.965144000	-1.068121000	6	-3.706304000	-2.048120000	3.456828000
1	4.811993000	-4.532547000	-0.505694000	1	-4.800889000	-2.104532000	3.517464000
1	3.224618000	-4.647116000	-1.287728000	1	-3.312873000	-3.072499000	3.527824000
6	3.540363000	-2.836885000	-0.163411000	6	-3.329943000	-1.481249000	2.079674000
6	2.786152000	-3.538449000	0.972430000	6	-3.955011000	-2.413619000	1.038568000
1	3.444272000	-4.277656000	1.445664000	1	-5.041728000	-2.382143000	1.190090000
1	1.910256000	-4.051631000	0.563297000	1	-3.617029000	-3.445706000	1.159906000
1	2.442537000	-2.855216000	1.754919000	1	-3.740897000	-2.084042000	0.017327000
6	4.758866000	-2.101830000	0.425018000	6	-4.065097000	-0.143617000	1.888592000
1	5.318893000	-2.787894000	1.074104000	1	-5.131937000	-0.295302000	2.102414000
1	4.451400000	-1.234286000	1.011716000	1	-3.981943000	0.212249000	0.859578000
1	5.442201000	-1.759921000	-0.357144000	1	-3.711539000	0.648678000	2.552071000
5	1.624237000	-1.175030000	-0.178815000	5	-1.080691000	-1.717603000	0.788143000
6	0.409601000	-0.617949000	2.565967000	6	0.197999000	-3.352225000	-1.338187000
1	-0.215088000	0.159826000	2.098727000	1	0.428107000	-3.686966000	-2.343978000
1	0.094615000	-1.556498000	2.098117000	1	0.265135000	-2.172317000	-1.072584000
1	0.243023000	-0.626011000	3.645259000	1	0.970745000	-3.618904000	-0.608719000

I2$_1$

E(scf)= −2070.86010038 a.u.

8	1.643735000	-2.764510000	0.476828000
7	0.155340000	-1.045251000	0.221759000
1	-0.281500000	-1.652580000	0.907777000
7	2.114300000	-1.197577000	-1.439185000
6	1.979485000	-3.995751000	0.029930000
6	1.898582000	-4.412298000	-1.238988000
1	1.583366000	-3.750654000	-2.036274000
1	2.170089000	-5.433025000	-1.485108000
6	2.441613000	-4.853280000	1.163465000
1	2.676299000	-5.867572000	0.828298000
1	1.667543000	-4.894213000	1.940514000
1	3.333448000	-4.405999000	1.622549000
6	-0.552701000	0.152537000	0.045948000
6	-1.936241000	0.086148000	-0.183188000
6	-2.674778000	1.259824000	-0.330316000
1	-3.738635000	1.175865000	-0.523568000
6	-2.070125000	2.511466000	-0.243457000
6	-0.702966000	2.556184000	0.043757000
1	-0.216122000	3.519788000	0.159046000
6	0.059673000	1.405959000	0.209443000
6	-2.831801000	3.815706000	-0.427388000
6	-2.727538000	4.651542000	0.854393000
1	-3.257687000	5.604086000	0.724324000
1	-1.682422000	4.871084000	1.101398000
1	-3.174986000	4.115913000	1.700623000
6	-4.309217000	3.584545000	-0.737456000

1

E(scf)= −2070.86942435 a.u.

8	-1.154735000	0.214278000	-2.141241000
7	-0.632694000	-0.639139000	0.003756000
1	-0.725892000	-1.281782000	0.773956000
7	-2.742358000	-1.419526000	-1.167265000
6	-0.056881000	-0.099400000	-2.879342000
6	0.248216000	-1.358323000	-3.218479000
1	-0.380842000	-2.190238000	-2.912245000
1	1.119338000	-1.563105000	-3.830846000
6	0.730327000	1.106555000	-3.271767000
1	1.544082000	0.843129000	-3.953713000
1	1.147652000	1.584489000	-2.375822000
1	0.074693000	1.837538000	-3.763822000
6	0.530259000	0.156788000	0.067939000
6	0.441051000	1.553063000	0.165816000
6	1.616019000	2.304157000	0.236072000
1	1.533000000	3.382629000	0.301680000
6	2.874983000	1.706271000	0.226257000
6	2.936443000	0.313791000	0.157356000
1	3.903483000	-0.179766000	0.147647000
6	1.788918000	-0.464887000	0.066782000
6	4.169338000	2.505698000	0.272634000
6	4.983025000	2.088316000	1.503500000
1	5.928980000	2.644634000	1.532944000
1	5.215109000	1.017279000	1.480646000
1	4.425199000	2.299740000	2.423905000
6	3.920453000	4.010992000	0.342227000

1	-4.809100000	4.551160000	-0.872463000	1	4.882131000	4.537401000	0.366950000
1	-4.809342000	3.051456000	0.080563000	1	3.357107000	4.281485000	1.243785000
1	-4.435698000	2.999406000	-1.656580000	1	3.357952000	4.362586000	-0.531360000
6	-2.207086000	4.600075000	-1.588322000	6	4.983866000	2.205090000	-0.992367000
1	-2.735335000	5.552928000	-1.724280000	1	5.932370000	2.757630000	-0.967455000
1	-2.275085000	4.026381000	-2.520610000	1	4.426566000	2.507625000	-1.887475000
1	-1.149713000	4.815912000	-1.395998000	1	5.208893000	1.135306000	-1.075393000
6	-2.591148000	-1.283926000	-0.169398000	6	-0.925647000	2.213313000	0.221183000
1	-1.964111000	-1.937642000	-0.792346000	1	-1.482868000	1.853116000	-0.650534000
6	-2.539226000	-1.855259000	1.236965000	6	-1.727702000	1.815529000	1.445243000
6	-2.225352000	-3.202502000	1.433556000	6	-3.104207000	2.054217000	1.442565000
1	-2.045336000	-3.834719000	0.566564000	1	-3.557039000	2.506372000	0.563123000
6	-2.110154000	-3.725650000	2.719337000	6	-3.889631000	1.711034000	2.537567000
1	-1.850088000	-4.772133000	2.855196000	1	-4.960822000	1.894791000	2.513641000
6	-2.314381000	-2.905486000	3.827475000	6	-3.306416000	1.118548000	3.657129000
1	-2.217335000	-3.310229000	4.831310000	1	-3.919093000	0.837859000	4.509671000
6	-2.633526000	-1.561201000	3.641546000	6	-1.932956000	0.888008000	3.673419000
1	-2.790459000	-0.913827000	4.500718000	1	-1.467781000	0.428695000	4.542429000
6	-2.739765000	-1.039093000	2.354326000	6	-1.148153000	1.242260000	2.577104000
1	-2.958013000	0.015535000	2.203264000	1	-0.077159000	1.058207000	2.587287000
6	-3.975021000	-1.271013000	-0.779896000	6	-0.834773000	3.715936000	0.065059000
6	-5.138742000	-1.234894000	-0.012729000	6	-0.750873000	4.569381000	1.165741000
1	-5.065488000	-1.278146000	1.070556000	1	-0.826283000	4.150585000	2.166032000
6	-6.387793000	-1.150218000	-0.627725000	6	-0.574010000	5.939635000	0.984575000
1	-7.287152000	-1.125231000	-0.017642000	1	-0.510424000	6.594703000	1.849828000
6	-6.484803000	-1.099218000	-2.015634000	6	-0.479045000	6.470148000	-0.300577000
1	-7.458555000	-1.034050000	-2.493883000	1	-0.342202000	7.539136000	-0.441457000
6	-5.324253000	-1.134746000	-2.789535000	6	-0.564099000	5.623057000	-1.405273000
1	-5.390339000	-1.097747000	-3.873943000	1	-0.494081000	6.029928000	-2.410944000
6	-4.080588000	-1.218826000	-2.173066000	6	-0.739527000	4.254743000	-1.220112000
1	-3.170126000	-1.234843000	-2.769602000	1	-0.792263000	3.582477000	-2.074583000
6	1.516036000	1.453404000	0.617862000	6	1.846168000	-1.974961000	-0.038735000
1	2.042340000	0.840077000	-0.121532000	1	1.147436000	-2.228340000	-0.852369000
6	1.780746000	0.805928000	1.970198000	6	1.322963000	-2.685785000	1.197664000
6	0.770423000	0.385069000	2.835574000	6	0.987529000	-2.014709000	2.374139000
1	-0.270601000	0.522491000	2.556601000	1	1.145243000	-0.941182000	2.436816000
6	1.081072000	-0.245696000	4.038044000	6	0.422370000	-2.701493000	3.449854000
1	0.274978000	-0.589831000	4.681405000	1	0.147012000	-2.156380000	4.349201000
6	2.409535000	-0.452447000	4.401237000	6	0.209112000	-4.074205000	3.369968000
1	2.651851000	-0.950491000	5.336430000	1	-0.235457000	-4.609591000	4.204594000
6	3.427211000	-0.015433000	3.554387000	6	0.571179000	-4.758981000	2.208593000
1	4.468690000	-0.165320000	3.827544000	1	0.411718000	-5.831787000	2.136764000
6	3.111081000	0.610748000	2.352383000	6	1.121110000	-4.068544000	1.135485000
1	3.904685000	0.958725000	1.694681000	1	1.393098000	-4.596666000	0.224397000
6	2.092058000	2.848601000	0.533497000	6	3.219355000	-2.453614000	-0.457679000
6	2.583189000	3.319634000	-0.685922000	6	3.599572000	-2.299646000	-1.793562000
1	2.593855000	2.651176000	-1.542320000	1	2.874087000	-1.893663000	-2.494867000
6	3.046892000	4.627076000	-0.808167000	6	4.883026000	-2.637598000	-2.209876000
1	3.430863000	4.977799000	-1.762846000	1	5.164704000	-2.515047000	-3.252736000
6	3.020294000	5.482529000	0.291979000	6	5.807416000	-3.132819000	-1.289340000
1	3.382665000	6.503081000	0.199771000	1	6.810983000	-3.398026000	-1.611560000
6	2.528953000	5.020768000	1.512273000	6	5.436544000	-3.286080000	0.044357000
1	2.505374000	5.681792000	2.374997000	1	6.151505000	-3.669605000	0.767914000

6	2.068475000	3.711687000	1.631276000	6	4.148912000	-2.946162000	0.458240000
1	1.689459000	3.344533000	2.581633000	1	3.858434000	-3.063527000	1.498908000
6	1.428285000	-0.869505000	-2.720400000	6	-3.116345000	-2.249429000	0.021566000
6	1.339264000	0.636078000	-3.015656000	6	-3.471231000	-1.365688000	1.223430000
1	0.615696000	0.802010000	-3.824049000	1	-3.640557000	-1.993955000	2.107212000
1	2.290097000	1.062463000	-3.347170000	1	-4.378703000	-0.783719000	1.040911000
1	0.988441000	1.178638000	-2.133080000	1	-2.672031000	-0.656980000	1.450625000
6	-0.015657000	-1.395498000	-2.735180000	6	-1.955265000	-3.201714000	0.408363000
1	-0.371435000	-1.369432000	-3.772586000	1	-2.338563000	-4.210727000	0.598731000
1	-0.686513000	-0.774003000	-2.140853000	1	-1.438734000	-2.904971000	1.328369000
1	-0.071645000	-2.431998000	-2.382177000	1	-1.216695000	-3.271332000	-0.395740000
6	2.119649000	-1.586385000	-3.897386000	6	-4.312845000	-3.165868000	-0.269541000
1	1.877970000	-2.657179000	-3.839778000	1	-3.988860000	-3.967607000	-0.949562000
1	1.668496000	-1.205407000	-4.823181000	1	-4.587595000	-3.638431000	0.681698000
6	3.629821000	-1.443511000	-3.923035000	6	-5.477741000	-2.443406000	-0.907831000
1	3.916296000	-0.393026000	-4.066592000	1	-5.824427000	-1.615212000	-0.276478000
1	4.045835000	-2.006272000	-4.767829000	1	-6.327489000	-3.122196000	-1.050363000
6	4.173162000	-1.965328000	-2.604976000	6	-4.969925000	-1.937942000	-2.239266000
1	5.268506000	-1.901980000	-2.570919000	1	-5.754354000	-1.426786000	-2.810997000
1	3.891185000	-3.019878000	-2.483727000	1	-4.650721000	-2.805699000	-2.835154000
6	3.605439000	-1.182577000	-1.415418000	6	-3.790563000	-0.959225000	-2.131471000
6	4.187312000	-1.806282000	-0.143790000	6	-3.245984000	-0.855393000	-3.566509000
1	5.264799000	-1.602638000	-0.142776000	1	-4.092819000	-0.856149000	-4.264656000
1	4.051195000	-2.889551000	-0.129704000	1	-2.609519000	-1.718378000	-3.789192000
1	3.757906000	-1.372173000	0.765162000	1	-2.667456000	0.053144000	-3.729646000
6	4.173373000	0.249341000	-1.450907000	6	-4.314252000	0.414490000	-1.694525000
1	5.265341000	0.188591000	-1.540691000	1	-5.055085000	0.788497000	-2.413283000
1	3.949633000	0.791124000	-0.527471000	1	-3.483647000	1.128698000	-1.652108000
1	3.808396000	0.842045000	-2.288096000	1	-4.779023000	0.367649000	-0.704050000
5	1.339521000	-1.633866000	-0.303880000	5	-1.535907000	-0.657790000	-1.095642000

Table S3. Cartesian coordinates (xyz) of the optimised geometries for all the species involved in the putative {**IV** + acetone} [2+2] cycloaddition mechanism to give **A** calculated at the level. Coordinates are given in Å.

TS$_{IV\text{-}A}$
E(scf)= −2070.80367270 a.u.
ν_{min}= −545.8924 cm^{-1}

8	-1.076629000	-2.250253000	2.628724000
7	-0.021493000	-0.854776000	0.968130000
7	-1.921489000	-2.414577000	-0.050003000
6	-0.189860000	-1.330149000	2.889188000
6	1.142568000	-1.818223000	3.368547000
1	1.848100000	-1.011172000	3.569455000
1	1.566572000	-2.521504000	2.647288000
1	0.945160000	-2.367336000	4.300652000
6	0.392863000	0.172471000	0.130230000
6	-0.482222000	1.197396000	-0.277911000
6	-0.042802000	2.182878000	-1.163603000
1	-0.760288000	2.939532000	-1.466915000
6	1.256333000	2.207511000	-1.657225000
6	2.124300000	1.201570000	-1.221470000
1	3.148508000	1.191741000	-1.583565000
6	1.722451000	0.199654000	-0.351059000
6	1.759741000	3.261110000	-2.631312000
6	2.943575000	4.007203000	-2.003746000
1	3.327630000	4.760029000	-2.704560000
1	3.759698000	3.317839000	-1.758609000
1	2.633656000	4.514258000	-1.081697000
6	0.680614000	4.280421000	-2.989748000
1	1.091424000	5.014131000	-3.693775000
1	0.331126000	4.817887000	-2.099305000
1	-0.183514000	3.796591000	-3.461371000
6	2.227606000	2.571803000	-3.919131000
1	2.611126000	3.319179000	-4.626282000
1	1.395218000	2.037184000	-4.392278000
1	3.025603000	1.849094000	-3.713084000
6	-1.892361000	1.289001000	0.256313000
1	-2.119259000	0.331533000	0.733970000
6	-2.060050000	2.328443000	1.348419000
6	-3.284748000	2.371942000	2.025257000
1	-4.080005000	1.697788000	1.713301000
6	-3.487997000	3.255142000	3.079237000
1	-4.444294000	3.270035000	3.595935000
6	-2.464235000	4.117367000	3.474297000
1	-2.618155000	4.807529000	4.299626000
6	-1.245328000	4.085241000	2.803717000
1	-0.441308000	4.752941000	3.103392000
6	-1.044000000	3.196194000	1.747340000
1	-0.087329000	3.170466000	1.233855000
6	-2.880311000	1.438073000	-0.883934000
6	-3.482121000	2.651240000	-1.215332000

A
E(scf)= −2070.87849003 a.u.

8	-1.490061000	-2.169644000	1.861472000
7	-0.230651000	-1.041371000	0.615337000
7	-2.410434000	-2.129221000	-0.497392000
6	-0.166480000	-1.605724000	1.988636000
6	0.799819000	-2.763060000	2.162117000
1	1.826689000	-2.416299000	2.297026000
1	0.744742000	-3.426519000	1.291225000
1	0.502594000	-3.326704000	3.053650000
6	0.383325000	0.165541000	0.232954000
6	-0.350824000	1.357362000	0.053695000
6	0.324578000	2.542505000	-0.238173000
1	-0.269660000	3.440556000	-0.370297000
6	1.708212000	2.601290000	-0.387273000
6	2.405403000	1.402665000	-0.277385000
1	3.478294000	1.383477000	-0.452269000
6	1.771476000	0.197604000	0.008833000
6	2.458930000	3.883837000	-0.708992000
6	3.500978000	4.144184000	0.385237000
1	4.070476000	5.053629000	0.153534000
1	4.205422000	3.308085000	0.466033000
1	3.012858000	4.276510000	1.358678000
6	1.529903000	5.092672000	-0.795257000
1	2.117949000	5.989131000	-1.025215000
1	1.006387000	5.260512000	0.154338000
1	0.778523000	4.962843000	-1.583968000
6	3.173301000	3.721570000	-2.056793000
1	3.739280000	4.632271000	-2.292643000
1	2.445803000	3.544206000	-2.857915000
1	3.870540000	2.876064000	-2.038689000
6	-1.859356000	1.391997000	0.210058000
1	-2.251335000	0.409267000	-0.081917000
6	-2.227585000	1.590101000	1.665137000
6	-3.168208000	0.751604000	2.259670000
1	-3.619438000	-0.034071000	1.665101000
6	-3.494361000	0.887240000	3.607112000
1	-4.213759000	0.209197000	4.058889000
6	-2.885172000	1.878216000	4.372406000
1	-3.127312000	1.981961000	5.426824000
6	-1.958421000	2.736411000	3.778842000
1	-1.479915000	3.512654000	4.370590000
6	-1.634034000	2.594477000	2.433390000
1	-0.889919000	3.241521000	1.977290000
6	-2.551672000	2.388515000	-0.712823000
6	-3.661562000	3.122544000	-0.289148000

1	-3.286488000	3.527514000	-0.602633000	1	-4.004713000	3.026817000	0.736803000
6	-4.338139000	2.735654000	-2.314178000	6	-4.331608000	3.974907000	-1.166040000
1	-4.800619000	3.687460000	-2.563306000	1	-5.191660000	4.538094000	-0.812578000
6	-4.605968000	1.608190000	-3.086917000	6	-3.902675000	4.108736000	-2.483211000
1	-5.278094000	1.676508000	-3.938271000	1	-4.422848000	4.776103000	-3.165346000
6	-4.008446000	0.390666000	-2.757084000	6	-2.794921000	3.381811000	-2.917520000
1	-4.212981000	-0.499964000	-3.347910000	1	-2.444770000	3.479011000	-3.942149000
6	-3.149464000	0.311575000	-1.667476000	6	-2.127913000	2.532270000	-2.040982000
1	-2.679157000	-0.630119000	-1.396602000	1	-1.252264000	1.985662000	-2.378857000
6	2.717951000	-0.809675000	0.179006000	6	2.606103000	-1.064604000	-0.008302000
1	2.152130000	-1.729159000	0.391567000	1	1.907121000	-1.908220000	0.049104000
6	3.301608000	-0.364596000	1.510439000	6	3.561910000	-1.197345000	1.161338000
6	2.950830000	0.837574000	2.124172000	6	3.658853000	-0.250405000	2.181352000
1	2.269727000	1.516586000	1.619957000	1	3.058783000	0.653605000	2.130847000
6	3.442031000	1.157318000	3.390972000	6	4.513611000	-0.458427000	3.265217000
1	3.147302000	2.092728000	3.860016000	1	4.571557000	0.288779000	4.052576000
6	4.296907000	0.281952000	4.054253000	6	5.285761000	-1.612950000	3.340326000
1	4.673024000	0.526999000	5.043996000	1	5.949686000	-1.774663000	4.185353000
6	4.673411000	-0.911451000	3.434752000	6	5.200917000	-2.563005000	2.321028000
1	5.345292000	-1.599960000	3.940841000	1	5.799562000	-3.469085000	2.368253000
6	4.182855000	-1.225130000	2.173400000	6	4.347779000	-2.353781000	1.244515000
1	4.469703000	-2.156350000	1.690018000	1	4.281464000	-3.090958000	0.447597000
6	3.788231000	-1.153796000	-0.836551000	6	3.298841000	-1.158902000	-1.358597000
6	3.550010000	-2.154610000	-1.781067000	6	2.541853000	-1.539286000	-2.470055000
1	2.614550000	-2.707358000	-1.742410000	1	1.497679000	-1.806525000	-2.330025000
6	4.490191000	-2.442917000	-2.766424000	6	3.106799000	-1.562714000	-3.740699000
1	4.289826000	-3.227753000	-3.491456000	1	2.504650000	-1.864108000	-4.594376000
6	5.686309000	-1.728926000	-2.821448000	6	4.441866000	-1.199700000	-3.918788000
1	6.421871000	-1.952416000	-3.589683000	1	4.886014000	-1.216605000	-4.910569000
6	5.935410000	-0.731776000	-1.880562000	6	5.202711000	-0.818269000	-2.816448000
1	6.867652000	-0.173462000	-1.911805000	1	6.244603000	-0.535789000	-2.945097000
6	4.993900000	-0.449901000	-0.893185000	6	4.634733000	-0.799809000	-1.543175000
1	5.191331000	0.318830000	-0.150333000	1	5.233107000	-0.513622000	-0.682019000
6	-1.319646000	-3.107600000	-1.223465000	6	-1.864010000	-2.208443000	-1.884001000
6	-0.684073000	-4.437323000	-0.806315000	6	-0.748606000	-3.259399000	-1.966315000
1	-0.105007000	-4.850991000	-1.641814000	1	-0.270677000	-3.225151000	-2.954035000
1	-1.429765000	-5.179082000	-0.509961000	1	-1.127135000	-4.273216000	-1.808537000
1	-0.010430000	-4.270442000	0.042624000	1	0.012496000	-3.050342000	-1.205155000
6	-0.225346000	-2.229349000	-1.829275000	6	-1.290056000	-0.861490000	-2.316630000
1	0.121597000	-2.688244000	-2.762805000	1	-1.035352000	-0.914962000	-3.382829000
1	0.631032000	-2.132473000	-1.157161000	1	-0.381974000	-0.589507000	-1.778575000
1	-0.600456000	-1.223350000	-2.046249000	1	-2.038376000	-0.074281000	-2.184379000
6	-2.372808000	-3.331385000	-2.312575000	6	-2.951396000	-2.532454000	-2.916455000
1	-2.610862000	-2.362359000	-2.774552000	1	-3.585733000	-1.643173000	-3.042974000
1	-1.914882000	-3.957065000	-3.089041000	1	-2.444790000	-2.710283000	-3.873379000
6	-3.655821000	-3.948476000	-1.784091000	6	-3.834320000	-3.697114000	-2.521470000
1	-3.457885000	-4.937857000	-1.351763000	1	-3.242703000	-4.614328000	-2.405152000
1	-4.373032000	-4.092875000	-2.601067000	1	-4.585113000	-3.892095000	-3.296734000
6	-4.228659000	-3.009870000	-0.736324000	6	-4.498814000	-3.312299000	-1.215754000
1	-5.172650000	-3.391198000	-0.327853000	1	-5.200162000	-4.082507000	-0.871668000
1	-4.443077000	-2.041665000	-1.209905000	1	-5.076658000	-2.391105000	-1.379740000
6	-3.278312000	-2.781691000	0.445794000	6	-3.496729000	-3.070041000	-0.081154000
6	-3.843780000	-1.613674000	1.256692000	6	-4.307957000	-2.451704000	1.060205000

1	-4.844381000	-1.884250000	1.614180000	1	-5.126354000	-3.133908000	1.320900000
1	-3.937007000	-0.722160000	0.625917000	1	-4.748705000	-1.505028000	0.725320000
1	-3.215638000	-1.399635000	2.128095000	1	-3.703977000	-2.280271000	1.954254000
6	-3.244661000	-4.026137000	1.339445000	6	-2.938409000	-4.421611000	0.388003000
1	-4.247522000	-4.194864000	1.752256000	1	-3.760081000	-5.043465000	0.765379000
1	-2.542218000	-3.882398000	2.164358000	1	-2.213547000	-4.274540000	1.192542000
1	-2.961420000	-4.922514000	0.780830000	1	-2.455950000	-4.966209000	-0.428606000
5	-1.036009000	-1.741275000	0.797878000	5	-1.503949000	-1.732797000	0.529453000
6	-0.642568000	0.001456000	3.412825000	6	-0.030466000	-0.599561000	3.104159000
1	-1.602385000	0.288303000	2.978486000	1	-0.794882000	0.173722000	3.042745000
1	-0.761479000	-0.091486000	4.503226000	1	-0.113819000	-1.117870000	4.065741000
1	0.100884000	0.778305000	3.210785000	1	0.962488000	-0.138736000	3.043692000

Table S4. Cartesian coordinates (xyz) of the optimised geometries for all the species involved in the {**II** + acetone} reaction mechanism calculated at the level. Coordinates are given in Å.

{II + acetone}
E(oniom)= –697.290733938760 a.u.

N	-0.603045	-0.507982	-3.031992
C	-1.220116	0.174040	-1.992787
B	-0.846377	0.108814	-0.578724
N	-2.240638	0.912121	-2.575985
C	-1.431409	-0.453322	-4.238110
H	-2.177339	-1.269283	-4.261328
C	-2.105303	0.902765	-4.036204
H	-3.079739	0.975629	-4.523754
C	0.161511	-1.697874	-2.713972
C	1.565054	-1.610890	-2.912420
C	2.351624	-2.731783	-2.622099
H	3.431424	-2.688959	-2.755528
C	1.767792	-3.909228	-2.147552
H	2.394814	-4.773576	-1.935289
C	0.392445	-3.980065	-1.937780
H	-0.053092	-4.897731	-1.560142
C	-0.429815	-2.871713	-2.204072
C	2.162640	-0.316336	-3.395906
H	1.978041	0.455425	-2.610445
C	3.649428	-0.351570	-3.729221
H	4.266624	-0.539587	-2.835422
H	3.980951	0.611029	-4.134332
H	3.887742	-1.122120	-4.469074
C	-1.902016	-2.990884	-1.913790
H	-2.320639	-2.019234	-1.569280
C	-2.678512	-3.506914	-3.123376
H	-2.652911	-2.778628	-3.949748
H	-3.729056	-3.685079	-2.869831
H	-2.260150	-4.447834	-3.498712
C	-2.738670	2.095188	-1.904830
C	-4.135121	2.136443	-1.656365
C	-4.669531	3.258565	-1.013304
H	-5.738663	3.325012	-0.824979
C	-3.837752	4.299813	-0.594661
H	-4.267278	5.161379	-0.085978
C	-2.464158	4.239721	-0.817573
H	-1.827836	5.050295	-0.467045
C	-1.889230	3.144886	-1.484328
C	-5.000221	0.980799	-2.088161
H	-4.494182	0.030955	-1.792589
C	-6.419599	0.975267	-1.528359

$TS1_2$
E(oniom)= –697.273706444634 a.u.
νmin = –127.6 cm^{-1}

N	-3.061147	1.384061	0.035440
C	-2.190833	0.330669	-0.350383
B	-0.775431	0.241695	-0.082406
N	-2.981640	-0.502906	-1.188749
C	-4.408823	1.166443	-0.501462
H	-4.551620	1.782930	-1.396673
C	-4.399606	-0.316327	-0.859397
H	-5.028859	-0.555297	-1.720665
C	-2.897010	1.997526	1.336014
C	-3.489340	1.502779	2.520994
C	-3.349603	2.223739	3.717399
H	-3.819654	1.852393	4.625766
C	-2.623474	3.413158	3.751964
H	-2.532759	3.970783	4.681920
C	-2.013244	3.885743	2.591188
H	-1.440016	4.811443	2.620696
C	-2.129110	3.188212	1.378294
C	-4.268086	0.216096	2.581597
H	-3.949792	-0.476453	1.762370
C	-5.774402	0.460854	2.516652
H	-6.115517	1.081475	3.354121
H	-6.334726	-0.479058	2.547834
H	-6.049771	0.989752	1.590200
C	-1.430882	3.748766	0.168967
H	-1.650419	3.142502	-0.749422
C	0.082119	3.794847	0.367516
H	0.372377	4.343471	1.266858
H	0.572598	4.271933	-0.489579
H	0.496859	2.775995	0.441483
C	-2.569334	-1.870263	-1.452148
C	-2.325033	-2.198853	-2.808265
C	-1.980870	-3.515840	-3.148665
H	-1.783249	-3.765733	-4.189191
C	-1.905401	-4.507180	-2.170963
H	-1.653032	-5.528826	-2.448227
C	-2.156970	-4.187157	-0.837708
H	-2.104072	-4.964283	-0.077290
C	-2.474276	-2.871840	-0.459471
C	-2.438614	-1.189003	-3.918459
H	-2.392588	-0.145666	-3.506780

H	-6.416407	0.991218	-0.431167
H	-6.956590	0.071803	-1.839398
H	-7.000627	1.837573	-1.874250
C	0.278889	4.445537	-1.835619
H	-0.196040	5.055810	-2.611450
H	1.334108	4.320520	-2.106257
H	0.253252	5.012217	-0.897189
C	-0.401199	3.086097	-1.692126
H	-0.143883	2.467724	-2.576823
N	0.984382	0.381736	2.895257
C	-0.067338	-0.235547	2.239972
B	-0.478278	-0.040707	0.846673
N	-0.653911	-1.078995	3.173472
C	0.898619	0.157358	4.338230
H	0.286066	0.928768	4.839662
C	0.199893	-1.202319	4.357954
H	-0.397940	-1.363711	5.257411
C	1.508617	1.617957	2.354353
C	0.789321	2.832293	2.400221
C	1.384135	3.989344	1.872708
H	0.846327	4.934270	1.913957
C	2.648679	3.936363	1.286104
H	3.094739	4.841049	0.876680
C	3.338001	2.725289	1.208716
H	4.307857	2.684517	0.717615
C	2.778802	1.551857	1.734878
C	-0.600817	2.934473	2.968638
H	-1.166632	1.989414	2.807689
C	3.528013	0.252867	1.594202
H	3.268778	-0.443275	2.417950
C	3.218131	-0.404252	0.250015
H	3.704846	0.121588	-0.583577
H	3.558610	-1.443364	0.220143
H	2.134579	-0.410365	0.043672
C	-1.387703	-2.228398	2.681305
C	-0.756211	-3.314949	2.035371
C	-1.540427	-4.404731	1.624603
H	-1.063608	-5.253966	1.139362
C	-2.920779	-4.406553	1.825952
H	-3.515133	-5.260357	1.505054
C	-3.544071	-3.312202	2.426599
H	-4.624385	-3.307461	2.555248
C	-2.791363	-2.207780	2.852045
C	0.720385	-3.350641	1.745204
H	1.124440	-2.326481	1.581538
C	1.499258	-4.057986	2.852239
H	1.100172	-5.059427	3.049670
C	-3.734603	-1.369055	-4.707340
H	-3.799637	-2.366126	-5.157454
H	-3.809318	-0.632102	-5.514179
H	-4.610884	-1.243849	-4.060537
C	-2.707186	-2.612846	1.004950
H	-3.157454	-1.605338	1.175103
C	-1.409755	-2.714689	1.803231
H	-0.975708	-3.718997	1.761793
H	-1.566087	-2.459063	2.854578
H	-0.643492	-2.022653	1.405869
N	3.123877	-0.917189	-0.255077
C	2.069474	-0.065274	-0.578607
B	0.753186	0.173714	0.033138
N	2.432984	0.559042	-1.787604
C	3.889971	-1.226255	-1.469011
H	3.448370	-2.082315	-2.012923
C	3.735055	0.062933	-2.262482
H	3.712944	-0.107891	-3.341715
C	2.970165	-2.011717	0.683838
C	2.089826	-3.094974	0.471211
C	2.036242	-4.122180	1.428835
H	1.360168	-4.960720	1.274152
C	2.839087	-4.080386	2.567344
H	2.785108	-4.882781	3.300403
C	3.719236	-3.014762	2.766235
H	4.345454	-2.990354	3.654974
C	3.801676	-1.974839	1.830397
C	1.207952	-3.209817	-0.741364
H	0.911616	-2.210825	-1.130229
C	1.869240	-4.029204	-1.848391
H	2.197241	-5.009492	-1.486080
H	1.170569	-4.193312	-2.677492
H	2.752018	-3.508515	-2.252197
C	4.774952	-0.850223	2.080087
H	5.169278	-0.457046	1.119407
C	4.111105	0.285258	2.859444
H	3.787983	-0.045501	3.851800
H	4.791352	1.132362	2.987039
H	3.216950	0.643562	2.325292
C	2.241513	1.994928	-1.917568
C	3.078980	2.935779	-1.269657
C	2.990465	4.289718	-1.625759
H	3.649368	5.014186	-1.151577
C	2.067315	4.715788	-2.581750
H	2.029346	5.763719	-2.873428
C	1.179273	3.801272	-3.144092
H	0.430288	4.143030	-3.856786

H	2.555511	-4.165544	2.582744
H	1.450576	-3.488535	3.794053
C	-3.505316	-1.017922	3.438766
H	-2.869670	-0.512848	4.195442
C	-3.869772	-0.026482	2.333273
H	-4.565298	-0.462865	1.608685
H	-4.324661	0.880226	2.740392
H	-2.974205	0.277601	1.762657
C	7.005788	0.345140	-0.915209
O	5.996985	0.055646	-1.522001
C	8.370462	0.283232	-1.567200
H	8.924983	1.211948	-1.393007
H	8.953487	-0.528520	-1.115946
H	8.267299	0.102239	-2.637955
C	6.968638	0.789623	0.530248
H	5.965108	0.634081	0.949834
H	7.712770	0.246326	1.122694
H	7.225197	1.854053	0.590314
H	-2.058829	-3.664635	-1.042628
H	1.598400	0.037302	-4.285684
H	0.040791	2.531692	-0.822154
H	-5.038343	0.959507	-3.198045
H	-4.417239	-1.339125	3.979486
H	0.896320	-3.856301	0.769853
H	4.626284	0.428198	1.680416
C	-0.583950	3.296371	4.452401
H	-1.598274	3.477578	4.824706
H	-0.149486	2.481546	5.052953
H	0.007908	4.199320	4.640751
H	-1.182229	3.691492	2.399587
H	-0.817449	-0.497638	-5.139804
H	-1.458235	1.717755	-4.391619
H	1.891286	0.133809	4.793051
H	0.932266	-2.023966	4.254322

I1$_2$
E(oniom)= – 697.300419168726 a.u.

N	-3.149140	-1.026517	0.056030
C	-2.084221	-0.088437	0.328425
B	-0.701753	-0.256496	-0.008811
N	-2.706392	0.915789	1.163411
C	-4.397983	-0.550259	0.664490
H	-4.552941	-1.057261	1.624606
C	-4.144250	0.935965	0.882254
H	-4.696158	1.352465	1.730720
C	-3.216391	-1.618581	-1.265853

C	1.228720	2.439843	-2.798578
C	4.055588	2.566307	-0.183081
H	3.823251	1.570961	0.254671
C	5.501307	2.589775	-0.678185
H	5.777664	3.571640	-1.078870
H	6.200095	2.347685	0.129520
H	5.649461	1.851746	-1.482971
C	0.187459	1.512026	-3.363776
H	0.259534	0.504855	-2.892411
C	-1.222320	2.058500	-3.140934
H	-1.465878	2.883304	-3.812757
H	-1.978241	1.263278	-3.276910
H	-1.353064	2.412232	-2.097827
C	0.740805	-0.295510	3.134409
O	1.166750	-0.757146	2.089894
C	0.234943	1.117165	3.240419
H	-0.848842	1.102775	3.041670
H	0.383006	1.523270	4.245137
H	0.687751	1.754349	2.480143
C	0.677404	-1.149532	4.377742
H	0.949199	-2.178891	4.134624
H	1.372066	-0.753668	5.127097
H	-0.327101	-1.109719	4.813304
H	5.659729	-1.223844	2.633312
H	0.238588	-3.675918	-0.457713
H	3.936615	3.273559	0.665369
H	0.377495	1.349561	-4.443102
H	-1.825398	4.761943	-0.046671
H	-4.018524	-0.327709	3.517052
H	-3.451528	-3.337537	1.395014
H	-1.562752	-1.270713	-4.593038
H	-5.190707	1.421456	0.227865
H	-4.718653	-0.928314	0.002717
H	4.929190	-1.460352	-1.227077
H	4.557927	0.763366	-2.035642

TS2$_2$
E(oniom)= – 697.272754642031 a.u.
νmin = –1186.9 cm^{-1}

N	3.062533	1.099592	0.026836
C	2.037608	0.157707	0.320489
B	0.662633	0.173491	-0.181590
N	2.636601	-0.754800	1.220210
C	4.303704	0.737898	0.730025
H	4.393471	1.346338	1.636593
C	4.095032	-0.730995	1.082872
H	4.576782	-1.023942	2.019462

C	-3.849979	-1.047351	-2.393256
C	-3.937998	-1.779122	-3.589578
H	-4.443246	-1.342378	-4.448385
C	-3.392783	-3.056854	-3.686851
H	-3.476459	-3.619253	-4.613669
C	-2.738657	-3.612324	-2.587320
H	-2.308372	-4.609624	-2.663365
C	-2.633888	-2.908435	-1.378725
C	-4.440074	0.337781	-2.406125
H	-3.961251	0.976073	-1.621170
C	-5.955652	0.309448	-2.212951
H	-6.450537	-0.254368	-3.012450
H	-6.377914	1.318941	-2.202511
H	-6.220763	-0.179345	-1.261688
C	-1.903250	-3.569243	-0.240125
H	-1.975836	-2.960752	0.700541
C	-0.430029	-3.786367	-0.577812
H	-0.287500	-4.263100	-1.550741
H	0.051701	-4.416578	0.177752
H	0.112962	-2.827255	-0.587810
C	-2.107142	2.230930	1.311978
C	-1.790925	2.607027	2.642975
C	-1.279138	3.887573	2.898500
H	-1.024788	4.169055	3.918146
C	-1.117953	4.809517	1.864173
H	-0.747223	5.809506	2.077680
C	-1.440019	4.447365	0.557708
H	-1.327299	5.172995	-0.245194
C	-1.912929	3.157120	0.262707
C	-2.003436	1.681673	3.811919
H	-2.253580	0.647072	3.452216
C	-3.120615	2.175099	4.730397
H	-2.894296	3.157713	5.158130
H	-3.283238	1.478275	5.559480
H	-4.066556	2.266614	4.183185
C	-2.216883	2.843370	-1.178456
H	-2.635342	1.813666	-1.286079
C	-0.977961	2.970701	-2.064760
H	-0.612115	4.002068	-2.110203
H	-1.188586	2.647333	-3.088485
H	-0.155938	2.351660	-1.673831
N	3.207537	0.476618	0.336635
C	2.027900	-0.234909	0.478985
B	0.833506	-0.250619	-0.461937
N	2.114118	-0.863152	1.717206
C	3.858700	0.678796	1.632863
H	3.500155	1.606275	2.116401

C	3.171750	1.596918	-1.332663
C	3.857851	0.927897	-2.373727
C	3.981723	1.545850	-3.628392
H	4.514199	1.034871	-4.427929
C	3.435885	2.806971	-3.862333
H	3.548060	3.278942	-4.836217
C	2.743341	3.457973	-2.844212
H	2.309884	4.440319	-3.026662
C	2.590793	2.864240	-1.580309
C	4.483465	-0.435564	-2.237482
H	4.010082	-1.024803	-1.416398
C	5.992067	-0.338991	-2.010612
H	6.490849	0.164962	-2.847505
H	6.445453	-1.328974	-1.899769
H	6.218717	0.243065	-1.104005
C	1.795573	3.612831	-0.545932
H	1.845099	3.111205	0.455472
C	0.334184	3.719215	-0.975978
H	0.221161	4.072606	-2.003775
H	-0.215118	4.403739	-0.322968
H	-0.157914	2.732117	-0.906828
C	2.037543	-2.036690	1.533439
C	1.698121	-2.266666	2.885119
C	1.169824	-3.515209	3.255592
H	0.895925	-3.692940	4.293501
C	1.011878	-4.525739	2.311340
H	0.619113	-5.495685	2.611017
C	1.359197	-4.300792	0.976294
H	1.234040	-5.107241	0.256922
C	1.864267	-3.061165	0.566371
C	1.896385	-1.227358	3.955724
H	2.146250	-0.232243	3.502488
C	2.999380	-1.626161	4.934993
H	2.762049	-2.558913	5.458792
H	3.151769	-0.849015	5.691907
H	3.953523	-1.777358	4.416866
C	2.215592	-2.795260	-0.868383
H	1.537952	-1.969220	-1.241003
C	2.131604	-3.981427	-1.820257
H	2.807033	-4.792691	-1.518621
H	2.411138	-3.681773	-2.840752
H	1.117801	-4.393647	-1.867966
N	-3.227603	-0.397331	0.293657
C	-2.046717	0.320995	0.367381
B	-0.869481	0.195853	-0.588006
N	-2.142011	1.082391	1.530827
C	-3.895046	-0.456539	1.595223

C	3.390193	-0.558268	2.387271
H	4.117651	-1.381963	2.274708
C	3.368264	1.520544	-0.654805
C	2.678589	2.750394	-0.579554
C	2.922477	3.722632	-1.564002
H	2.396967	4.675172	-1.514517
C	3.833282	3.485821	-2.592258
H	4.017237	4.252864	-3.343084
C	4.515660	2.269378	-2.659178
H	5.228905	2.093140	-3.461915
C	4.295110	1.274739	-1.696328
C	1.695119	3.068090	0.511253
H	1.172371	2.154056	0.872662
C	2.356155	3.793913	1.681282
H	2.895891	4.688424	1.352402
H	1.599658	4.108151	2.412845
H	3.072981	3.141112	2.203228
C	5.044926	-0.027392	-1.811377
H	5.204693	-0.478274	-0.808818
C	4.287464	-1.014635	-2.699381
H	4.242684	-0.672220	-3.738292
H	4.763087	-2.000923	-2.691911
H	3.251661	-1.140516	-2.347016
C	1.692044	-2.248401	1.861527
C	2.430698	-3.314649	1.291149
C	2.122612	-4.632069	1.659441
H	2.700960	-5.455603	1.245839
C	1.080267	-4.897313	2.549625
H	0.864875	-5.922690	2.845350
C	0.305614	-3.850859	3.043350
H	-0.529854	-4.064597	3.709127
C	0.580799	-2.517549	2.692300
C	3.535668	-3.118611	0.285448
H	3.467470	-2.127939	-0.210830
C	4.917793	-3.293383	0.913800
H	5.037938	-4.289618	1.355404
H	5.710991	-3.158529	0.171017
H	5.079366	-2.556758	1.717325
C	-0.306372	-1.421469	3.212095
H	-0.152741	-0.487230	2.618881
C	-1.786277	-1.791870	3.135965
H	-2.092467	-2.495377	3.910254
H	-2.410528	-0.881407	3.214146
H	-2.030323	-2.224758	2.143790
C	0.722894	0.182324	-2.921091
O	1.285114	0.222884	-1.789245
C	-0.602151	-0.444840	-3.121216
H	-3.557405	-1.335835	2.175076
C	-3.417403	0.842363	2.229340
H	-4.142536	1.655059	2.044655
C	-3.407289	-1.539397	-0.580375
C	-2.784000	-2.783180	-0.337368
C	-3.088270	-3.869647	-1.177295
H	-2.624766	-4.836248	-0.988557
C	-3.985437	-3.725391	-2.234814
H	-4.225117	-4.574935	-2.865392
C	-4.581644	-2.486275	-2.483126
H	-5.269504	-2.376013	-3.319115
C	-4.300268	-1.381117	-1.667547
C	-1.791737	-2.993257	0.771814
H	-1.251026	-2.051173	1.019002
C	-2.446568	-3.570355	2.025037
H	-2.996471	-4.492453	1.808635
H	-1.688310	-3.799938	2.784614
H	-3.155432	-2.853546	2.468736
C	-4.941839	-0.056580	-1.989790
H	-5.100832	0.538517	-1.065527
C	-4.069417	0.734073	-2.965085
H	-3.999498	0.237528	-3.938302
H	-4.462653	1.742115	-3.126736
H	-3.041442	0.831801	-2.576996
C	-1.740551	2.480361	1.540885
C	-2.491296	3.479904	0.876359
C	-2.208818	4.829286	1.137359
H	-2.799186	5.604849	0.653990
C	-1.175903	5.185098	2.005841
H	-0.979530	6.234984	2.216119
C	-0.384268	4.197275	2.588606
H	0.442975	4.482591	3.236747
C	-0.637513	2.836120	2.351568
C	-3.577753	3.176707	-0.122177
H	-3.484690	2.146456	-0.527086
C	-4.972087	3.383890	0.467686
H	-5.117152	4.415277	0.809531
H	-5.750907	3.163200	-0.269924
H	-5.132948	2.724236	1.335491
C	0.247004	1.788590	2.966968
H	0.151614	0.830858	2.394574
C	1.721017	2.187415	2.971401
H	1.953009	2.956077	3.710064
H	2.350934	1.302804	3.172882
H	2.019994	2.557482	1.969697
C	-0.537560	-0.880368	-2.791786
O	-1.311604	-0.536965	-1.816603

H	-1.412808	0.173696	-2.713023
H	-0.787411	-0.640583	-4.181531
H	-0.653295	-1.381083	-2.534681
C	1.482345	0.748314	-4.074905
H	2.448019	1.140511	-3.739201
H	1.643797	-0.016845	-4.844792
H	0.914661	1.559532	-4.548018
H	6.060808	0.149062	-2.219139
H	0.869914	3.694922	0.104693
H	3.404205	-3.847323	-0.543384
H	-0.017493	-1.170982	4.251635
H	-3.012852	3.527707	-1.539262
H	-1.057142	1.572026	4.377265
H	-4.196882	0.839173	-3.365899
H	-2.392833	-4.534186	-0.001063
H	-5.270356	-0.745887	0.024658
H	-4.401579	1.506540	-0.032281
H	3.234191	-0.373117	3.452233
H	4.944475	0.733902	1.526823

I2$_2$
E(oniom)= – 697.354919507640 a.u.

N	3.025528	0.904311	0.391712
C	1.651012	0.792090	0.124922
B	0.726534	-0.053895	0.926432
N	1.423979	1.700851	-0.947603
C	3.741340	1.363627	-0.786561
H	3.960503	0.540211	-1.493485
C	2.706942	2.323889	-1.356297
H	2.829798	3.316392	-0.897057
C	3.661299	-0.023945	1.302698
C	4.062084	0.497214	2.556961
C	4.691931	-0.348622	3.481069
H	4.999170	0.045463	4.447011
C	4.925382	-1.689601	3.173286
H	5.412693	-2.336534	3.899326
C	4.530809	-2.200716	1.937546
H	4.712341	-3.246966	1.703033
C	3.894266	-1.380747	0.991973
C	3.839893	1.938011	2.936236
H	3.004857	2.376469	2.331150
C	5.107995	2.767579	2.738413
H	5.939125	2.379933	3.338431
H	4.945339	3.811907	3.023424
H	5.426895	2.752810	1.689586
C	3.487194	-1.988306	-0.321186

C	0.768160	-0.365476	-2.889252
H	1.153101	-0.513139	-1.564835
H	1.390996	-0.785721	-3.679075
H	0.885415	0.719819	-2.781704
C	-1.024500	-2.027039	-3.627350
H	-0.830276	-1.846019	-4.722160
H	-0.474377	-2.964837	-3.323330
H	-2.122089	-2.172469	-3.432207
H	-5.951773	-0.209929	-2.419812
H	-0.983429	-3.673309	0.424128
H	-3.443722	3.829119	-1.012080
H	-0.103925	1.565201	3.993333
H	2.232340	4.619544	-0.395272
H	4.280524	-1.028294	-3.154889
H	3.247211	-2.359929	-0.925027
H	0.941343	-1.064003	4.494908
H	5.191888	0.906184	0.106608
H	4.449115	-1.390800	0.268784
H	-4.980630	-0.507519	1.481987
H	-3.260091	0.755101	3.307069

TS3$_2$
E(oniom)= –697.340791378712 a.u.
νmin = –139.2 cm$^{–1}$

N	-2.696080	1.796808	-0.043028
C	-1.318942	1.390177	0.010473
B	-0.785632	0.092698	-0.382676
N	-0.667927	2.571330	0.525212
C	-2.915672	2.959278	0.801647
H	-3.009092	2.714741	1.878786
C	-1.610080	3.695551	0.557871
H	-1.659736	4.239503	-0.401733
C	-3.732068	0.821239	-0.256226
C	-3.908542	0.356403	-1.592469
C	-4.943862	-0.538708	-1.880848
H	-5.075207	-0.896044	-2.900179
C	-5.828454	-0.962073	-0.884836
H	-6.640487	-1.640033	-1.130862
C	-5.665791	-0.502166	0.417477
H	-6.358144	-0.824139	1.192195
C	-4.615289	0.371723	0.752335
C	-3.050586	0.828727	-2.736567
H	-1.984496	0.941188	-2.407987
C	-3.564490	2.160025	-3.286275
H	-4.595347	2.075974	-3.644905
H	-2.941683	2.512138	-4.113756
H	-3.548335	2.929352	-2.502471

H	3.172329	-3.041022	-0.164899
C	4.623796	-1.926783	-1.341878
H	4.815724	-0.887598	-1.649685
H	4.377740	-2.501414	-2.240109
H	5.556341	-2.327083	-0.928875
C	0.389443	2.717742	-0.837258
C	-0.200335	3.135724	-2.056544
C	-1.078923	4.229303	-2.076937
H	-1.498217	4.564144	-3.022653
C	-1.401960	4.895459	-0.898054
H	-2.067058	5.755527	-0.918653
C	-0.869262	4.452889	0.313161
H	-1.152336	4.973043	1.227215
C	0.033554	3.379688	0.372794
C	0.078576	2.452578	-3.369838
H	0.486856	1.426253	-3.181369
C	1.058037	3.242441	-4.236483
H	0.729041	4.278416	-4.377633
H	1.171766	2.788282	-5.225481
H	2.052950	3.276784	-3.765831
C	0.598529	2.965199	1.699032
H	0.178292	1.946733	1.957675
C	0.362367	3.918639	2.866708
H	0.770018	4.915804	2.665475
H	0.853651	3.540801	3.771855
H	-0.702883	4.027896	3.098431
N	-2.958392	-0.687349	-0.740356
C	-1.640773	-0.885753	-0.627892
B	-0.784056	-0.623129	0.707871
N	-1.202435	-1.426098	-1.790816
C	-3.469641	-0.922780	-2.092310
H	-3.611485	0.039114	-2.610740
C	-2.329777	-1.749505	-2.691933
H	-2.544072	-2.824379	-2.647710
C	-3.799459	-0.103269	0.283821
C	-3.990856	1.291709	0.337789
C	-4.835486	1.818010	1.327967
H	-4.989158	2.894579	1.385182
C	-5.474398	0.973946	2.234310
H	-6.127251	1.393115	3.000222
C	-5.284687	-0.408044	2.167330
H	-5.793684	-1.052537	2.884119
C	-4.444546	-0.974032	1.199476
C	-3.325456	2.231487	-0.624864
H	-2.303428	1.879786	-0.892162
C	-4.162161	2.450362	-1.883428
H	-5.182321	2.769010	-1.639455
C	-4.493641	0.751616	2.205417
H	-4.508999	-0.175815	2.826978
C	-5.610654	1.696114	2.648588
H	-5.632021	2.602511	2.031446
H	-5.484077	2.002313	3.691512
H	-6.596028	1.225284	2.553786
C	0.638438	2.941684	0.015891
C	1.600468	3.299010	0.986836
C	2.901309	3.635757	0.582116
H	3.644900	3.904907	1.326597
C	3.237222	3.640434	-0.769956
H	4.248914	3.891685	-1.078202
C	2.270809	3.344499	-1.734483
H	2.543776	3.394372	-2.786103
C	0.959950	3.013899	-1.365654
C	1.255293	3.366953	2.453142
H	0.420585	2.664947	2.680217
C	0.854968	4.784743	2.860060
H	1.631767	5.513508	2.603958
H	0.665035	4.853109	3.935103
H	-0.066107	5.087098	2.335936
C	-0.108181	2.775841	-2.393627
H	-0.397001	1.687374	-2.368864
C	0.233270	3.187972	-3.821687
H	0.516339	4.244830	-3.880723
H	-0.632378	3.040077	-4.478520
H	1.056290	2.592032	-4.230275
N	2.491360	-1.793749	1.071338
C	1.264355	-1.695768	0.549067
B	0.694540	-0.661738	-0.568637
N	0.523524	-2.681294	1.054014
C	2.590282	-2.836471	2.105458
H	2.623425	-2.375678	3.103979
C	1.296164	-3.629075	1.862746
H	1.470288	-4.553151	1.289354
C	3.585923	-0.883913	0.816600
C	3.810875	0.200255	1.689963
C	4.908513	1.041058	1.446115
H	5.094720	1.886681	2.106799
C	5.747642	0.810064	0.358155
H	6.591708	1.474955	0.173211
C	5.515052	-0.267218	-0.501542
H	6.186725	-0.426670	-1.344009
C	4.435936	-1.133595	-0.291473
C	2.920097	0.488245	2.866292
H	1.868765	0.199052	2.650197
C	3.416287	-0.186299	4.142328

H	-3.711864	3.230112	-2.511638
H	-4.232601	1.535538	-2.489129
C	-4.259356	-2.466103	1.114445
H	-3.224368	-2.697895	0.745580
C	-4.444880	-3.201083	2.439100
H	-5.463248	-3.108474	2.830723
H	-4.229111	-4.269594	2.328830
H	-3.753128	-2.808379	3.201885
C	-0.079557	-2.346804	-1.850469
C	0.018220	-3.465357	-0.990135
C	1.061961	-4.382202	-1.210602
H	1.171774	-5.237707	-0.543314
C	1.956515	-4.216856	-2.263461
H	2.742922	-4.952679	-2.430659
C	1.858916	-3.102563	-3.100038
H	2.574927	-2.984943	-3.911481
C	0.862991	-2.142349	-2.894045
C	-0.912995	-3.712231	0.163734
H	-1.886854	-3.178516	0.059914
C	-1.218546	-5.186549	0.420989
H	-0.329257	-5.735723	0.755544
H	-1.974074	-5.292270	1.209298
H	-1.597457	-5.685893	-0.477278
C	0.767336	-0.913864	-3.752746
H	0.764472	-0.016471	-3.062832
C	1.875704	-0.728256	-4.783009
H	1.888666	-1.534471	-5.524921
H	1.745584	0.216343	-5.325471
H	2.863890	-0.691270	-4.306218
C	-1.336380	-0.978716	3.075034
O	-1.565932	-1.196031	1.743812
C	-1.162772	-2.018343	3.895103
H	1.243848	-0.445293	1.958325
H	-1.031313	-1.861400	4.959514
H	-1.119351	-3.030276	3.509049
C	-1.421114	0.454124	3.507281
H	-2.403819	0.859766	3.224994
H	-1.295165	0.546807	4.588104
H	-0.657752	1.055609	2.993579
H	-4.958846	-2.869293	0.353505
H	-3.140862	3.210412	-0.126389
H	3.499187	2.002439	3.988758
H	2.574101	-1.487919	-0.719029
H	1.703626	2.790660	1.611487
H	-0.872002	2.298016	-3.917508
H	-0.447999	-3.255986	1.078474
H	-0.216639	-0.896030	-4.267092
H	4.467581	0.054393	4.342780
H	2.832525	0.140793	5.011203
H	3.332975	-1.281410	4.080137
C	4.177118	-2.314776	-1.188020
H	3.086647	-2.340429	-1.441847
C	4.960708	-2.329030	-2.496664
H	6.044117	-2.304067	-2.336483
H	4.736024	-3.232344	-3.076325
H	4.686270	-1.462937	-3.124552
C	-0.816613	-2.971895	0.591558
C	-1.008184	-3.752501	-0.570688
C	-2.317762	-4.044480	-0.969365
H	-2.491653	-4.646769	-1.858680
C	-3.405819	-3.547319	-0.245863
H	-4.422024	-3.762132	-0.578607
C	-3.202877	-2.755311	0.882105
H	-4.062673	-2.339528	1.409722
C	-1.905802	-2.441921	1.319384
C	0.138508	-4.255439	-1.407814
H	0.962714	-3.511234	-1.438121
C	0.651139	-5.608602	-0.919434
H	-0.156878	-6.350487	-0.882903
H	1.428651	-6.000594	-1.584602
H	1.077888	-5.538851	0.092641
C	-1.698094	-1.517591	2.484978
H	-1.826311	-0.472410	2.089058
C	-2.642641	-1.776603	3.654823
H	-2.701989	-2.841121	3.903709
H	-2.313045	-1.239864	4.551090
H	-3.665824	-1.428343	3.428247
C	1.305189	-0.426638	-2.955011
O	1.464451	-0.944108	-1.685664
C	0.171136	-0.573974	-3.635085
H	-1.613327	-0.751949	-0.744999
H	0.091800	-0.188565	-4.645101
H	-0.692500	-1.052089	-3.188147
C	2.555953	0.228360	-3.444053
H	3.406933	-0.467395	-3.379434
H	2.448598	0.574128	-4.473338
H	2.794487	1.088037	-2.799975
H	4.380312	-3.246125	-0.619105
H	2.862205	1.596127	3.027077
H	-3.022894	0.069254	-3.541344
H	-3.503233	1.212333	2.420361
H	-1.041480	3.298842	-2.080559
H	2.118061	3.017753	3.064039
H	-0.184550	-4.340191	-2.468851

H	4.679145	1.858285	-0.519677
H	2.769087	2.432034	-2.446742
H	-4.423408	-1.453266	-2.065768
H	-2.097992	-1.475835	-3.722612
H	-0.653141	-1.538395	2.848865
H	-3.796164	3.526482	0.483687
H	-1.349222	4.401467	1.359382
H	3.490179	-3.436136	1.961340
H	0.767195	-3.875512	2.784580

2

E(oniom)= −697.376950454872 a.u.

N	-2.922143	-0.397313	-1.070023
C	-1.702462	-0.857262	-0.566869
B	-0.634884	0.054868	-0.028972
N	-1.776469	-2.247811	-0.675838
C	-3.631295	-1.446545	-1.797298
H	-3.321024	-1.491772	-2.858729
C	-3.142802	-2.666145	-1.031460
H	-3.753494	-2.825833	-0.129980
C	-2.982965	0.959020	-1.575182
C	-3.853985	1.850283	-0.906458
C	-3.965114	3.171428	-1.366577
H	-4.637659	3.860327	-0.860337
C	-3.225557	3.605302	-2.466316
H	-3.326088	4.629524	-2.819037
C	-2.356101	2.727642	-3.114953
H	-1.782963	3.074248	-3.971987
C	-2.218275	1.400507	-2.679917
C	-4.650646	1.433800	0.302244
H	-4.304629	0.446492	0.681644
C	-6.148072	1.376542	0.009241
H	-6.540221	2.351023	-0.302226
H	-6.710759	1.056028	0.892529
H	-6.364394	0.666051	-0.797635
C	-1.252791	0.509446	-3.414839
H	-0.387439	1.113377	-3.774308
C	-1.922482	-0.206367	-4.584772
H	-2.699344	-0.901447	-4.223315
H	-1.197140	-0.791649	-5.159360
H	-2.405173	0.499794	-5.269400
C	-1.103517	-3.108941	0.276308
C	-0.473840	-4.265522	-0.240892
C	0.206077	-5.127356	0.632409
H	0.697909	-6.013135	0.238115
C	0.273489	-4.843722	1.995519
H	0.825793	-5.502745	2.661272
C	-0.372924	-3.718814	2.507399
H	-0.317685	-3.511824	3.574089
C	-1.099696	-2.856415	1.671913
C	-0.503453	-4.619426	-1.706661

H	-0.635448	-3.705496	-2.324216
C	-1.611656	-5.625600	-2.018938
H	-1.537696	-6.514506	-1.381761
H	-1.566699	-5.955337	-3.061394
H	-2.603317	-5.179740	-1.848084
C	-1.850866	-1.700534	2.271041
H	-1.178904	-0.802490	2.255014
C	-2.374981	-1.953786	3.683820
H	-2.966796	-2.874296	3.732804
H	-3.013547	-1.125083	4.009263
H	-1.558486	-2.041060	4.409426
N	2.848557	1.658551	0.166843
C	1.722962	1.155363	0.719622
B	0.951057	-0.167653	0.217095
N	1.363721	1.972324	1.715647
C	3.135403	3.016676	0.650219
H	2.741674	3.763521	-0.060431
C	2.357231	3.025102	1.968466
H	2.992721	2.735904	2.822479
C	3.281801	1.268569	-1.160922
C	2.610861	1.729862	-2.313343
C	3.131470	1.388768	-3.572044
H	2.640193	1.752936	-4.472600
C	4.264789	0.583537	-3.676530
H	4.660666	0.329037	-4.659699
C	4.887370	0.085139	-2.530203
H	5.747984	-0.574206	-2.635300
C	4.406706	0.409790	-1.254906
C	1.345642	2.540852	-2.256007
H	0.735577	2.284026	-1.359830
C	1.620641	4.040391	-2.308637
H	2.196845	4.314545	-3.200303
H	0.682728	4.608808	-2.331022
H	2.192320	4.374756	-1.428844
C	5.000835	-0.179710	-0.005868
H	4.269261	-0.947197	0.363805
C	6.376188	-0.816886	-0.167806
H	7.107949	-0.110680	-0.575773
H	6.757667	-1.167466	0.798702
H	6.342528	-1.689734	-0.832490
C	0.439252	1.586960	2.762561
C	0.830282	0.669069	3.760362
C	-0.056419	0.411090	4.816315
H	0.232085	-0.282278	5.603845
C	-1.307675	1.026712	4.856800
H	-1.985874	0.819279	5.684281
C	-1.704745	1.894346	3.837928

H	-2.698888	2.336304	3.871815
C	-0.845653	2.186866	2.769592
C	2.152680	-0.050073	3.734653
H	2.460654	-0.287317	2.693138
C	3.243566	0.742306	4.451660
H	2.971869	0.942327	5.495427
H	4.191357	0.191722	4.456045
H	3.423584	1.713781	3.965778
C	-1.263449	3.056341	1.621798
H	-1.372449	2.341214	0.724678
C	-2.550905	3.844871	1.806881
H	-2.537328	4.451461	2.718054
H	-2.713742	4.520174	0.957227
H	-3.429972	3.176616	1.849011
C	1.928615	-2.130140	-0.969938
O	1.951880	-1.136184	-0.040658
C	2.509395	-3.302864	-0.689214
H	-1.051616	1.207030	0.085683
H	2.567233	-4.083257	-1.438089
H	2.902955	-3.499770	0.302020
C	1.381628	-1.759705	-2.318297
H	1.951389	-0.905759	-2.714616
H	1.476689	-2.597287	-3.012313
H	0.333383	-1.445408	-2.250585
H	5.048643	0.585271	0.796740
H	0.675326	2.245012	-3.101835
H	-4.441029	2.147005	1.136013
H	-0.788524	-0.237523	-2.721246
H	-2.708302	-1.407741	1.629436
H	0.480664	-5.033550	-2.008321
H	2.042679	-1.051110	4.207029
H	-0.448505	3.748364	1.334494
H	-4.714229	-1.309389	-1.744423
H	-3.136329	-3.587803	-1.626125
H	4.207284	3.174616	0.778152
H	1.879190	3.981858	2.184438

{III + acetone}

E(oniom)= –665.357138842445 a.u.

O	-2.561173	5.144508	-0.816253
H	0.291358	4.250614	-0.400431
N	3.422339	-0.531575	0.524125
B	0.981767	-1.268274	0.452596
C	2.315004	-1.197651	0.994424
N	-2.949047	-1.459505	-0.405338
B	-0.410233	-1.530142	-0.090197
C	2.753832	-1.924947	2.286844
C	2.354044	-1.087910	3.503192
H	2.638528	-1.577874	4.439407
H	1.268678	-0.924803	3.530496
H	2.830099	-0.095983	3.486806
C	2.137386	-3.314447	2.371785
H	2.518549	-3.876615	3.229995
H	2.337800	-3.903849	1.468552
H	1.044031	-3.258613	2.470525
C	4.299962	-1.981081	2.117203
H	4.832855	-2.023946	3.070063
H	4.567328	-2.877117	1.549413
C	4.676008	-0.726375	1.289278
C	5.854662	-0.997394	0.357893
H	6.754648	-1.263108	0.920855
H	6.094558	-0.121354	-0.258290
H	5.639169	-1.821081	-0.340391
C	5.000294	0.476478	2.179473
H	5.888684	0.293829	2.791002
H	4.165075	0.704137	2.863231
H	5.188142	1.378184	1.584028
C	3.272804	0.414632	-0.561499
C	2.829580	1.733027	-0.299215
C	2.354672	2.167781	1.076600
H	2.667780	1.409449	1.842910
C	0.822002	2.213380	1.086520
H	0.432922	2.470674	2.074917
H	0.401714	1.226943	0.820818
H	0.424185	2.936396	0.359500
C	2.761589	2.645129	-1.362274
H	2.444158	3.669025	-1.173783
C	2.962674	3.515067	1.490849
H	2.710119	3.754943	2.528818
H	2.601415	4.339813	0.868099
H	4.054948	3.498222	1.407561

$TS1_3$

E(oniom)= –665.349716831346 a.u.

νmin = –204.7 cm^{-1}

O	1.187580	1.371077	-0.683241
H	-1.540621	2.183234	-0.147476
N	-3.447125	-0.704334	-0.304542
B	-0.846504	-0.798007	-0.379373
C	-2.173838	-1.034711	-0.819380
N	3.262566	-0.815147	0.282704
B	0.674974	-0.638231	-0.018877
C	-2.499721	-1.831973	-2.094742
C	-2.853107	-0.878461	-3.241488
H	-3.246857	-1.414786	-4.107903
H	-1.974553	-0.310319	-3.564953
H	-3.610014	-0.140800	-2.919671
C	-1.362487	-2.760147	-2.511626
H	-1.635662	-3.365392	-3.381386
H	-1.081885	-3.445456	-1.703209
H	-0.461126	-2.190955	-2.774807
C	-3.751516	-2.634063	-1.624189
H	-4.424443	-2.889180	-2.447815
H	-3.421660	-3.576596	-1.177386
C	-4.458817	-1.754847	-0.543565
C	-4.742189	-2.581720	0.718807
H	-5.426509	-3.408796	0.512400
H	-5.193299	-1.964239	1.504807
H	-3.819940	-3.006029	1.134513
C	-5.774785	-1.166931	-1.056183
H	-6.519889	-1.950021	-1.224613
H	-5.634045	-0.632471	-2.003745
H	-6.199616	-0.452277	-0.340420
C	-3.641390	0.371502	0.637554
C	-4.211782	1.577626	0.150686
C	-4.425504	1.830394	-1.331501
H	-4.243324	0.872790	-1.907098
C	-3.393549	2.857881	-1.822583
H	-3.435105	2.968409	-2.910476
H	-2.375353	2.534182	-1.555717
H	-3.556833	3.845728	-1.380998
C	-4.527419	2.599438	1.055787
H	-4.987280	3.516646	0.691453
C	-5.855170	2.296295	-1.632890
H	-6.038530	2.329178	-2.711664
H	-6.053586	3.298252	-1.237711
H	-6.594140	1.620213	-1.187964

C	3.085139	2.255588	-2.661834
H	3.034665	2.976979	-3.475001
C	3.449182	0.935115	-2.923158
H	3.655333	0.626702	-3.947255
C	3.537215	-0.005501	-1.886814
C	3.834397	-1.456219	-2.226126
H	4.139701	-2.004228	-1.292855
C	2.552866	-2.111840	-2.757053
H	2.707081	-3.172511	-2.972325
H	2.195419	-1.629898	-3.672271
H	1.743902	-2.035290	-2.012127
C	4.989175	-1.587736	-3.228660
H	5.276127	-2.636473	-3.358146
H	5.873000	-1.035486	-2.892440
H	4.718677	-1.203712	-4.218506
C	-1.690516	-1.968912	-0.588348
H	-0.232730	5.224585	-1.808547
C	-1.845772	-3.218371	-1.488170
C	-1.596591	-2.831985	-2.946299
H	-1.677329	-3.696843	-3.611716
H	-0.593583	-2.404673	-3.076161
H	-2.316717	-2.074763	-3.293457
C	-0.891643	-4.324418	-1.058756
H	-1.063934	-5.248870	-1.618971
H	-0.989647	-4.556169	0.008881
H	0.155639	-4.030762	-1.218878
C	-3.333277	-3.601869	-1.240697
H	-3.778184	-4.150828	-2.074068
H	-3.395349	-4.240038	-0.354147
C	-4.066957	-2.265956	-0.959263
C	-4.639156	-1.650427	-2.235805
H	-5.446933	-2.261482	-2.648595
H	-3.865810	-1.550641	-3.015336
H	-5.045133	-0.641978	-2.055504
C	-5.183596	-2.439319	0.066881
H	-5.979039	-3.086696	-0.315866
H	-5.640601	-1.476787	0.332707
H	-4.816444	-2.882439	1.003067
C	-3.125245	-0.222181	0.327353
C	-3.137072	1.010754	-0.364504
C	-2.842372	1.121152	-1.850851
H	-2.604653	0.106988	-2.264285
C	-1.607705	2.003143	-2.081736
H	-1.306373	1.988225	-3.133758
H	-0.754648	1.645520	-1.490746
H	-1.798991	3.049605	-1.811817
C	-4.062957	1.674259	-2.599675

C	-4.255272	2.460259	2.417141
H	-4.525358	3.252431	3.111402
C	-3.617697	1.310914	2.878911
H	-3.368578	1.220963	3.935322
C	-3.284679	0.266551	2.001855
C	-2.480814	-0.899582	2.546297
H	-2.340809	-1.670087	1.741705
C	-1.092972	-0.388837	2.959514
H	-0.382965	-1.224270	3.079056
H	-1.117677	0.169756	3.898250
H	-0.668574	0.276518	2.185603
C	-3.194655	-1.578814	3.723027
H	-2.642954	-2.464124	4.055437
H	-4.204626	-1.897808	3.441920
H	-3.292900	-0.911669	4.585719
C	1.944119	-1.216447	0.356427
H	-0.591296	1.583062	1.222069
C	1.964471	-2.632127	0.990425
C	1.789379	-2.533834	2.507641
H	1.823572	-3.518878	2.981366
H	0.818185	-2.073638	2.764393
H	2.572731	-1.911888	2.966210
C	0.889805	-3.533347	0.397339
H	0.982225	-4.563229	0.755818
H	0.936506	-3.554925	-0.698559
H	-0.118941	-3.181971	0.662300
C	3.391609	-3.124003	0.623077
H	3.768822	-3.893206	1.301722
H	3.374758	-3.551099	-0.384519
C	4.273013	-1.852863	0.622181
C	4.913001	-1.603943	1.990412
H	5.595251	-2.413313	2.264609
H	4.149062	-1.530368	2.781476
H	5.487891	-0.664725	2.004361
C	5.377377	-1.938437	-0.431350
H	6.105186	-2.716376	-0.179251
H	5.925604	-0.992123	-0.522849
H	4.979421	-2.172676	-1.427064
C	3.638935	0.465676	-0.278158
C	3.900097	1.556433	0.584395
C	3.668498	1.487697	2.083893
H	3.250041	0.480936	2.349598
C	2.632584	2.534547	2.520634
H	2.435695	2.464828	3.595601
H	1.681346	2.376504	2.000234
H	2.964673	3.556044	2.312791
C	4.990682	1.674644	2.841944

H	-3.878864	1.717356	-3.677465
H	-4.311891	2.691925	-2.273613
H	-4.946770	1.044254	-2.428922
C	-3.395897	2.186964	0.356138
H	-3.443956	3.142768	-0.175580
C	-3.586043	2.153313	1.735810
H	-3.795459	3.072496	2.278599
C	-3.496129	0.941773	2.422597
H	-3.607231	0.928040	3.505562
C	-3.103279	-1.541644	2.533800
H	-3.149579	-2.422588	1.839370
C	-3.260746	-0.257612	1.736932
C	-1.725354	-1.550330	3.208723
H	-1.557717	-2.479887	3.759337
H	-1.606252	-0.717573	3.907966
H	-0.926394	-1.461205	2.453966
C	-4.229470	-1.711650	3.563950
H	-4.191760	-2.704227	4.024173
H	-5.214587	-1.590936	3.100800
H	-4.157759	-0.977262	4.373674
C	-0.180617	5.198965	-0.719527
H	0.447981	6.014081	-0.347403
C	-1.569523	5.264167	-0.126886
C	-1.660081	5.444213	1.372035
H	-1.134653	4.622423	1.874434
H	-2.704702	5.458597	1.684968
H	-1.166861	6.374650	1.673905

I1$_3$
E(oniom)= –665.373515146683 a.u.

O	-1.250784	-0.801623	-1.084278
H	1.391394	-1.601623	-1.825066
N	3.251100	0.723151	0.248093
B	0.849193	0.833722	-0.482174
C	2.207979	1.230215	-0.606013
N	-3.121118	0.840261	0.449687
B	-0.701549	0.385256	-0.359510
C	2.835483	2.321096	-1.519728
C	2.687545	1.974744	-3.006044
H	3.204440	2.697516	-3.642449
H	1.633650	1.958884	-3.302128
H	3.097916	0.978486	-3.217959
C	2.218585	3.700224	-1.266753
H	2.731663	4.483372	-1.831130
H	2.280466	3.958740	-0.199301
H	1.162498	3.721776	-1.554069

H	4.841850	1.600353	3.923508
H	5.447879	2.647958	2.637881
H	5.715882	0.900078	2.547763
C	4.360840	2.762378	0.033131
H	4.589079	3.598607	0.691431
C	4.526387	2.904621	-1.343172
H	4.894842	3.841478	-1.754372
C	4.209710	1.845312	-2.194275
H	4.310004	1.972781	-3.270958
C	3.363785	-0.477061	-2.657925
H	3.219157	-1.439376	-2.099240
C	3.759630	0.620072	-1.683663
C	2.024341	-0.115766	-3.313312
H	1.657790	-0.928404	-3.945812
H	2.096777	0.786187	-3.927207
H	1.262339	0.078973	-2.539574
C	4.451482	-0.713941	-3.716770
H	4.234484	-1.612201	-4.303783
H	5.437335	-0.840134	-3.257152
H	4.525000	0.121700	-4.421120
C	-0.650095	2.384481	0.475337
H	-0.788934	3.359081	0.951733
C	0.551794	2.382926	-0.429025
C	0.949718	3.694256	-1.055087
H	0.087648	4.166304	-1.536876
H	1.755722	3.533549	-1.774341
H	1.297252	4.373737	-0.267929

TS2$_3$
E(oniom)= –665.349379803972 a.u.
νmin = –1087.5 cm^{-1}

O	1.500149	1.358337	0.480346
H	-0.738374	1.217022	1.994495
N	-3.224511	0.765916	0.177838
B	-0.787103	0.627929	-0.634071
C	-2.121641	1.187389	-0.599042
N	2.920982	-0.988392	-0.599711
B	0.759111	0.358661	-0.344539
C	-2.551076	2.483573	-1.318094
C	-1.385487	3.462866	-1.468282
H	-1.702275	4.383033	-1.970698
H	-0.575525	3.026694	-2.067790
H	-0.957783	3.752695	-0.501930
C	-3.139948	2.184433	-2.697514
H	-3.535560	3.084040	-3.176026
H	-3.963178	1.449413	-2.625076
H	-2.384224	1.751982	-3.363210

C	4.343457	2.316431	-1.097970
H	4.919880	1.673094	-1.774798
H	4.796418	3.312429	-1.128145
C	4.366970	1.679653	0.315562
C	4.074454	2.734456	1.394968
H	4.866845	3.483229	1.448719
H	3.968011	2.271613	2.381684
H	3.127863	3.255059	1.181557
C	5.705135	1.006696	0.629134
H	6.510757	1.743216	0.698198
H	5.996536	0.271818	-0.138501
H	5.663614	0.467806	1.583638
C	3.424491	-0.703680	0.399520
C	3.918283	-1.560613	-0.615050
C	4.340580	-1.061997	-1.984562
H	4.080232	0.029839	-2.081687
C	3.594239	-1.799536	-3.106441
H	3.859405	-1.393655	-4.087934
H	2.505977	-1.688948	-2.979543
H	3.823199	-2.869493	-3.121266
C	4.047269	-2.933448	-0.351437
H	4.439622	-3.591818	-1.124789
C	5.860243	-1.200137	-2.154622
H	6.194005	-0.783915	-3.109442
H	6.185573	-2.244209	-2.113694
H	6.385304	-0.660200	-1.347974
C	3.692785	-3.465760	0.886226
H	3.807721	-4.529383	1.077706
C	3.195800	-2.624498	1.881660
H	2.921428	-3.040857	2.849690
C	3.048623	-1.249985	1.655242
C	2.490675	-0.380580	2.768755
H	2.309279	0.656046	2.369918
C	1.143534	-0.925487	3.265434
H	0.722589	-0.279398	4.042147
H	1.232916	-1.930304	3.688135
H	0.419317	-0.971351	2.440897
C	3.507558	-0.277564	3.914756
H	3.151296	0.398371	4.697905
H	4.466175	0.109016	3.549609
H	3.701170	-1.250185	4.377849
C	-1.778588	1.116894	0.364811
H	0.956176	-1.899999	-0.161151
C	-1.553603	2.370476	1.246376
C	-1.029915	1.934428	2.615991
H	-0.867043	2.792875	3.274544
H	-0.065402	1.415063	2.510839

C	-3.626750	3.024569	-0.334264
H	-3.127683	3.600415	0.452510
H	-4.352707	3.689118	-0.809856
C	-4.304164	1.776359	0.295797
C	-5.581696	1.388401	-0.457336
H	-6.358002	2.151144	-0.353956
H	-5.994297	0.443681	-0.083373
H	-5.385405	1.257218	-1.532546
C	-4.658883	2.044002	1.759253
H	-5.403812	2.839389	1.848875
H	-3.776837	2.341222	2.340559
H	-5.065943	1.144696	2.246615
C	-3.434912	-0.645799	0.429020
C	-3.159214	-1.159406	1.720796
C	-2.597622	-0.316827	2.851626
H	-2.381078	0.718995	2.470159
C	-1.271474	-0.910959	3.351052
H	-0.797566	-0.254096	4.086851
H	-0.569463	-1.052518	2.516859
H	-1.409211	-1.887983	3.824779
C	-3.387979	-2.518366	1.989031
H	-3.190479	-2.906312	2.987598
C	-3.613653	-0.207378	3.997794
H	-3.257848	0.472835	4.777287
H	-3.805791	-1.177114	4.468205
H	-4.576868	0.173831	3.629031
C	-3.851892	-3.380612	0.999347
H	-4.018666	-4.431927	1.220670
C	-4.094956	-2.884186	-0.279534
H	-4.443783	-3.561299	-1.058441
C	-3.902362	-1.527744	-0.580560
C	-4.200225	-1.089373	-2.002577
H	-4.151914	0.036981	-2.066941
C	-3.143822	-1.649867	-2.959909
H	-3.324079	-1.326175	-3.988570
H	-3.116753	-2.743551	-2.952602
H	-2.137465	-1.295299	-2.665772
C	-5.613606	-1.510836	-2.433921
H	-5.881522	-1.058842	-3.393956
H	-6.363087	-1.205265	-1.695937
H	-5.698369	-2.596611	-2.551791
C	1.602835	-0.749884	-0.868395
H	-0.994082	1.824989	0.446738
C	1.136484	-1.882050	-1.813764
C	0.314025	-2.902819	-1.029934
H	-0.038636	-3.716587	-1.670057
H	-0.573719	-2.430851	-0.581165

H	-1.721954	1.247457	3.122577	H	0.893922	-3.345092	-0.206998
C	-0.587295	3.349564	0.603135	C	0.337274	-1.316146	-2.978044
H	-0.549939	4.301263	1.140737	H	0.168966	-2.064801	-3.757837
H	-0.848304	3.558404	-0.440467	H	0.834136	-0.452466	-3.434478
H	0.438441	2.936437	0.595655	H	-0.656846	-0.959336	-2.639643
C	-2.980811	2.979123	1.354533	C	2.479401	-2.492030	-2.317635
H	-3.130290	3.565562	2.264241	H	2.399915	-3.551991	-2.571345
H	-3.154575	3.644664	0.503133	H	2.804761	-1.962938	-3.218698
C	-3.944934	1.778570	1.264802	C	3.496430	-2.230449	-1.185913
C	-4.267167	1.200894	2.644389	C	3.511376	-3.374742	-0.170832
H	-4.840180	1.910540	3.248898	H	3.853634	-4.307941	-0.628173
H	-3.347056	0.961715	3.203524	H	2.504745	-3.559976	0.239079
H	-4.857092	0.274899	2.573872	H	4.179106	-3.159755	0.678570
C	-5.246774	2.143098	0.555720	C	4.909431	-2.000562	-1.713026
H	-5.814160	2.883669	1.129315	H	5.309032	-2.907672	-2.174217
H	-5.894391	1.268406	0.415521	H	5.600829	-1.711654	-0.910443
H	-5.064709	2.568746	-0.441332	H	4.950586	-1.206499	-2.464077
C	-3.702731	-0.342932	-0.153361	C	3.715648	-0.041265	0.159653
C	-3.726733	-1.568842	0.552778	C	3.861724	-0.177126	1.558397
C	-3.026824	-1.764251	1.886965	C	3.096961	-1.209238	2.367968
H	-2.530616	-0.806152	2.195432	H	2.388699	-1.761659	1.695706
C	-1.921127	-2.822210	1.764098	C	2.247557	-0.515191	3.442455
H	-1.399562	-2.955220	2.718835	H	1.615601	-1.237686	3.969324
H	-1.171218	-2.516223	1.024920	H	1.588054	0.234509	2.984865
H	-2.311293	-3.799151	1.464601	H	2.860158	-0.005493	4.191977
C	-4.039122	-2.150074	2.974964	C	4.060870	-2.225683	2.995514
H	-3.558095	-2.214242	3.956385	H	3.520491	-2.963539	3.597117
H	-4.508176	-3.119150	2.776710	H	4.797910	-1.746140	3.647346
H	-4.842344	-1.401829	3.042683	H	4.612801	-2.769092	2.212650
C	-4.401570	-2.662098	-0.013213	C	4.710831	0.710905	2.238632
H	-4.449425	-3.602222	0.534774	H	4.852285	0.597140	3.312446
C	-5.012440	-2.560956	-1.261540	C	5.364139	1.739436	1.563225
H	-5.550001	-3.410857	-1.676907	H	6.023504	2.414905	2.103909
C	-4.923986	-1.370003	-1.983516	C	5.161421	1.909362	0.193259
H	-5.374303	-1.307422	-2.973517	H	5.644223	2.737631	-0.323511
C	-4.122036	0.993846	-2.309440	C	4.095957	1.302341	-2.001609
H	-3.829130	1.862364	-1.659026	H	3.542665	0.439857	-2.454888
C	-4.264972	-0.252901	-1.452431	C	4.339803	1.031768	-0.527095
C	-2.996807	0.776808	-3.329618	C	3.181620	2.536352	-2.128526
H	-2.841415	1.669503	-3.943438	H	2.909919	2.717608	-3.173344
H	-3.205561	-0.059584	-4.001999	H	3.658337	3.438079	-1.737994
H	-2.047855	0.564943	-2.812510	H	2.250018	2.372931	-1.565984
C	-5.438927	1.366115	-3.005229	C	5.405948	1.476974	-2.788031
H	-5.361437	2.347071	-3.486330	H	5.426116	0.779408	-3.679072
H	-6.270361	1.406104	-2.293447	H	6.307585	1.244459	-2.150642
H	-5.709678	0.646368	-3.785379	H	5.519179	2.531713	-3.177345
C	0.703170	-2.192055	-1.193871	C	-0.381780	2.189370	1.629136

H	0.898312	-3.256710	-1.350546
C	-0.711540	-1.866065	-1.483053
C	-1.568241	-2.822893	-2.242403
H	-1.139501	-3.023358	-3.231077
H	-2.579600	-2.419451	-2.355985
H	-1.621522	-3.783849	-1.714788

$I2_3$

E(oniom)= –665.387825564089 a.u.

O	-1.472519	-1.506093	-0.056130
H	-0.277515	-3.444482	2.404181
N	3.380302	0.144176	-0.682035
B	0.865599	-0.504045	-0.587465
C	2.184590	0.038701	-1.217337
N	-2.865968	1.061115	-0.047927
B	-0.674007	-0.269581	-0.125671
C	2.343286	0.193579	-2.759997
C	1.483822	-0.844627	-3.457422
H	1.563235	-0.776440	-4.547586
H	0.416368	-0.714563	-3.198006
H	1.741607	-1.871466	-3.170685
C	1.947077	1.600927	-3.188093
H	2.016130	1.724457	-4.274608
H	2.579028	2.372626	-2.724643
H	0.897809	1.816783	-2.905063
C	3.866355	-0.056416	-2.963028
H	4.045654	-1.125622	-3.106349
H	4.273189	0.463891	-3.832421
C	4.527740	0.403820	-1.647365
C	4.870517	1.885769	-1.664001
H	5.607395	2.112980	-2.443990
H	5.307215	2.219887	-0.706034
H	3.988143	2.516457	-1.863909
C	5.749760	-0.416099	-1.274826
H	6.588405	-0.206363	-1.950718
H	5.564418	-1.498980	-1.322991
H	6.096715	-0.201339	-0.253735
C	3.582269	-0.173894	0.728673
C	3.738097	-1.535538	1.096235
C	3.741035	-2.685456	0.098352
H	3.813645	-2.286286	-0.945708
C	2.437901	-3.475969	0.206128
H	2.424625	-4.344434	-0.453980
H	1.561023	-2.823173	-0.068065
H	2.228743	-3.819392	1.224693
C	3.896021	-1.849053	2.453373
H	3.978467	-2.893888	2.755658

H	-0.771940	3.036874	2.187254
C	0.950355	2.278243	1.212164
C	1.807713	3.493282	1.393751
H	1.363877	4.209494	2.086204
H	1.986635	3.975989	0.425293
H	2.788588	3.179337	1.774613

$TS3_3$

E(oniom)= –665.366443036808 a.u.

νmin = –540.0 cm^{-1}

O	-1.322435	1.432506	0.379660
H	-0.074006	3.587093	-1.868497
N	3.445860	-0.280964	0.610114
B	0.839051	-0.097884	0.560110
C	2.195721	-0.272964	1.194786
N	-2.938463	-1.044388	-0.124446
B	-0.684126	0.144391	0.170609
C	2.398890	-0.739580	2.667048
C	1.477081	0.031620	3.602415
H	1.614167	-0.275608	4.645693
H	0.418178	-0.141373	3.353297
H	1.642424	1.114021	3.561245
C	2.131723	-2.237722	2.782115
H	2.312509	-2.604199	3.797220
H	2.773883	-2.816219	2.098608
H	1.085271	-2.475438	2.524066
C	3.895145	-0.402894	2.905075
H	3.981981	0.642712	3.216850
H	4.355156	-1.017977	3.682062
C	4.581498	-0.582674	1.527481
C	5.071093	-2.016767	1.321084
H	5.836838	-2.289525	2.052518
H	5.505588	-2.152091	0.316280
H	4.247003	-2.742543	1.415305
C	5.762051	0.368163	1.351994
H	6.587809	0.105280	2.021240
H	5.488670	1.413934	1.563537
H	6.150965	0.345536	0.325335
C	3.585808	0.305411	-0.708707
C	3.678502	1.714203	-0.862069
C	3.732239	2.677278	0.314788
H	4.079971	2.122425	1.230338
C	2.345172	3.245510	0.611986
H	2.368880	4.030043	1.370051
H	1.657202	2.428085	0.986665
H	1.857621	3.649207	-0.285690
C	3.722201	2.248918	-2.156407

C	4.960193	-3.596324	0.319690
H	5.065476	-4.311812	-0.502833
H	4.863578	-4.185185	1.239868
H	5.889904	-3.024339	0.395429
C	3.935792	-0.848048	3.423361
H	4.060892	-1.110859	4.473269
C	3.806273	0.486447	3.047104
H	3.839617	1.264607	3.809858
C	3.610556	0.845841	1.703345
C	3.435852	2.320587	1.384006
H	3.062458	2.436296	0.333354
C	2.391202	2.957232	2.307868
H	2.117238	3.960043	1.964768
H	2.742237	3.046179	3.340594
H	1.464775	2.351625	2.327459
C	4.782523	3.047169	1.516587
H	4.667733	4.123976	1.352205
H	5.512260	2.669081	0.783991
H	5.219878	2.915156	2.512660
C	-1.485759	0.986694	-0.052621
H	1.115950	-1.709147	-0.738967
C	-1.022195	2.448522	0.092706
C	-0.885221	2.768265	1.585778
H	-0.631577	3.818407	1.750926
H	-0.096059	2.146291	2.043515
H	-1.814436	2.556984	2.136952
C	0.284509	2.752780	-0.623685
H	0.530594	3.817000	-0.584283
H	0.225496	2.447865	-1.680596
H	1.114678	2.193375	-0.169117
C	-2.197804	3.246550	-0.533628
H	-2.265286	4.277373	-0.176783
H	-2.067872	3.279682	-1.620062
C	-3.458520	2.419104	-0.192660
C	-4.123243	2.905110	1.096755
H	-4.476419	3.935219	1.000705
H	-3.423283	2.869155	1.947428
H	-4.989079	2.279823	1.367394
C	-4.484213	2.473558	-1.325215
H	-4.894696	3.481626	-1.439257
H	-5.326472	1.793783	-1.140258
H	-4.047874	2.184004	-2.289417
C	-3.711700	-0.118815	-0.054905
C	-4.257510	-0.594955	1.161637
C	-3.872047	-0.033475	2.520255
H	-3.119766	0.786819	2.384968
C	-3.208764	-1.112818	3.383696
H	3.744750	3.330255	-2.292177
C	4.747771	3.806200	0.078407
H	4.910688	4.385381	0.993044
H	4.404490	4.508944	-0.690018
H	5.716005	3.411138	-0.247639
C	3.730513	1.420090	-3.278801
H	3.772275	1.854493	-4.275842
C	3.678971	0.037864	-3.119156
H	3.694599	-0.606063	-3.997817
C	3.586598	-0.538363	-1.842057
C	3.487571	-2.050331	-1.738657
H	3.185900	-2.330749	-0.695055
C	2.415483	-2.599707	-2.688164
H	2.217830	-3.657651	-2.490465
H	2.705050	-2.509132	-3.739640
H	1.462796	-2.054068	-2.561330
C	4.855700	-2.682642	-2.035176
H	4.795049	-3.775344	-2.038435
H	5.592289	-2.387693	-1.270800
H	5.247250	-2.370882	-3.009008
C	-1.573188	-1.032920	-0.122905
H	1.598168	1.237982	1.331539
C	-1.150311	-2.473239	-0.482258
C	-0.968280	-2.563656	-2.000063
H	-0.752466	-3.587893	-2.317576
H	-0.128457	-1.926008	-2.330360
H	-1.862608	-2.225627	-2.544114
C	0.120783	-2.922016	0.220423
H	0.304809	-3.991303	0.078150
H	0.081606	-2.725943	1.304417
H	1.002543	-2.387674	-0.169469
C	-2.374513	-3.307917	-0.016361
H	-2.474892	-4.261592	-0.539660
H	-2.273019	-3.525043	1.051504
C	-3.593061	-2.383248	-0.237140
C	-4.229369	-2.597930	-1.609438
H	-4.619916	-3.614411	-1.713808
H	-3.501154	-2.437374	-2.421613
H	-5.066236	-1.902388	-1.784475
C	-4.654720	-2.579039	0.842115
H	-5.118242	-3.568273	0.765897
H	-5.457199	-1.833584	0.761598
H	-4.237839	-2.489911	1.853439
C	-3.718958	0.163630	0.083321
C	-4.208753	0.885625	-1.030876
C	-3.853572	0.542014	-2.468130
H	-3.146670	-0.327796	-2.480914

H	-2.899825	-0.712769	4.352963
H	-2.303366	-1.501308	2.887106
H	-3.866334	-1.966522	3.566584
C	-5.104885	0.549910	3.230623
H	-4.823566	1.036206	4.169230
H	-5.840074	-0.224929	3.470402
H	-5.605347	1.295685	2.596689
C	-5.179029	-1.653345	1.122376
H	-5.615143	-2.015513	2.051955
C	-5.535150	-2.252684	-0.084066
H	-6.258906	-3.063782	-0.098187
C	-4.946322	-1.820013	-1.272122
H	-5.198350	-2.316604	-2.207438
C	-3.367373	-0.390214	-2.597999
H	-2.770014	0.548783	-2.466494
C	-4.026684	-0.761727	-1.279652
C	-2.396103	-1.504017	-3.011048
H	-1.815670	-1.223452	-3.891400
H	-2.911998	-2.443322	-3.225227
H	-1.691257	-1.706840	-2.180254
C	-4.410721	-0.136908	-3.698771
H	-3.938129	0.272667	-4.596422
H	-5.181178	0.566604	-3.368127
H	-4.920814	-1.060428	-3.993584
C	-0.360998	-2.555679	1.790192
H	0.269338	-1.703587	2.018258
C	-1.247107	-2.528707	0.774984
C	-2.134635	-3.697392	0.446129
H	-1.983376	-4.529362	1.136707
H	-1.948526	-4.035102	-0.578811
H	-3.186172	-3.380620	0.492194

I3_3
E(oniom)= –665.387859520299 a.u.

O	-1.243190	1.557716	0.458379
H	-0.861312	3.461678	-2.277099
N	3.386775	-0.360960	0.548638
B	0.784419	-0.141366	0.525282
C	2.137387	-0.082052	1.269906
N	-2.912989	-1.012972	-0.321894
B	-0.694360	0.265691	0.111833
C	2.353608	-0.771489	2.658124
C	1.496042	-0.097574	3.726394
H	1.680449	-0.528894	4.716456
H	0.416434	-0.212440	3.518861
H	1.687320	0.978254	3.803883

C	-3.134373	1.714010	-3.146043
H	-2.837874	1.459910	-4.167687
H	-2.215421	1.974719	-2.595899
H	-3.751214	2.615339	-3.191796
C	-5.117565	0.153572	-3.252384
H	-4.866816	-0.164583	-4.268897
H	-5.818768	0.990265	-3.335163
H	-5.647036	-0.673869	-2.758034
C	-5.048402	1.988350	-0.806039
H	-5.442040	2.541277	-1.657795
C	-5.377588	2.390362	0.486714
H	-6.037699	3.240515	0.642855
C	-4.846199	1.707670	1.580595
H	-5.079133	2.045721	2.589013
C	-3.406996	-0.055152	2.636040
H	-2.864519	-0.990077	2.340600
C	-4.008303	0.597673	1.401850
C	-2.381422	0.897282	3.265766
H	-1.830918	0.412797	4.074746
H	-2.849345	1.800659	3.666137
H	-1.654968	1.223956	2.498723
C	-4.493102	-0.440846	3.652953
H	-4.070092	-1.035919	4.468256
H	-5.292872	-1.024427	3.186008
H	-4.958856	0.441813	4.104864
C	-0.204622	2.637414	-1.363800
H	0.363917	1.781325	-1.711962
C	-1.057106	2.544210	-0.327654
C	-1.859183	3.706544	0.184450
H	-1.666080	4.615024	-0.388741
H	-1.630457	3.886211	1.239739
H	-2.929788	3.465042	0.124270

TS4_3
E(oniom)= –665.366048895769 a.u.
νmin = –564.8 cm^{-1}

O	-1.226004	-1.306003	-0.401274
H	0.420814	-3.545231	1.479402
N	3.434310	0.270848	-0.660076
B	0.824101	0.350458	-0.409453
C	2.160310	0.583121	-1.060322
N	-3.068404	0.944754	0.120745
B	-0.702378	0.018730	-0.113980
C	2.262351	0.981775	-2.575566
C	1.422616	0.008723	-3.389199
H	1.528041	0.175076	-4.465349
H	0.352837	0.093753	-3.141570

C	2.062379	-2.269493	2.629316
H	2.275507	-2.738741	3.595129
H	2.682396	-2.778164	1.871959
H	1.008525	-2.473288	2.388193
C	3.863249	-0.491013	2.875837
H	3.982844	0.537132	3.235789
H	4.319167	-1.146972	3.622498
C	4.525880	-0.631403	1.478267
C	5.054821	-2.052450	1.263369
H	5.800244	-2.320783	2.016120
H	5.524729	-2.159012	0.271811
H	4.239737	-2.791122	1.314026
C	5.697341	0.343000	1.331357
H	6.503030	0.101432	2.031654
H	5.411735	1.388213	1.511704
H	6.121471	0.306694	0.319140
C	3.614291	0.376384	-0.680913
C	3.661034	1.791664	-0.760500
C	3.397841	2.720825	0.414772
H	3.194187	2.116100	1.341013
C	2.151336	3.571805	0.145050
H	1.843596	4.128152	1.034448
H	1.298979	2.947542	-0.168587
H	2.310892	4.297164	-0.659553
C	3.940565	2.398109	-1.994882
H	3.974242	3.485450	-2.061370
C	4.617080	3.607587	0.707142
H	4.417691	4.289046	1.540325
H	4.895240	4.221750	-0.155940
H	5.490666	2.994162	0.970942
C	4.170548	1.636601	-3.138763
H	4.398087	2.123931	-4.084145
C	4.091964	0.247468	-3.065980
H	4.252357	-0.346625	-3.964689
C	3.796933	-0.396561	-1.855536
C	3.661780	-1.909982	-1.871415
H	3.294852	-2.259065	-0.869806
C	2.629324	-2.345483	-2.921601
H	2.392714	-3.408955	-2.824934
H	2.985853	-2.179541	-3.943349
H	1.690223	-1.779760	-2.809366
C	5.023874	-2.567535	-2.136579
H	4.932130	-3.655212	-2.211005
H	5.723352	-2.343074	-1.314808
H	5.480103	-2.209668	-3.064913
C	-1.560576	-0.881306	-0.322311
H	2.036340	1.018433	1.508442
H	1.697644	-1.034609	-3.182046
C	1.786603	2.414673	-2.796048
H	1.868851	2.700918	-3.851675
H	2.379931	3.135837	-2.218135
H	0.727634	2.539862	-2.519251
C	3.785392	0.834677	-2.876926
H	3.986506	-0.142819	-3.323345
H	4.155410	1.594285	-3.569996
C	4.479899	0.910322	-1.498312
C	4.737731	2.368475	-1.099717
H	5.465901	2.839060	-1.768131
H	5.144845	2.439485	-0.077492
H	3.824147	2.975249	-1.138904
C	5.787419	0.131858	-1.456654
H	6.543026	0.585574	-2.106347
H	5.653233	-0.909830	-1.784793
H	6.204383	0.089590	-0.441780
C	3.669373	-0.293974	0.654264
C	4.026903	-1.665354	0.721288
C	4.105612	-2.549155	-0.513634
H	4.124774	-1.906223	-1.434123
C	2.857676	-3.433408	-0.591301
H	2.862301	-4.059238	-1.486941
H	1.944277	-2.815813	-0.615004
H	2.761462	-4.090173	0.279620
C	4.280309	-2.237998	1.974847
H	4.540085	-3.294228	2.040279
C	5.388238	-3.395602	-0.519454
H	5.515588	-3.902632	-1.481392
H	5.366938	-4.173388	0.252156
H	6.276077	-2.781021	-0.339842
C	4.190367	-1.478328	3.141284
H	4.399432	-1.935435	4.106599
C	3.816607	-0.138451	3.068554
H	3.732808	0.445085	3.985030
C	3.531081	0.469199	1.834992
C	3.080042	1.919801	1.846331
H	2.664791	2.196618	0.825422
C	1.944982	2.141713	2.853054
H	1.640959	3.193466	2.879467
H	2.231253	1.860439	3.872431
H	1.055636	1.541401	2.589914
C	4.265170	2.837110	2.175119
H	3.973610	3.891522	2.153467
H	5.086196	2.697317	1.453239
H	4.672122	2.628285	3.170999
C	-1.710946	1.103521	0.157157

C	-0.995102	-2.222825	-0.804349
C	-0.808567	-2.196156	-2.321890
H	-0.560986	-3.187134	-2.714462
H	0.005581	-1.510151	-2.610517
H	-1.717219	-1.849786	-2.838100
C	0.339408	-2.497663	-0.123770
H	0.785518	-3.441571	-0.460230
H	0.240584	-2.560892	0.973488
H	1.105408	-1.726444	-0.394533
C	-2.107933	-3.216099	-0.379313
H	-2.116658	-4.141533	-0.959180
H	-1.972175	-3.481420	0.673599
C	-3.422278	-2.408194	-0.537782
C	-4.023807	-2.582697	-1.930132
H	-4.236077	-3.634559	-2.143965
H	-3.343327	-2.215230	-2.715397
H	-4.971335	-2.028414	-2.037304
C	-4.463161	-2.789968	0.509693
H	-4.772737	-3.834287	0.396715
H	-5.365915	-2.169239	0.426725
H	-4.097617	-2.664923	1.540708
C	-3.767295	0.045502	0.187696
C	-4.526168	0.830988	-0.709844
C	-4.415146	0.710137	-2.220407
H	-3.593068	-0.007873	-2.476024
C	-4.044361	2.058727	-2.850312
H	-3.880255	1.958957	-3.927558
H	-3.109686	2.443705	-2.414329
H	-4.816261	2.818201	-2.700266
C	-5.731676	0.180813	-2.810479
H	-5.672001	0.105375	-3.900843
H	-6.577661	0.833757	-2.573036
H	-5.962956	-0.819882	-2.414348
C	-5.412225	1.787742	-0.188070
H	-6.013234	2.386414	-0.871335
C	-5.526903	1.988726	1.185421
H	-6.223886	2.728556	1.573081
C	-4.734942	1.247757	2.062602
H	-4.804094	1.430764	3.133865
C	-2.956962	-0.437228	2.594533
H	-2.289727	-1.166631	2.068068
C	-3.841442	0.278824	1.585278
C	-2.050191	0.583193	3.295153
H	-1.180927	0.094212	3.755804
H	-2.575209	1.151235	4.065803
H	-1.657547	1.307909	2.556775
C	-3.799197	-1.229501	3.605627

H	1.628422	1.872592	-0.261060
C	-1.481100	2.591622	0.506926
C	-1.282254	2.703011	2.019930
H	-1.012365	3.717528	2.323843
H	-0.485462	2.018794	2.359078
H	-2.192947	2.421528	2.572024
C	-0.310440	3.223845	-0.232186
H	-0.229234	4.293856	-0.020353
H	-0.401260	3.098329	-1.321712
H	0.664964	2.751614	0.060547
C	-2.812035	3.260897	0.064653
H	-3.034689	4.179365	0.612782
H	-2.750266	3.518473	-0.997299
C	-3.895120	2.178314	0.263089
C	-4.549355	2.275885	1.641294
H	-5.113157	3.206738	1.751377
H	-3.797651	2.245255	2.447701
H	-5.248381	1.443168	1.820674
C	-4.977379	2.257068	-0.810525
H	-5.558724	3.180490	-0.720813
H	-5.681393	1.417130	-0.737686
H	-4.555691	2.230712	-1.823840
C	-3.693706	-0.354790	-0.056756
C	-4.038856	-1.134527	1.072750
C	-3.646485	-0.760599	2.492580
H	-3.067130	0.198524	2.480602
C	-2.731284	-1.830943	3.098485
H	-2.402246	-1.550914	4.102899
H	-1.826542	-1.961375	2.481940
H	-3.216698	-2.808284	3.166088
C	-4.899139	-0.559328	3.360951
H	-4.631223	-0.202506	4.359960
H	-5.460987	-1.490654	3.486700
H	-5.577647	0.176495	2.906703
C	-4.755588	-2.326010	0.877989
H	-5.038572	-2.925554	1.741984
C	-5.101049	-2.756822	-0.401301
H	-5.667736	-3.675609	-0.533563
C	-4.702535	-2.015073	-1.513289
H	-4.941167	-2.375504	-2.512553
C	-3.512422	-0.099715	-2.616559
H	-3.097504	0.903666	-2.338597
C	-3.989269	-0.817278	-1.364236
C	-2.379311	-0.909298	-3.260800
H	-1.921856	-0.367354	-4.090924
H	-2.724953	-1.876608	-3.635156
H	-1.594782	-1.118123	-2.508537

H	-3.162932	-1.704815	4.358719
H	-4.373617	-2.018099	3.099396
H	-4.512824	-0.591763	4.137347
C	-0.761650	2.588204	-1.644151
H	-0.190868	1.742588	-2.013335
C	-1.321861	2.575682	-0.423268
C	-2.119024	3.714718	0.143450
H	-2.170919	4.554524	-0.551693
H	-1.678829	4.051587	1.087260
H	-3.138896	3.370525	0.366002

I4$_3$
E(oniom)= −665.415088693348 a.u.

O	-1.280677	1.472314	-0.163009
H	-2.198040	2.934495	-3.037434
N	3.313391	0.878851	0.099926
B	1.026875	0.137376	-0.714518
C	2.061974	1.190780	-0.260965
N	-2.785510	-1.107902	-0.297105
B	-0.577365	0.256217	-0.567895
C	1.922875	2.723848	-0.084573
C	1.313365	3.349176	-1.326554
H	0.982415	4.376255	-1.154368
H	0.436628	2.770334	-1.666590
H	2.004714	3.352962	-2.175641
C	1.094718	2.992510	1.167547
H	0.862311	4.053847	1.283343
H	1.607279	2.654465	2.076848
H	0.127642	2.444957	1.110114
C	3.400390	3.205577	0.086079
H	3.807380	3.493303	-0.887879
H	3.489684	4.075536	0.740366
C	4.175863	1.986502	0.623977
C	4.215742	1.975907	2.149377
H	4.796190	2.820577	2.535651
H	4.675966	1.056245	2.545757
H	3.203584	2.052668	2.578995
C	5.588071	1.887473	0.062161
H	6.201349	2.738312	0.378665
H	5.592773	1.869617	-1.038312
H	6.097515	0.973176	0.394071
C	3.763137	-0.504333	0.021823
C	4.374402	-0.950124	-1.177004
C	4.467235	-0.066949	-2.410943
H	4.360067	1.010368	-2.111456
C	3.309472	-0.409504	-3.355154

C	-4.661546	0.125269	-3.612141
H	-4.342522	0.771167	-4.435989
H	-5.526794	0.591143	-3.130232
H	-5.005648	-0.816070	-4.054749
C	0.148561	-2.563886	1.109383
H	0.636904	-1.697044	1.541186
C	-0.780198	-2.452859	0.143741
C	-1.479276	-3.636761	-0.461191
H	-1.137435	-4.575744	-0.022310
H	-1.316432	-3.659120	-1.543480
H	-2.562317	-3.539736	-0.301740

TS5$_3$
E(oniom)= −665.377108862566 a.u.
νmin = −403.9 cm^{-1}

O	1.392934	-0.702681	-1.493437
H	-0.746988	-2.536701	-3.310797
N	-3.122333	0.271159	0.676464
B	-0.665198	-0.263392	0.337622
C	-1.893875	-0.339277	1.065250
N	2.887922	0.752635	0.660130
B	0.734538	-0.020272	-0.449760
C	-2.141115	-1.019827	2.439825
C	-1.824864	-2.515640	2.393334
H	-2.119047	-3.027039	3.310287
H	-0.745661	-2.684977	2.236969
H	-2.336473	-2.993575	1.545270
C	-1.309224	-0.352150	3.541747
H	-1.523672	-0.779988	4.524208
H	-1.522751	0.725006	3.584959
H	-0.238996	-0.475183	3.345453
C	-3.669532	-0.798394	2.691000
H	-4.231906	-1.678149	2.358551
H	-3.908704	-0.639279	3.746819
C	-4.062812	0.402949	1.794483
C	-3.832306	1.732216	2.535346
H	-4.464197	1.808686	3.422171
H	-4.053018	2.587750	1.881242
H	-2.785172	1.822607	2.854554
C	-5.524152	0.335449	1.346520
H	-6.205031	0.429609	2.196681
H	-5.759968	-0.615023	0.838525
H	-5.762517	1.135598	0.635243
C	-3.524930	0.223999	-0.709275
C	-3.955656	-0.978954	-1.326003
C	-4.017904	-2.303520	-0.585235
H	-3.581484	-2.181007	0.444096

H	3.306968	0.237259	-4.236900
H	2.337673	-0.271971	-2.845202
H	3.350227	-1.447232	-3.697933
C	4.846973	-2.267116	-1.242305
H	5.314810	-2.625986	-2.158464
C	5.824682	-0.202888	-3.115259
H	5.925521	0.542560	-3.911437
H	5.947555	-1.186875	-3.581579
H	6.655182	-0.063953	-2.415600
C	4.714780	-3.130335	-0.153692
H	5.099624	-4.146524	-0.215924
C	4.073530	-2.696120	1.004255
H	3.947842	-3.382907	1.840102
C	3.570337	-1.388837	1.106013
C	2.808035	-1.007300	2.363272
H	2.389111	0.026708	2.247001
C	1.615942	-1.948229	2.579690
H	0.911348	-1.520173	3.306146
H	1.918930	-2.934989	2.937357
H	1.055339	-2.088744	1.635058
C	3.742613	-1.020627	3.581879
H	3.206912	-0.732806	4.492075
H	4.576345	-0.315560	3.443075
H	4.176295	-2.010830	3.755678
C	-1.483548	-1.014625	-0.637364
H	1.584924	-0.917220	-1.004837
C	-1.062545	-2.430929	-1.133654
C	-1.148811	-2.435276	-2.659774
H	-0.882776	-3.411579	-3.075868
H	-0.450153	-1.696259	-3.082582
H	-2.152677	-2.175375	-3.022979
C	0.314087	-2.860579	-0.673650
H	0.565435	-3.870984	-1.000473
H	0.432909	-2.805564	0.420650
H	1.099890	-2.162516	-1.079136
C	-2.130215	-3.347998	-0.474460
H	-2.290693	-4.280144	-1.020971
H	-1.797650	-3.613002	0.534289
C	-3.400351	-2.488491	-0.384962
C	-4.249764	-2.658645	-1.639424
H	-4.408936	-3.719468	-1.865765
H	-3.769008	-2.209817	-2.524544
H	-5.249026	-2.208299	-1.532685
C	-4.230327	-2.788183	0.856563
H	-4.592000	-3.822663	0.846287
H	-5.110381	-2.134700	0.930599
H	-3.662035	-2.649029	1.792958
C	-3.183777	-3.379772	-1.291162
H	-3.244909	-4.336670	-0.765475
H	-2.124908	-3.081582	-1.317084
H	-3.503250	-3.545388	-2.323720
C	-4.360579	-0.949180	-2.667803
H	-4.692637	-1.868771	-3.147419
C	-5.479962	-2.750266	-0.427046
H	-5.550710	-3.674014	0.153976
H	-5.959924	-2.928362	-1.394065
H	-6.062997	-1.974346	0.095874
C	-4.348970	0.239826	-3.396718
H	-4.679584	0.247733	-4.432049
C	-3.905190	1.415971	-2.794488
H	-3.892677	2.340260	-3.369152
C	-3.467838	1.422451	-1.462287
C	-2.936733	2.714471	-0.869643
H	-2.336565	2.465950	0.057141
C	-1.985366	3.430385	-1.841957
H	-1.463664	4.253842	-1.346312
H	-2.520802	3.851173	-2.700006
H	-1.235170	2.734931	-2.236714
C	-4.097395	3.629164	-0.457168
H	-3.745947	4.629067	-0.188326
H	-4.616829	3.208732	0.419343
H	-4.833829	3.738337	-1.259655
C	1.634221	0.998227	0.338656
H	-0.609481	0.897342	-0.819784
C	1.266694	2.371724	0.951209
C	1.520977	3.398376	-0.155976
H	1.272348	4.411500	0.182906
H	0.893526	3.198397	-1.036426
H	2.568125	3.412201	-0.490430
C	-0.148854	2.462482	1.463571
H	-0.341920	3.408482	1.977877
H	-0.399055	1.640311	2.156103
H	-0.906277	2.372369	0.652950
C	2.304250	2.504394	2.103261
H	2.555793	3.542034	2.329570
H	1.882517	2.065181	3.011493
C	3.539496	1.692695	1.657355
C	4.591013	2.552266	0.972108
H	5.029305	3.275272	1.671012
H	4.169011	3.128566	0.131262
H	5.420540	1.950336	0.572210
C	4.159719	0.894652	2.791217
H	4.611405	1.556294	3.540857
H	4.956379	0.223271	2.437173

C	-3.481852	-0.050460	0.427843
C	-4.551670	0.671013	-0.150093
C	-5.029360	0.511442	-1.584487
H	-4.297756	-0.119673	-2.149957
C	-5.116963	1.862749	-2.307300
H	-5.323073	1.722724	-3.373341
H	-4.166649	2.409741	-2.233171
H	-5.900892	2.506747	-1.898973
C	-6.412900	-0.161800	-1.601387
H	-6.797437	-0.241748	-2.623757
H	-7.148184	0.404459	-1.020074
H	-6.369312	-1.176387	-1.177676
C	-5.240946	1.605542	0.643645
H	-6.072282	2.160759	0.208637
C	-4.881263	1.840459	1.966986
H	-5.429168	2.566946	2.563822
C	-3.810774	1.141478	2.523350
H	-3.520345	1.336932	3.555282
C	-1.941868	-0.513997	2.463394
H	-1.421840	-1.208953	1.755217
C	-3.099421	0.194103	1.775053
C	-0.899702	0.513243	2.918932
H	0.007195	0.015895	3.286279
H	-1.270997	1.172310	3.706300
H	-0.600329	1.150008	2.060053
C	-2.459055	-1.365295	3.633124
H	-1.636812	-1.889477	4.130683
H	-3.175781	-2.120315	3.275578
H	-2.965944	-0.760374	4.391854
C	-1.779196	2.171697	-2.391641
H	-1.346250	1.287689	-2.846087
C	-1.793067	2.337040	-1.054226
C	-2.365498	3.549565	-0.375613
H	-2.800693	4.247812	-1.092977
H	-1.581722	4.062465	0.193754
H	-3.138775	3.242060	0.341535

$I5_3$

E(oniom)= –665.398884742070 a.u.

O	-1.070195	2.779437	0.637412
H	-0.692722	3.051551	-2.060611
N	2.666018	-0.344307	-0.885785
B	0.346110	0.841710	-0.579953
C	1.546109	0.472674	-1.219483
N	-2.529366	-0.569829	0.841621
B	-1.136905	1.443723	-0.019835

H	3.425942	0.261485	3.316194
C	3.632499	-0.355845	0.073037
C	4.382792	-0.139949	-1.107485
C	4.305778	1.143923	-1.918587
H	3.721488	1.920002	-1.358921
C	3.549996	0.871097	-3.224643
H	3.388288	1.790886	-3.793919
H	2.559609	0.431470	-3.009347
H	4.080667	0.168348	-3.873886
C	5.703069	1.717970	-2.189374
H	5.636881	2.694593	-2.681263
H	6.291310	1.068865	-2.848511
H	6.273661	1.844269	-1.262649
C	5.183899	-1.186002	-1.587819
H	5.780729	-1.034393	-2.486657
C	5.212933	-2.422755	-0.942657
H	5.849868	-3.220156	-1.323732
C	4.407778	-2.646143	0.172453
H	4.390043	-3.629038	0.638795
C	2.639181	-1.964582	1.824484
H	2.077865	-1.050386	2.145676
C	3.591028	-1.627156	0.688252
C	1.593993	-2.955243	1.284277
H	0.725003	-3.027646	1.964454
H	1.998354	-3.957066	1.136867
H	1.181595	-2.605038	0.319550
C	3.378424	-2.517462	3.054366
H	3.539048	-1.704551	3.812771
H	4.382966	-2.951562	2.783821
H	2.778781	-3.329232	3.542548
C	-0.422812	-1.760102	-2.627429
H	-1.169494	-1.073287	-2.241060
C	0.868491	-1.666880	-2.299327
C	1.943346	-2.582480	-2.805429
H	1.540397	-3.336248	-3.483903
H	2.435034	-3.082035	-1.960729
H	2.716047	-2.005503	-3.326839

$TS6_3$

E(oniom)= –665.384379690092 a.u.

νmin = –261.8 cm^{-1}

O	-0.379516	-2.645244	0.632521
H	0.704150	-2.190786	-1.735435
N	2.909035	0.418768	0.768256
B	0.542773	-0.469004	0.098541
C	1.494128	0.325064	0.861097
N	-3.208370	-0.491360	-0.687941
B	-0.986468	-1.247649	0.461593

C	1.782815	1.025557	-2.646462	C	1.095431	1.168097	2.089991
C	0.495928	1.043662	-3.473185	C	-0.249892	1.864767	1.921866
H	0.694515	1.245215	-4.528781	H	-0.478820	2.522429	2.763277
H	-0.193162	1.827114	-3.111937	H	-1.063736	1.126872	1.839178
H	-0.040418	0.083429	-3.403035	H	-0.279390	2.464878	0.999370
C	2.377336	2.436768	-2.615740	C	1.026563	0.259234	3.328709
H	2.679381	2.774260	-3.608931	H	0.800124	0.828837	4.232422
H	3.258884	2.479172	-1.950740	H	1.976747	-0.270896	3.488032
H	1.654315	3.162302	-2.217032	H	0.249492	-0.504110	3.201458
C	2.807000	-0.005836	-3.197410	C	2.255762	2.187721	2.201982
H	2.267084	-0.875874	-3.584612	H	2.061827	3.022591	1.519667
H	3.421656	0.392228	-4.009695	H	2.382605	2.603207	3.205684
C	3.659199	-0.439942	-1.978577	C	3.504108	1.413597	1.715070
C	4.882158	0.470090	-1.780043	C	4.210237	0.724843	2.887801
H	5.579303	0.398962	-2.617757	H	4.435320	1.437018	3.685920
H	5.427023	0.209840	-0.866282	H	5.158400	0.263101	2.577553
H	4.578458	1.525872	-1.689769	H	3.577262	-0.068962	3.316372
C	4.157332	-1.875758	-2.169456	C	4.499287	2.352911	1.025036
H	4.850535	-1.942029	-3.012503	H	4.881374	3.103095	1.723144
H	3.328865	-2.567209	-2.361884	H	4.043159	2.891719	0.177696
H	4.679329	-2.243757	-1.273283	H	5.360701	1.806978	0.621394
C	3.047809	-0.441970	0.507513	C	3.568512	0.203968	-0.505196
C	2.957879	-1.716052	1.125439	C	3.231383	0.949760	-1.666388
C	2.393117	-2.936352	0.420000	C	2.115265	1.979335	-1.705293
H	2.001457	-2.628161	-0.595096	H	1.566030	1.992193	-0.726814
C	1.210345	-3.516356	1.214115	C	1.095354	1.610811	-2.792305
H	0.655164	-4.243370	0.605045	H	0.291318	2.356724	-2.848130
H	0.508157	-2.717628	1.517133	H	0.636768	0.640190	-2.550890
H	1.538477	-4.025842	2.125117	H	1.549366	1.532532	-3.783290
C	3.363087	-1.869665	2.460029	C	3.959198	0.755141	-2.849120
H	3.329669	-2.855612	2.919840	H	3.701989	1.332716	-3.736352
C	3.477446	-4.006341	0.229988	C	2.698156	3.384539	-1.923557
H	3.117613	-4.831606	-0.390571	H	1.917054	4.148245	-1.881016
H	3.810528	-4.426177	1.184423	H	3.201954	3.476382	-2.890283
H	4.364871	-3.576587	-0.259718	H	3.439556	3.612649	-1.139965
C	3.798619	-0.778991	3.208786	C	5.012800	-0.154779	-2.909045
H	4.111840	-0.911558	4.240843	H	5.578251	-0.280291	-3.828287
C	3.821518	0.486337	2.624828	C	5.329013	-0.904819	-1.779590
H	4.135976	1.343886	3.218737	H	6.144517	-1.625337	-1.827834
C	3.455439	0.674913	1.284114	C	4.611723	-0.755649	-0.582734
C	3.496199	2.092137	0.742019	C	4.984891	-1.676191	0.565206
H	3.344845	2.073116	-0.376085	H	4.205591	-1.575760	1.376739
C	2.358687	2.923375	1.348169	C	4.981966	-3.146100	0.110639
H	2.394054	3.957144	0.990442	H	5.065969	-3.822170	0.966668
H	2.392858	2.940833	2.439906	H	5.815109	-3.367646	-0.564469
H	1.376847	2.519740	1.047587	H	4.056778	-3.392681	-0.424237
C	4.861126	2.749537	1.002913	C	6.354392	-1.298727	1.145539

H	4.940506	3.704315	0.473561
H	5.681356	2.107693	0.664570
H	5.021322	2.953192	2.066596
C	-1.705201	0.415620	1.063115
H	-1.915751	1.452621	-0.979437
C	-1.324127	0.397560	2.561675
C	0.046499	-0.264057	2.655156
H	0.388950	-0.343073	3.693219
H	0.819445	0.323207	2.126206
H	0.075149	-1.278405	2.221594
C	-1.304765	1.794016	3.146564
H	-1.129983	1.785271	4.226143
H	-2.232044	2.345152	2.946160
H	-0.505806	2.403978	2.681866
C	-2.449831	-0.478699	3.185551
H	-2.114231	-1.044436	4.056183
H	-3.269037	0.166411	3.513383
C	-2.937956	-1.403327	2.050406
C	-2.216688	-2.737716	2.049919
H	-2.401699	-3.289590	2.979727
H	-1.117992	-2.620493	1.961656
H	-2.542940	-3.390963	1.221684
C	-4.443492	-1.601287	2.047658
H	-4.768548	-2.164301	2.932110
H	-4.785896	-2.162226	1.165941
H	-4.996620	-0.651315	2.050045
C	-3.088798	-0.799710	-0.489158
C	-2.503201	-1.739137	-1.366093
C	-1.216144	-2.485803	-1.062081
H	-0.780943	-2.102709	-0.102495
C	-0.163820	-2.241219	-2.147054
H	0.854400	-2.390701	-1.736956
H	-0.178724	-1.190767	-2.497897
H	-0.283882	-2.887424	-3.017372
C	-1.497013	-3.988916	-0.911482
H	-0.587224	-4.514623	-0.582004
H	-1.816244	-4.443848	-1.854662
H	-2.284834	-4.179226	-0.168548
C	-3.127759	-1.981831	-2.601076
H	-2.689668	-2.710440	-3.283705
C	-4.281635	-1.295682	-2.970617
H	-4.755136	-1.499502	-3.930642
C	-4.819453	-0.329298	-2.119266
H	-5.699404	0.233470	-2.431772
C	-4.788495	1.086212	-0.042856
H	-4.382687	1.038105	0.999982
C	-4.231617	-0.056729	-0.877555
H	6.684476	-2.025763	1.893712
H	6.306274	-0.312098	1.634783
H	7.126314	-1.245308	0.371414
C	-2.175199	-1.300868	-0.608095
H	-1.465464	-0.855319	1.533650
C	-2.357087	-2.374100	-1.720652
C	-1.517700	-1.946843	-2.921549
H	-1.574249	-2.679455	-3.733059
H	-0.448026	-1.857576	-2.637825
H	-1.820409	-0.971228	-3.325820
C	-1.977462	-3.760747	-1.241842
H	-2.310232	-4.539934	-1.933741
H	-2.383755	-3.983196	-0.245654
H	-0.884858	-3.861743	-1.125755
C	-3.881127	-2.289515	-2.023592
H	-4.130105	-2.585370	-3.044448
H	-4.420375	-2.959141	-1.347463
C	-4.270808	-0.830875	-1.726966
C	-4.142379	0.043312	-2.963636
H	-4.740369	-0.356285	-3.792320
H	-3.100808	0.105691	-3.323381
H	-4.499251	1.072584	-2.787424
C	-5.657230	-0.692329	-1.120968
H	-6.432762	-0.961520	-1.847532
H	-5.867471	0.337417	-0.797942
H	-5.804200	-1.330915	-0.244091
C	-3.426748	0.560776	0.301981
C	-3.146726	1.912910	0.003357
C	-2.577054	2.394978	-1.321193
H	-2.261175	1.512457	-1.934308
C	-1.332719	3.264136	-1.114581
H	-0.759633	3.359796	-2.050117
H	-0.644804	2.798762	-0.378261
H	-1.568495	4.269476	-0.758034
C	-3.645443	3.190392	-2.089042
H	-3.244005	3.574063	-3.033518
H	-4.003297	4.052310	-1.515178
H	-4.520807	2.565792	-2.322985
C	-3.410355	2.884430	0.984432
H	-3.198625	3.931848	0.767582
C	-3.920118	2.531971	2.230979
H	-4.106719	3.298256	2.982548
C	-4.183215	1.192849	2.518866
H	-4.570055	0.918703	3.502897
C	-4.243302	-1.249870	1.969528
H	-4.125462	-1.926048	1.085215
C	-3.952565	0.190893	1.566610

C	-4.314901	2.401393	-0.670898
H	-4.497099	3.254769	-0.012602
H	-4.796725	2.606110	-1.630393
H	-3.213881	2.365602	-0.859842
C	-6.317464	1.025754	0.065430
H	-6.686854	1.786114	0.763010
H	-6.660955	0.047755	0.416696
H	-6.805031	1.217555	-0.897797
C	-0.656964	3.932071	-1.423713
H	-0.476197	4.889401	-1.898867
C	-0.834367	3.879812	-0.089838
C	-0.786840	5.107438	0.779460
H	-0.591454	6.007864	0.192584
H	-0.001998	4.998339	1.536425
H	-1.738769	5.228642	1.308835

I6$_3$
E(oniom)= –665.412445464415 a.u.

O	-0.536658	2.948175	-0.105199
H	0.909070	2.204991	1.978007
N	2.755845	-0.458737	-0.804358
B	0.722603	0.903404	0.001691
C	1.516607	0.058260	-0.973032
N	-2.922140	0.143392	0.834429
B	-0.975735	1.528211	-0.230240
C	1.101908	-0.317810	-2.425923
C	-0.364738	-0.672699	-2.537926
H	-0.672568	-0.875566	-3.564388
H	-0.995479	0.176585	-2.149001
H	-0.631184	-1.539860	-1.916191
C	1.429301	0.864528	-3.341030
H	1.163651	0.649060	-4.380359
H	2.495332	1.129451	-3.314580
H	0.858845	1.759192	-3.035888
C	2.010738	-1.539374	-2.740605
H	1.522178	-2.454882	-2.392710
H	2.209701	-1.663438	-3.807692
C	3.299087	-1.302791	-1.924931
C	4.350642	-0.557141	-2.739624
H	4.630379	-1.122149	-3.635205
H	5.274673	-0.385542	-2.163009
H	3.981343	0.426047	-3.076099
C	3.889653	-2.601694	-1.386410
H	4.221348	-3.252303	-2.203243
H	3.168641	-3.171790	-0.785863
H	4.759526	-2.419072	-0.740819
C	3.441689	-0.407799	0.479144

C	-3.203893	-1.682491	3.008354
H	-3.267092	-2.752553	3.223754
H	-3.289893	-1.132819	3.949180
H	-2.172009	-1.485086	2.604971
C	-5.681621	-1.422244	2.482472
H	-6.202802	-2.249509	1.932570
H	-6.289691	-0.480213	2.353207
H	-5.694381	-1.688407	3.577568
C	1.355940	-2.039566	-0.875858
H	2.420352	-1.946928	-1.077423
C	0.933482	-2.442321	0.398017
C	1.860960	-2.822115	1.508631
H	2.775226	-2.208423	1.468253
H	2.141677	-3.879591	1.416699
H	1.362006	-2.670799	2.467918

TS7$_3$
E(oniom)= –665.357885557871 a.u.
νmin = –1072.2 cm^{-1}

O	-0.770690	2.870763	-0.855671
H	1.084140	1.251765	1.130296
N	2.734065	-0.730129	-0.733253
B	0.627604	0.780200	-0.372631
C	1.486980	-0.264523	-1.023300
N	-2.834947	0.228202	0.797913
B	-1.004862	1.389871	-0.669778
C	1.119010	-0.881715	-2.393370
C	-0.357874	-1.195865	-2.532139
H	-0.620876	-1.538930	-3.533179
H	-0.963428	-0.273525	-2.310939
H	-0.693653	-1.947472	-1.806424
C	1.531341	0.132608	-3.467487
H	1.269746	-0.213722	-4.469936
H	2.608226	0.345661	-3.449497
H	1.011773	1.089786	-3.295117
C	2.006117	-2.157135	-2.443367
H	1.480643	-2.980467	-1.949983
H	2.240086	-2.480884	-3.460404
C	3.276042	-1.793322	-1.637937
C	4.381690	-1.271042	-2.552353
H	4.651093	-2.010423	-3.312557
H	5.297494	-1.025643	-1.992210
H	4.060685	-0.356875	-3.081935
C	3.806636	-2.981920	-0.837641
H	4.164379	-3.775861	-1.501231
H	3.031805	-3.416294	-0.189839
H	4.641771	-2.693919	-0.186995

C	3.044863	-1.307854	1.501387	C	3.411742	-0.301321	0.478107
C	1.824715	-2.204361	1.366489	C	3.101477	-0.936544	1.707129
H	1.547654	-2.305630	0.285065	C	2.095488	-2.070304	1.812952
C	0.654699	-1.531969	2.089268	H	1.866755	-2.466882	0.785888
H	-0.278656	-2.092565	1.967288	C	3.729048	-0.499541	2.881868
H	0.484978	-0.520534	1.643144	H	3.481975	-0.972902	3.831217
H	0.848710	-1.393392	3.153847	C	2.653521	-3.240961	2.636979
C	3.767055	-1.331300	2.701749	H	2.002155	-4.117422	2.564215
H	3.463820	-2.010058	3.498003	H	2.741541	-2.989159	3.699382
C	2.086005	-3.621319	1.899686	H	3.650306	-3.532895	2.288128
H	1.251311	-4.287195	1.656041	C	4.669784	0.528849	2.852210
H	2.197542	-3.632115	2.989708	H	5.154887	0.852732	3.770479
H	2.998139	-4.049864	1.472741	C	4.985594	1.138311	1.640519
C	4.863082	-0.490731	2.895971	H	5.724834	1.938817	1.620071
H	5.425667	-0.534094	3.826353	C	4.364408	0.745903	0.443529
C	5.220930	0.418319	1.903697	C	4.764144	1.480664	-0.825940
H	6.058828	1.093910	2.071705	H	4.079369	1.174049	-1.660485
C	4.506906	0.494555	0.696132	C	4.625653	3.001085	-0.663289
C	4.891096	1.594469	-0.279780	H	4.777824	3.512293	-1.619041
H	4.178082	1.591070	-1.145570	H	5.340419	3.414042	0.053356
C	4.771224	2.965178	0.403301	H	3.609424	3.256007	-0.312791
H	4.887021	3.781117	-0.315908	C	6.209634	1.121139	-1.204326
H	5.519872	3.107555	1.188099	H	6.510513	1.624180	-2.128830
H	3.776983	3.070743	0.875381	H	6.316380	0.036433	-1.361368
C	6.313710	1.378856	-0.814622	H	6.920751	1.411297	-0.424401
H	6.596410	2.172111	-1.514282	C	-1.924638	1.165388	0.616290
H	6.391540	0.416669	-1.344346	H	-1.558012	0.895773	-1.671041
H	7.057225	1.371816	-0.010666	C	-1.889595	2.022030	1.915340
C	-2.005983	1.098166	0.895545	C	-1.797209	3.513269	1.640093
H	-1.406478	1.234811	-1.361841	H	-2.318591	4.108151	2.395766
C	-2.165134	1.794864	2.280968	H	-2.206497	3.773467	0.649913
C	-1.118815	1.263723	3.252159	H	-0.754191	3.858347	1.628196
H	-1.193924	1.745424	4.230968	C	-3.263536	1.691842	2.571449
H	-0.096657	1.469812	2.863566	H	-3.249957	1.786430	3.659263
H	-1.190109	0.178099	3.398519	H	-4.018461	2.386680	2.191356
C	-2.068963	3.300811	2.135489	C	-3.603134	0.264422	2.110289
H	-2.258405	3.819285	3.078075	C	-3.089841	-0.767284	3.103672
H	-2.752525	3.696449	1.375373	H	-1.988396	-0.779586	3.155235
H	-1.059351	3.595433	1.779002	H	-3.431477	-1.786927	2.862648
C	-3.603806	1.368499	2.705415	H	-3.453385	-0.551339	4.116137
H	-3.737663	1.340447	3.788825	C	-5.087283	0.059950	1.847446
H	-4.328368	2.083379	2.304514	H	-5.667852	0.152642	2.773363
C	-3.821007	-0.006598	2.050467	H	-5.299450	-0.934107	1.429230
C	-3.346982	-1.132049	2.958572	H	-5.495619	0.790970	1.135405
H	-3.835645	-1.076485	3.939107	C	-3.268386	-0.626939	-0.305845
H	-2.259362	-1.081168	3.143137	C	-2.946121	-2.001156	-0.352149
H	-3.575865	-2.128111	2.549010	C	-2.113215	-2.722676	0.694276

C	-5.260556	-0.239711	1.616970
H	-5.930500	-0.271002	2.485016
H	-5.382216	-1.189707	1.078526
H	-5.635278	0.548835	0.948906
C	-3.259384	-0.509004	-0.427996
C	-2.949793	-1.864533	-0.671187
C	-2.178397	-2.756687	0.286564
H	-1.716215	-2.129040	1.100694
C	-1.022063	-3.471288	-0.427685
H	-0.352499	-3.953881	0.293571
H	-0.419938	-2.743448	-1.003699
H	-1.369227	-4.240303	-1.123656
C	-3.122253	-3.792145	0.916090
H	-2.568391	-4.500424	1.542103
H	-3.655033	-4.375310	0.157342
H	-3.879471	-3.306192	1.550513
C	-3.374574	-2.444236	-1.880851
H	-3.152386	-3.493778	-2.075085
C	-4.062230	-1.703442	-2.836657
H	-4.373795	-2.168095	-3.770867
C	-4.347946	-0.359604	-2.594889
H	-4.874349	0.222929	-3.351568
C	-4.320300	1.723904	-1.210381
H	-4.101417	2.053205	-0.162682
C	-3.968619	0.254009	-1.394509
C	-3.450345	2.566895	-2.146869
H	-3.560462	3.636433	-1.946674
H	-3.669421	2.393380	-3.201783
H	-2.371236	2.322010	-1.977802
C	-5.817543	1.967438	-1.450028
H	-6.080948	3.006272	-1.220603
H	-6.436512	1.315555	-0.824758
H	-6.101550	1.790437	-2.493373
C	1.394844	1.951246	1.028449
H	2.485726	2.033323	1.080813
C	0.793801	2.552849	-0.198194
C	1.600954	3.341130	-1.190387
H	2.621993	2.949509	-1.271329
H	1.647490	4.393001	-0.876512
H	1.127909	3.303222	-2.181028
H	-1.660343	-1.967915	1.387656
C	-0.952007	-3.499426	0.061293
H	-0.259633	-3.868456	0.824508
H	-0.375175	-2.839551	-0.616651
H	-1.290720	-4.358981	-0.524137
C	-3.004907	-3.681868	1.499772
H	-2.411725	-4.268282	2.209622
H	-3.530890	-4.391577	0.852042
H	-3.766499	-3.130693	2.071671
C	-3.428423	-2.770068	-1.426338
H	-3.184444	-3.831540	-1.473018
C	-4.200217	-2.200594	-2.434352
H	-4.555920	-2.810430	-3.263419
C	-4.512447	-0.842254	-2.381240
H	-5.107935	-0.395772	-3.178117
C	-4.436598	1.437097	-1.352671
H	-4.148247	1.933154	-0.391419
C	-4.065726	-0.039303	-1.323873
C	-3.654678	2.121684	-2.476866
H	-3.747899	3.209651	-2.432380
H	-3.961254	1.792412	-3.470963
H	-2.562429	1.890724	-2.366277
C	-5.952125	1.624168	-1.517326
H	-6.221591	2.682693	-1.430036
H	-6.513084	1.067198	-0.760415
H	-6.302297	1.287495	-2.499703
C	1.308465	2.265228	-0.017344
H	2.341202	2.521626	0.228471
C	0.446243	3.240663	-0.408582
C	0.735025	4.707632	-0.514003
H	1.760093	4.931020	-0.207224
H	0.049965	5.298120	0.109348
H	0.590680	5.039075	-1.548989
C	-0.712106	1.535484	2.756816
H	0.214545	1.445344	2.117080
H	-0.474719	2.229196	3.568025
H	-0.891509	0.547318	3.198173
C	0.788117	-1.539772	2.410248
H	0.312641	-0.848837	1.689230
H	0.084080	-2.352192	2.613147
H	0.955381	-0.999358	3.346015

$I7_3$
E(oniom)= –665.437123080338 a.u.

O	-0.453383	-2.118837	-0.253746
H	0.455341	0.910320	1.478125
N	-2.504439	0.939902	-0.486270

$TS8_3$
E(oniom)= –665.414745528435 a.u.
νmin = –659.1 cm^{-1}

O	0.218059	2.362254	-0.111775
H	-0.091392	-0.767801	1.507570

B	-0.372354	0.221402	0.890375	N	2.645717	-1.040940	-0.535582
C	-1.267859	1.133092	-0.008935	B	0.571747	-0.037741	0.759992
N	3.073245	-0.775852	0.213688	C	1.367774	-1.007884	-0.200132
B	0.462870	-0.941847	-0.338690	N	-3.154930	0.601235	-0.344484
C	-0.813423	2.522137	-0.552896	B	-0.500588	1.079833	-0.139506
C	-0.146288	3.332708	0.548035	C	0.680241	-2.145419	-0.983957
H	0.025760	4.368129	0.237346	C	-0.348926	-2.845833	-0.120168
H	0.834311	2.901789	0.803609	H	-0.857484	-3.656403	-0.650781
H	-0.739423	3.350227	1.469244	H	-1.124836	-2.134031	0.216284
C	0.132163	2.331304	-1.731612	H	0.088438	-3.268156	0.791795
H	0.495647	3.285761	-2.121856	C	0.067471	-1.482492	-2.214896
H	-0.339737	1.780839	-2.557558	H	-0.664639	-2.137718	-2.708725
H	1.010695	1.746662	-1.414112	H	0.819657	-1.193156	-2.958403
C	-2.147860	3.192239	-0.983203	H	-0.481882	-0.539998	-1.925427
H	-2.598042	3.698712	-0.123754	C	1.853429	-3.094233	-1.353068
H	-2.022219	3.934506	-1.774831	H	1.992106	-3.829622	-0.555419
C	-3.043208	2.017504	-1.405598	H	1.682427	-3.648002	-2.278298
C	-2.827502	1.669929	-2.875205	C	3.091830	-2.179411	-1.440416
H	-2.922264	2.558997	-3.508354	C	3.338806	-1.700057	-2.863073
H	-3.565753	0.939539	-3.242047	H	3.486278	-2.544499	-3.546074
H	-1.823965	1.248523	-3.055726	H	4.239833	-1.067374	-2.937233
C	-4.521701	2.273721	-1.151976	H	2.488770	-1.111655	-3.250989
H	-4.891922	3.092581	-1.778705	C	4.346944	-2.838914	-0.890490
H	-4.733732	2.543979	-0.102154	H	4.677722	-3.659855	-1.538137
H	-5.135214	1.388921	-1.372024	H	4.192106	-3.261851	0.112426
C	-3.398532	-0.095168	0.022619	H	5.183660	-2.130562	-0.812037
C	-3.988029	0.099984	1.301293	C	3.599738	-0.070071	-0.007094
C	-3.687453	1.292312	2.200190	C	4.196505	-0.316827	1.254847
H	-2.860845	1.906524	1.761992	C	3.897559	-1.549601	2.095392
C	-3.216146	0.831661	3.585772	H	3.266442	-2.266115	1.508845
H	-2.844995	1.671935	4.178427	C	3.107899	-1.163688	3.350687
H	-2.382949	0.107218	3.479183	H	2.807311	-2.047077	3.920818
H	-4.007941	0.340364	4.156761	H	2.188473	-0.615005	3.078270
C	-4.905665	-0.848631	1.771975	H	3.680532	-0.513253	4.018526
H	-5.359634	-0.714533	2.752941	C	5.093949	0.629560	1.769123
C	-4.928327	2.190836	2.327204	H	5.549660	0.461537	2.744248
H	-4.725670	3.050520	2.973626	C	5.197635	-2.278324	2.474210
H	-5.780306	1.652470	2.755379	H	4.981062	-3.247897	2.934706
H	-5.241241	2.573582	1.343167	H	5.788026	-1.705282	3.198472
C	-5.244687	-1.964167	1.006271	H	5.836420	-2.450878	1.602595
H	-5.960189	-2.689702	1.387299	C	5.400879	1.788475	1.056643
C	-4.662710	-2.145236	-0.244696	H	6.094007	2.516755	1.474221
H	-4.927311	-3.019815	-0.838913	C	4.816677	2.014399	-0.187499
C	-3.731286	-1.225590	-0.758534	H	5.057460	2.924286	-0.737030
C	-3.159779	-1.509434	-2.138645	C	3.910297	1.096714	-0.743923
H	-2.320503	-0.795623	-2.343125	C	3.341104	1.411719	-2.118985
C	-2.566690	-2.919512	-2.240771	H	2.568023	0.647396	-2.390828

H	-2.179541	-3.113213	-3.244676
H	-3.286837	-3.703740	-2.000278
H	-1.709099	-3.013173	-1.544983
C	-4.261756	-1.327395	-3.198249
H	-3.894852	-1.595167	-4.194420
H	-4.607612	-0.284411	-3.236037
H	-5.131626	-1.958994	-2.991621
C	1.932772	-1.433181	0.114801
H	0.580567	-0.517882	-1.490970
C	2.255001	-2.918928	0.468789
C	1.867078	-3.145493	1.927574
H	1.941446	-4.200189	2.206632
H	0.823975	-2.831244	2.108186
H	2.489474	-2.564510	2.620972
C	1.569967	-3.900704	-0.462992
H	1.918942	-4.924453	-0.305597
H	1.712881	-3.641785	-1.517712
H	0.472638	-3.890918	-0.309523
C	3.797340	-3.000775	0.270837
H	4.266393	-3.752730	0.908758
H	4.015228	-3.272701	-0.766241
C	4.319792	-1.586540	0.554916
C	4.713451	-1.413524	2.014639
H	5.442793	-2.172879	2.320257
H	3.843030	-1.513808	2.687787
H	5.175948	-0.431933	2.209457
C	5.482619	-1.194944	-0.345394
H	6.377778	-1.781592	-0.104847
H	5.752004	-0.135324	-0.238617
H	5.263673	-1.363471	-1.409586
C	3.194470	0.644494	-0.095656
C	3.195177	1.610586	0.935722
C	3.062883	1.279618	2.415360
H	2.891456	0.177204	2.538597
C	1.856667	1.973749	3.049665
H	1.752003	1.712296	4.105636
H	0.920136	1.611223	2.533231
H	1.890842	3.060922	2.972828
C	4.357161	1.663923	3.151244
H	4.287612	1.417793	4.216390
H	4.565511	2.736448	3.080392
H	5.223304	1.127553	2.737334
C	3.359877	2.961323	0.581870
H	3.361246	3.719644	1.365338
C	3.518784	3.346156	-0.746793
H	3.637883	4.398479	-0.999798
C	3.527603	2.381341	-1.754914

C	2.639774	2.772215	-2.159568
H	2.305507	3.017560	-3.171564
H	3.278448	3.588595	-1.813259
H	1.738827	2.757801	-1.516087
C	4.472737	1.364320	-3.161193
H	4.086119	1.544016	-4.169439
H	4.973812	0.385608	-3.160070
H	5.235765	2.125368	-2.965808
C	-1.998596	1.334488	-0.425767
H	-0.894632	0.722725	-1.422007
C	-2.392911	2.741108	-0.987744
C	-2.324651	3.749492	0.156675
H	-2.572345	4.760233	-0.176138
H	-1.299728	3.774360	0.571626
H	-3.000605	3.486181	0.983083
C	-1.497016	3.182377	-2.139021
H	-1.850658	4.119167	-2.581863
H	-1.437548	2.436837	-2.936374
H	-0.465607	3.367027	-1.785638
C	-3.841268	2.529848	-1.486967
H	-4.451843	3.434512	-1.433245
H	-3.827873	2.206315	-2.532871
C	-4.408414	1.398510	-0.617817
C	-5.010193	1.923784	0.686765
H	-5.940317	2.471588	0.510733
H	-4.318739	2.613819	1.202289
H	-5.232358	1.110467	1.393417
C	-5.482688	0.634537	-1.378832
H	-6.314114	1.295393	-1.648325
H	-5.902765	-0.189695	-0.781241
H	-5.093079	0.200350	-2.311134
C	-3.228916	-0.622426	0.442788
C	-2.975030	-0.603297	1.839637
C	-2.633861	0.647283	2.637677
H	-2.508467	1.522251	1.950989
C	-1.308671	0.458765	3.380749
H	-0.872998	1.418669	3.669671
H	-0.561441	-0.022303	2.691932
H	-1.396537	-0.163317	4.270667
C	-3.773244	0.964922	3.622232
H	-3.603635	1.931074	4.107773
H	-3.848488	0.212290	4.413774
H	-4.744421	1.002520	3.113880
C	-3.049688	-1.800715	2.569728
H	-2.839562	-1.785051	3.639613
C	-3.389237	-3.002677	1.956459
H	-3.444712	-3.920278	2.537517

H	3.649953	2.690189	-2.792737
C	3.418273	0.015371	-2.593796
H	3.583387	-1.015627	-2.181540
C	3.378576	1.021406	-1.451407
C	2.083702	0.005725	-3.340082
H	2.073627	-0.736616	-4.141619
H	1.839863	0.977313	-3.775181
H	1.250594	-0.265716	-2.627495
C	4.584537	0.305647	-3.552074
H	4.716390	-0.516588	-4.263715
H	5.527788	0.440536	-3.012935
H	4.412449	1.213009	-4.142188
C	-1.104906	-0.983011	1.653862
H	-1.642030	-0.949699	2.607643
C	-1.131712	-2.115853	0.931767
C	-1.804059	-3.418655	1.252843
H	-2.320021	-3.353922	2.214589
H	-1.078811	-4.240897	1.288568
H	-2.545317	-3.667440	0.482483

C	-3.663963	-3.015721	0.591892
H	-3.941431	-3.955323	0.115098
C	-3.952091	-2.012216	-1.652664
H	-3.831388	-1.029801	-2.180347
C	-3.591449	-1.845946	-0.183216
C	-3.056582	-3.033877	-2.365793
H	-3.358565	-3.155701	-3.410985
H	-3.098788	-4.021443	-1.895114
H	-2.002632	-2.713287	-2.359115
C	-5.418625	-2.471610	-1.777584
H	-5.770356	-2.394977	-2.810428
H	-6.083516	-1.865617	-1.146176
H	-5.545282	-3.513304	-1.463784
C	1.431921	1.142252	1.442131
H	2.170222	1.094376	2.246045
C	1.177054	2.329567	0.874800
C	1.748664	3.678313	1.188655
H	2.513439	3.591059	1.965404
H	0.965250	4.361725	1.534614
H	2.205883	4.125432	0.298397

3a (R,R)/(S,S)

E(oniom)= –665.470389135933 a.u.

O	0.526715	-1.977257	1.344066
H	0.218958	0.369485	0.884171
N	-2.828245	0.789088	-0.839659
B	-0.889533	-0.149157	0.502834
C	-1.523784	0.719453	-0.567441
N	3.020025	-0.639867	0.292875
B	0.548139	-1.049174	0.293802
C	-0.808770	1.667050	-1.571757
C	0.246302	2.504887	-0.870241
H	0.656437	3.278125	-1.530116
H	1.101908	1.897145	-0.532096
H	-0.145402	3.007620	0.022517
C	-0.199543	0.841186	-2.699974
H	0.225669	1.480918	-3.480601
H	-0.937420	0.179127	-3.176765
H	0.618747	0.196557	-2.325195
C	-1.980050	2.544065	-2.104715
H	-2.109563	3.416655	-1.457789
H	-1.811279	2.910999	-3.119087
C	-3.233504	1.648932	-2.006218
C	-3.448196	0.794497	-3.250168
H	-3.720268	1.409774	-4.114707
H	-4.254631	0.055341	-3.107959
H	-2.540557	0.230616	-3.525155

3_{taut}

E(scf) = –665.470119950887 a.u.

O	0.412910	-1.980728	1.291187
H	0.048723	0.955074	1.005439
N	-2.786869	0.720224	-0.902127
B	-0.760412	0.106767	0.575689
C	-1.493571	0.757737	-0.621932
N	2.953881	-0.613060	0.363246
B	0.534269	-1.013719	0.301789
C	-0.821502	1.611476	-1.734336
C	0.211911	2.552998	-1.143650
H	0.640414	3.217392	-1.902093
H	1.055715	1.999172	-0.693596
H	-0.200565	3.182387	-0.346358
C	-0.192498	0.678204	-2.764610
H	0.203837	1.235783	-3.619744
H	-0.906067	-0.063107	-3.151346
H	0.654849	0.115836	-2.323803
C	-2.027018	2.378002	-2.353656
H	-2.187923	3.312179	-1.807957
H	-1.877094	2.634888	-3.404080
C	-3.241290	1.448926	-2.147559
C	-3.432840	0.467261	-3.295811
H	-3.710429	0.985900	-4.220498
H	-4.228888	-0.267257	-3.083823
H	-2.513402	-0.104284	-3.509482

C	-4.493216	2.442982	-1.697982	C	-4.529627	2.215455	-1.897018
H	-4.768789	3.091194	-2.538159	H	-4.850490	2.754350	-2.796783
H	-4.373935	3.088541	-0.814676	H	-4.424134	2.960847	-1.094610
H	-5.352747	1.788263	-1.496687	H	-5.355163	1.550837	-1.605672
C	-3.791660	0.030256	-0.056059	C	-3.720824	0.012070	-0.035909
C	-4.417253	0.651321	1.052682	C	-4.353382	0.721019	1.015102
C	-4.057320	2.052376	1.521394	C	-4.043853	2.175300	1.335123
H	-3.500592	2.591357	0.709488	H	-3.493226	2.643697	0.477293
C	-3.132627	1.965106	2.740696	C	-3.135748	2.253481	2.567025
H	-2.861216	2.958736	3.109019	H	-2.900095	3.290174	2.825141
H	-2.198947	1.437724	2.488203	H	-2.181564	1.732411	2.385725
H	-3.594537	1.417758	3.568856	H	-3.591696	1.787175	3.446335
C	-5.371399	-0.077246	1.775738	C	-5.272379	0.037622	1.822960
H	-5.860209	0.381308	2.634667	H	-5.763641	0.563714	2.641067
C	-5.311511	2.881490	1.836906	C	-5.330773	2.987774	1.546571
H	-5.052423	3.930935	2.012732	H	-5.110848	4.058999	1.607530
H	-5.821108	2.523410	2.739014	H	-5.837655	2.713424	2.479100
H	-6.036403	2.844603	1.017253	H	-6.045034	2.834627	0.731231
C	-5.695077	-1.387106	1.419398	C	-5.558041	-1.310382	1.602987
H	-6.447175	-1.934254	1.985615	H	-6.281481	-1.822980	2.235311
C	-5.042041	-2.000705	0.352762	C	-4.905773	-2.003662	0.585981
H	-5.276678	-3.034023	0.098861	H	-5.114548	-3.062618	0.435564
C	-4.065821	-1.314854	-0.388063	C	-3.964760	-1.364338	-0.237625
C	-3.323374	-2.067102	-1.479946	C	-3.238309	-2.195685	-1.282269
H	-2.530488	-1.412122	-1.924041	H	-2.458169	-1.572365	-1.789538
C	-2.605644	-3.284240	-0.884849	C	-2.504932	-3.368087	-0.620720
H	-1.985696	-3.785134	-1.634167	H	-1.915052	-3.929936	-1.351405
H	-3.298655	-4.024094	-0.475823	H	-3.186859	-4.070758	-0.133875
H	-1.938682	-2.967300	-0.063823	H	-1.808181	-2.999129	0.150822
C	-4.291066	-2.471857	-2.601315	C	-4.229092	-2.688835	-2.347358
H	-3.764840	-3.003907	-3.400736	H	-3.717958	-3.275162	-3.118155
H	-4.772672	-1.587713	-3.042044	H	-4.730013	-1.843447	-2.841067
H	-5.083854	-3.134951	-2.239060	H	-5.007300	-3.328445	-1.917309
C	1.871238	-0.983241	-0.588597	C	1.868838	-1.036463	-0.568953
H	1.767303	-0.209219	-1.374228	H	1.797448	-0.307084	-1.399260
C	2.290650	-2.351643	-1.278580	C	2.346396	-2.432248	-1.148855
C	1.499399	-3.543365	-0.747524	C	1.540297	-3.607576	-0.603024
H	1.820673	-4.483475	-1.201907	H	1.900490	-4.564521	-0.987916
H	0.423441	-3.430284	-0.926576	H	0.476247	-3.521033	-0.848361
H	1.623276	-3.638239	0.347967	H	1.603353	-3.643281	0.501449
C	2.092508	-2.258989	-2.796710	C	2.244597	-2.427281	-2.680053
H	2.456303	-3.164358	-3.295126	H	2.648878	-3.353097	-3.103527
H	2.627517	-1.409855	-3.235111	H	2.800019	-1.595048	-3.127530
H	1.032093	-2.154840	-3.051140	H	1.202979	-2.348693	-3.007882
C	3.803003	-2.489342	-0.933429	C	3.835563	-2.525857	-0.703114
H	4.134274	-3.531871	-0.895506	H	4.175597	-3.558808	-0.577966
H	4.403675	-1.979465	-1.698018	H	4.478955	-2.057863	-1.459588

C	3.993692	-1.740327	0.408278	C	3.933511	-1.693408	0.598373
C	3.642595	-2.633638	1.603198	C	3.523387	-2.518348	1.822486
H	4.279089	-3.517958	1.650579	H	4.172205	-3.382992	1.966212
H	2.588335	-2.968282	1.542368	H	2.481506	-2.879041	1.717068
H	3.733344	-2.087306	2.548626	H	3.542505	-1.911768	2.735055
C	5.431162	-1.237777	0.574360	C	5.352270	-1.156672	0.813553
H	6.131086	-2.073481	0.666460	H	6.057875	-1.971966	0.997377
H	5.532285	-0.621963	1.476971	H	5.393140	-0.483313	1.679109
H	5.766834	-0.618036	-0.271130	H	5.725013	-0.585008	-0.050812
C	3.395585	0.749990	0.399196	C	3.330624	0.781529	0.374193
C	3.158851	1.378007	1.648794	C	3.081900	1.494060	1.576066
C	2.534522	0.631838	2.815181	C	2.466663	0.827790	2.794813
H	2.388405	-0.449985	2.533921	H	2.233956	-0.248456	2.555089
C	1.148899	1.214375	3.119538	C	1.141086	1.507902	3.155063
H	0.660485	0.667527	3.931964	H	0.645724	0.994522	3.983876
H	0.494546	1.114674	2.227669	H	0.446475	1.448920	2.280576
H	1.186349	2.269756	3.396411	H	1.260254	2.557815	3.425745
C	3.451394	0.673476	4.045167	C	3.452140	0.847145	3.972013
H	3.049114	0.051215	4.851115	H	3.045033	0.300190	4.828397
H	3.565599	1.687821	4.439879	H	3.671502	1.865850	4.306282
H	4.451309	0.299073	3.800524	H	4.402423	0.378040	3.695565
C	3.485601	2.731470	1.808243	C	3.400196	2.857157	1.643980
H	3.307859	3.215656	2.767486	H	3.213158	3.404636	2.566738
C	4.031547	3.465712	0.755653	C	3.948627	3.521711	0.547365
H	4.280892	4.514917	0.894246	H	4.186730	4.580278	0.613894
C	4.257959	2.848866	-0.473525	C	4.195224	2.820685	-0.631333
H	4.686568	3.425778	-1.291318	H	4.631844	3.341057	-1.482067
C	4.245220	0.888498	-2.031661	C	4.246642	0.753380	-2.038225
H	3.894274	-0.177815	-2.057625	H	3.885268	-0.309077	-2.009549
C	3.949397	1.494464	-0.670951	C	3.900918	1.452507	-0.735328
C	3.505783	1.648001	-3.142483	C	3.566981	1.439732	-3.231877
H	3.584048	1.122193	-4.098922	H	3.684213	0.849344	-4.145562
H	3.913428	2.654161	-3.288775	H	3.990594	2.430885	-3.427150
H	2.442495	1.761475	-2.901947	H	2.495037	1.576262	-3.048541
C	5.759782	0.871236	-2.287246	C	5.771480	0.705950	-2.219670
H	5.996363	0.409556	-3.250186	H	6.048520	0.169449	-3.131438
H	6.271651	0.294359	-1.498457	H	6.239604	0.189478	-1.364372
H	6.190197	1.877605	-2.286554	H	6.211033	1.706837	-2.275590
C	-1.509165	-0.967861	1.694244	C	-1.468351	-0.714618	1.755277
H	-2.449074	-0.845639	2.232355	H	-2.366326	-0.509319	2.340741
C	-0.628526	-1.904481	2.091339	C	-0.723461	-1.780083	2.077194
C	-0.670804	-2.871629	3.228892	C	-0.872488	-2.803126	3.154822
H	-1.628830	-2.800750	3.750026	H	-1.786373	-2.619506	3.725555
H	0.133768	-2.668225	3.943901	H	-0.017040	-2.772786	3.838293
H	-0.539594	-3.897046	2.866775	H	-0.917969	-3.811076	2.727642

3b (R,S)/(S,R)

E(scf) = –665.462474000736 a.u.

O	-0.709025	1.235416	-1.505650
H	1.019665	-1.270486	-1.610343
N	3.118412	-0.737051	0.799195
B	1.120912	-0.328822	-0.800576
C	1.903686	-1.018294	0.369207
N	-3.156759	0.023708	-0.697599
B	-0.581797	0.022430	-0.843849
C	1.433117	-2.277087	1.154465
C	0.051484	-2.076061	1.745846
H	-0.205635	-2.867436	2.457529
H	-0.734446	-2.073760	0.965045
H	-0.042903	-1.110986	2.261447
C	1.467432	-3.466583	0.200926
H	1.227547	-4.402140	0.718964
H	2.446776	-3.592842	-0.275594
H	0.714436	-3.346485	-0.604730
C	2.481146	-2.412630	2.301467
H	2.104653	-1.921115	3.203101
H	2.688000	-3.451223	2.565970
C	3.741208	-1.680554	1.807213
C	4.712118	-2.624089	1.110757
H	5.127161	-3.355240	1.815034
H	5.567716	-2.088213	0.666726
H	4.228845	-3.193352	0.302455
C	4.447411	-0.909223	2.908969
H	4.850993	-1.586498	3.671379
H	3.775000	-0.204637	3.422352
H	5.289179	-0.316180	2.524009
C	3.884617	0.381174	0.261525
C	3.815873	1.629561	0.925855
C	2.887232	1.880189	2.103681
H	2.550741	0.901755	2.537557
C	1.642156	2.625172	1.613351
H	0.926715	2.788592	2.424591
H	1.124236	2.049004	0.825761
H	1.886048	3.599704	1.179331
C	4.598270	2.687756	0.442432
H	4.550968	3.658788	0.935210
C	3.593523	2.652871	3.227429
H	2.966015	2.693839	4.124631
H	3.803457	3.689923	2.940543
H	4.546311	2.189515	3.501568
C	5.424291	2.518698	-0.668517
H	6.035374	3.347586	-1.023313
C	5.452779	1.295835	-1.336797
H	6.078394	1.180937	-2.221651

C	4.670709	0.214292	-0.901398
C	4.670944	-1.054269	-1.739276
H	3.990646	-1.816011	-1.279960
C	4.126272	-0.763216	-3.143145
H	4.083760	-1.673664	-3.748765
H	4.733809	-0.032914	-3.685715
H	3.102835	-0.358403	-3.086026
C	6.083471	-1.653536	-1.806029
H	6.082736	-2.602756	-2.352169
H	6.481325	-1.842026	-0.799470
H	6.786831	-0.987818	-2.318272
C	-1.930438	-0.805263	-0.684624
H	-1.910641	-1.432636	0.228423
C	-2.088033	-1.773976	-1.943142
C	-1.163659	-1.377957	-3.094273
H	-1.314885	-2.004369	-3.975612
H	-0.097561	-1.454025	-2.802108
H	-1.334714	-0.329145	-3.394634
C	-1.780794	-3.221448	-1.541784
H	-1.936774	-3.904943	-2.382779
H	-2.413731	-3.567134	-0.718045
H	-0.730441	-3.326982	-1.221796
C	-3.581304	-1.607550	-2.339150
H	-3.761530	-1.804553	-3.400759
H	-4.194214	-2.312216	-1.762437
C	-3.965742	-0.168349	-1.910591
C	-3.573585	0.853945	-2.985372
H	-4.027051	0.623942	-3.949914
H	-2.474691	0.877798	-3.112025
H	-3.865630	1.868983	-2.695772
C	-5.470555	-0.050633	-1.645288
H	-6.043814	-0.189566	-2.566204
H	-5.725117	0.937966	-1.242534
H	-5.831912	-0.790827	-0.914851
C	-3.739850	0.395076	0.569408
C	-3.695475	1.769399	0.920354
C	-3.064327	2.807997	0.010477
H	-2.516499	2.281848	-0.826897
C	-2.025359	3.647850	0.767573
H	-1.511842	4.334005	0.086971
H	-1.263124	2.998046	1.214619
H	-2.472620	4.243845	1.567620
C	-4.155244	3.696166	-0.605100
H	-3.724152	4.413421	-1.310031
H	-4.698161	4.263260	0.157232
H	-4.888057	3.089971	-1.150270
C	-4.250952	2.182601	2.138823

H	-4.229989	3.238079	2.405797
C	-4.824844	1.261789	3.015333
H	-5.247645	1.597433	3.958753
C	-4.855271	-0.089440	2.675648
H	-5.304334	-0.804302	3.362688
C	-4.425188	-2.023471	1.144427
H	-3.916906	-2.240879	0.167791
C	-4.323903	-0.540888	1.458108
C	-3.729333	-2.862382	2.226259
H	-3.668573	-3.914266	1.931888
H	-4.264672	-2.819193	3.181059
H	-2.711951	-2.500320	2.410613
C	-5.897378	-2.433807	0.987103
H	-5.991233	-3.492334	0.729217
H	-6.371293	-1.843841	0.184505
H	-6.473244	-2.261592	1.901811
C	1.600442	1.040519	-1.495734
H	2.587448	1.435388	-1.746800
C	0.533731	1.776926	-1.832403
C	0.423258	3.094149	-2.527697
H	1.416282	3.507093	-2.723024
H	-0.143290	3.801865	-1.912111
H	-0.109191	2.989181	-3.479312

References

1. T. J. Hadlington, J. Li and C. Jones, *Can. J. Chem.*, 2014, **92**, 427.
2. S. L. Taylor, D. Y. Lee and J. C. Martin, *J. Org. Chem.*, 1983, **48**, 4156.
3. M. Arrowsmith, J. Böhnke, H. Braunschweig, M. A. Celik, T. Dellermann and K. Hammond, *Chem. Eur. J.*, 2016, **22**, 17169.
4. J. Böhnke, H. Braunschweig, W. C. Ewing, C. Hörl, T. Kramer, I. Krummenacher, J. Mies and A. Vargas, *Angew. Chem. Int. Ed.*, 2014, **53**, 9082.
5. G. Sheldrick, *Acta Cryst.*, 2015, **A71**, 3.
6. G. Sheldrick, *Acta Cryst.*, 2008, **A64**, 112.
7. Gaussian 09, Revision C.01, M. J. Frisch, G. W. Trucks, H. B. Schlegel, G. E. Scuseria, M. A. Robb, J. R. Cheeseman, G. Scalmani, V. Barone, B. Mennucci, G. A. Petersson, H. Nakatsuji, M. Caricato, X. Li, H. P. Hratchian, A. F. Izmaylov, J. Bloino, G. Zheng, J. L. Sonnenberg, M. Hada, M. Ehara, K. Toyota, R. Fukuda, J. Hasegawa, M. Ishida, T. Nakajima, Y. Honda, O. Kitao, H. Nakai, T. Vreven, J. A. Montgomery, Jr., J. E. Peralta, F. Ogliaro, M. Bearpark, J. J. Heyd, E. Brothers, K. N. Kudin, V. N. Staroverov, R. Kobayashi, J. Normand, K. Raghavachari, A. Rendell, J. C. Burant, S. S. Iyengar, J. Tomasi, M. Cossi, N. Rega, J. M. Millam, M. Klene, J. E. Knox, J. B. Cross, V. Bakken, C. Adamo, J. Jaramillo, R. Gomperts, R. E. Stratmann, O. Yazyev, A. J. Austin, R. Cammi, C. Pomelli, J. W. Ochterski, R. L. Martin, K. Morokuma, V. G. Zakrzewski, G. A. Voth, P. Salvador, J. J. Dannenberg, S. Dapprich, A. D. Daniels, Ö. Farkas, J. B. Foresman, J. V. Ortiz, J. Cioslowski and D. J. Fox, *Gaussian*, Inc., Wallingford CT, 2009.
8. a) J. P. Perdew, K. Burke and M. Ernzerhof, *Phys. Rev. Lett.*, 1996, **77**, 3865; b) J. P. Perdew, K. Burke and M. Ernzerhof, *Phys. Rev. Lett.*, 1997, **78**, 1396; c) C. Adamo, V. Barone, *J. Chem. Phys.*, 1999, **110**, 6158.
9. S. Grimme, J. Antony, S. Ehrlich and H. Krieg, *J. Chem. Phys.*, 2010, **132**, 154104.
10. T. H. Dunning Jr. and P. J. Hay, in Methods of Electronic Structure Theory, H. F. Schaefer III, ed., Plenum Press, 1977, **2**.
11. Y. Zhao and D. G. Truhlar, *Theor. Chem. Acc.*, 2008, **120**, 215.
12. A. V. Marenich, C. J. Cramer and D. G. Truhlar, *J. Phys. Chem. B.*, 2009, **113**, 6378.
13. Q, Cui, H. Guo and M. Karplus, *J. Chem. Phys*., **2002**, *117*, 5617.
14. J. J. P. Stewart, *J. Mol. Model*., **2007**, *13*, 1173.

15. S. Schenker, C. Schneider, S. B. Tsogoeva and T. Clark, *J. Chem. Theory Comput.*, **2011**, *7*, 3586.
16. T. Vreven, K. S. Byun, I. Komaromi, S. Dapprich, J. Montgomery Jr., K. Morokuma and M. J. Frisch, *J. Chem. Theory Comput.*, 2006, **2**, 815.
17. F. Weigend and R. Ahlrichs, *Phys. Chem. Chem. Phys.*, 2005, **7**, 3297.
18. T. Onak, H. L. Landesman, R. E. Williams and I. Shapiro, *J. Phys. Chem.*, 1959, **63**, 1533.
19. D. Auerhammer, M. Arrowsmith, H. Braunschweig, R. D. Dewhurst, J. O. C. Jiménez-Halla and T. Kupfer, *Chem. Sci.*, 2017, **8**, 7066.
